# NeuroD1-USP1-MYCN axis drives tumor progression in neuroblastoma

**DOI:** 10.1186/s12967-026-07844-5

**Published:** 2026-02-11

**Authors:** Gen Li, Yanling Chen, Ran Zhuo, Juanjuan Yu, Jianping Bao, Di Wu, Hongli Yin, Xiaolu Li, Zhiheng Li, Chun Yang, Hairong Wang, Fang Fang, Yunyun Xu, Xiaohan Hu, Chenxi Feng, Mei Li, Lixiao Xu, Duancheng Guo, Li Zhang, Chaonan Zheng, Xiaodong Wang, Yuan Wang, Zimu Zhang, Jian Pan

**Affiliations:** 1 Institute of Pediatric Research, Children’s Hospital of Soochow University, Suzhou, 215025 China; 2 Pediatric Cancer Center, Jiangsu Key Laboratory of Neuropsychiatric Diseases, Department of Pharmacology, College of Pharmaceutical Sciences, Soochow University, Suzhou, 215123 China; 3 Pediatric Hematology & Oncology Key Laboratory of Higher Education Institutions in Jiangsu Province, Suzhou, 215025 China; 4 Department of Pediatric Surgery, Children’s Hospital of Soochow University, Suzhou, 215025 China; 5 Department of Neonatology, Children’s Hospital of Soochow University, Suzhou, 215025 China; 6 Cancer Institute, Fudan University Shanghai Cancer Center, Shanghai, 200032 China; 7 Department of Orthopedics, Children’s Hospital of Soochow University, Suzhou, 215025 China

**Keywords:** Neuroblastoma, NeuroD1, USP1, MYCN, Pimozide

## Abstract

**Background:**

Neuroblastoma, originating from the sympathetic neural crest, is the most prevalent extracranial solid tumor in children. Amplification of MYCN is a widely recognized indicator of poor prognosis in neuroblastoma. However, the structural properties of the N-Myc protein encoded by MYCN have impeded the development of direct inhibitors with favorable drug-like properties. This study aimed to investigate the upstream regulatory mechanisms of N-Myc stabilization in neuroblastoma and explore potential therapeutic strategies targeting these mechanisms.

**Methods:**

The regulatory role of NeuroD1 in neuroblastoma was evaluated through in vitro and in vivo experiments. Mechanistic studies were performed to examine the effects of NeuroD1 knockdown on N-Myc ubiquitination and degradation. Transcriptional target screening through RNAseq and ChIPseq was conducted to identify downstream effectors of NeuroD1, and the interaction between USP1 and N-Myc was assessed by co-IP and western blot. The therapeutic efficacy of Pimozide was investigated in neuroblastoma cells in vitro.

**Results:**

NeuroD1 was identified as a critical regulator associated with MYCN amplification. NeuroD1 promoted the proliferation of neuroblastoma cells in vitro and in vivo. Mechanistically, NeuroD1 knockdown increased K48-linked polyubiquitination of N-Myc, leading to its proteasomal degradation. USP1 was identified as a key downstream effector of NeuroD1 and was shown to interact with N-Myc, removing K48-linked polyubiquitin chains and stabilizing the protein. Pimozide effectively suppressed USP1 expression, reduced N-Myc levels, and inhibited neuroblastoma cell proliferation.

**Conclusion:**

This study uncovered a novel oncogenic axis in neuroblastoma, where NeuroD1 transcriptionally upregulates USP1, promoting N-Myc stabilization and tumor progression. Furthermore, the findings highlight the therapeutic potential of repurposing Pimozide as a promising treatment strategy for this aggressive tumor subtype.

**Supplementary Information:**

The online version contains supplementary material available at 10.1186/s12967-026-07844-5.

## Introduction

Neuroblastoma (NB), originating from sympathetic neural crest cells during early neural development [1], is the most common malignant extracranial solid tumor in children. Accounting for 10% of pediatric cancers and 15% of cancer-related deaths in children [2], it ranks as the third most prevalent pediatric tumor [3]. MYCN amplification, detected in 20–30% of neuroblastoma cases, is linked to severe malignancy and the worst clinical outcomes [4]. As such, MYCN amplification has become a key biomarker for tumor risk stratification [5]. However, the distinct structural characteristics of the N-Myc protein encoded by MYCN present significant challenges for drug development targeting this protein [6]. Thus, unraveling the upstream regulatory mechanisms of N-Myc in MYCN-amplified neuroblastoma could provide novel therapeutic targets, offering substantial potential for clinical application and improved treatment strategies.

NeuroD1 is an important transcription factor required for proper neural development [7, 9] and is critical in the differentiation of neural crest cells [10]. Its expression has been closely linked to adverse outcomes in neuroblastoma patients. NeuroD1 is aberrantly overexpressed in tumor tissues of NB mouse models, where it promotes NB cell survival and tumor sphere formation in vitro [11, 12]. Additional research has indicated that NeuroD1 promotes the proliferation of neuroblastoma cells by increasing ALK expression [13]. Moreover, N-Myc has been recognized as a direct transcriptional activator of NeuroD1, and the phenotypic changes in NB cells caused by N-Myc knockdown can be reversed by overexpressing NeuroD1 [14]. Collectively, these findings establish NeuroD1 as a critical regulator of NB pathogenesis, suggesting its involvement in MYCN regulation. However, the precise mechanisms through which NeuroD1 regulates MYCN remain to be elucidated.

In this investigation, we identified that NeuroD1 supports the growth of MYCN-amplified neuroblastoma by inhibiting the ubiquitin-proteasome degradation of N-Myc. K48-linked polyubiquitin chains serve as canonical degradation signals, targeting substrate proteins for proteasomal destruction [15]. In neuroblastoma, the E3 ubiquitin ligase FBXW7 marks N-Myc for proteasomal degradation through ubiquitination [16]. Conversely, deubiquitinating enzymes (DUBs) such as USP3 [17], USP5 [18], USP7 [19, 20], and USP36 [21] were reported to decrease K48-linked polyubiquitin modification from N-Myc, thereby stabilizing the protein and supporting neuroblastoma growth. Our investigation into NeuroD1’s transcriptional targets identified Ubiquitin Specific Protease1 (USP1) as a novel deubiquitinating enzyme that specifically targets N-Myc, providing new insights into the regulation of tumor progression.

USP1 is characterized by a conserved USP domain, a defining feature of the USP family of deubiquitinating enzymes [22]. Its primary role involves coordinating DNA damage response (DDR) elements [23] and affecting translesion synthesis [24]. USP1 is also critical for the initiation and advancement of several types of cancer. For example, it promotes aerobic glycolysis and facilitates the progression of T-cell acute lymphoblastic leukemia [25]. USP1’s deubiquitinase activity protects ID proteins (ID1/ID2/ID3) from proteasomal degradation in osteosarcoma, maintaining stem cell-like properties in tumor cells and driving malignancy [26]. Furthermore, a deubiquitinating enzyme screen identified USP1 as a regulator that deubiquitinates BCAT2 at lysine 229, enhancing PDAC cell proliferation [27]. These findings highlight the diverse pathways through which USP1 contributes to tumorigenesis. However, its role in neuroblastoma and its potential regulatory relationship with N-Myc remain unexplored. Interestingly, the FDA-approved USP1 inhibitor Pimozide [28] has shown effective USP1 inhibition and tumor growth suppression in various cancers [29, 32]. Therefore, elucidating the role and mechanisms of USP1 in regulating N-Myc in MYCN-amplified neuroblastoma could provide valuable insights and enable the rational design of novel therapeutics.

This research investigates NeuroD1’s role in neuroblastoma, unveiling for the first time the function and mechanism of the NeuroD1-USP1-MYCN regulatory axis in promoting tumor growth. These findings offer new insights into neuroblastoma and provide promising therapeutic targets and strategies for clinical treatment of this highly aggressive cancer.

## Materials and methods

### Cell culture and reagents

Details on the sources and culture conditions for all cell lines are available in Supplementary Tables S1 and S2. Mycoplasma testing was routinely conducted using the Mycolor One-Step Mycoplasma Detector (Vazyme, China) to ensure all cell cultures remained free of contamination. MG-132, Cycloheximide (CHX), and Doxycycline Hydrochloride (DOX) were purchased from Selleck (China). Pimozide was purchased from MedChemExpress (China). The antibodies are provided in the supporting information (Supplementary Table S3).

### Construction of stable knockdown and overexpression cell lines

NeuroD1-specific shRNA and scrambled shRNA plasmids, along with a USP1 overexpression plasmid tagged with GFP, were constructed by GENEWIZ (China). Lentiviral particles were generated as previously described [33]. The sequences for the shRNA are provided in the supporting information (Supplementary Table S4).

### RNA Extraction, qRT-PCR, and RNA sequencing (RNAseq)

Total RNA was extracted with TRIzol reagent (Invitrogen, USA) and subsequently converted to cDNA using the HiScript III All-in-one RT SuperMix (Vazyme) as per the manufacturer’s guidelines. Quantitative real-time PCR (qRT-PCR) was performed using 2×Taq Pro Universal SYBR qPCR Master Mix (Vazyme) on a Roche LightCycler^®^ 480 Instrument II. GAPDH served as the internal control for normalizing the relative mRNA expression levels. The primer sequences utilized are provided in the supporting information (Supplementary Table S5).

Gene expression profiles of both parental and NeuroD1 knockdown IMR-32 cells were analyzed using RNAseq, which was performed following the standard protocols provided by GENEWIZ.

### Immunoprecipitation and western blotting

Proteins were extracted as previously described [33]. Immunoprecipitation assays were conducted using BeaverBeads™ Protein A/G for Immunoprecipitation (BEAVER, China). Western blotting was performed as described previously [34]. Protein samples underwent SDS-PAGE separation and were subsequently transferred to PVDF membranes (Millipore, USA) via electrotransfer. For improved antibody detection, membranes were sectioned into strips according to the target proteins’ molecular weights.

### Chromatin immunoprecipitation sequence (ChIPseq) and PCR (ChIP-PCR)

The ChIP experiment followed the established protocol as previously described [33], with detailed procedures provided in the supporting information. ChIPseq was performed following the standard protocols provided by BGI Genomics Co., Ltd (Shenzhen, China).

ChIP samples were normalized to 1 ng for ChIP-PCR, performed with 2×Taq Pro Universal SYBR qPCR Master Mix and analyzed using the LightCycler^®^ 480 Instrument II. Relative enrichment levels were calculated and normalized to the input control. Primer sequences for the analysis are available in Supplementary Table S6 of the supporting information.

### In vivo procedure for NB xenograft preparation and NeuroD1 knockdown in nude mice

Neuroblastoma xenograft assays were conducted on 4-5-week-old female Balb/c-nude mice (Cavens Biogle Model Animal Research Co., Ltd, China) to examine the effects of NeuroD1 knockdown on tumor progression in vivo. Stable BE(2)-M17 cells expressing either shNC or shNeuroD1 were subcutaneously implanted into the axillary region of each mouse at a density of 2 × 10^6^ cells per mouse, with six mice per group. DOX was administered in drinking water at 2 mg/mL on the day of cell implantation. Tumor volumes were measured starting on day 10 post-implantation and subsequently every other day. Tumor volume was calculated using the formula L × S^2^/ 2, where L is the longest axis, and S is the shortest axis of the tumor, measured in millimeters. Mice were euthanized humanely with an intraperitoneal injection of a ketamine (120 mg/kg) and xylazine (150 mg/kg) solution (2:1 ratio) when tumors in the shNC group reached 1 × 10^3^ mm^3^. The xenografts were dissected, weighed, fixed, and paraffin-embedded for immunohistochemical analysis by Servicebio (China).

### Statistical analysis

Statistical analyses were performed using GraphPad Prism 9.0.0 software (GraphPad Software). Comparisons between two groups were evaluated using a two-tailed paired Student’s t-test. A p-value less than 0.05 was considered statistically significant (*p < 0.05, **p < 0.01). Data are expressed as mean ± standard deviation (SD).

## Results

### Elevated NeuroD1 expression is closely associated with poor prognosis in neuroblastoma

**Fig. 1:**
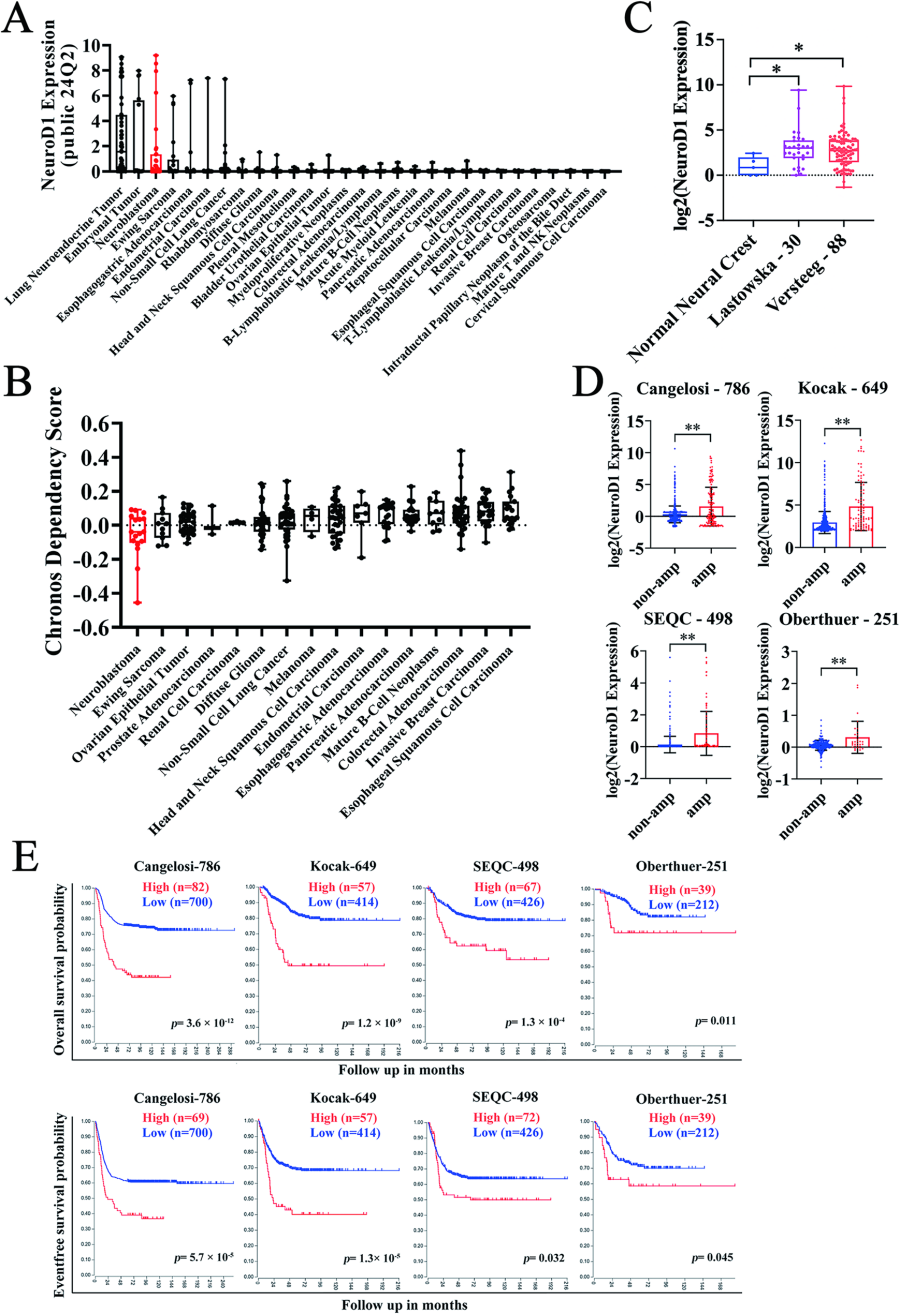
High NeuroD1 expression correlates with clinical malignancy in neuroblastoma. (**A**) NeuroD1 expression levels across various tumor cell lines were analyzed using the Dependency Map (DepMap) portal (https://depmap.org/portal/). Expression data from the 24Q2 release were utilized, and cell lines were ranked in descending order based on mean expression levels. (**B**) The dependency of different tumor cell lines on NeuroD1 was assessed using DepMap. The Chronos dependency score, derived from cell depletion assays, reflects gene essentiality; lower scores indicate higher dependency. Data from the 24Q2 release were ranked in ascending order of dependency scores. (**C**) NeuroD1 mRNA levels were compared between the normal neural crest dataset and two neuroblastoma datasets from the Gene Expression Omnibus (GEO) database: normal neural crest (GSE14340, *n* = 5), Lastowska-30 (Neuroblastoma, GSE13136, *n* = 30), and Versteeg-88 (Neuroblastoma, GSE16476, *n* = 88). (**D**) NeuroD1 mRNA was analyzed in MYCN-amplified (amp) and non-amplified (non-amp) neuroblastoma patient datasets. The datasets included were Cangelosi-786 (PMID: 32825087, amp *n* = 153, non-amp *n* = 629), Kocak-649 (GSE45547, amp *n* = 93, non-amp *n* = 550), SEQC-498 (GSE62564, amp *n* = 92, non-amp *n* = 401), and Oberthuer-251 (PMID: 17075126, amp *n* = 31, non-amp *n* = 220). (**E**) Overall and eventfree survival probability were analyzed in neuroblastoma datasets, comparing patients with elevated NeuroD1 expression (red line) to those with reduced NeuroD1 expression (blue line). Data are presented as mean ± SD. Statistical significance is indicated by *p < 0.05 and **p < 0.01

To explore the role of NeuroD1, we analyzed its expression across various cancer cell lines using the DepMap portal [35] and The Human Protein Atlas [36, 37]. Our results showed that NeuroD1 expression in neuroblastoma was significantly higher than in most other cancer types (Fig. 1A and Supplementary Fig. S1). Additionally, NeuroD1 had the lowest dependency score in neuroblastoma (Fig. 1B), highlighting its critical role in the onset and advancement of this disease. Given NeuroD1’s role in neural crest cell differentiation, which are the progenitors of neuroblastoma, we analyzed its expression in normal neural crest cells versus neuroblastoma patient datasets. NeuroD1 showed sustained overexpression in neuroblastoma patient samples relative to normal neural crest cells (Fig. 1C), suggesting its potential involvement in the tumor’s origin. Since MYCN amplification is a characteristic feature of high-risk neuroblastoma, we analyzed NeuroD1 expression in neuroblastoma patient datasets, distinguishing between those with and without MYCN amplification. NeuroD1 expression was notably higher in patients with MYCN amplification (Fig. 1D). Patients exhibiting elevated NeuroD1 expression demonstrated significantly poorer overall and eventfree survival probabilities compared to those with lower NeuroD1 expression (Fig. 1E). Collectively, these data establish NeuroD1 as a pivotal driver of neuroblastoma tumorigenesis and metastatic aggression.

### NeuroD1 sustains the development and progression of MYCN-amplified neuroblastoma in vivo and in vitro

**Fig. 2:**
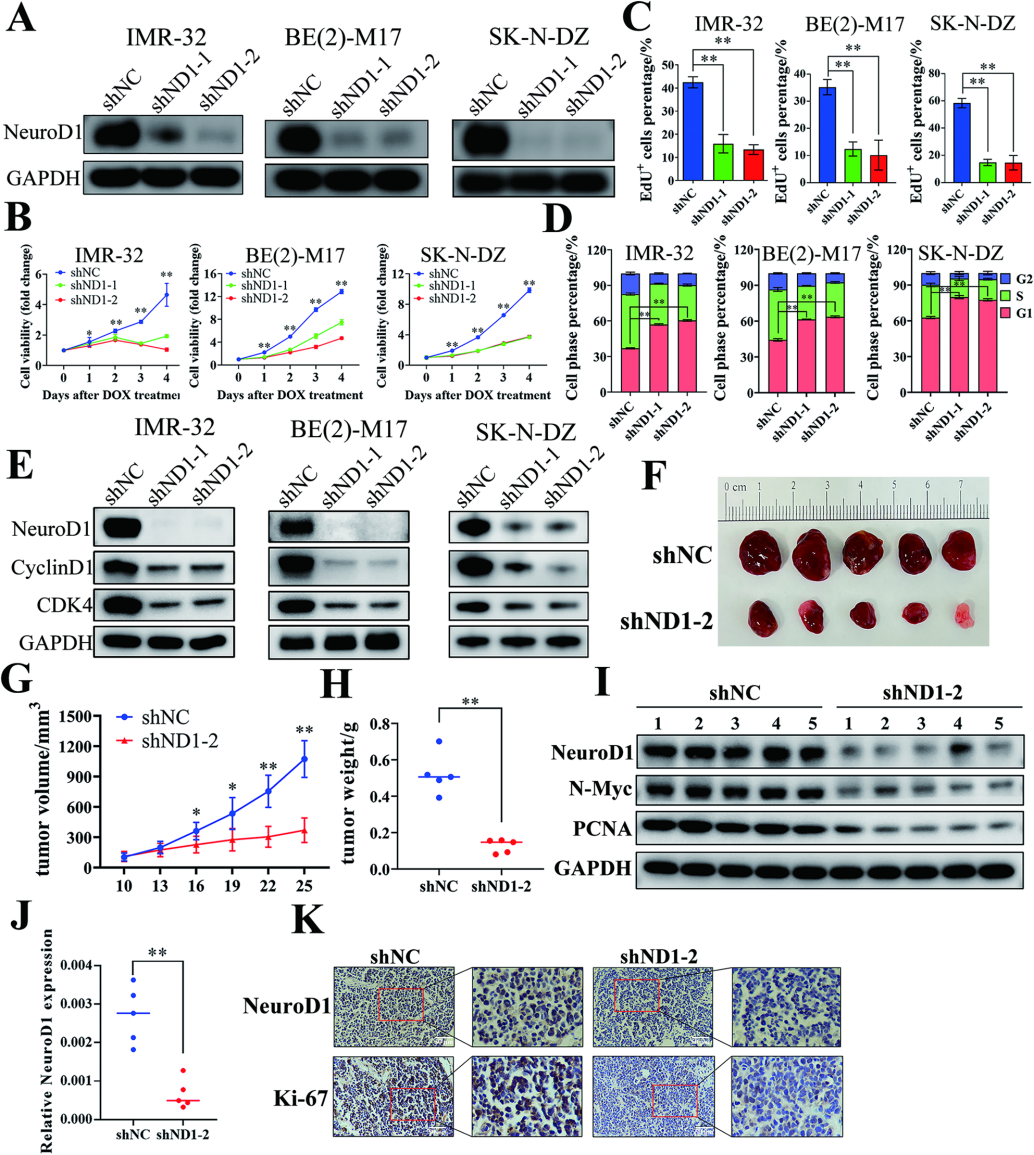
Knockdown of NeuroD1 suppresses the progression of MYCN-amplified neuroblastoma in vitro and *in vivo.* IMR-32, BE(2)-M17, and SK-N-DZ cells were stably transfected to express shNC (negative control), shND1-1 (shNeuroD1-1), and shND1-2 (shNeuroD1-2), respectively. (**A**) RNA interference was induced by treating cells with Doxycycline (1 µg/mL, 48 h). Western blot analysis was performed to assess the protein levels of NeuroD1 and GAPDH. (**B**) CCK-8 assay was used to assess cell viability at the time points shown in the figure. Cell proliferation rates were calculated by comparing the OD values on each day to those on day 0 (*n* = 3 per group). (**C**) RNA interference was induced with Doxycycline (1 µg/mL, 48 h), followed by EdU staining. The proportion of EdU-positive cells was determined by dividing the count of EdU-positive cells by the total cell number within a field of view (*n* = 3 per group). (**D**) RNA interference was induced with Doxycycline (1 µg/mL, 48 h). Cells were then collected, stained, and analyzed for cell cycle distribution by flow cytometry to determine the proportion of cells in each phase of the cell cycle (*n* = 3 per group). (**E**) Cells were treated with Doxycycline (1 µg/mL, 48 h), and total cellular protein was extracted. Western blot analysis was conducted to evaluate the protein levels of NeuroD1, CyclinD1, CDK4, and GAPDH. (**F**) BE(2)-M17 cells stably expressing shNC or shND1-2 were subcutaneously injected into the right forelimb axilla of nude mice (*n* = 5 per group). Doxycycline (2 mg/mL) was added to the drinking water of the mice after cell injection. Tumors were harvested on day 25, and tumor sizes were compared. (**G**) Tumor volumes were measured every two days starting on day 10 post-injection. (**H**) Tumors were harvested on day 25 post-injection, and their weights were measured in grams. (**I**) Protein was extracted from tumor tissues, and Western blot analysis was performed to measure the protein levels of NeuroD1, N-Myc, PCNA, and GAPDH. (**J**) RNA was extracted from tumor tissues, reverse transcribed, and analyzed by RT-qPCR to quantify NeuroD1 mRNA levels. GAPDH mRNA levels were used as an internal control. (**K**) Immunohistochemical staining was performed to detect NeuroD1 and Ki-67 protein levels in tumor tissues. Images were captured at 200× magnification under a microscope, with a white scale bar indicating 50 μm. Data are presented as mean ± SD. Statistical significance is indicated by *p < 0.05 and **p < 0.01

Given the strong clinical association between NeuroD1 expression and neuroblastoma with MYCN-amplification, we investigated the function of NeuroD1 in this tumor subtype. In MYCN-amplified neuroblastoma cell lines IMR-32, BE(2)-M17, and SK-N-DZ, NeuroD1 knockdown markedly reduced cell proliferation (Fig. 2A, B), suppressed DNA replication (Fig. 2C and Supplementary Fig. S2), and induced cell cycle arrest at the G1-S phase transition (Fig. 2D and Supplementary Fig. S3). CyclinD1 and CDK4, key regulators of the G1-to-S phase transition [38], were downregulated at the protein level following NeuroD1 knockdown (Fig. 2E), further supporting the essential function of NeuroD1 in driving cell cycle progression. Subcutaneous xenograft experiments in nude mice were conducted to assess NeuroD1’s impact on tumor progression in vivo. Knockdown of NeuroD1 significantly slowed tumor growth, resulting in markedly smaller tumors at the endpoint (Fig. 2F-H and Supplementary Fig. S4). Tumor tissues from the NeuroD1 knockdown group exhibited significantly reduced NeuroD1 protein and mRNA levels, along with a reduction of the proliferation markers PCNA and Ki-67 (Fig. 2I-K), further confirming the suppressive effect of NeuroD1 knockdown on tumor growth in vivo. In summary, the findings indicate that NeuroD1 is crucial for maintaining the advancement of MYCN-amplified neuroblastoma in both in vitro and in vivo settings.

### NeuroD1 knockdown suppresses N-Myc protein levels in MYCN-amplified neuroblastoma

**Fig. 3:**
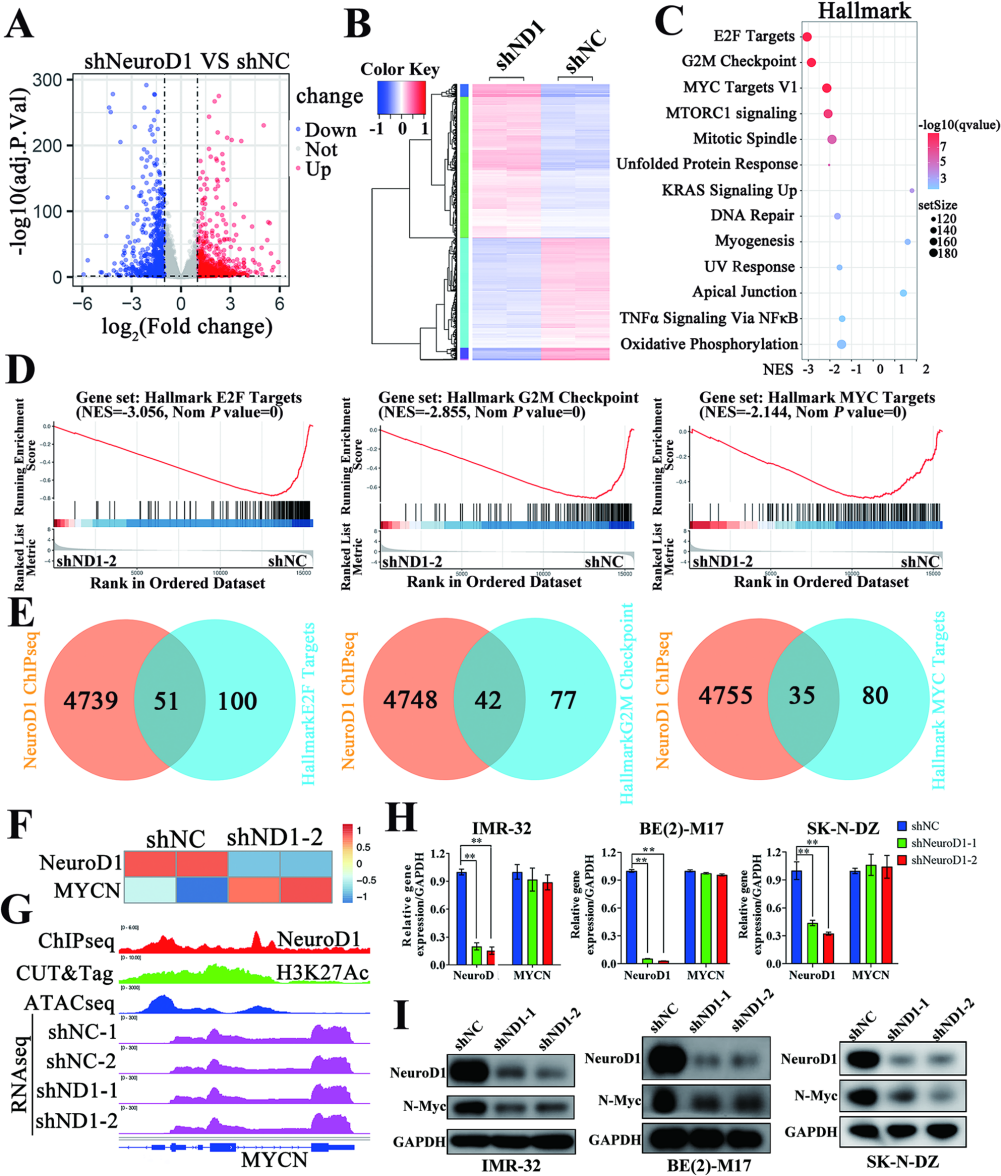
NeuroD1 is positively associated with N-Myc and its regulatory genes in MYCN-amplified neuroblastoma. (**A**) The volcano plots depict differentially expressed genes between the shNC (negative control) and shND1 (shNeuroD1) groups identified through RNA-seq. ach point represents a gene, where blue points denote significantly downregulated genes, and red points indicate significantly upregulated genes in IMR-32 cells. Thresholds: log2FoldChange < -1 or > 1 with an adjusted *p* < 0.05. (**B**) A heatmap illustrates the differential gene expression between the shNC and shND1 groups in IMR-32 cells. (**C**,** D**) Gene Set Enrichment Analysis (GSEA) was performed to explore the functional roles of NeuroD1 in IMR-32 cells. (**E**) The Venn diagram highlights the overlap between genes located at NeuroD1-binding sites (from ChIP-seq data) and Hallmark pathway genes. (**F**) A heatmap shows the mRNA expression levels of NeuroD1 and MYCN in the shNC and shND1 groups of IMR-32 cells. (**G**) IGV tracks display ChIP-seq, CUT&Tag for indicated antibodies, ATAC-seq, and RNA-seq data from shNC and shND1 groups, focusing on the MYCN locus in the genome. (**H**) RT-qPCR quantified the mRNA levels of NeuroD1 and MYCN in IMR-32, BE(2)-M17, and SK-N-DZ cells across shNC, shND1-1, and shND1-2 groups, with GAPDH mRNA level used as an internal control. (**I**) Western blot showed NeuroD1, N-Myc, and GAPDH protein levels in IMR-32, BE(2)-M17, and SK-N-DZ cells from shNC, shND1-1, and shND1-2 groups. Data was presented as mean ± SD. Statistical significance is indicated by *p < 0.05 and **p < 0.01

We conducted transcriptomic sequencing on parental and NeuroD1-knockdown IMR-32 cells to investigate how NeuroD1 facilitates proliferation. Knockdown of NeuroD1 resulted in the downregulation of 731 genes and the upregulation of 923 genes (Fig. 3A, B, and Supplementary Table S7), highlighting its critical role in cellular processes. GO and KEGG pathway analyses of the downregulated genes showed significant enrichment in DNA replication, G1-to-S phase transition, and cell cycle checkpoint pathways (Supplementary Fig. S5A, B), aligning with the phenotypic effects observed following NeuroD1 knockdown.

GSEA analysis indicated that the downregulated genes were significantly linked to hallmark pathways such as E2F Targets, G2M Checkpoint, and MYC Targets (Fig. 3C, D, and Supplementary Table S8). To determine whether these genes are directly regulated by NeuroD1, we performed ChIP-seq analysis in IMR-32 cells (Supplementary Fig. S6 and Supplementary Table S9). Surprisingly, only a small proportion of NeuroD1-bound genes overlapped with hallmark genes in these pathways (Fig. 3E), suggesting that NeuroD1 indirectly influences these targets. Interestingly, hallmark genes in E2F Targets, G2M Checkpoint, and MYC Targets pathways are strongly associated with MYCN in MYCN-amplified neuroblastoma [39, 40]. ChIP-seq data confirmed NeuroD1 binding within the MYCN genomic region, marked by high levels of H3K27Ac modification and open chromatin structure. However, NeuroD1 knockdown did not alter the mRNA level of MYCN (Fig. 3F-H). Notably, NeuroD1 knockdown markedly decreased N-Myc protein expression (Fig. 3I). These findings demonstrate that NeuroD1 regulates N-Myc protein stability without impacting MYCN mRNA expression, underscoring its critical role in tumor progression.

### NeuroD1 deubiquitinates and stabilizes N-Myc protein

**Fig. 4:**
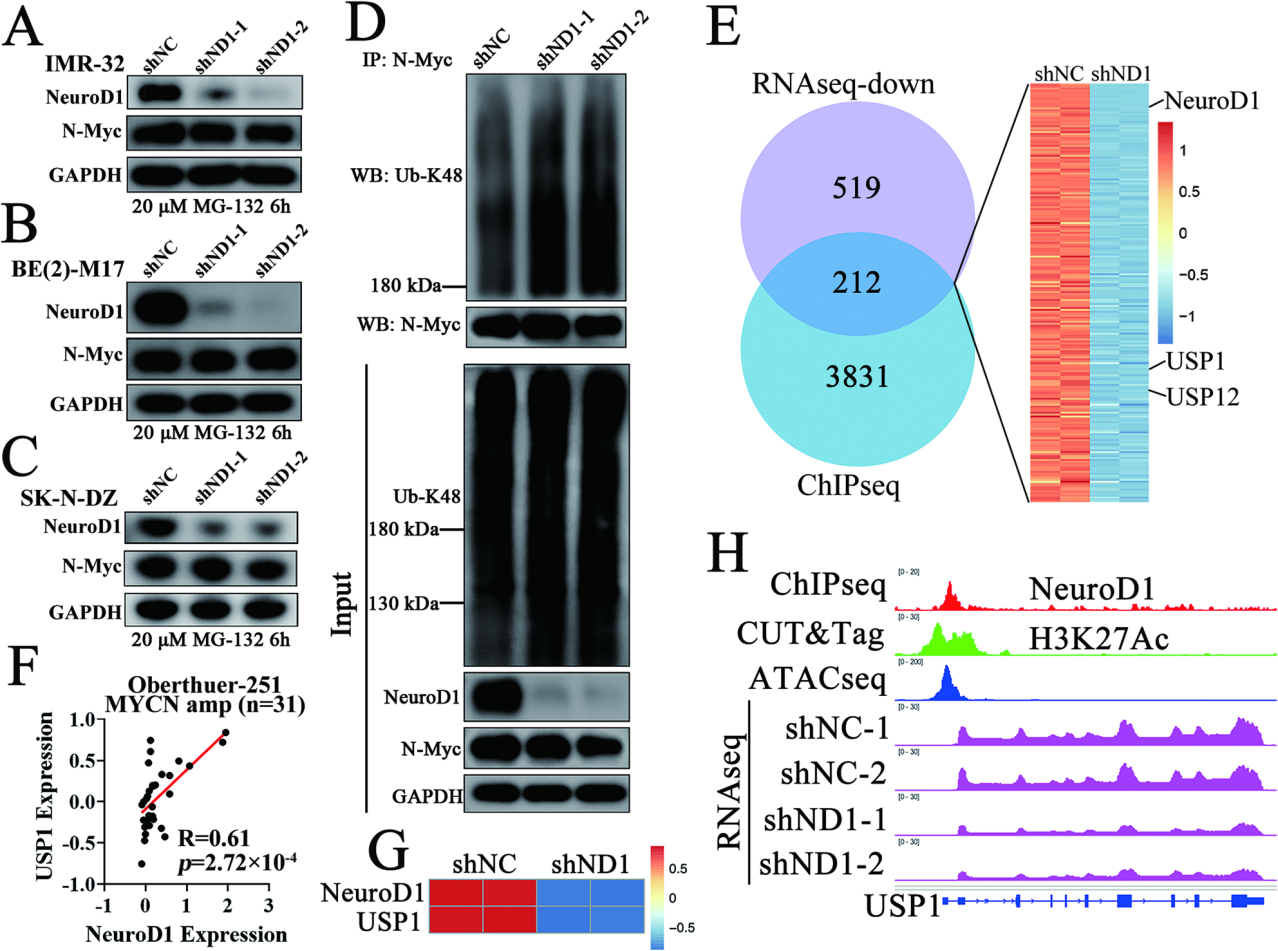
NeuroD1 inhibits N-Myc ubiquitin-proteasome degradation and positively correlates with USP1 expression. (**A-C**) IMR-32, BE(2)-M17, and SK-N-DZ cells were stably transduced with shNC (negative control), shND1-1 (shNeuroD1-1), and shND1-2 (shNeuroD1-2). RNA interference was induced by treating cells with Doxycycline (1 µg/mL, 48 h), followed by the addition of MG-132 (20 µM, 6 h). Western blot was performed to detect the levels of NeuroD1, N-Myc, and GAPDH. (**D**) 293FT cells were transfected with shNC, shND1-1, or shND1-2 constructs. After induction with Doxycycline (1 µg/mL, 48 h), cells were treated with MG-132 (20 µM, 6 h). Total protein was extracted, and immunoprecipitation (IP) with an N-Myc antibody was performed. Western blot was used to detect the indicated proteins. The input group represents whole-cell lysates. (**E**) The Venn diagram and heatmap illustrate the overlap between genes located at NeuroD1-binding sites identified by ChIP-seq and genes downregulated in RNA-seq following NeuroD1 knockdown. (**F**) Correlation analysis of NeuroD1 and USP1 mRNA levels in MYCN-amplified neuroblastoma patients (*n* = 31) was performed using the Oberthuer-251 dataset. (**G**) The heatmap shows the mRNA levels of NeuroD1 and USP1 in IMR-32 cells from the shNC and shND1 groups. (**H**) IGV tracks visualize ChIP-seq, CUT&Tag for indicated antibodies, ATAC-seq, and RNA-seq data for shNC and shND1 groups around the USP1 locus in the genome

Based on our findings, we hypothesized that NeuroD1 knockdown induces ubiquitin-proteasome-mediated degradation of N-Myc protein. To test this, we treated NeuroD1-knockdown cells with the proteasome inhibitor MG-132. MG-132 treatment effectively restored N-Myc protein levels, which were diminished due to NeuroD1 knockdown (Fig. 4A-C). Co-immunoprecipitation assays revealed that NeuroD1 knockdown resulted in increased K48-linked polyubiquitin chains on N-Myc (Fig. 4D), indicating that NeuroD1 modulates N-Myc stability through the ubiquitin-proteasome pathway. Given that NeuroD1 functions as a transcription factor and lacks direct deubiquitinating activity, we sought to identify potential DUBs that might mediate this effect. Through integrative analysis of ChIP-seq and transcriptomic data, we identified USP1 and USP12 as potential candidates (Fig. 4E). Further analysis of expression profiles from MYCN-amplified neuroblastoma patient datasets revealed a stronger correlation between NeuroD1 and USP1 expression compared to USP12 (Fig. 4F and Supplementary Fig. S7), suggesting a more prominent regulatory relationship between NeuroD1 and USP1 in MYCN-amplified neuroblastoma. Additionally, transcriptomic data demonstrated that NeuroD1 knockdown significantly reduced USP1 transcription in IMR-32 cells. ChIP-seq analysis confirmed that NeuroD1 binds directly to the USP1 promoter region, which also exhibits high levels of H3K27Ac marks and open chromatin indicative of active transcription (Fig. 4G, H). These results indicate that NeuroD1 stabilizes N-Myc protein by preventing its ubiquitin-proteasome degradation, likely through the transcriptional activation of USP1. This highlights USP1 as a critical downstream target of NeuroD1.

### Identification of USP1 as a direct target gene of NeuroD1

**Fig. 5:**
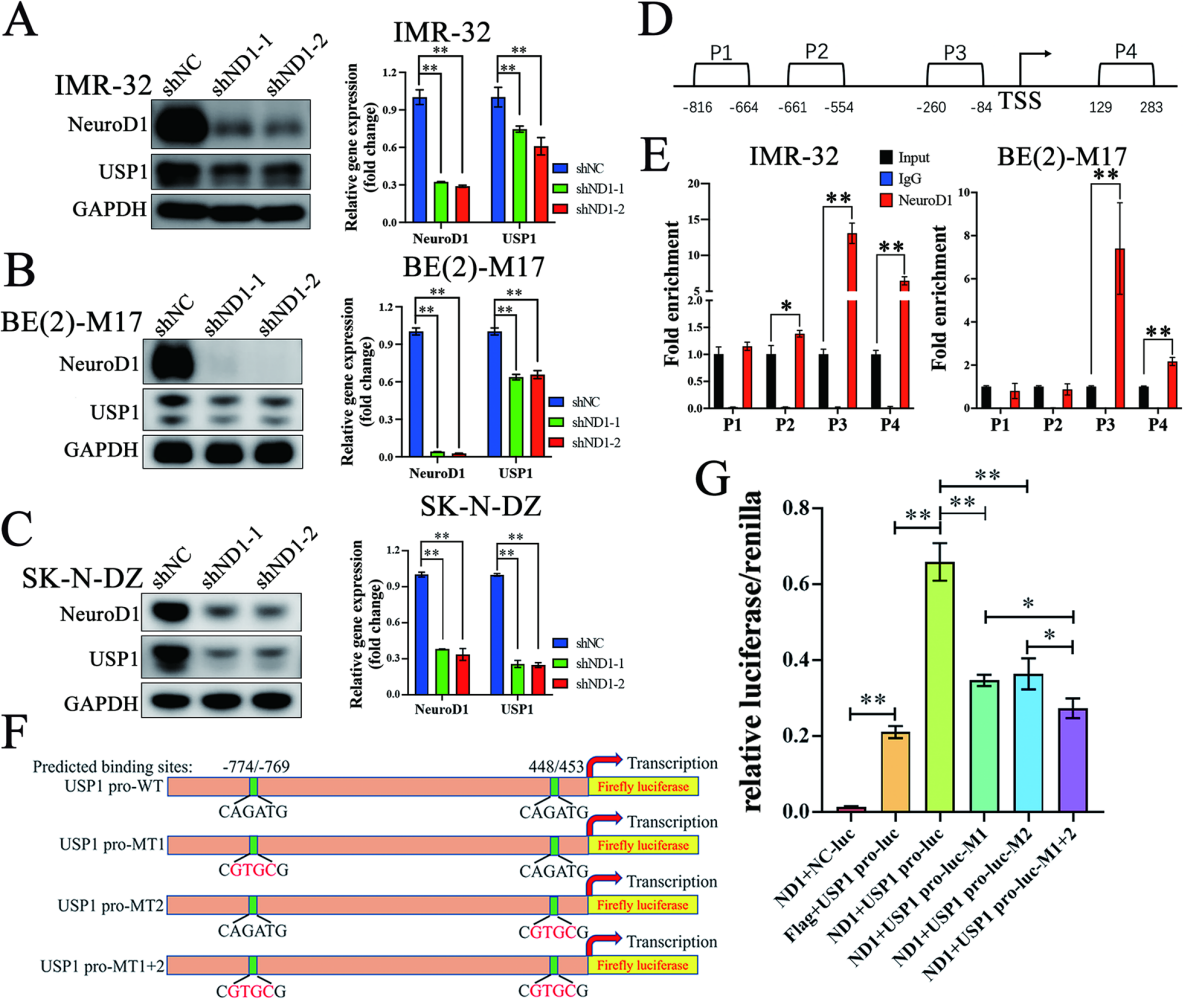
NeuroD1 binds to the USP1 promoter region and transcriptionally activates USP1. (**A-C**) IMR-32, BE(2)-M17, and SK-N-DZ cells were stably transfected with shNC (negative control), shND1-1 (shNeuroD1-1), and shND1-2 (shNeuroD1-2). RNA interference was induced by treating cells with Doxycycline (1 µg/mL, 48 h). Protein levels of NeuroD1, USP1, and GAPDH were assessed by Western blot, and mRNA levels of NeuroD1, USP1, and GAPDH were quantified via RT-qPCR. (**D**) Schematic diagram showing the genomic location of PCR primers targeting the USP1 promoter region. TSS: transcriptional start site. (**E)** ChIP assays were performed in IMR-32 and BE(2)-M17 cells using a NeuroD1 antibody. RT-qPCR was conducted with primers targeting the USP1 promoter region, as shown in **D**, to evaluate the binding of NeuroD1 to the USP1 promoter. (**F**) Schematic representation of USP1 promoter-driven luciferase reporter constructs: USP1 pro-WT (wild-type USP1 promoter), USP1 pro-MT1 (mutation at site 1), USP1 pro-MT2 (mutation at site 2), and USP1 pro-MT1 + 2 (mutations at both site 1 and site 2). (**G**) 293FT cells were transfected with the indicated luciferase reporter constructs. After 24 h, dual-luciferase assays were performed, with Renilla luciferase activity serving as an internal control. ND1: pLVX-EF1a-3×flag-NeuroD1; flag: pLVX-EF1a-3×flag; NC-luc: luciferase vector lacking the USP1 promoter. Data was presented as mean ± SD. Statistical significance is indicated by *p < 0.05 and **p < 0.01

To confirm whether NeuroD1 directly regulates the transcription of USP1, we further investigated its regulatory relationship with USP1. Silencing of NeuroD1 in MYCN-amplified neuroblastoma resulted in a marked reduction in both USP1 protein and mRNA levels (Fig. 5A-C), indicating that NeuroD1 modulates USP1 transcription.

Previous ChIP-seq analysis revealed strong enrichment of NeuroD1 in the promoter region of USP1. To validate this observation, we designed primers targeting various sites within the USP1 promoter and performed ChIP-qPCR. The results confirmed significant enrichment of NeuroD1 at the USP1 promoter region in MYCN-amplified neuroblastoma cells (Fig. 5D, E). Furthermore, two NeuroD1-binding motifs were identified within the USP1 promoter region. To assess if NeuroD1 directly activates USP1 transcription, we developed luciferase reporter plasmids with either the wild-type USP1 promoter or variants containing single or double mutations in the NeuroD1-binding sites. NeuroD1 overexpression significantly increased luciferase activity from the wild-type promoter, whereas mutations in either motif substantially diminished this enhancement. Double mutations completely abolished NeuroD1-mediated transcriptional activation (Fig. 5F, G). These findings demonstrate that NeuroD1 directly transcribes USP1 by binding to specific motifs in its promoter, highlighting the pivotal role of the NeuroD1/USP1 axis in the progression of MYCN-amplified neuroblastoma.

### USP1 stably binds to N-Myc

**Fig. 6:**
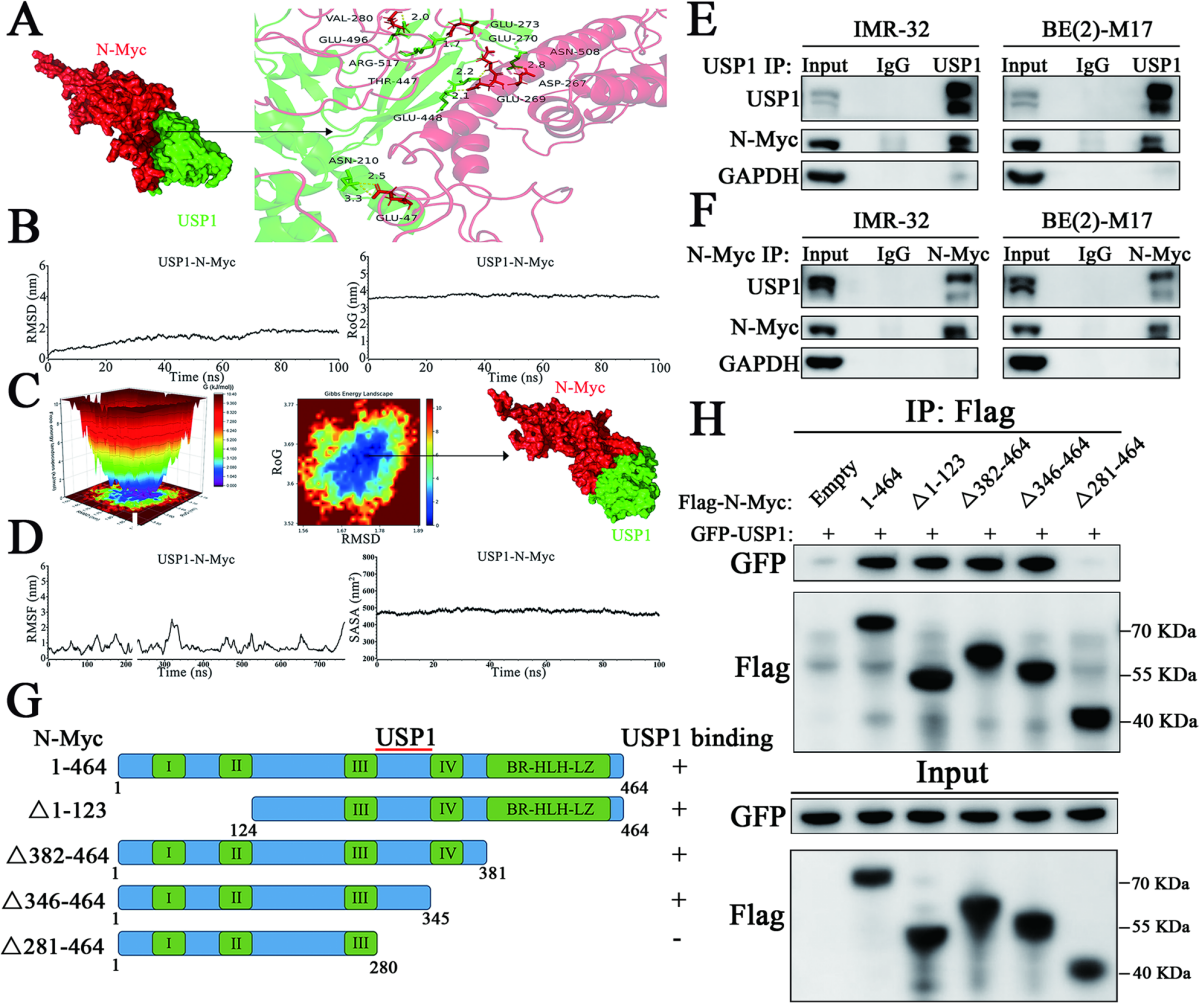
USP1 directly binds to N-Myc protein in MYCN-amplified neuroblastoma. (**A**) The binding model of the USP1-N-Myc complex is shown, with a detailed view of the interaction on the right. (**B**) Root mean square deviation (RMSD) and radius of gyration (RoG) values were calculated from 100 ns-long molecular dynamics simulation trajectories to evaluate the stability of the complex. (**C**) The free energy landscape based on RMSD and RoG results illustrates the stable binding between USP1 and N-Myc. (**D**) Root mean square fluctuation (RMSF) and solvent-accessible surface area (SASA) analyses further confirm the stability of the USP1-N-Myc interaction. (**E**) Immunoprecipitation (IP) was performed in IMR-32 and BE(2)-M17 cells using a USP1 antibody or an isotype-matched IgG control, followed by Western blot analysis to detect USP1, N-Myc, and GAPDH protein levels. The input group represents whole-cell lysates. (**F**) Reciprocal IP was performed in IMR-32 and BE(2)-M17 cells using an N-Myc antibody or an isotype-matched IgG control, followed by Western blot analysis to detect USP1, N-Myc, and GAPDH protein levels. The input group represents whole-cell lysates. (**G**) Schematic diagram of the full-length N-Myc protein and its truncation mutants. Numbers following ∆ indicate the deleted amino acid regions. (**H**) 293FT cells were transfected with GFP-tagged USP1 and Flag-tagged N-Myc constructs (Empty indicates the Flag-empty vector). GFP immunoprecipitation was performed using a GFP antibody, followed by Western blot analysis to detect GFP and Flag proteins. The input group represents whole-cell lysates

Previous studies have shown that in neuroblastoma, the N-Myc protein can be deubiquitinated by USP3 [17], USP5 [18], USP7 [19, 20], and USP36 [21], which remove K48-linked polyubiquitin chains to prevent proteasomal degradation. However, our RNA-seq and ChIP-seq analyses did not identify these DUBs. The regulatory relationship between USP1, our candidate gene, and N-Myc protein remains unexplored. To investigate, we first simulated the interaction stability between USP1 and N-Myc through protein docking. The N-Myc structure was derived from prior experiments, and the USP1 structure was obtained from the PDB database. Protein docking analysis revealed that USP1 could bind to N-Myc with a binding energy of -519 kJ/mol (Fig. 6A). We further assessed the stability of the USP1-N-Myc interaction using molecular dynamics (MD) simulations. Root-mean-square deviation (RMSD) analysis indicated that the USP1-N-Myc complex reached a stable state after approximately 70 ns of simulation, and the radius of gyration (RoG) analysis corroborated this finding (Fig. 6B). Free energy landscape (FEL) mapping based on RMSD and RoG data revealed a single energy well, indicating a stable binding state. Examination of the lowest energy conformation confirmed that USP1-N-Myc binding was stable, with no significant displacement compared to the docking conformation (Fig. 6C). Further analyses of RMSF and SASA also supported these findings (Fig. 6D). Together, these results suggest that USP1 can stably bind to N-Myc and form a robust binary complex.

To confirm this interaction in MYCN-amplified neuroblastoma cells, Co-IP experiments demonstrated that endogenous USP1 and N-Myc proteins physically interact, providing direct evidence of USP1-mediated regulation of N-Myc (Fig. 6E, F). To pinpoint the specific region of N-Myc responsible for USP1 binding, we constructed plasmids expressing either full-length N-Myc or various deletion mutants (Fig. 6G). Co-IP experiments demonstrated that exogenous USP1 can bind to full-length N-Myc (1–464) and its truncated forms (Δ1–123), N-Myc (Δ382–464), and N-Myc (Δ346–464) (Fig. 6H, lines 2–5). However, N-Myc (Δ281–464) completely lost its ability to bind USP1 under the same conditions (Fig. 6H, line 6). These findings demonstrate that USP1 physically interacts with N-Myc and that the 281–464 amino acid region of N-Myc is essential for this interaction.

### USP1 deubiquitinates and stabilizes N-Myc

**Fig. 7:**
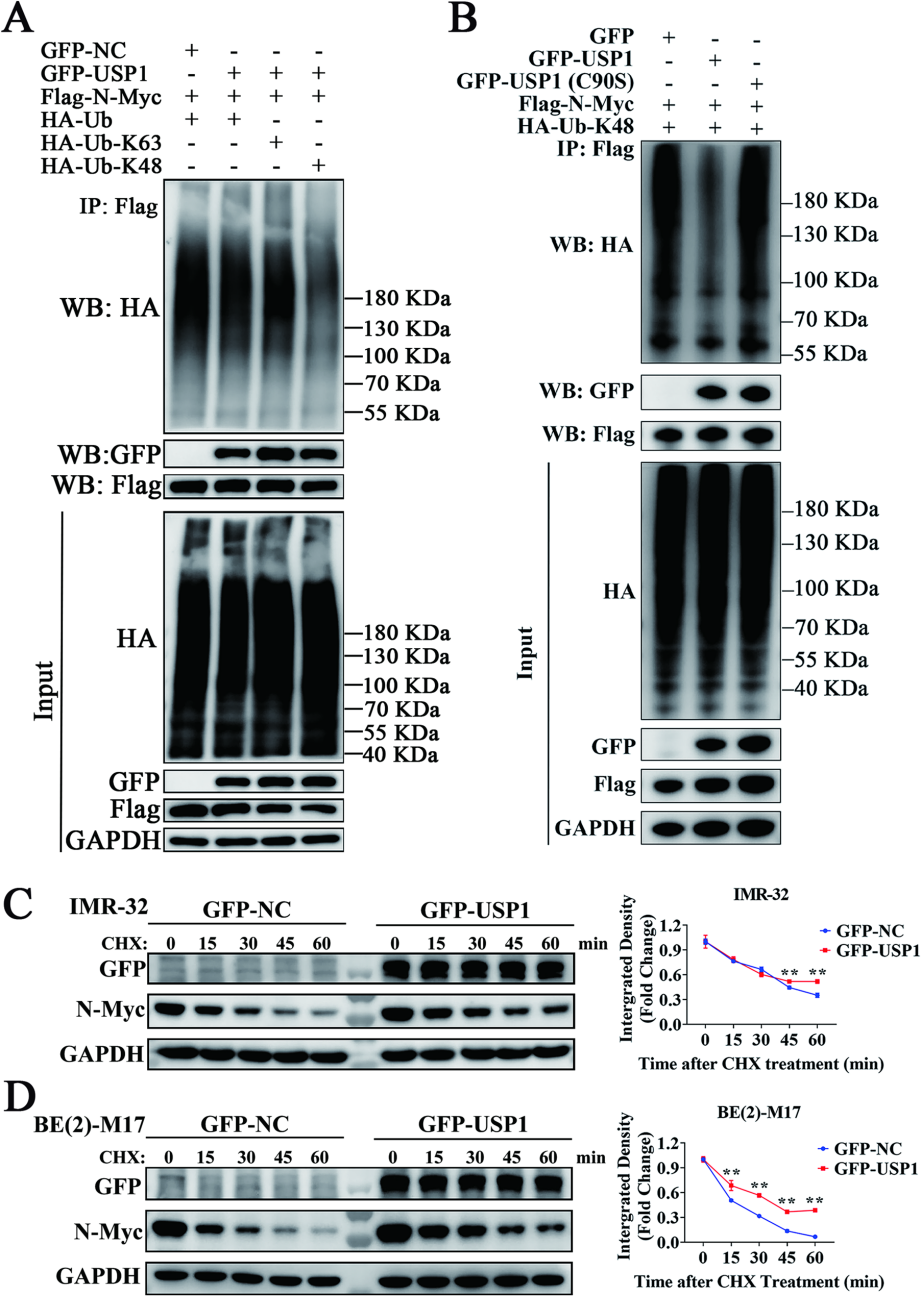
USP1 inhibits K48-linked polyubiquitination of N-Myc and extends its half-life in MYCN-amplified neuroblastoma. (**A**) 293FT cells were transfected with the indicated plasmids. GFP-NC represents the GFP empty vector, GFP-USP1 represents GFP-tagged USP1, Flag-N-Myc represents Flag-tagged N-Myc, HA-Ub represents HA-tagged ubiquitin, HA-Ub-K48 represents the HA-tagged ubiquitin mutant (retaining only lysine 48), and HA-Ub-K63 represents the HA-tagged ubiquitin mutant (retaining only lysine 63). “+” indicates plasmid transfection, while “-” indicates no transfection. After 24 h of transfection, cells were treated with MG-132 (20 µM, 6 h). Total cell protein was collected, and IP was performed, followed by Western blotting to detect the indicated proteins. The input group represents whole-cell lysates. (**B**) 293FT cells were transfected with the indicated plasmids. GFP-NC represents the GFP empty vector, GFP-USP1 represents GFP-tagged USP1, GFP-USP1 (C90S) represents the catalytically inactive USP1 mutant (C90S; cysteine at position 90 mutated to serine), Flag-N-Myc represents Flag-tagged N-Myc, and HA-Ub-K48 represents the HA-tagged ubiquitin mutant (retaining only lysine 48). “+” indicates plasmid transfection, while “-” indicates no transfection. After 24 h of transfection, cells were treated with MG-132 (20 µM, 6 h). Total cell protein was collected, and IP was performed using a Flag antibody, followed by Western blotting to detect the indicated proteins. The input group represents whole-cell lysates. (**C**,** D**) IMR-32 and BE(2)-M17 cells were transfected with GFP-NC (GFP empty vector) or GFP-USP1 (GFP-tagged USP1). After 48 h of transfection, cells were treated with cycloheximide (CHX, 100 µg/mL). Protein levels of GFP, N-Myc, and GAPDH were analyzed by Western blot at time points 0, 15, 30, 45, and 60 min. The densitometric analysis of N-Myc bands is shown on the right. The integrated density of the N-Myc band was normalized to the corresponding GAPDH band to calculate the “Integrated Density.” The relative integrated density for each time point was expressed as a fold change relative to the 0-minute time point for the GFP-NC or GFP-USP1 groups. Data was presented as mean ± SD. Statistical significance is indicated by *p < 0.05 and **p < 0.01

To examine USP1’s involvement in modulating N-Myc ubiquitination, we co-expressed USP1, N-Myc, and either wild-type or mutant Ub (K48: retaining only lysine-48 to form K48-linked polyubiquitination, or K63: retaining only lysine-63 to form K63-linked polyubiquitination) in 293FT cells. Co-IP revealed significant ubiquitination of N-Myc, which was partially reduced by USP1 overexpression (Fig. 7A, lines 1–2). Notably, USP1 preferentially removed K48-linked polyubiquitination compared to K63-linked modifications on N-Myc (Fig. 7A, line 3–4). To further confirm that the effect of USP1 on N-Myc ubiquitination depends on its enzymatic activity, we generated a catalytic mutant of USP1 (C90S) by substituting cysteine-90 with serine, as previously described [23]. Co-IP experiments showed that both wild-type USP1 and USP1(C90S) could bind to N-Myc; however, USP1(C90S) failed to remove K48-linked polyubiquitination from N-Myc (Fig. 7B). Finally, we assessed the impact of USP1 on the stability of N-Myc protein. Protein half-life assays demonstrated that USP1 overexpression markedly increased N-Myc protein stability (Fig. 7C, D). In conclusion, USP1 stabilizes N-Myc protein by enzymatically removing K48-linked polyubiquitination, thereby preventing its proteasomal degradation.

### Pimozide inhibits USP1 and proliferation in MYCN-amplified neuroblastoma

**Fig. 8:**
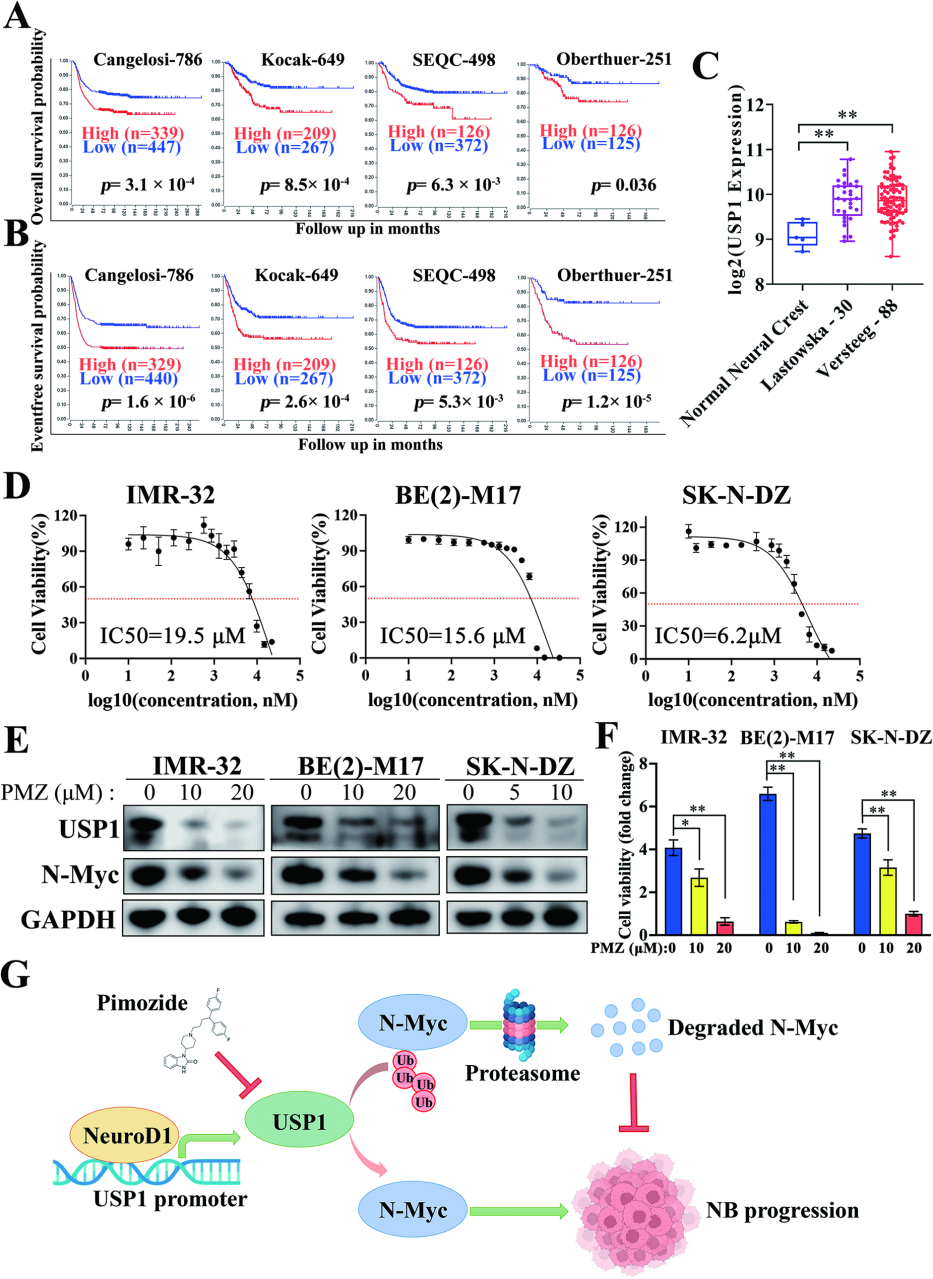
Pimozide suppresses USP1 protein levels and cell proliferation in MYCN-amplified neuroblastoma. (**A**,** B**) Kaplan-Meier survival analyses were conducted to compare overall and eventfree survival probability between neuroblastoma patients with high USP1 expression (red line) and low USP1 expression (blue line). The datasets included were Cangelosi-786 (PMID: 32825087), Kocak-649 (GSE45547), SEQC-498 (GSE62564), and Oberthuer-251 (PMID: 17075126). (**C**) USP1 mRNA levels were compared between normal neural crest cells and neuroblastoma samples using datasets from the Gene Expression Omnibus (GEO) database: normal neural crest (GSE14340, *n* = 5), Lastowska-30 (neuroblastoma, GSE13136, *n* = 30), and Versteeg-88 (neuroblastoma, GSE16476, *n* = 88). (**D**) IMR-32, BE(2)-M17, and SK-N-DZ were treated with Pimozide (PMZ) for 48 h. The half-maximal inhibitory concentrations (IC50) were determined to be 19.5 µM for IMR-32, 15.6 µM for BE(2)-M17, and 6.2 µM for SK-N-DZ. (**E**) IMR-32, BE(2)-M17, and SK-N-DZ cells were treated with the indicated concentrations of Pimozide for 48 h. Western blot analysis was performed to measure the protein levels of USP1, N-Myc, and GAPDH. (**F**) IMR-32, BE(2)-M17, and SK-N-DZ cells were treated with the indicated concentrations of Pimozide (PMZ) for 48 h. Cell viability was assessed using the CCK-8 assay, with results expressed as fold changes relative to the cell viability at 0 h within the same treatment group. (**G**) Schematic diagram of the mechanism in this study. Data was presented as mean ± SD. Statistical significance is indicated by *p < 0.05 and **p < 0.01

Our previous studies demonstrated the regulatory role of NeuroD1 in USP1 expression and the stabilization of N-Myc by USP1. To further explore the function of USP1, we first examined the protein levels of USP1 in patient tissues and found that its expression was higher in samples from two MYCN-amplified cases compared to eight non-amplified cases (Supplementary Fig. S8A), preliminarily supporting a link between USP1 and MYCN status. To address the limitation of small sample size and enhance statistical robustness, we analyzed independent neuroblastoma gene expression datasets. The results confirmed a positive correlation between USP1 mRNA levels and both MYCN and the proliferation marker MKI67 in MYCN-amplified tumors (Supplementary Fig. S8B, C), reinforcing the significant role of USP1 in this aggressive subtype. Subsequently, we observed that Elevated USP1 expression was closely linked to reduced overall survival and eventfree survival when compared to lower USP1 expression (Fig. 8A, B). Furthermore, USP1 expression was markedly higher in tumor samples compared to neural crest cells, the origin of neuroblastoma, across two independent datasets (Fig. 8C), indicating a potential oncogenic role for USP1 in neuroblastoma development and progression.

Pimozide, an FDA-approved USP1 inhibitor, has demonstrated efficacy in inhibiting USP1 activity and tumor growth across various cancers [29, 32]. Pimozide demonstrated potent inhibition of MYCN-amplified neuroblastoma cells, with 48 h IC50 values of 19.5 µM for IMR-32, 15.6 µM for BE(2)-M17, and 6.2 µM for SK-N-DZ cells (Fig. 8D). Pimozide dose-dependently inhibited USP1 and N-Myc proteins, significantly decreasing tumor cell viability (Fig. 8E, F). These results highlight the pivotal function of USP1 in promoting the progression of MYCN-amplified neuroblastoma. By targeting USP1, Pimozide effectively reduces its levels and inhibits tumor cell proliferation, presenting a promising therapeutic approach for aggressive neuroblastoma.

## Discussion

Neuroblastoma is the most prevalent extracranial solid tumor in children and the primary cause of cancer-related mortality in infants [41]. Among the numerous genetic alterations related to neuroblastoma, MYCN amplification is the most extensively studied and is the alteration most strongly correlated with the malignancy of the disease [4]. The MYCN oncogene encodes the transcription factor N-Myc, a master regulator of cell fate decisions and transcriptional programs. By modulating target gene expression, N-Myc coordinates proliferation, metabolic reprogramming, apoptosis evasion, and differentiation blockade [42]. MYCN amplification—among the earliest identified genetic markers in neuroblastoma—remains a premier prognostic indicator of aggressive disease. Present in 20–30% of patients, it stratifies a molecular cohort with < 50% long-term survival despite intensive therapy [41, 43, 44]. The results indicate that MYCN amplification is a crucial driver of high-risk neuroblastoma.

However, despite its critical oncogenic role, direct pharmacological targeting of N-Myc has remained challenging due to its helix-loop-helix structure and the lack of well-defined, druggable binding pockets [45]. Consequently, increasing attention has shifted toward identifying upstream regulators and signaling networks that modulate N-Myc stability and activity, thereby revealing exploitable vulnerabilities in MYCN-amplified neuroblastoma. In this regard, emerging evidence suggests that N-Myc operates within broader adaptive signaling networks activated under therapeutic stress [46]. In this context, an EGFR/cathepsins/MYCN axis provides a mechanistic link between growth factor signaling, lysosomal regulation, and MYCN-amplified neuroblastoma. Under chemotherapeutic stress, the tumor microenvironment, particularly tumor-associated macrophages, undergoes dynamic remodeling and secretes elevated levels of EGFR family ligands, such as HB-EGF, leading to the activation of EGFR/ERBB4 receptors on neuroblastoma cells and the initiation of pro-survival and drug-resistance signaling [47]. Concurrently, MYCN amplification not only directly promotes proliferation and chemoresistance but may also transcriptionally regulate the expression and secretion of multiple cathepsins, thereby shaping a tumor microenvironment that favors invasion and survival [48]. Notably, certain cathepsins, such as cathepsin D, act as negative regulators of the EGFR/MAPK pathway, providing an intrinsic counterbalance to excessive proliferative signaling. This regulatory interplay may explain the improved prognosis observed in neuroblastoma patients with high EGFR expression, accompanied by elevated levels of cathepsin D [48, 49]. Collectively, the convergence of EGFR signaling and MYCN-driven transcription on cathepsin regulation establishes a feed-forward circuit that sustains chemoresistance and tumor progression, underscoring the importance of upstream N-Myc regulatory pathways in shaping malignant phenotypes.

NeuroD1, a member of the bHLH transcription factor family, is crucial for neuronal differentiation during embryonic development and essential for nervous system formation [7, 9]. It is also involved in the differentiation of neural crest cells, which are the origin of neuroblastoma [10]. Previous studies have demonstrated that NeuroD1 promotes tumorigenesis in neuroblastoma [11, 13]. The study indicates that NeuroD1 expression is markedly higher in neuroblastoma patients than in neural crest cells, and it is closely linked to MYCN amplification and adverse clinical outcomes. However, the specific mechanisms through which NeuroD1 promotes neuroblastoma, especially MYCN-amplified neuroblastoma, remain unclear. We performed comprehensive in vitro and in vivo functional studies to confirm NeuroD1’s critical role in sustaining MYCN-amplified neuroblastoma. NeuroD1 silencing resulted in reduced proliferation and G1-phase cell cycle arrest in MYCN-amplified neuroblastoma. Transcriptomic analysis showed that silencing NeuroD1 led to the downregulation of various genes and signaling pathways related to DNA replication and the G1 to S phase transition, aligning with the observed changes in cellular phenotype. The downregulated genes were predominantly enriched in E2F Targets, G2M Checkpoint, and MYC Targets, which are hallmark gene sets strongly associated with MYCN [39, 40]. Furthermore, in the transcriptomic data, we observed a downregulation of several direct N-Myc target genes, including SKP2, MDM2, TP53, and ODC1. Interestingly, we found that NeuroD1 does not regulate MYCN transcription directly but instead influences the ubiquitin-proteasome degradation of N-Myc.

Research indicates that in neuroblastoma, the E3 ubiquitin ligase FBXW7 facilitates the K48-linked polyubiquitination of the N-Myc protein, marking it for proteasomal degradation [16]. In our study, we observed that silencing NeuroD1 also resulted in the accumulation of K48-linked polyubiquitination on the N-Myc protein, triggering proteasomal degradation. This clarifies that silencing NeuroD1 decreases N-Myc protein levels while leaving MYCN mRNA expression unchanged. However, since NeuroD1 is a transcription factor, it does not directly regulate post-translational modifications of proteins. Previous research has identified DUBs such as USP3 [17], USP5 [18], USP7 [19, 20], and USP36 [21], which can recognize and remove K48-linked polyubiquitin chains from N-Myc, thereby stabilizing the N-Myc protein and promoting neuroblastoma growth. However, transcriptomic analysis revealed that silencing NeuroD1 in IMR-32 cells did not significantly reduce the mRNA levels of USP3, USP5, or USP7, suggesting that NeuroD1 does not regulate N-Myc through these DUBs. To further investigate the mediator of the NeuroD1-MYCN regulatory axis, we performed transcriptomic sequencing combined with NeuroD1 ChIP-seq and identified the deubiquitinase USP1 as a direct transcriptional target of NeuroD1.

USP1 belongs to the USP deubiquitinase family, which is most commonly known for its regulation of multiple steps in the DNA damage response [23] and its role in modulating translation [24]. Additionally, USP1 has been shown to influence the differentiation of specific cell types [26]. Furthermore, USP1 is implicated in various cancers, including liver cancer [50], T-cell acute lymphoblastic leukemia [25], osteosarcoma [26], pancreatic ductal adenocarcinoma [27], and ovarian cancer [51]. However, the role of USP1 in neuroblastoma and its interaction with N-Myc protein had not been previously reported. In this study, we demonstrate that USP1 directly binds to N-Myc, removes K48-linked polyubiquitin chains, and stabilizes N-Myc protein levels. Analysis of clinical gene expression data from neuroblastoma patients revealed that elevated USP1 is significantly associated with poorer overall and event-free survival, underscoring a critical role for USP1 in neuroblastoma progression. We acknowledge, however, that this study did not include rescue experiments to determine whether USP1 overexpression is sufficient to restore N-Myc protein levels following NeuroD1 silencing, which represents a limitation and will be an important focus of future investigation to further validate the functional hierarchy of the NeuroD1-USP1-MYCN axis.

Pimozide, an FDA-approved antipsychotic drug, has been shown to inhibit USP1 [28] and demonstrate antitumor effects in various cancers, including B-cell lymphoma [32], glioblastoma [30, 31], and breast cancer [52]. In our study, we found that Pimozide treatment suppressed USP1 and N-Myc protein, leading to notable anti-proliferative effects. However, despite the promising anti-tumor effects of Pimozide in MYCN-amplified neuroblastoma, several limitations should be acknowledged. Notably, the therapeutic efficacy of pimozide was not evaluated in vivo. This does not detract from our principal mechanistic conclusions; however, future studies employing neuroblastoma xenograft or genetically engineered mouse models will be necessary to confirm antitumor efficacy, safety, and translational relevance. Pimozide exhibits known neurological adverse effects related to dopaminergic dysfunction, particularly under prolonged or high-dose exposure, raising safety concerns in pediatric populations. These neurotoxic liabilities may restrict its direct translational applicability despite its efficacy in destabilizing N-Myc. Moreover, Pimozide is a multi-target compound, and its inhibitory effect on USP1 may not be specific. Therefore, the development of more selective USP1 inhibitors and careful assessment of efficacy‑safety balance will be essential to advance this strategy toward clinical translation.

In summary, our study identifies NeuroD1 as a transcriptional activator of USP1 and demonstrates USP1-mediated N-Myc stabilization, revealing a targetable signaling axis (NeuroD1-USP1-MYCN) unique to MYCN-driven neuroblastoma pathogenesis (Fig. 8G). Moreover, unlike transcription factors, which are challenging to target with drugs, USP1 is a promising therapeutic target. Notably, the FDA-approved drug Pimozide has been shown to inhibit USP1, making it a potential therapeutic agent for MYCN-amplified neuroblastoma, with significant clinical application potential.

## Supplementary Information

Below is the link to the electronic supplementary material.

Supplementary Material 1

Supplementary Material 2

Supplementary Material 3

Supplementary Material 4

Supplementary Material 5

## Data Availability

Our sequencing and processed data files had been submitted to the Gene Expression Omnibus (GEO; http://www.ncbi.nlm.nih.gov/geo/) repository, GSE285629 (IMR-32 NeuroD1 ChIPseq), GSE285628 (IMR-32 H3K27Ac CUT&Tag), GSE285630 (IMR-32 ATACseq), GSE285627 (IMR-32 RNAseq).
