# Supplementary material for: NeuroD1-USP1-MYCN axis drives tumor progression in neuroblastoma
Source: J Transl Med. 2026 Feb 11;24:381. doi: 10.1186/s12967-026-07844-5 (PMC12997678; doi:10.1186/s12967-026-07844-5)
Supplement: Supplementary file 4 — Supplementary Material 4 [file 12967_2026_7844_MOESM4_ESM.pdf]

Supplementary materials

1. Supplementary methods

Cell proliferation and EdU incorporation assay

Cell proliferation was assessed using the CCK-8 assay. Briefly, CCK-8 reagent (DOJINDO) was added to each well, followed by incubation for 2 hours. After incubation, absorbance was measured at 450 nm using a Microplate Absorbance Reader (Bio-Rad).

EdU incorporation assay was performed using the BeyoClick™ EdU Cell Proliferation Kit (Beyotime) according to the manufacturer's instructions. Briefly, cells were incubated with EdU for 2 h. Afterward, they were fixed with 4% paraformaldehyde for 10 minutes, blocked with Immunol Staining Blocking Buffer (Beyotime) for 1 hour, and then treated with the Click Reaction Mixture for 30 minutes. Finally, cells were incubated with DAPI for 5 minutes.

Cell cycle analysis

Cell cycle analysis was performed using the Cell Cycle Assay Kit (Fcmacs) following the manufacturer's instructions. In brief, cells were harvested and fixed in 70% ethanol. After fixation, the cells were stained with propidium iodide (PI) solution and analyzed on a Gallios flow cytometer (Beckman Coulter). The data were subsequently processed using ModFit LT software (Verity Software House).

RNAseq data analysis

Expression analysis: Transcript sequences in FASTA format were first generated from a known GFF annotation file and properly indexed. Using this indexed file as the reference gene file, HTSeq (v0.6.1) was utilized to estimate gene and isoform expression levels from the paired-end clean sequencing data.

Differential expression analysis: Differential expression analysis was performed using the DESeq2 Bioconductor package, which employs a model based on the negative binomial distribution. Estimates of dispersion and logarithmic fold changes were calculated with data-driven prior distributions. Genes with an adjusted p-value (Padj) < 0.05 were considered differentially expressed.

GO and KEGG enrichment analysis: GOSeq (v1.34.1) was utilized to identify Gene Ontology (GO) terms associated with enriched genes, with significance determined by an adjusted p-value (Padj) < 0.05. The topGO package was employed to generate directed acyclic graphs (DAGs) for visualization. Additionally, KEGG (Kyoto Encyclopedia of Genes and Genomes)—a comprehensive database of genomes, biological pathways, diseases, drugs, and chemical substances—was used for pathway enrichment analysis. In-house scripts were applied to identify significant differentially expressed genes enriched in KEGG pathways.

ChIPseq

ChIP was performed on 5×10^7 IMR-32 cells following an established protocol. The cells were initially cross-linked with 1% paraformaldehyde, and the reaction was quenched with glycine for 5 minutes. After centrifugation, the cell pellets were lysed in

cell lysis buffer containing protease inhibitors. The lysed cells were gently disrupted using a 1 mL insulin syringe for efficient lysis. The resulting precipitate was resuspended in shearing buffer supplemented with protease inhibitors, and the chromatin was sonicated to generate DNA fragments ranging from 300 to 800 bp. The supernatant was then incubated overnight at 4 °C with an H3K27Ac antibody (Abcam). On the following day, Dynabeads Protein G (Thermo Fisher Scientific) was added to the mixture and incubated at 4 °C for 4 hours to facilitate immunoprecipitation. The antibody-chromatin complexes bound to the beads were washed sequentially with lysis buffer and TE buffer. The complexes were then eluted using an elution buffer and incubated with 5 M NaCl at 65 °C overnight to reverse cross-linking. To eliminate RNA contamination, RNase (CST) was added, and the mixture was incubated at 37 °C for 30 minutes. Proteinase K (Invitrogen), 1 M Tris-HCl (pH 8.0), and 0.5 M EDTA (pH 8.0) were subsequently added, followed by incubation at 45 °C for 1 hour. Finally, DNA fragments were purified using the PCR Purification Kit (QIAGEN) and prepared for downstream analysis.

Cleavage Under Target & Tagmentation (CUT&Tag)

CUT&Tag assay was performed using the Hyperactive Universal CUT&Tag Assay Kit for Illumina (Vazyme) according to the manufacturer’s instructions. DNA was extracted and amplified with i5 and i7 primers from the TruePrep Index Kit V2 for Illumina (Vazyme). The resulting libraries were purified using VAHTS DNA Clean Beads (Vazyme) and subsequently sequenced by Novogene Bioinformatics Technology Co.,

Ltd. The raw CUT&Tag data have been deposited in the Gene Expression Omnibus (GEO) database.

Assay for Transposase Accessible Chromatin with high-throughput sequencing (ATACseq)

ATACseq assay was performed using the Hyperactive ATAC-Seq Library Prep Kit for Illumina (Vazyme) according to the manufacturer's instructions. DNA was extracted and amplified with i5 and i7 primers from the TruePrep Index Kit V2 for Illumina (Vazyme). The resulting libraries were purified using VAHTS DNA Clean Beads (Vazyme) and subsequently sequenced by Novogene. The raw ATACseq data have been deposited in the Gene Expression Omnibus (GEO) database.

Dual-luciferase reporter assay

293FT cells were seeded in 12-well plates and transfected with the target plasmids, firefly luciferase reporter plasmid (pGL3-promoter-luc), and Renilla luciferase expression plasmid (pRL-CMV) using jetPRIME transfection reagent (Polyplus) at a 5:4:1 ratio. After 6 h, the medium was replaced with fresh medium containing 1 μg/mL doxycycline. 48 h post-transfection, luciferase activity was measured using the Dual-Luciferase Reporter Assay System (Promega). The firefly luciferase activity was normalized to Renilla luciferase activity to calculate the relative activity of promoter-transfected cells.

Protein structure prediction

The amino acid sequences of N-Myc and USP1 were retrieved from the National Center for Biotechnology Information (NCBI). High-resolution structures of N-Myc and USP1 were predicted using the monomer_casp14 model in Alphafold v2.3.2. The following database versions were utilized: UniProt 2023-03-01, UniRef90 2023-03-01, PDB_mmCIF 2023-03-03, and PDB_seqres 2023-03-03. Additionally, the Predicted Targeting Metric (pTM) was employed to assess the quality of the protein structures predicted by Alphafold.

Protein-Protein Docking

This study used Schrödinger software for protein-protein docking. First, the proteins were preprocessed, including bond-level assignment, hydrogenation, zero-order bond assignment to metal atoms, and disulfide bond creation for both proteins. Next, the hydrogen bond network was optimized, and protein energy minimization was performed using the OPLS_4 force field. The Protein-Protein docking module (Piper) in Schrödinger was then used for molecular docking. Standard mode was applied for docking, with the number of rotatable probes for the ligand set to 70,000 to ensure sufficient conformational sampling of the ligand protein. The number of generated conformations was set to 30. For proteins with multiple chains, only one chain was used for docking. Piper clusters the top 1,000 rotational conformations based on the Root Mean Square Deviation (RMSD) between each atom and selects the representative conformation in each cluster based on the most neighbors. Piper then ranks the

generated conformations based on the number of clusters in each category. The top-ranked conformation, which has the largest number of clusters, represents the optimal binding mode predicted for the USP1 and N-Myc protein interaction.

Molecular Dynamics (MD) Simulation

On an Ubuntu 20.04.01 platform with an Intel Core i9-12900k CPU, GeForce RTX 4070 GPU, and 64 GB of RAM, GROMACS (2023.1 single precision) was employed for conducting the MD simulation. The software SPDBV 4.10 was initially used to modify the heavy ions and small molecules of the proteins. Using the CHARMM36 force field, the protein's topology structure was calculated. The TIP3P water model was applied to solve the complex, which was encased in a cube-shaped box with at least 1.2 nm spacing on all sides. The system was neutralized by adding Na+ and Cl- ions, followed by the addition of 0.15 M NaCl to restore it to a near-physiological state. Subsequently, the system underwent energy minimization using the steepest descent method for 5000 iterations, with a maximum force of less than 1000 kJ/mol/nm. To achieve a well-equilibrated and stable system, 100 ps of restricted NVT (number of particles, volume, temperature) and NPT (number of particles, pressure, temperature) equilibration was performed at 310 K and 1 bar. The final MD simulation of the complex was performed for 100 ns over 50 million steps. During the simulation, the Verlet cut-off scheme and Leap-frog integrator with a 2 fs step size were used, and trajectory data was saved every 10 ps. Unless otherwise stated, RMSD calculations refer only to the protein backbone. The "gmx rms" command was utilized to compute

RMSD, employing the least-squares method to fit the structure to the reference structure (t2 = 0) and then quantifying RMSD based on the following formula:

$$RMSD\ (t_1,\ t_2) = \left[\frac{1}{M}\sum_{i=1}^{N} m_i \| r_i(t_1) - r_i(t_2) \|^2\right]^{1/2} \quad (1)$$

Where, $M = \sum_{i=1}^{N} m_i$, r_i (t) is the the position of the atom *i* at frame *t*. Unless explicitly stated otherwise, the Root Mean Square Fluctuation (RMSF) calculations pertain solely to the protein backbone. The RMSF value is determined using the following formula:

$$\text{RMSFi} = \sqrt{\frac{1}{T}\sum_{t=1}^{T}(r_i\ (t) - \bar{r})^2} \quad (2)$$

Where, ri (t) is the position vector of the atom i at frame t and is the average atom position over all T frames. Radius of Gyration (RoG) plays a pivotal role in the evaluation of protein structure and its conformational alterations. In this research, the RoG value is determined using the following formula:

$$\text{RoG} = \left(\frac{\sum_i \| r_i \|^2 m_i}{\sum_i m_i}\right)^{1/2} \quad (3)$$

Where, mi is the molecular weight of atom i, ri is the position of atom i relative to the center of the molecule. The Solvent Accessible Surface Area (SASA) was estimated using GROMACS commands. In this research, the SASA value is determined using the following formula:

$$\text{SASA} = \sum \frac{R}{\sqrt{R^2 - Z_i^2}}\ x\ D\ x\ L_i \quad (4)$$

Where, Li is the length of the arc drawn on a given section i; Zi is the perpendicular distance of section i from the centre of the sphere. Free energy landscape (FEL) of receptor-ligand binding represents a vivid and informative two- or three-dimensional depiction of the changes in free energy that occur during the binding process between

receptor and ligand. The translational and rotational motion of the MD simulation trajectory was corrected using the gmx trjconv command. Upon obtaining the computed results of the RMSD and RoG, the FEL of protein-ligand complex stability was calculated using the gmx sham command. After analyzing the energy well, the gmx trjconv command was used to extract the conformation of the energy minimum point.

Immunohistochemistry of Neuroblastoma Patient Tissues

Immunohistochemical staining on formalin-fixed, paraffin-embedded neuroblastoma tumor tissue sections was performed by Shanghai Noblebio Co., Ltd., following a standard protocol. Briefly, sections were deparaffinized, rehydrated, and subjected to heat-mediated antigen retrieval. Endogenous peroxidase activity was blocked with 3% hydrogen peroxide, and non-specific binding sites were blocked with serum. The sections were then incubated overnight at 4°C with primary antibodies specific for N-Myc and USP1. Subsequently, the corresponding horseradish peroxidase-conjugated secondary antibodies were applied, and the signal was visualized using 3,3'-diaminobenzidine (DAB) substrate, followed by counterstaining with hematoxylin.

A total of 10 neuroblastoma patient samples, comprising 2 MYCN-amplified and 8 non-amplified cases, were analyzed in this study. Their clinicopathological characteristics are summarized in Supplementary Table S10.

2. Supplementary figures (Figures S1–S7)

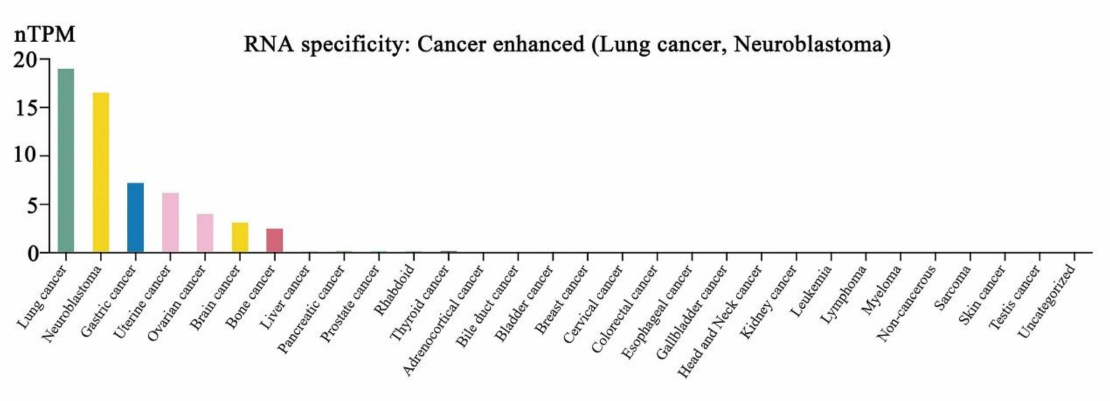

Figure S1. NeuroD1 is Highly Expressed in Neuroblastoma.

The cell line data is sourced from the Human Protein Atlas (https://www.proteinatlas.org), which provides genome-wide RNA expression profiles of human protein-coding genes across 1,132 human cancer cell lines.

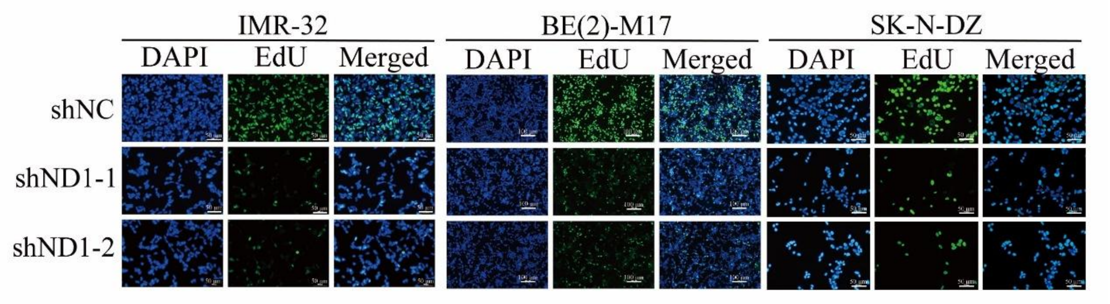

Figure S2. Knockdown of NeuroD1 Inhibits DNA Replication in Neuroblastoma Cells.

DNA replication was assessed using the BeyoClick™ EdU Cell Proliferation Kit with Alexa Fluor 488. The white scale bars represent 50 μm in IMR-32 and SK-N-DZ cells, and 100 μm in BE(2)-M17 cells.

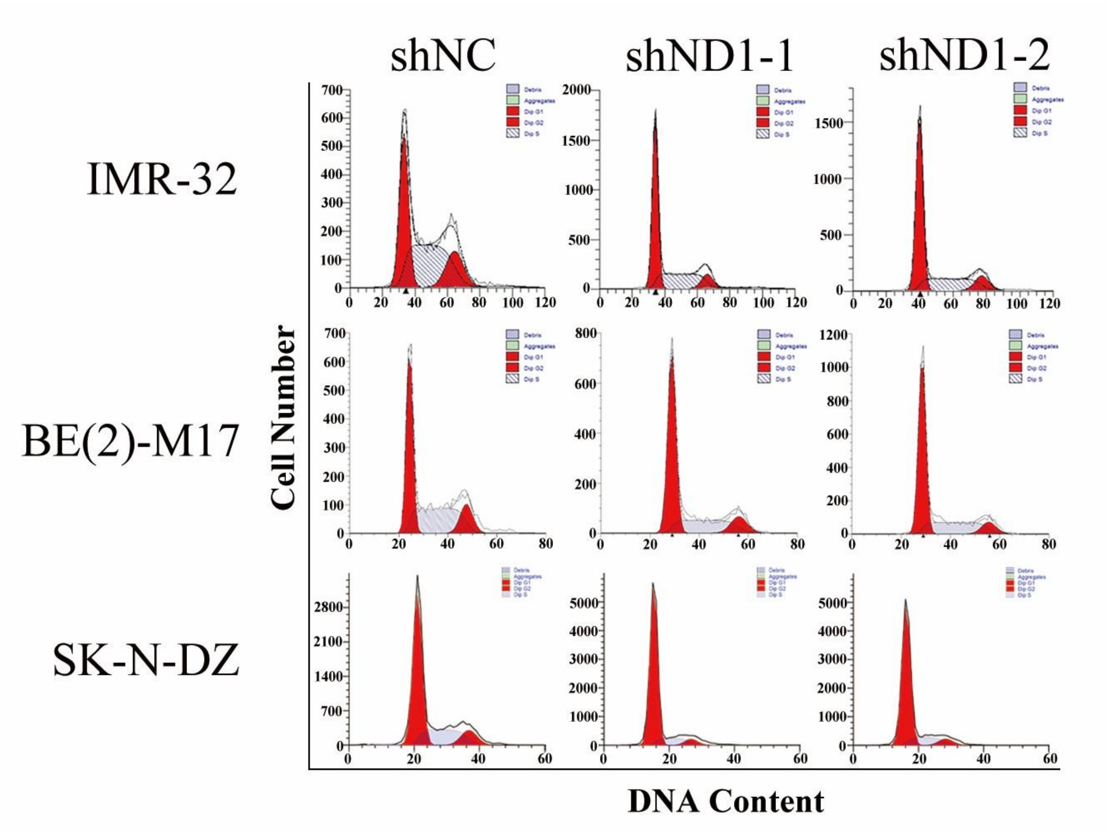

Figure S3. Knockdown of NeuroD1 Induces G1 Phase Arrest in Neuroblastoma Cells.

Cell cycle distribution was analyzed using the Cell Cycle Assay Kit followed by flow cytometry to determine the percentage of cells in each phase.

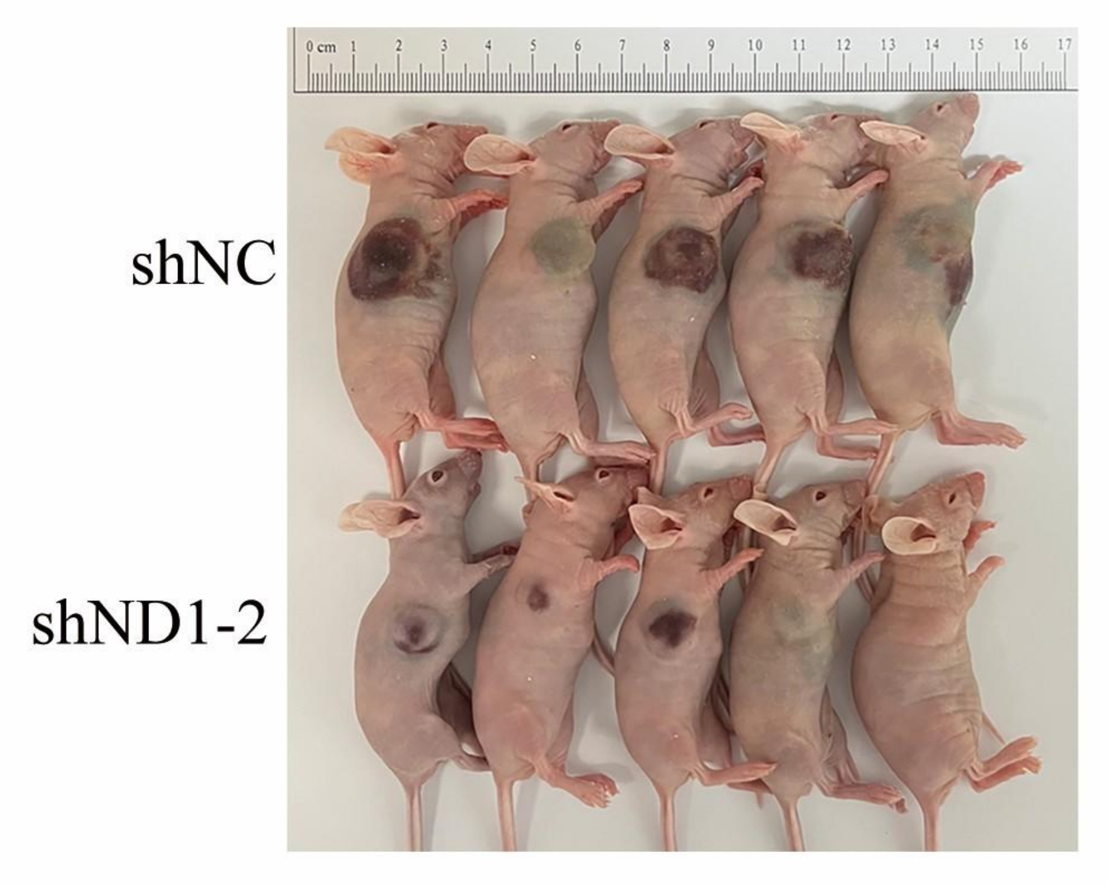

Figure S4. Knockdown of NeuroD1 Suppresses *In Vivo* Tumorigenesis of Neuroblastoma Cells.

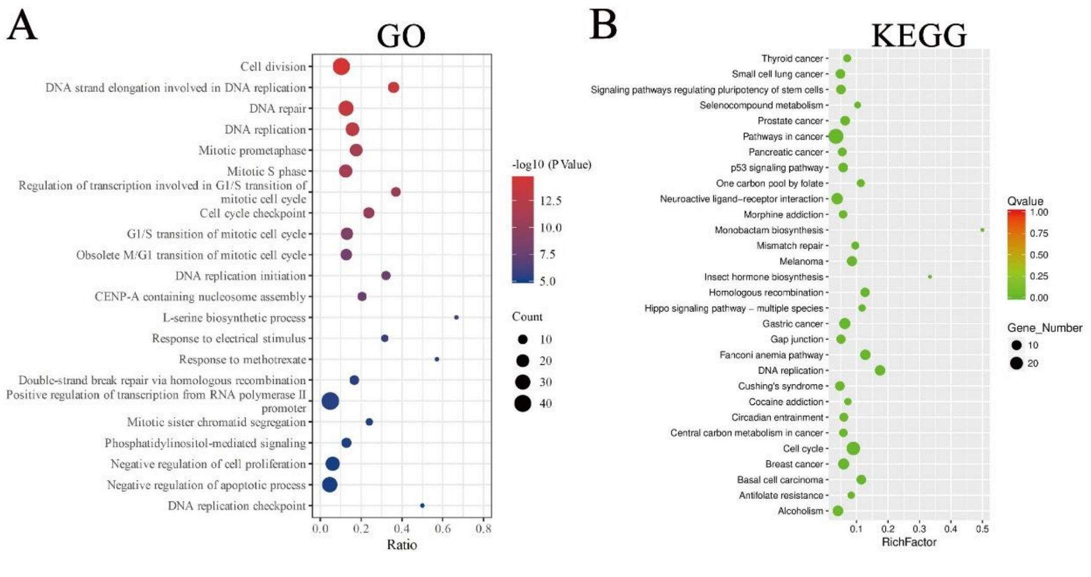

Figure S5. GO and KEGG Analysis of Downregulated Genes Following NeuroD1

Knockdown.

Gene Ontology (GO) and Kyoto Encyclopedia of Genes and Genomes (KEGG) pathway enrichment analyses were performed on genes downregulated after NeuroD1 knockdown in IMR-32.

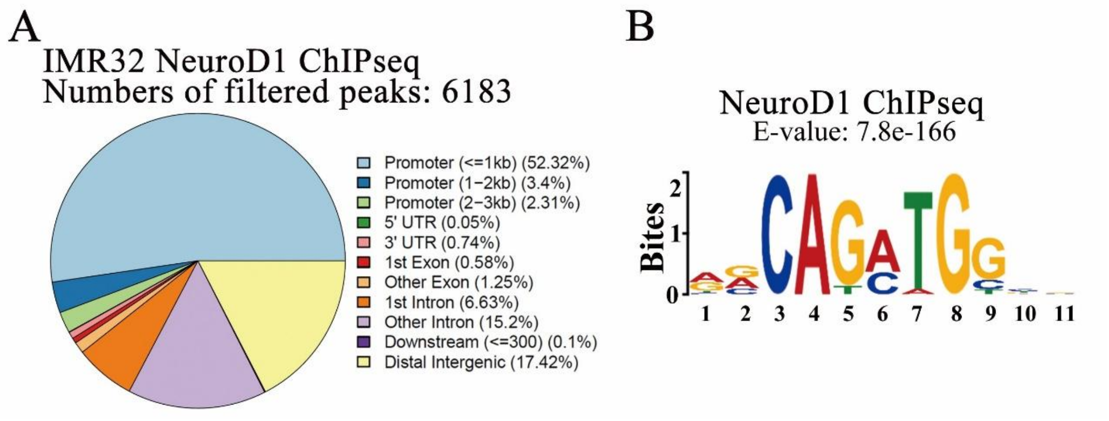

Figure S6. NeuroD1 ChIP-seq Analysis.

A Distribution of NeuroD1 binding sites across the genome in IMR-32 cells. **B** Motif analysis of NeuroD1 binding sites using HOMER revealed significant enrichment for NeuroD1-specific motifs.

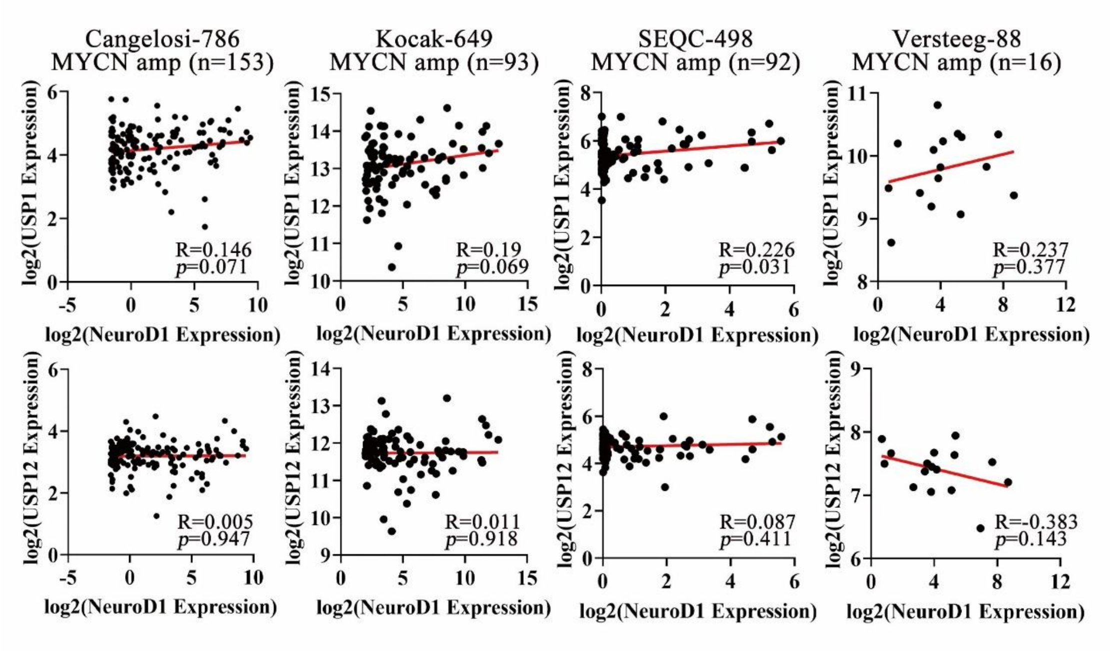

Figure S7. Correlation of NeuroD1 with USP1 and USP12 in MYCN-Amplified Neuroblastoma Patients.

Correlation analysis of NeuroD1 with USP1 and USP12 mRNA levels in MYCN-amplified neuroblastoma patients was performed using the Cangelosi-786 (n=153), Kocak-649 (n=93), SEQC-498 (n=92), and Versteeg-88 (n=16) datasets.

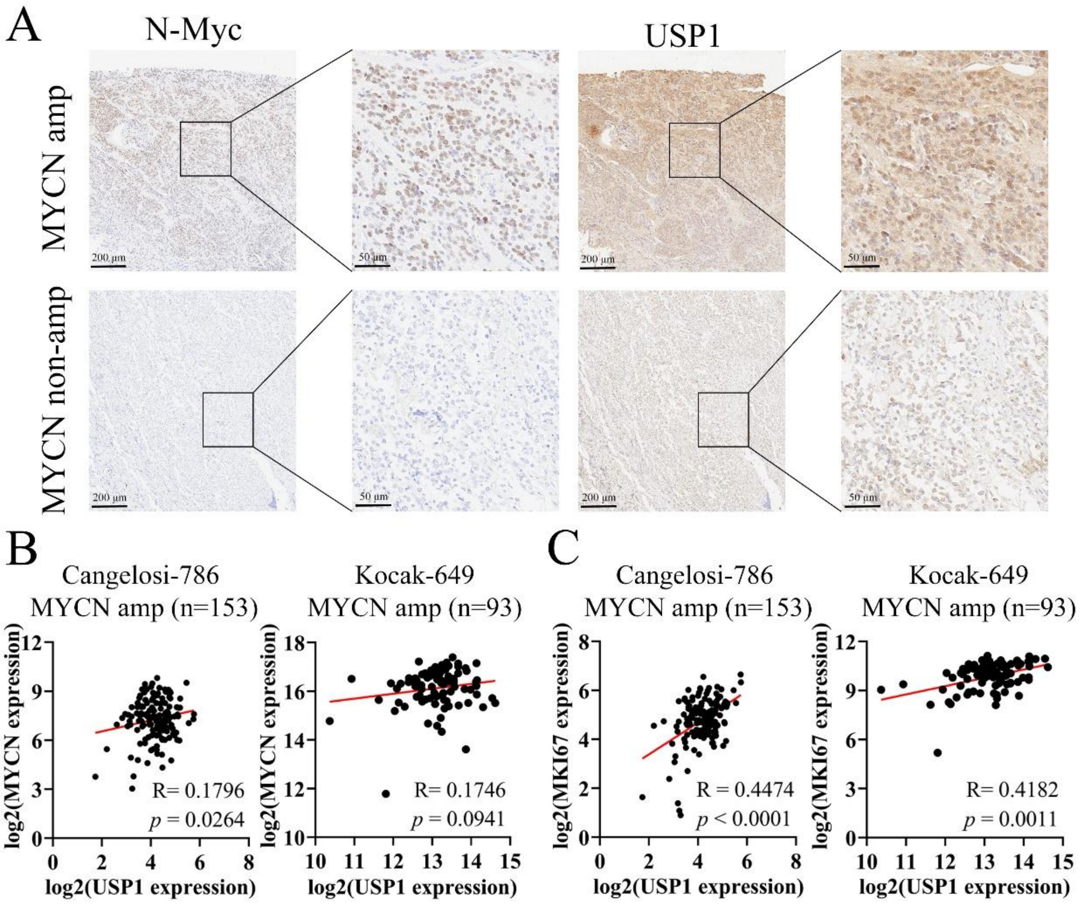

Figure S8. Correlation of USP1 with MYCN and MKI67 in Neuroblastoma Patients.

A Detection of N-Myc and USP1 by immunohistochemistry in human neuroblastoma tissues. Formalin-fixed, paraffin-embedded tissue sections were stained with anti-N-Myc and anti-USP1 antibodies. MYCN amp: MYCN amplified neuroblastoma patient tissue. MYCN non-amp: MYCN non-amplified neuroblastoma patient tissue. **B, C** Correlation analysis of USP1 with MYCN (B), and MKI67 (C) mRNA levels in MYCN-amplified neuroblastoma patients was performed using the Cangelosi-786 (n=153), and Kocak-649 (n=93) datasets.

3. Supplementary Tables

Supplementary Table S1. Cell lines used in this study.

Cell line	Source
IMR-32	The Children's Hospital of Soochow University
BE(2)-M17	The Children's Hospital of Soochow University
SK-N-DZ	The Children's Hospital of Soochow University
HEK-293FT	The Children's Hospital of Soochow University

Supplementary Table S2. Cell culturing of different neuroblastoma cell lines.

Cell line	Cell culturing medium	Serum	Penicillin/streptomycin
IMR-32	MEM	10%	1%
BE(2)-M17	MEM/F12	10%	1%
SK-N-DZ	DMEM	10%	1%
HEK-293FT	DMEM	10%	1%

Supplementary Table S3. Antibodies used in this study.

antibody	Company	Catalog Number
HA-Tag	Cell Signaling Technology	3724S
DYKDDDDK-Tag	Cell Signaling Technology	14793S
GAPDH	Cell Signaling Technology	2118S
CDK4	Cell Signaling Technology	12790S
N-Myc	Cell Signaling Technology	51705S
NeuroD1	Cell Signaling Technology	7019S
NeuroD1	Proteintech	12081-1-AP
USP1	Proteintech	14346-1-AP
GFP	Proteintech	66002-1-Ig
HRP- Goat Anti-Rabbit	Proteintech	SA00001-2
HRP- Goat Anti-Mouse	Proteintech	SA00001-1
CyclinD1	Abcam	ab134175
PCNA	Abcam	ab92552
Ki-67	Abcam	ab16667
H3K27Ac	Abcam	ab4729
Ubiquitin (linkage-specific K48)	Abcam	ab140601

Supplementary Table S4. ShRNA sequences.

shRNA	sequences (5′–3′)
shNC	CCGGCAACAAGATGAAGAGCACCAACTCGAGTTGGTGCTCTTCATCTTGTTGTTTTTG
shNeuroD1-1	CCGGGCCTTGCTATTCTAAGACGCACTCGAGTGCGTCTTAGAATAGCAAGGCTTTTT
shNeuroD1-2	CCGGGCACAATTTGAGCAATTCATTCTCGAGAATGAATTGCTCAAATTGTGCTTTTT

Supplementary Table S5. Primer sequences for RT-qPCR.

Gene	Forward primer (5′–3′)	Reverse primer (5′–3′)
NeuroD1	GGTGCCTTGCTATTCTAAGACGC	GCAAAGCGTCTGAACGAAGGAG
USP1	GCTCTAAAGGATGAAGCCAATCAA	ACTAGCCTGGAGCTGTTCAACC
MYCN	ACCACAAGGCCCTCAGTACCTC	TGACAGCCTTGGTGTTGGAGGA
GAPDH	ATCATCCCTGCCTCTACTGG	CCCTCCGACGCCTGCTTCAC

Supplementary Table S6. Primer sequences for ChIP-PCR.

Location	Forward primer (5′–3′)	Reverse primer (5′–3′)
P1	CTCACCAAAGCAGGCACA	CAGGAGGAAACGAAAGAACA
P2	GCCGAGAAAGGGAAGAGG	GCTGCCACGGATAACAATACA
P3	GCCTGAGGAACACTCCGTAT	CAATCCATGATGAACAATCCC
P4	CTGTGGAACCCGTTAGGCT	GAACGTGGCGTCCTTTGA

Supplementary Table S7. Differentially expressed genes detected by RNASeq in IMR-32 cells following NeuroD1 knockdown.

Supplementary Table S8. GSEA analysis results following NeuroD1 knockdown in IMR-32 cells.

Supplementary Table S9. Peaks detected by ChIPseq analysis of NeuroD1 in IMR-32 cells.

Supplementary Table S10. Summary of neuroblastoma patient characteristics.

Patient ID	MYCN Status	Age (months)	Sex	INSS Stage	Risk Group*	Primary Site	Status (Follow-up months)
**NB-01**	**Amplified**	15	M	4	High	Adrenal	DOD (18)
**NB-02**	**Amplified**	8	F	3	High	Retroperitoneal	DOD (24)
NB-03	Non-amplified	36	F	1	Low	Mediastinum	NED (60)
NB-04	Non-amplified	4	M	4S	Low	Adrenal	NED (48)
NB-05	Non-amplified	22	M	2B	Intermediate	Adrenal	NED (54)
NB-06	Non-amplified	18	F	3	Intermediate	Retroperitoneal	AWD (36)
NB-07	Non-amplified	60	M	4	High	Mediastinum	DOD (30)
NB-08	Non-amplified	10	F	1	Low	Adrenal	NED (66)
NB-09	Non-amplified	28	M	3	Intermediate	Retroperitoneal	NED (42)
NB-10	Non-amplified	3	F	4S	Low	Adrenal	NED (72)

Table notes:

*Risk Group was determined according to the Children's Oncology Group (COG) classification system, integrating age, INSS stage, MYCN status, histology, and ploidy.

Abbreviations: INSS, International Neuroblastoma Staging System; DOD, dead of disease; AWD, alive with disease; NED, no evidence of disease.
