# Supplementary material for: NeuroD1-USP1-MYCN axis drives tumor progression in neuroblastoma
Source: J Transl Med. 2026 Feb 11;24:381. doi: 10.1186/s12967-026-07844-5 (PMC12997678; doi:10.1186/s12967-026-07844-5)

Figure2

A

IMR-32-NeuroD1

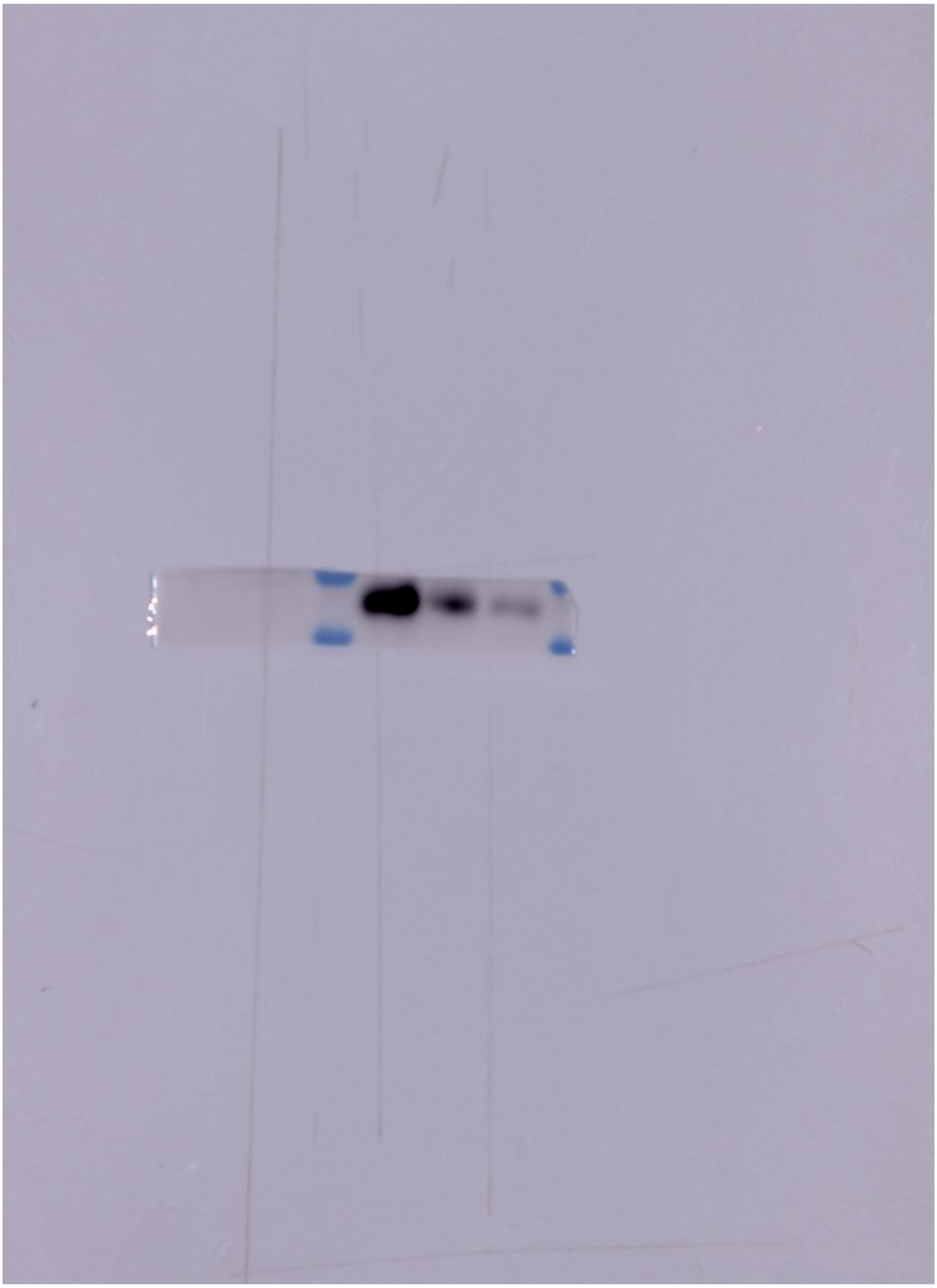

IMR-32-GAPDH

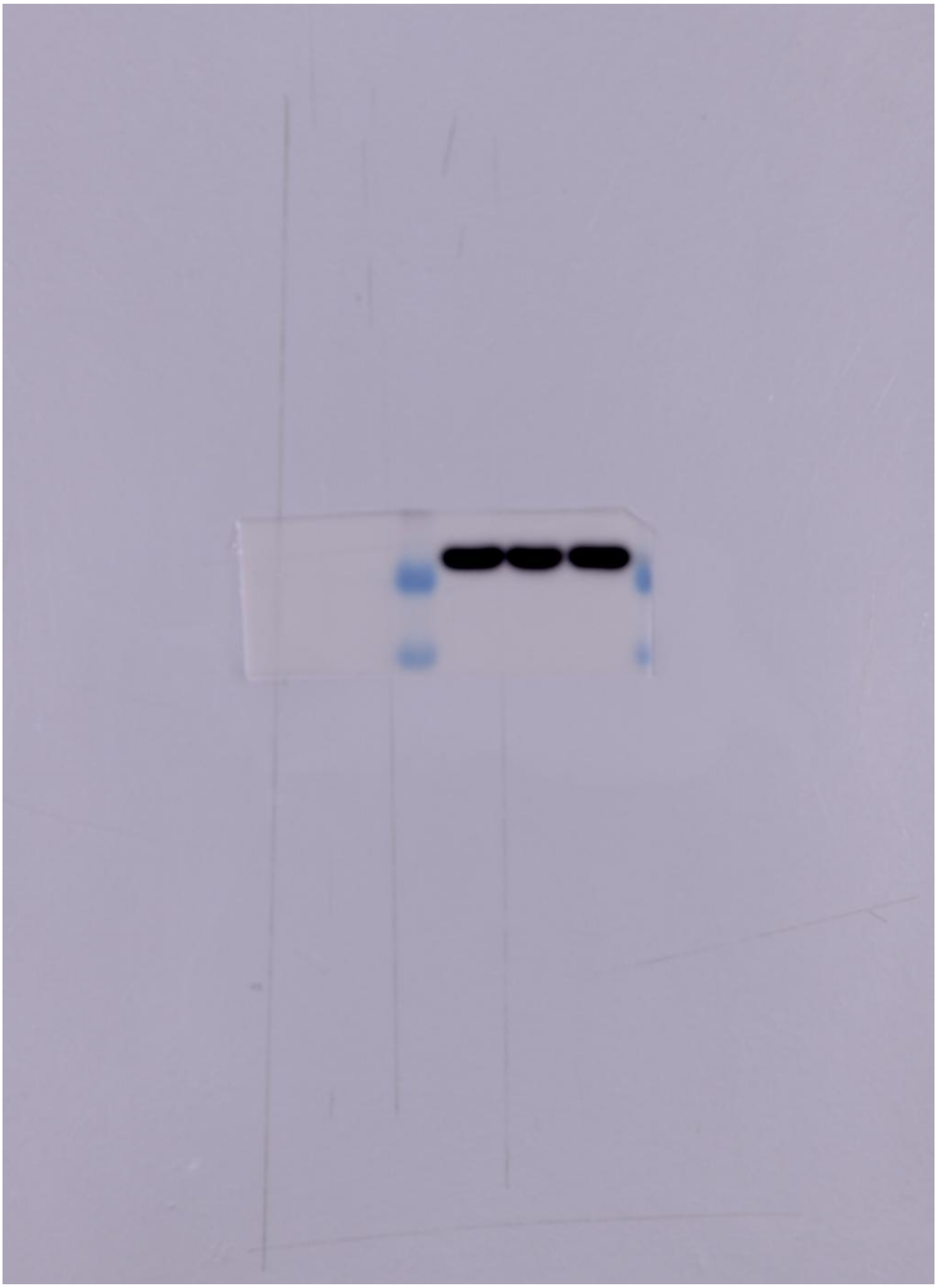

BE(2)-M17-NeuroD1

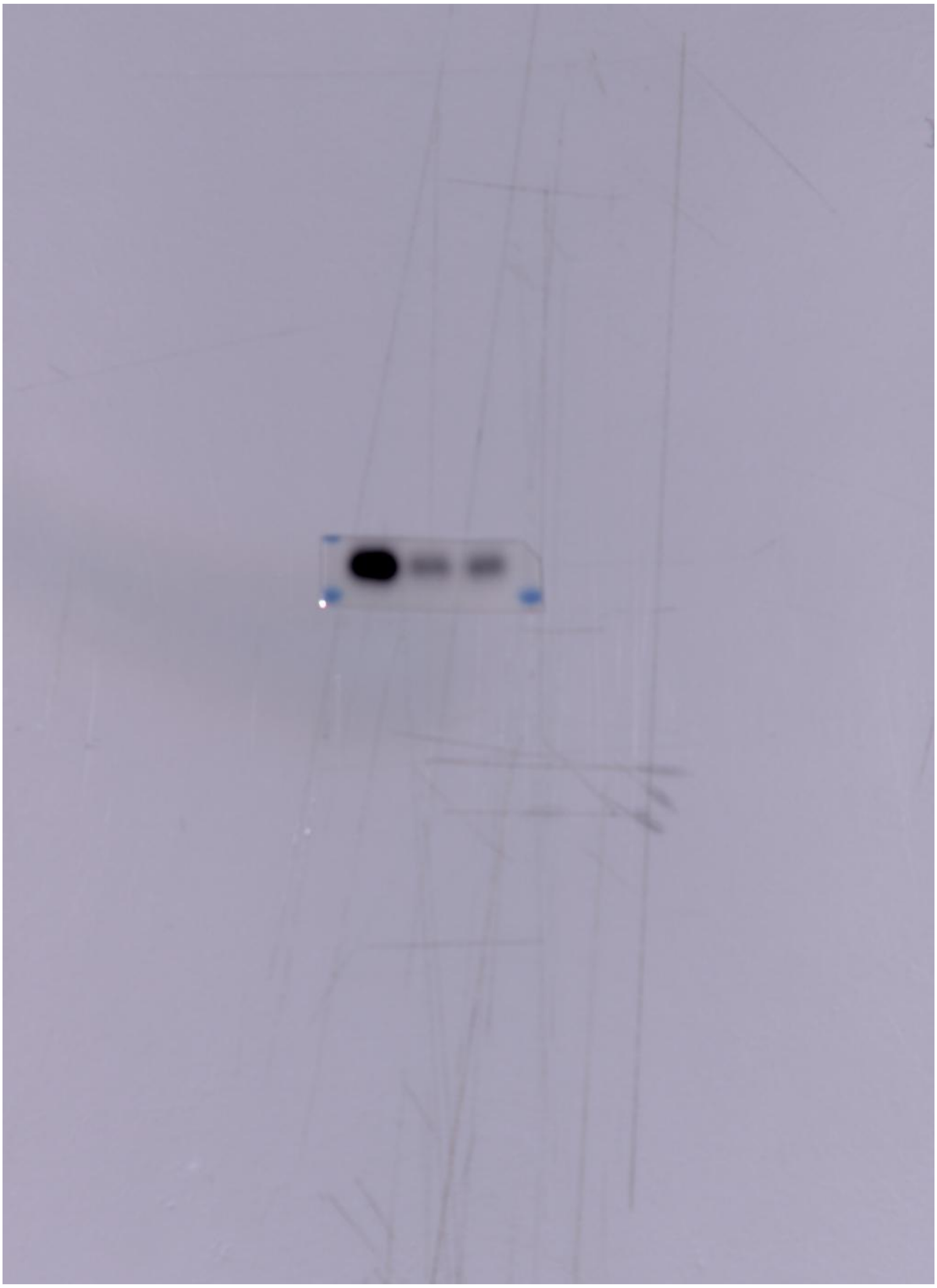

BE(2)-M17-GAPDH

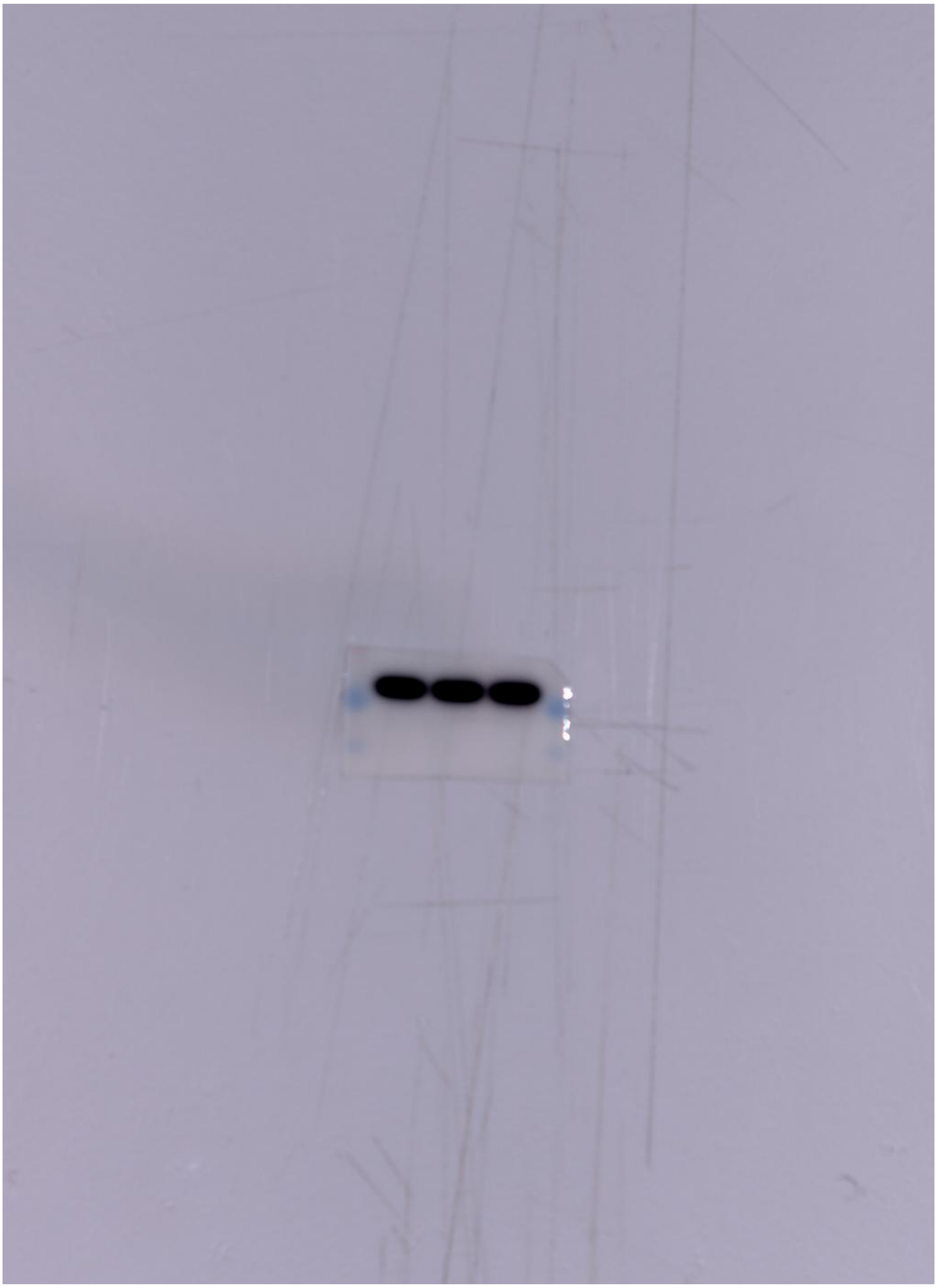

SK-N-DZ-NeuroD1

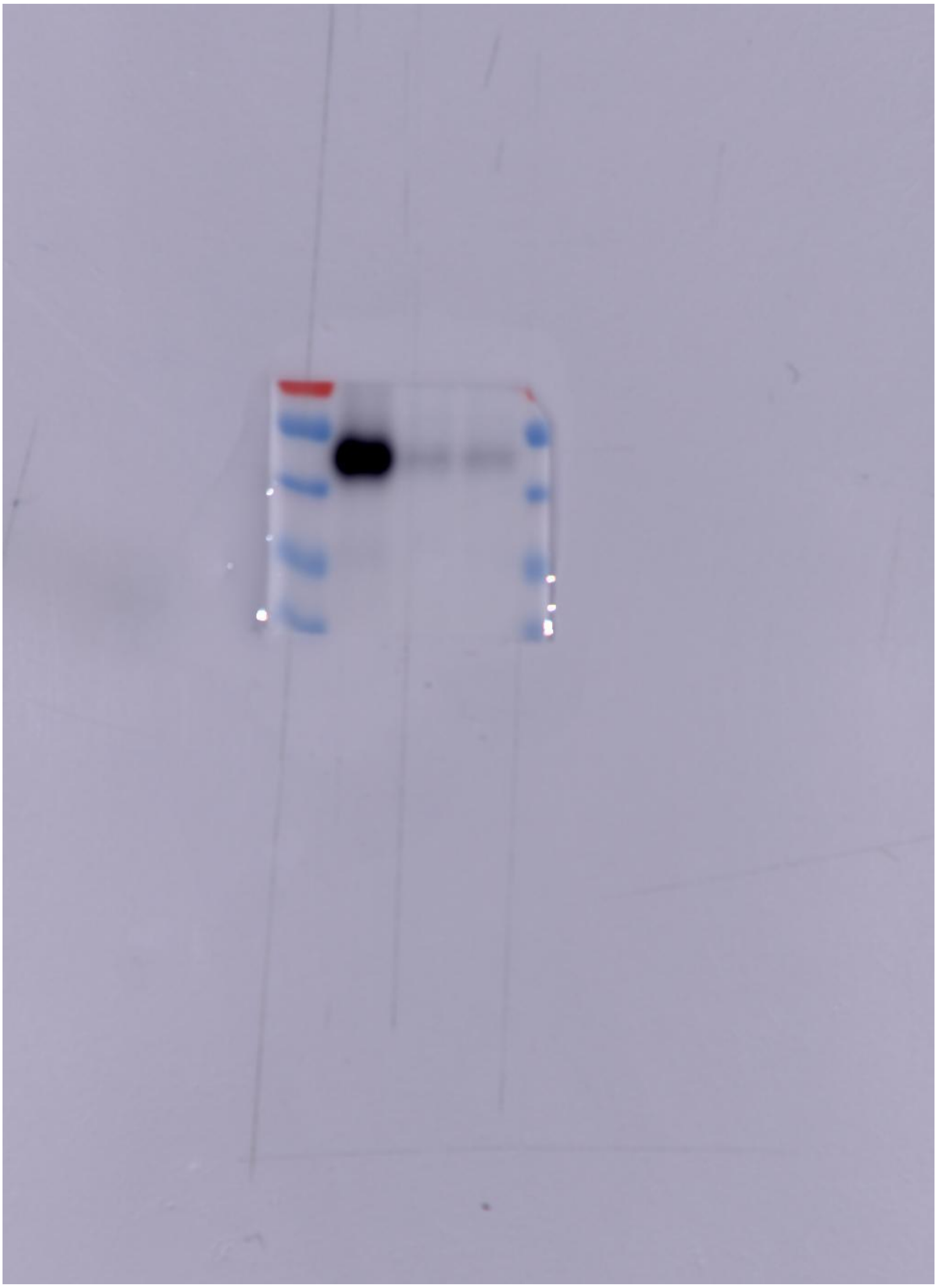

SK-N-DZ-GAPDH

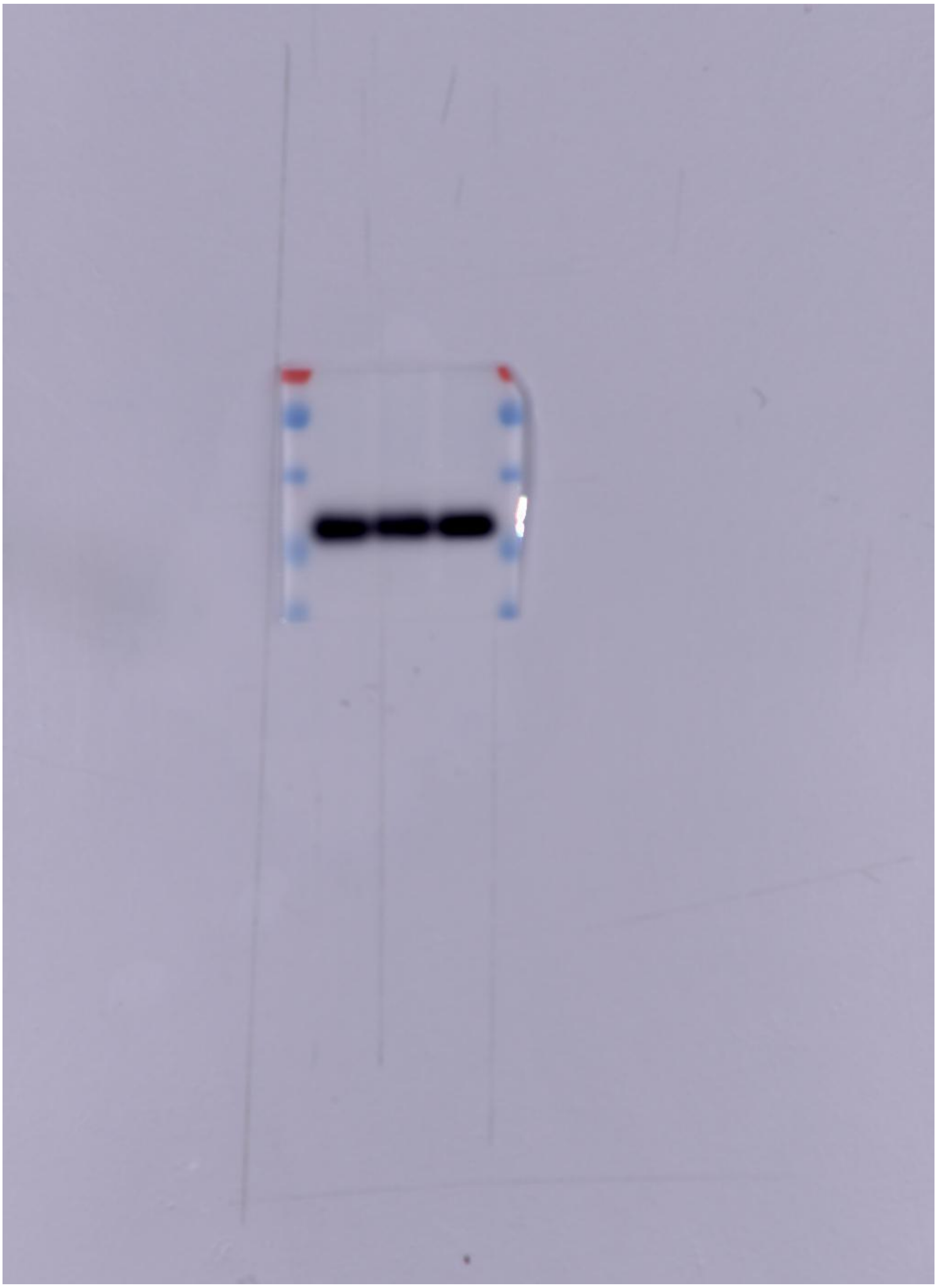

E

IMR-32-NeuroD1

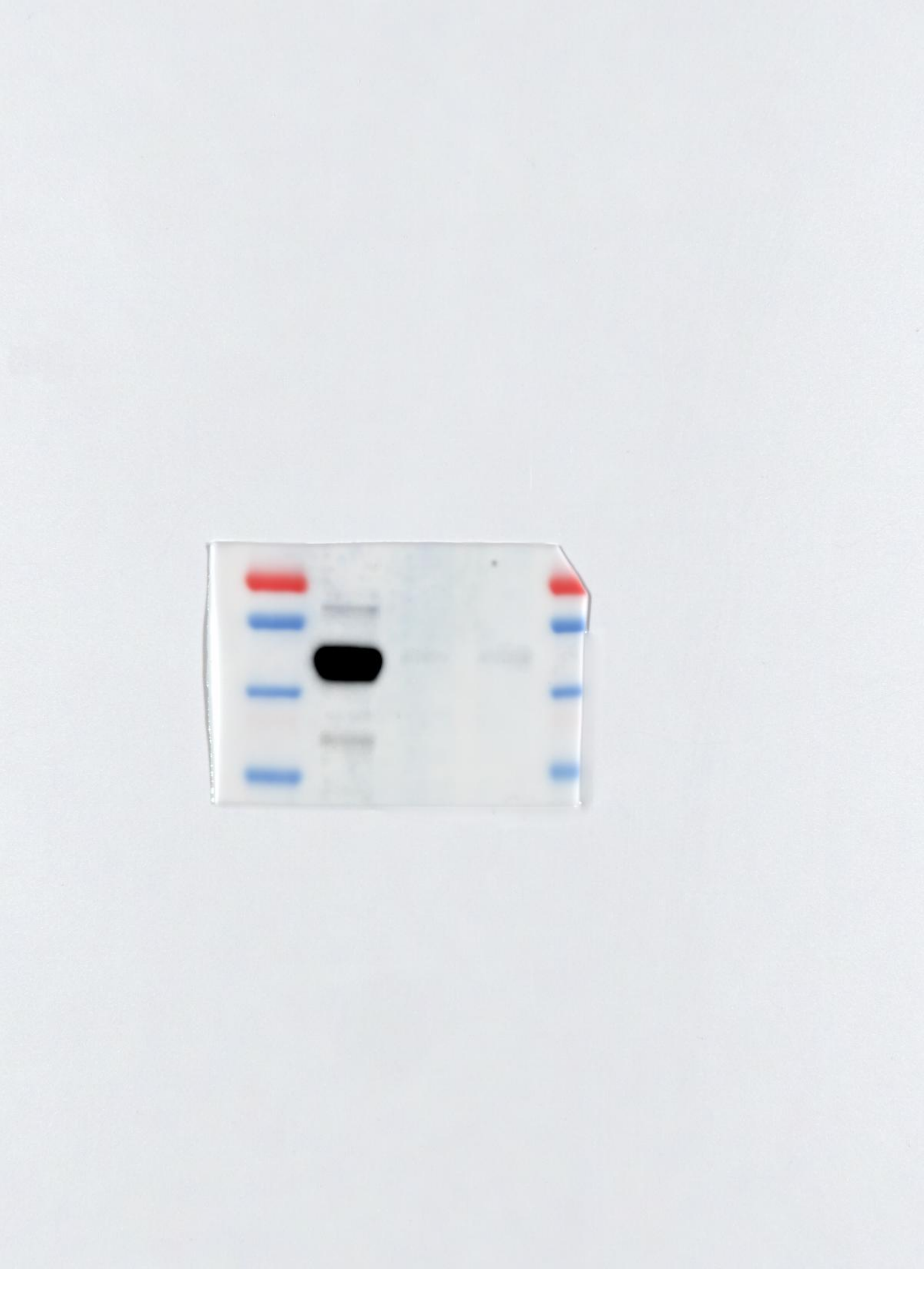

IMR-32-CyclinD1

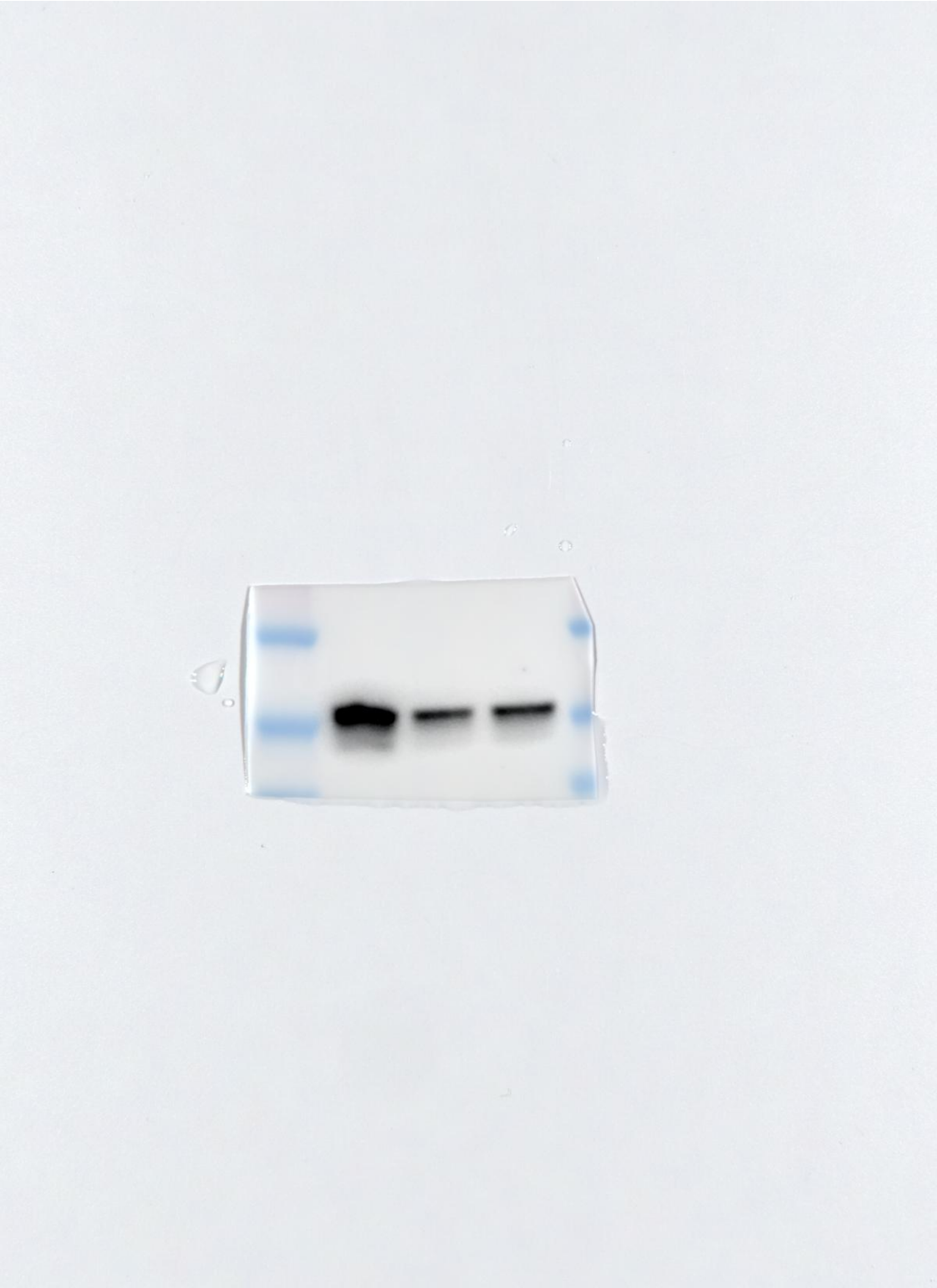

IMR-32-CDK4

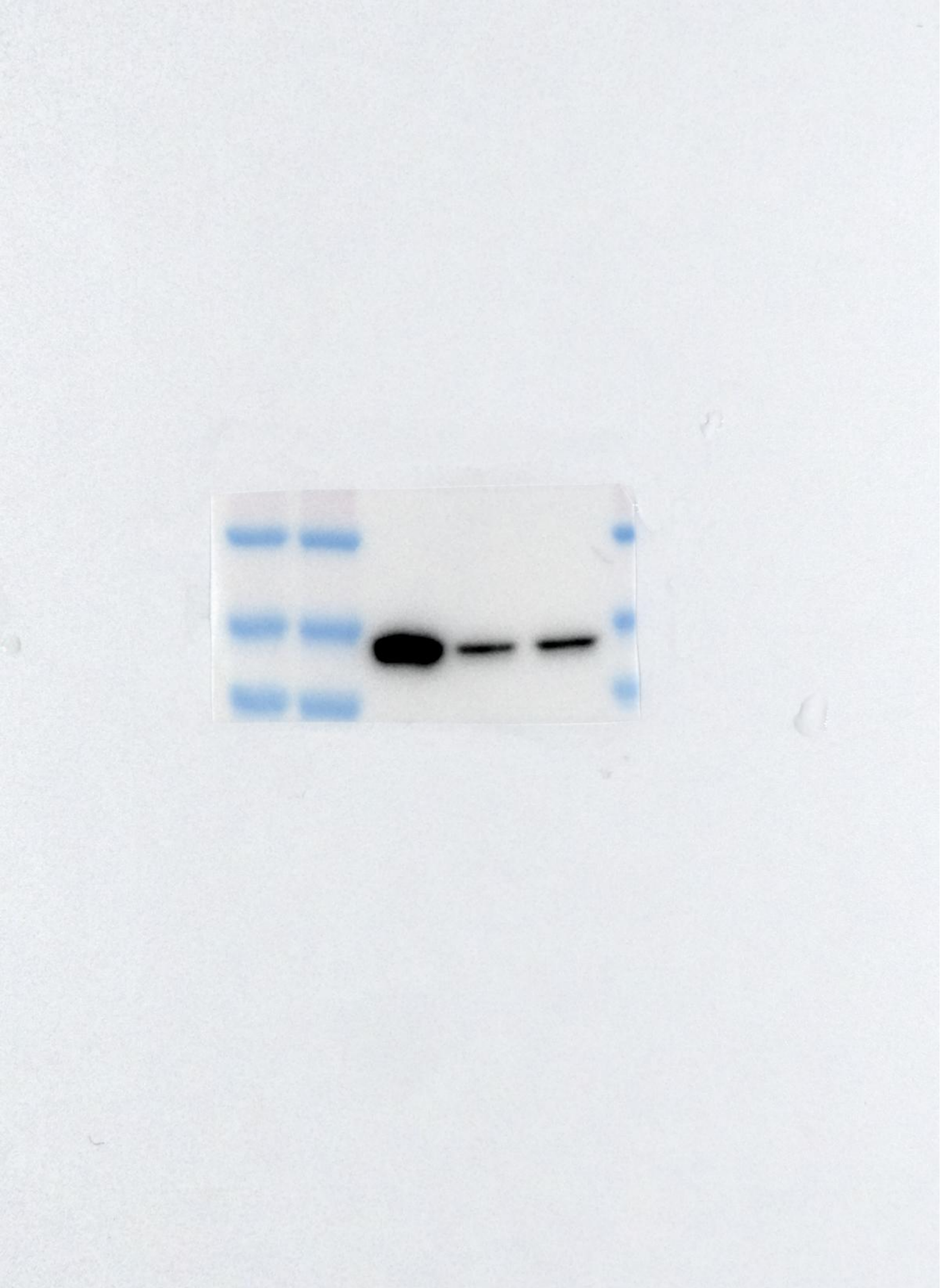

IMR-32-GAPDH

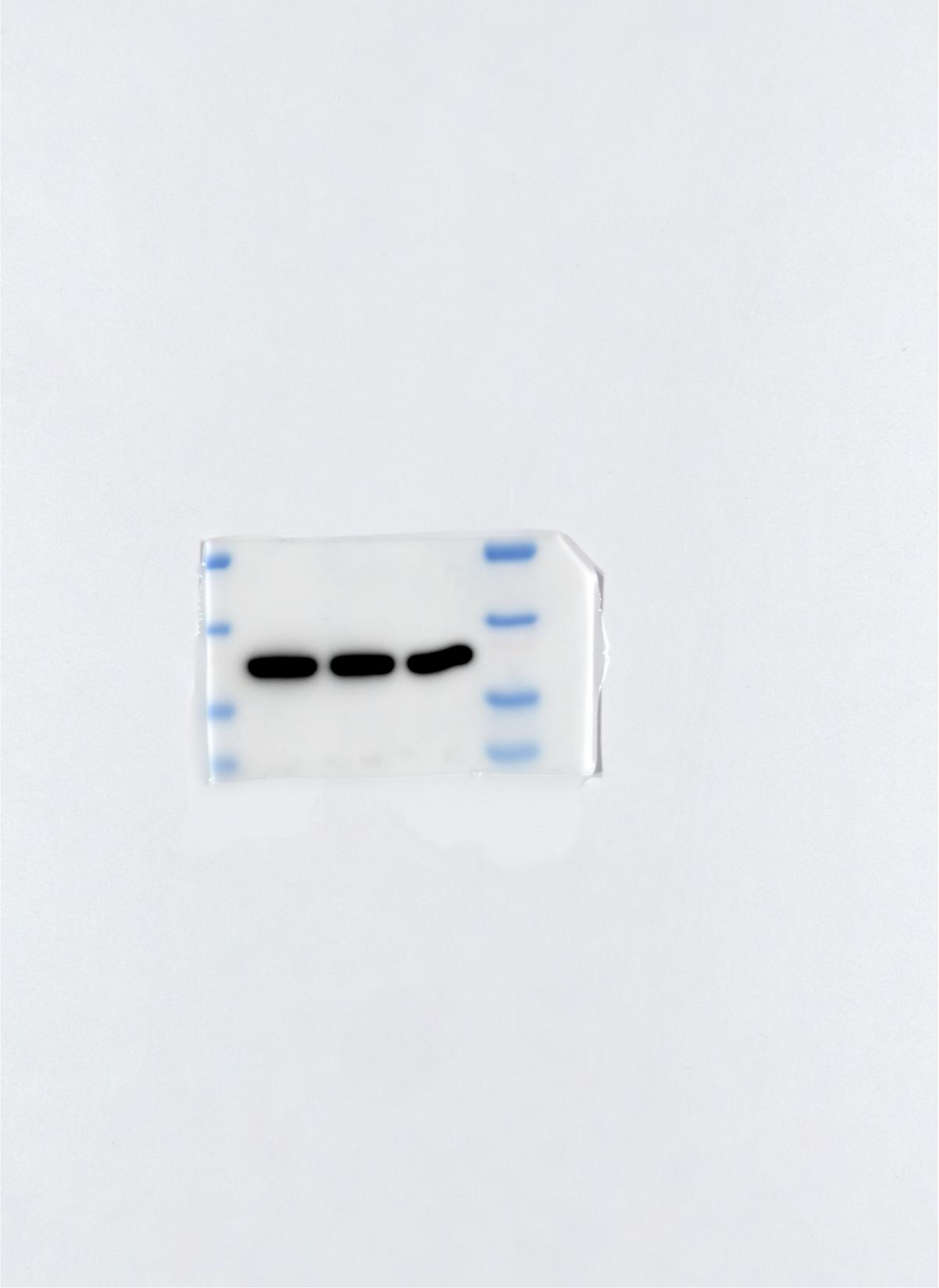

BE(2)-M17-NeuroD1

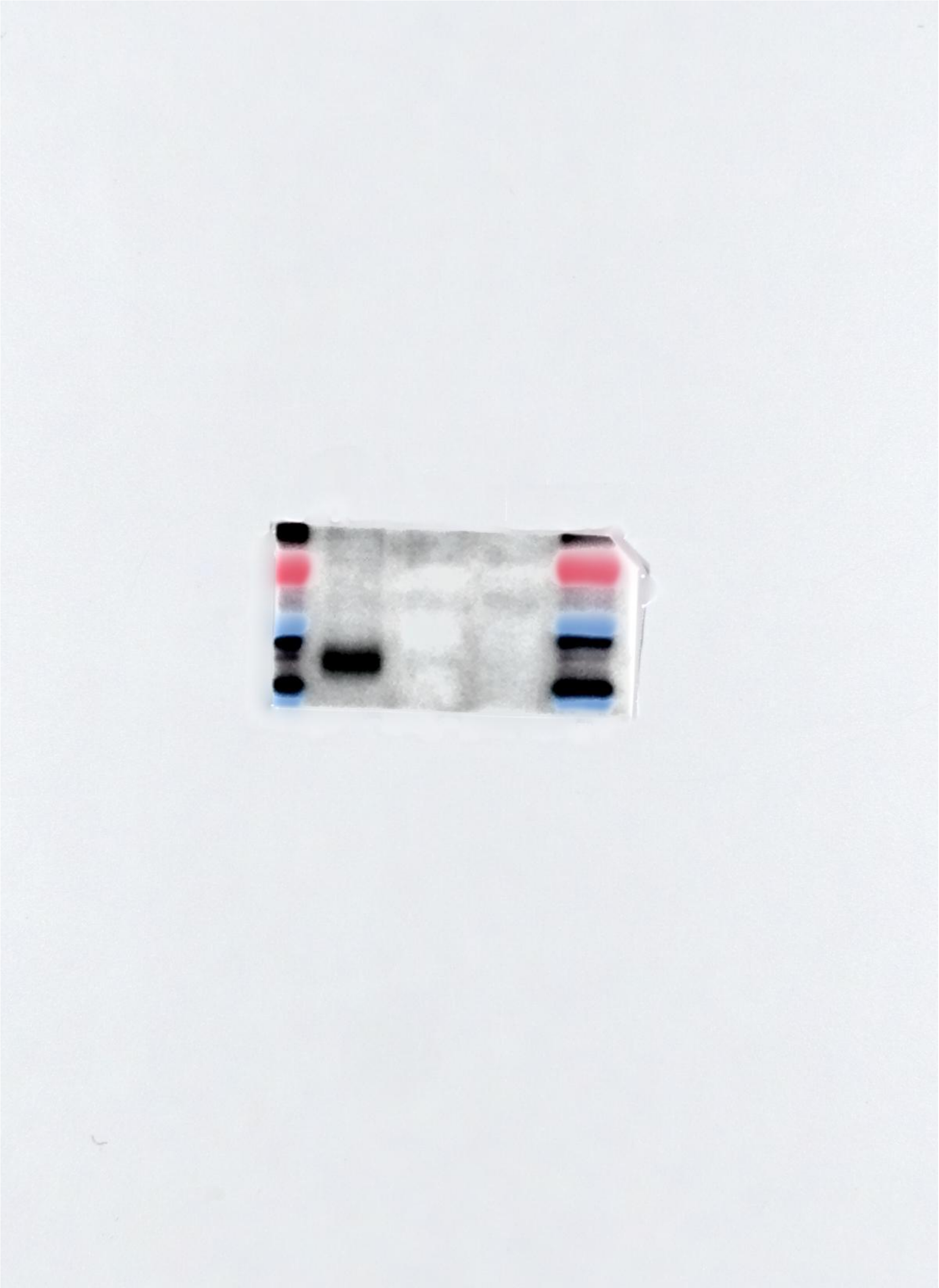

BE(2)-M17-CyclinD1

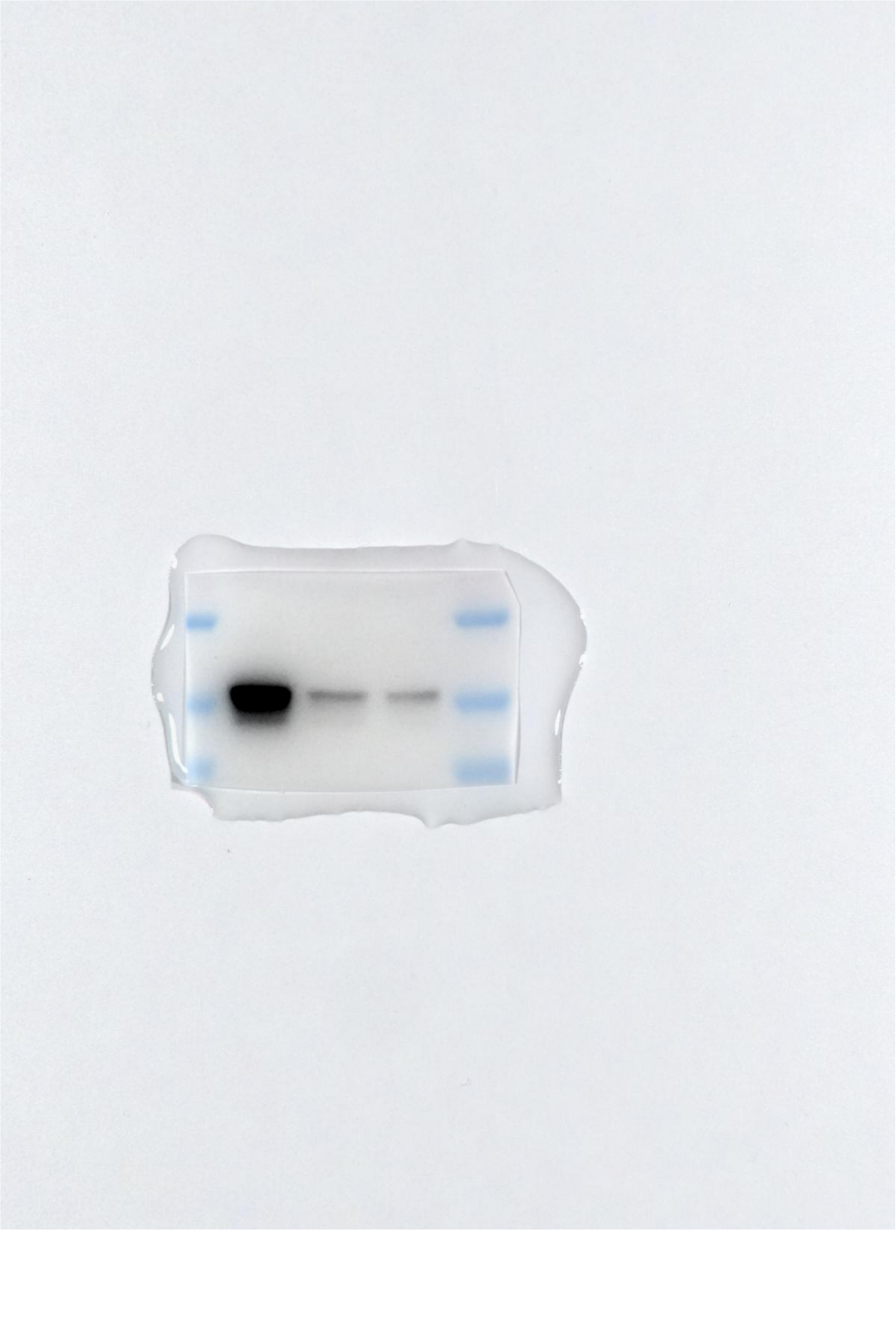

BE(2)-M17-CDK4

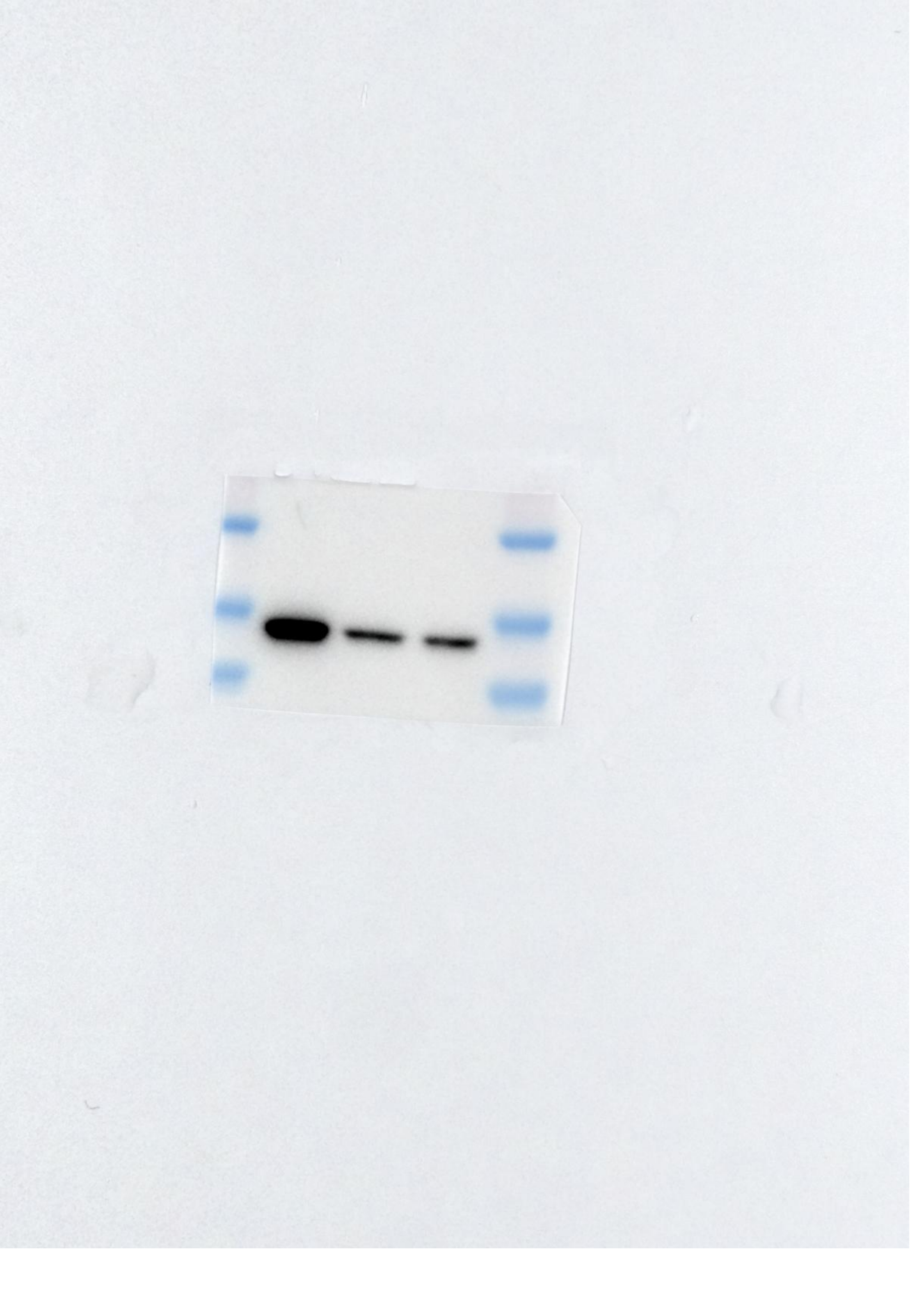

BE(2)-M17-GAPDH

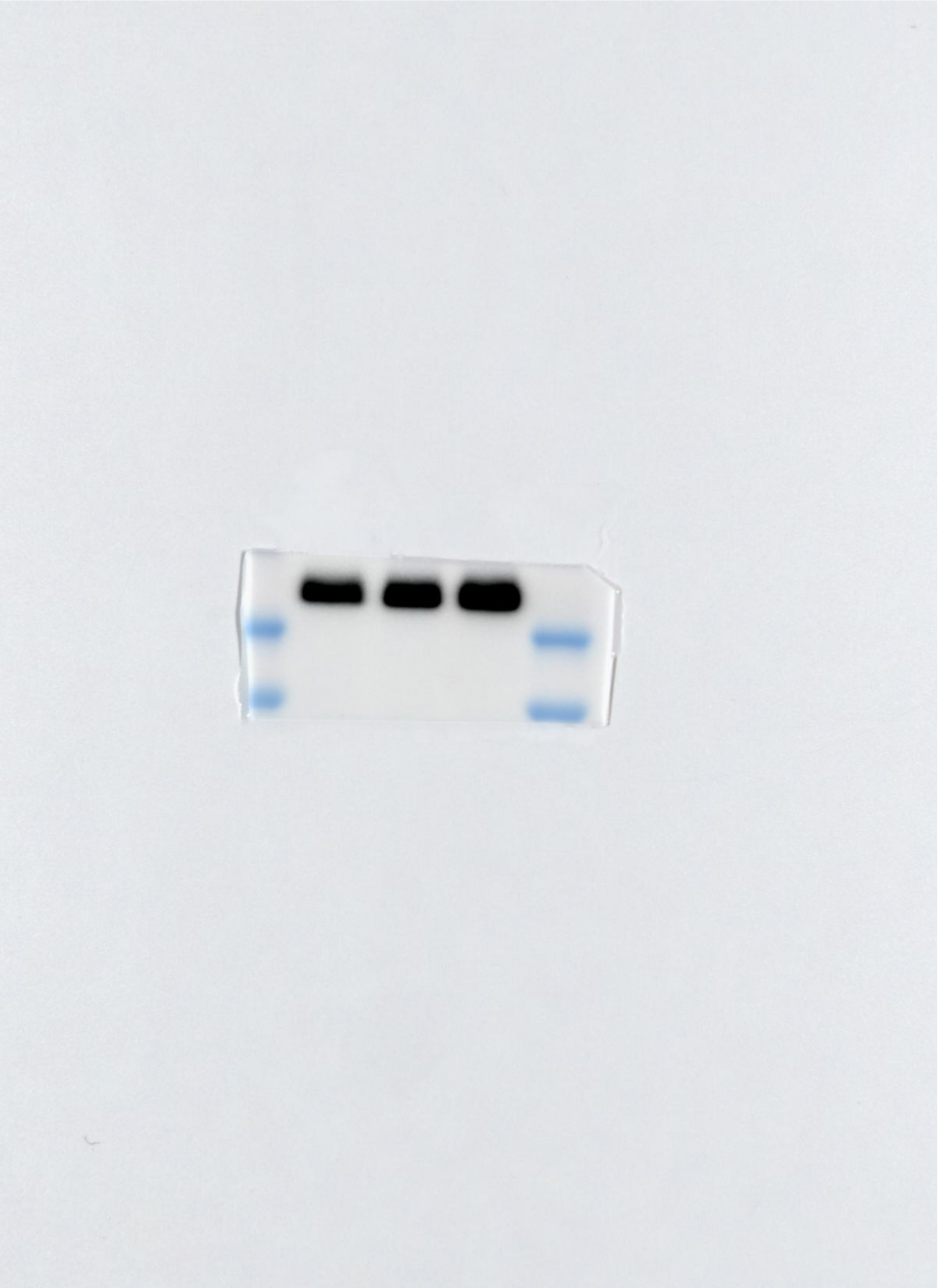

SK-N-DZ-NeuroD1

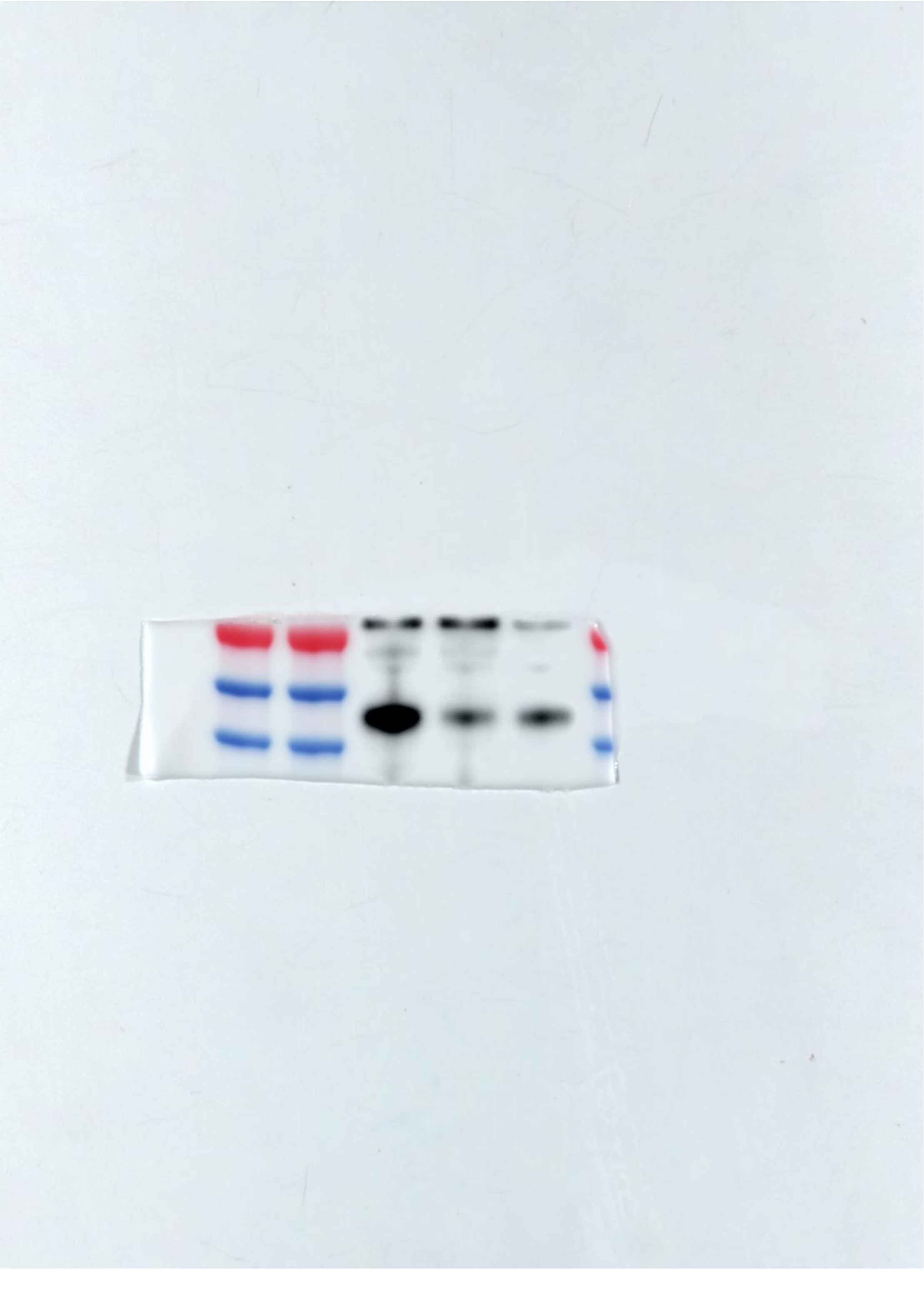

SK-N-DZ-CyclinD1

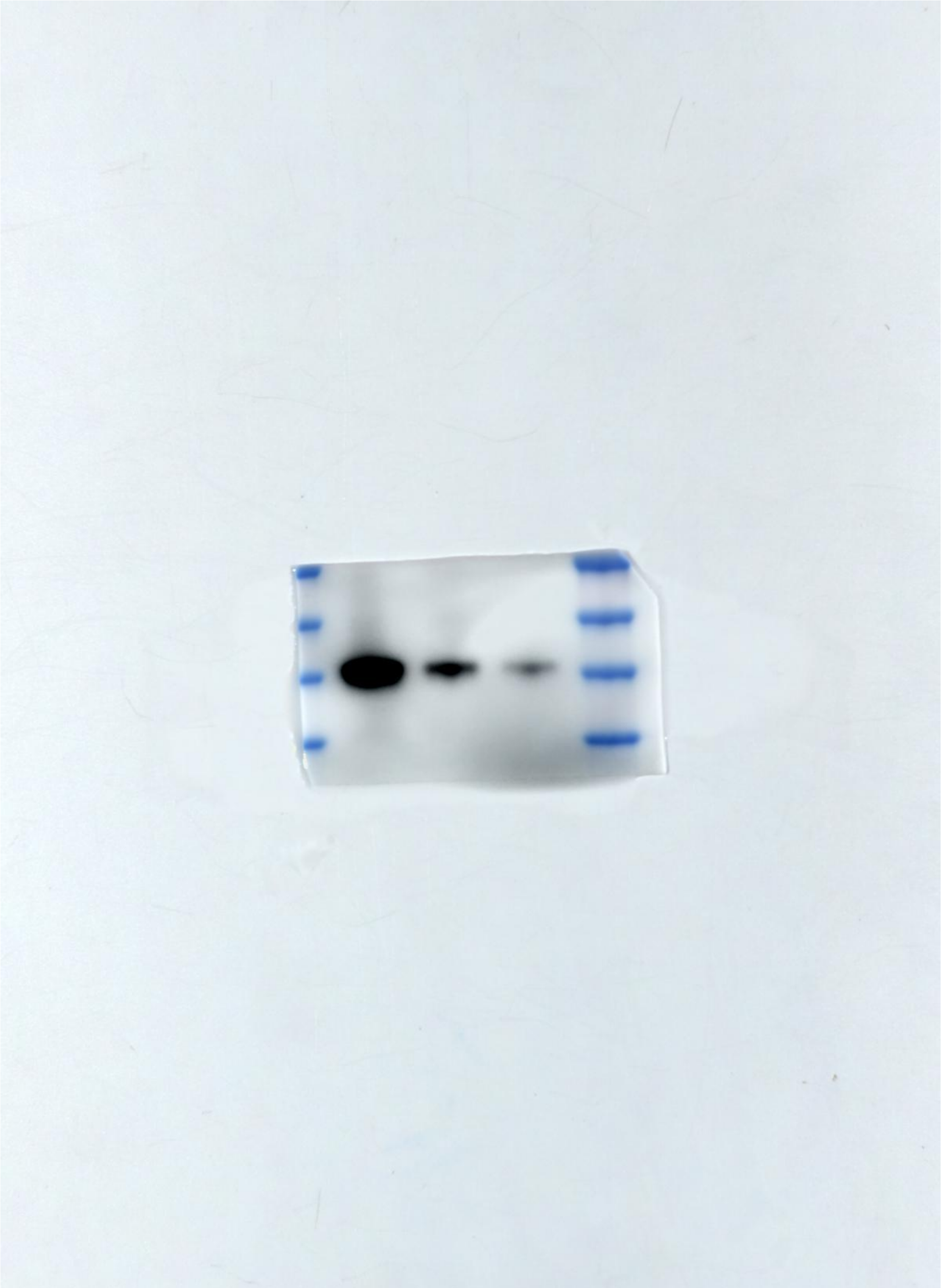

SK-N-DZ-CDK4

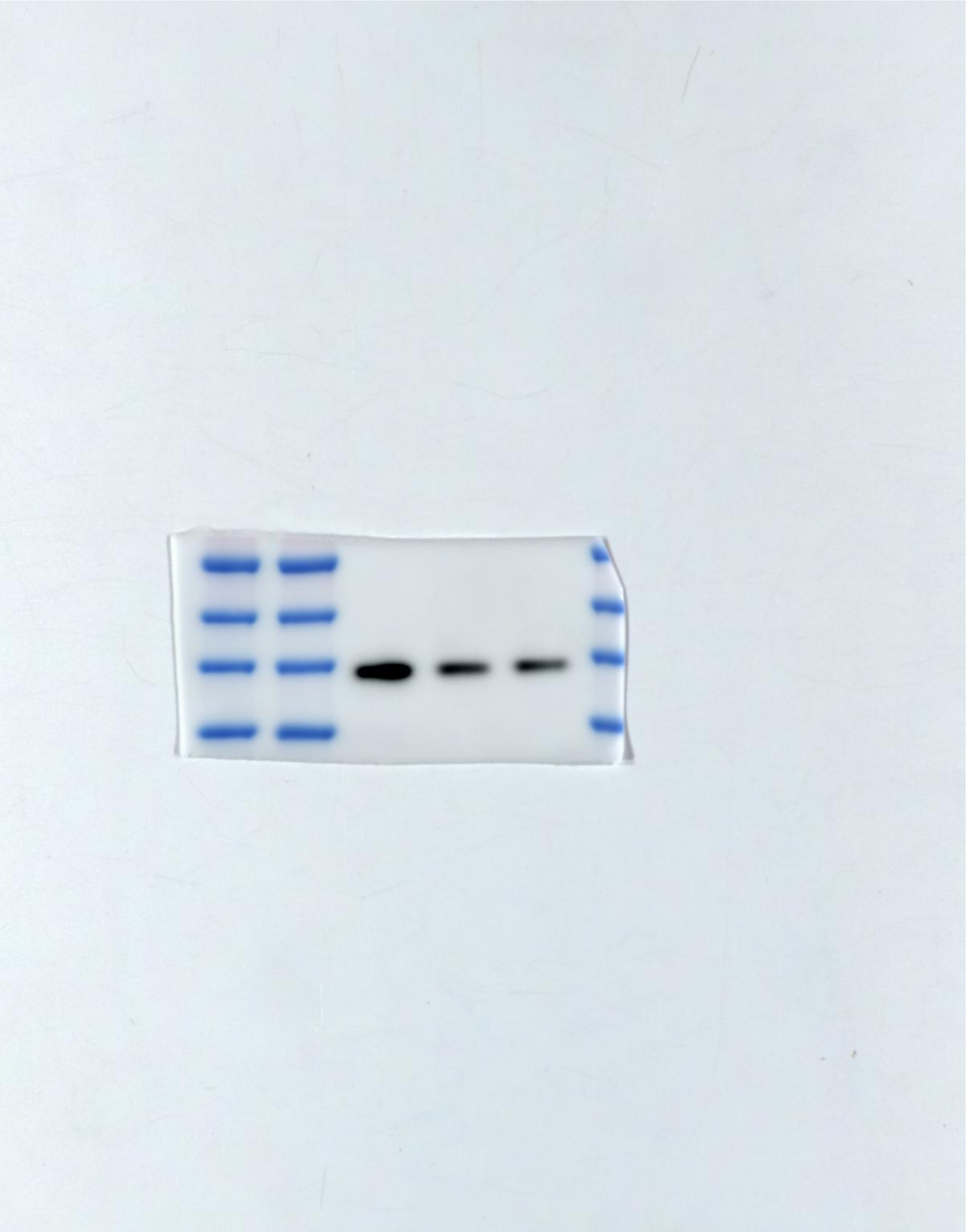

SK-N-DZ-GAPDH

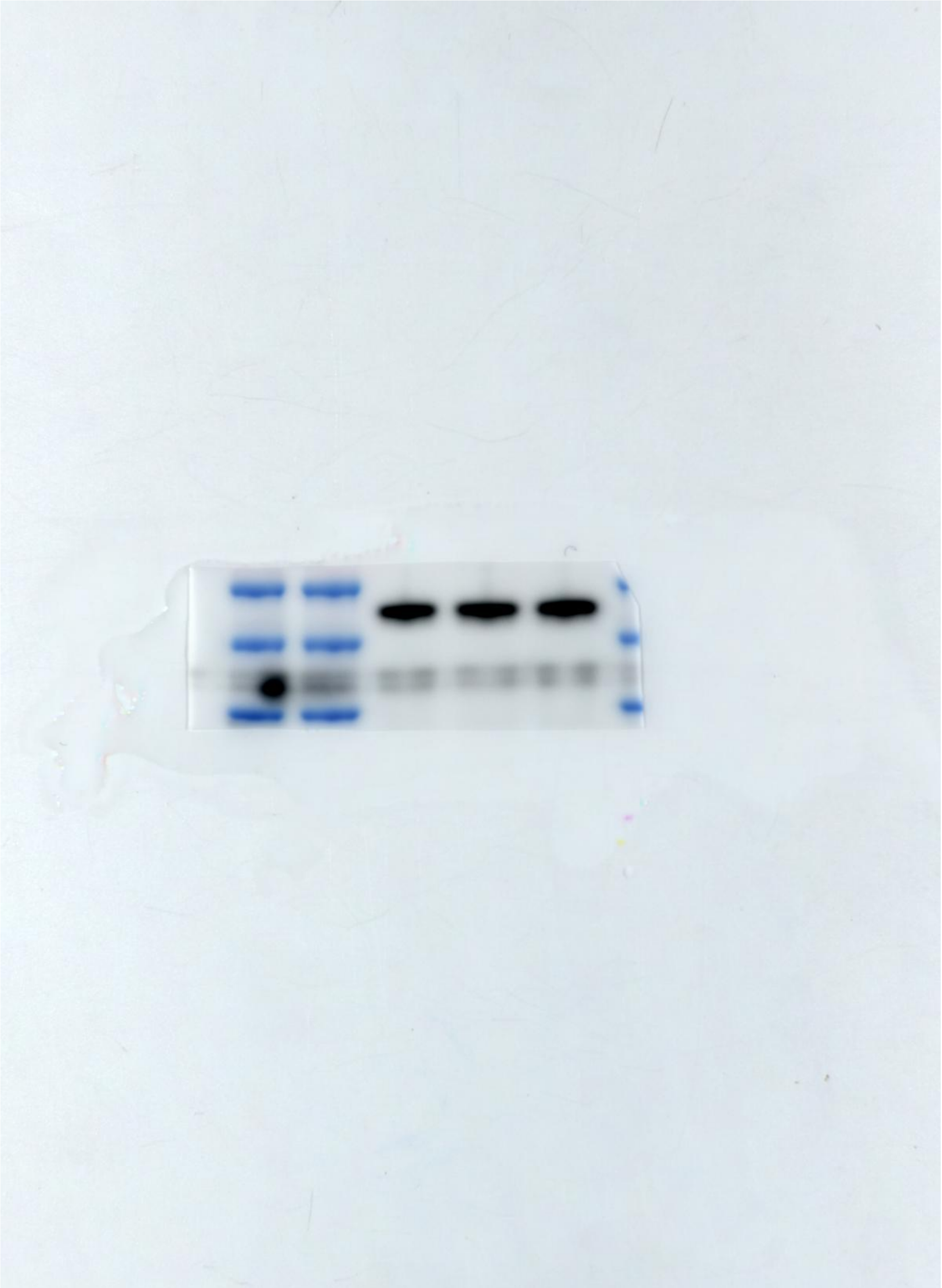

I

NeuroD1

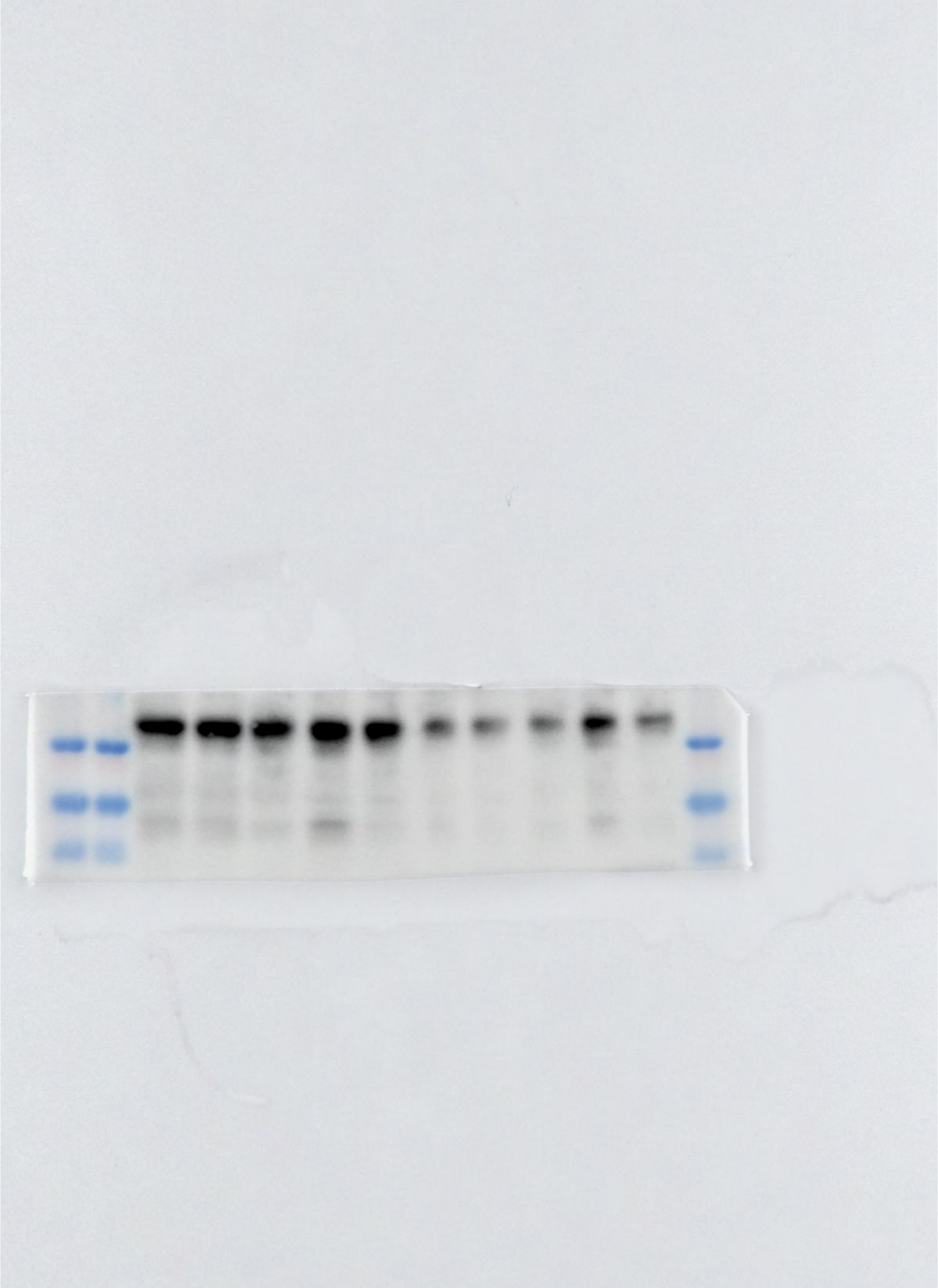

N-Myc

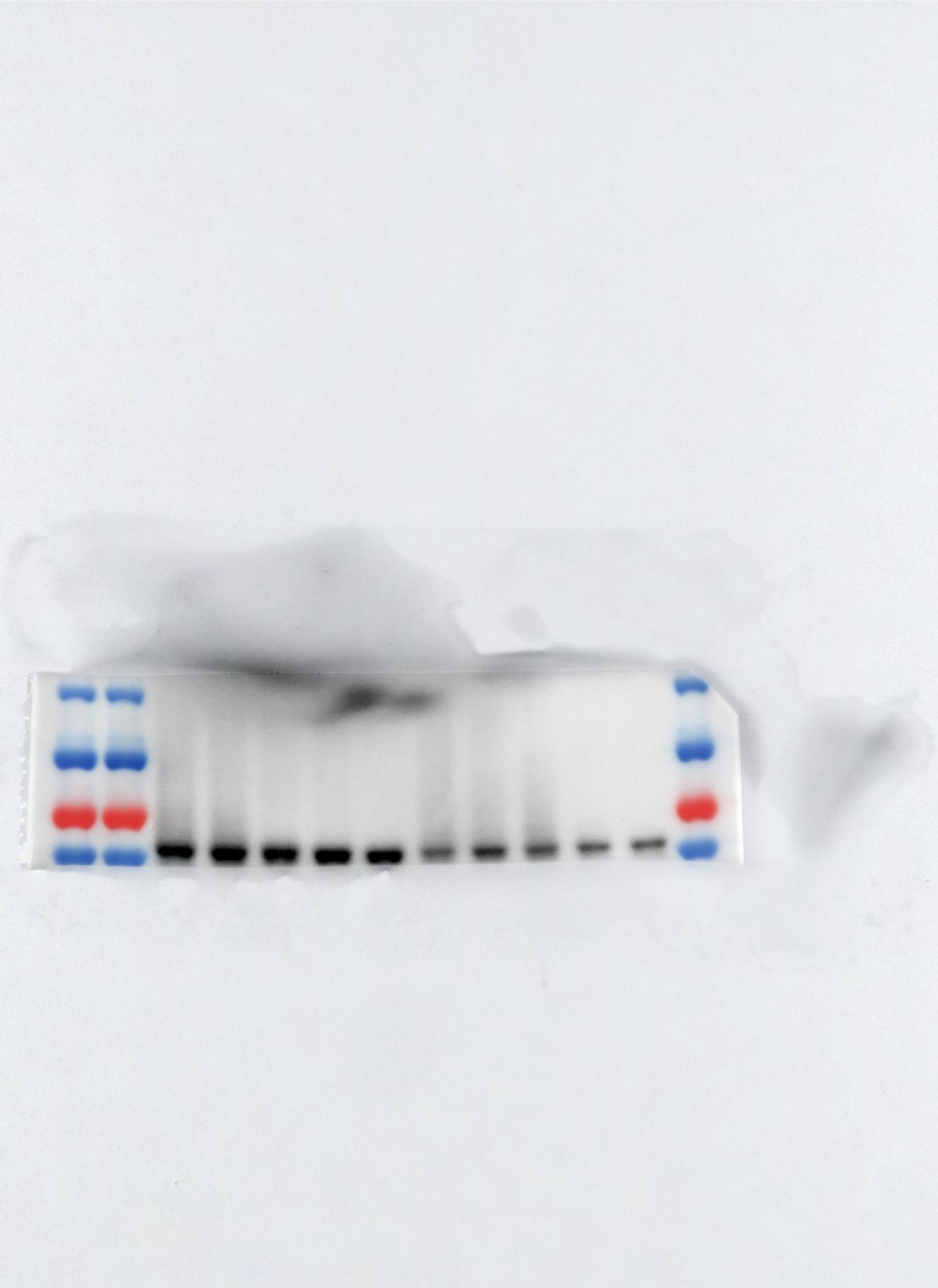

PCNA

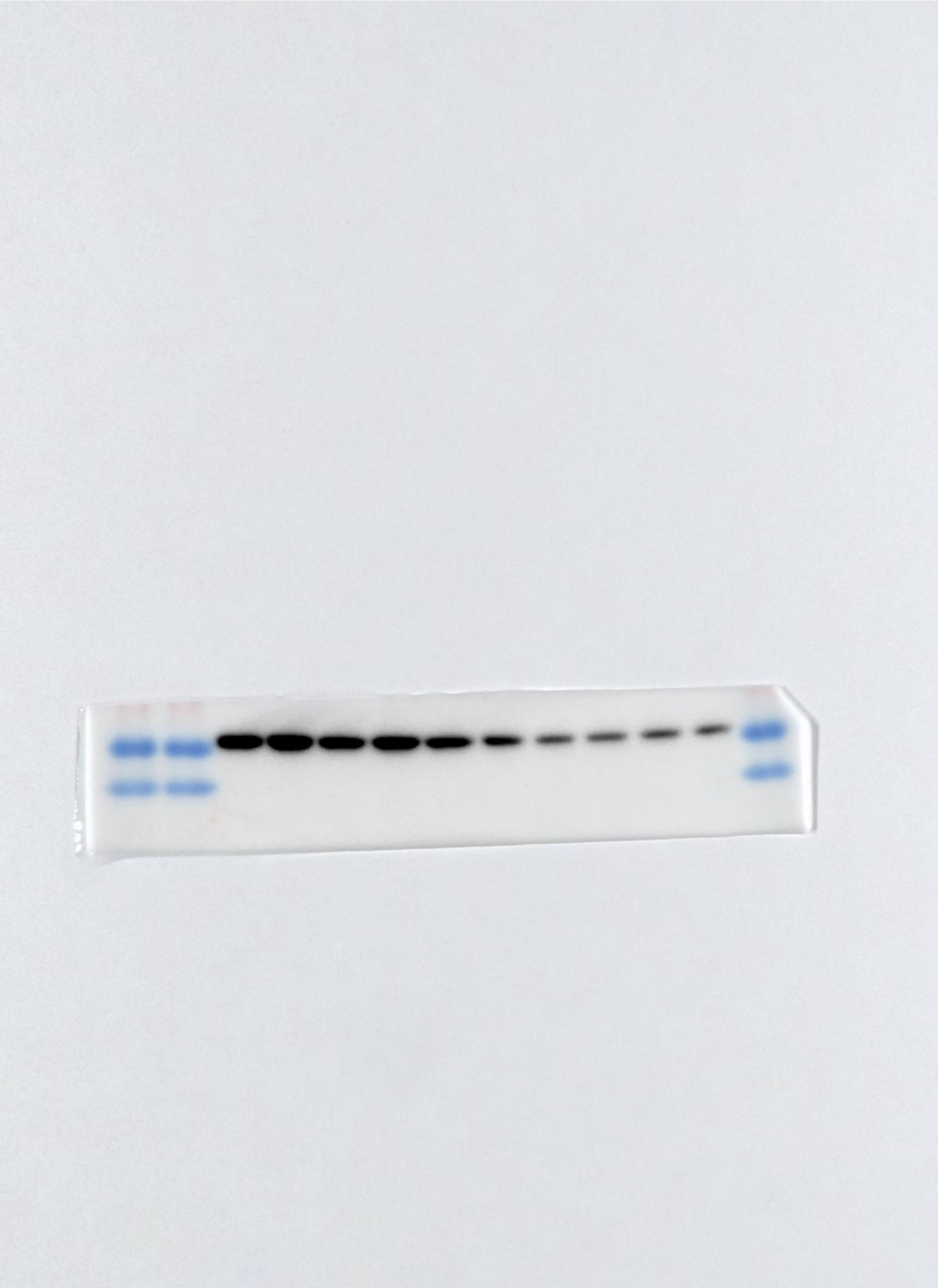

GAPDH

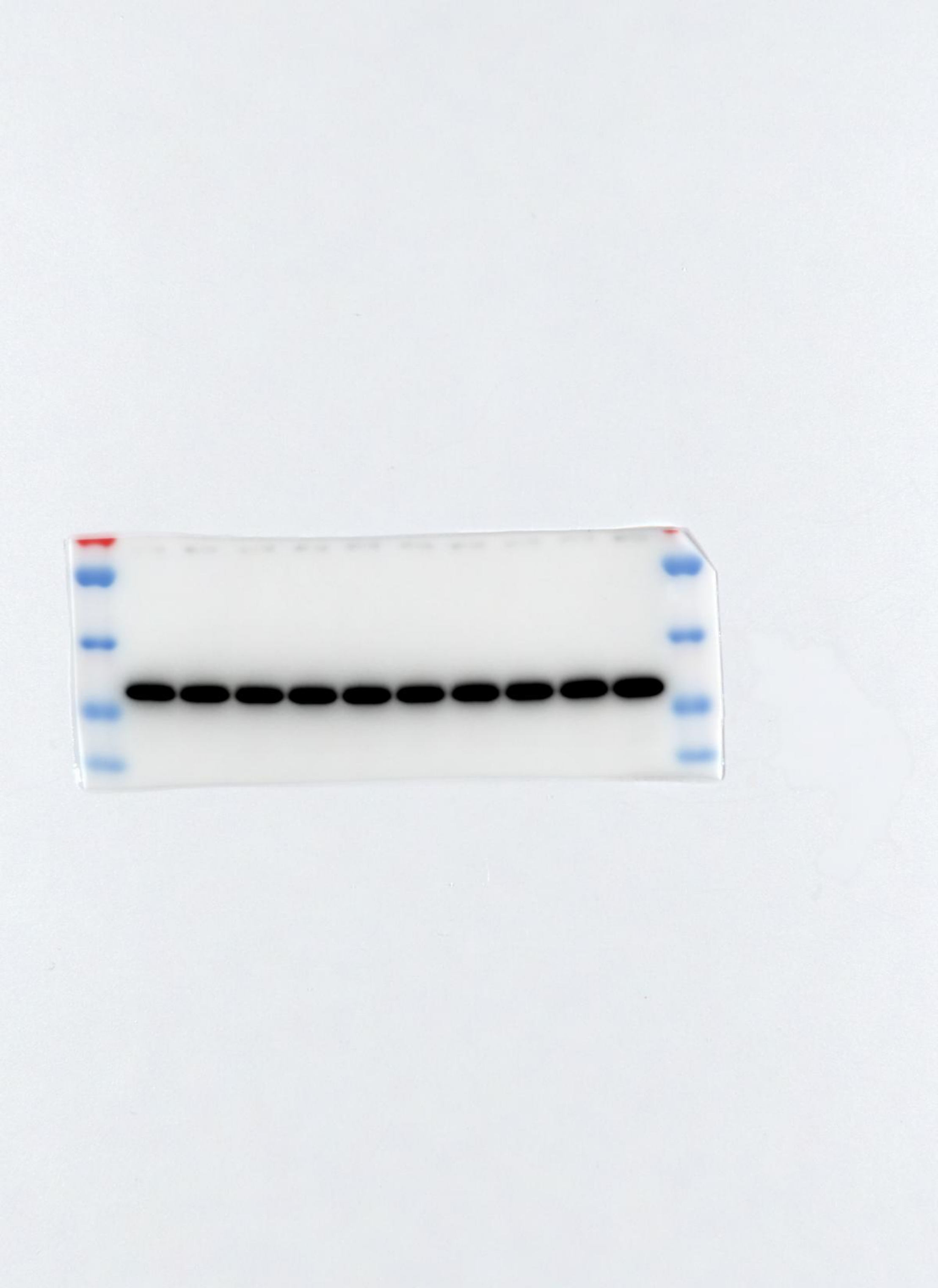

FIgure 3

IMR-32-NeuroD1

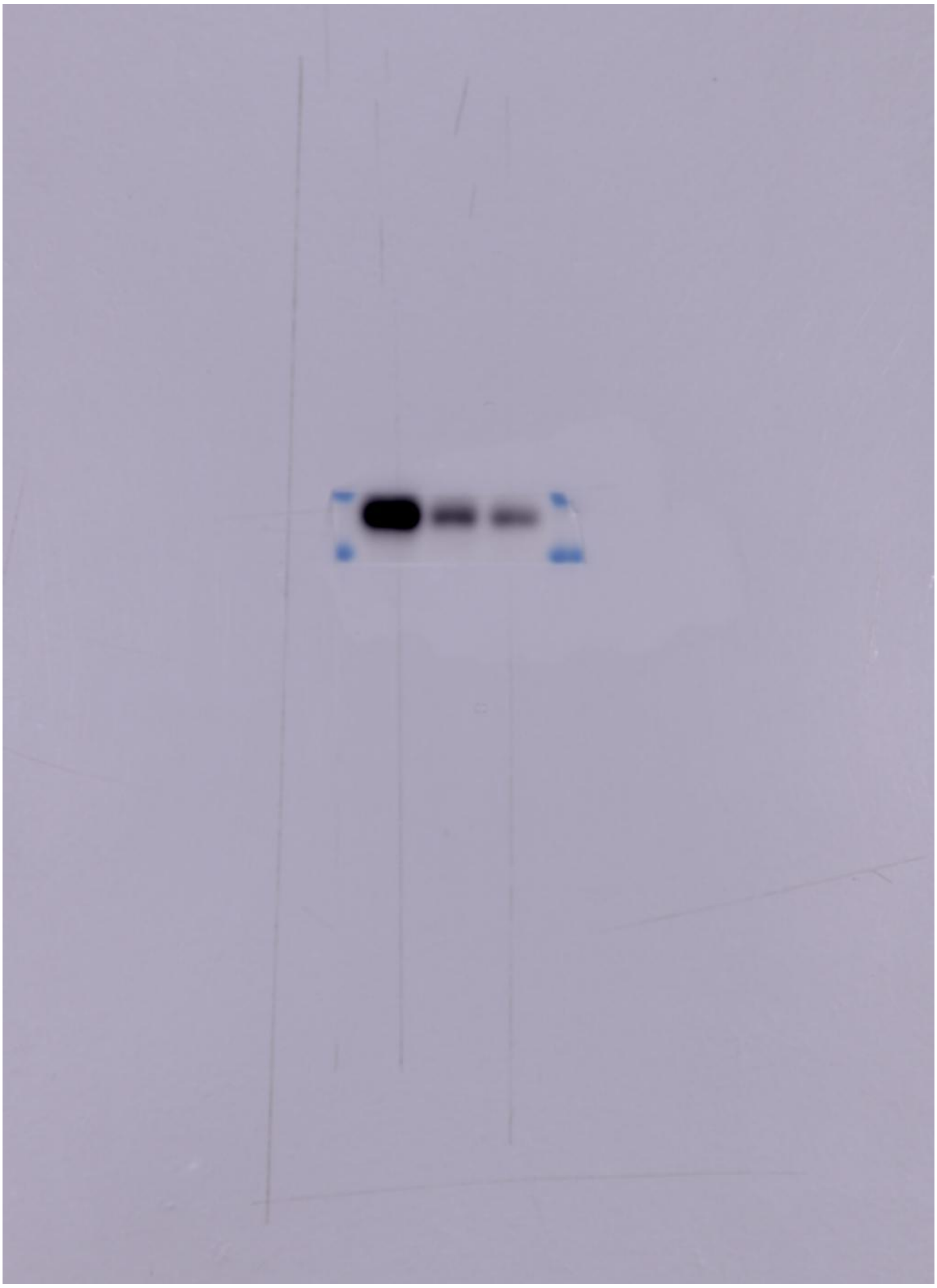

IMR-32-N-Myc

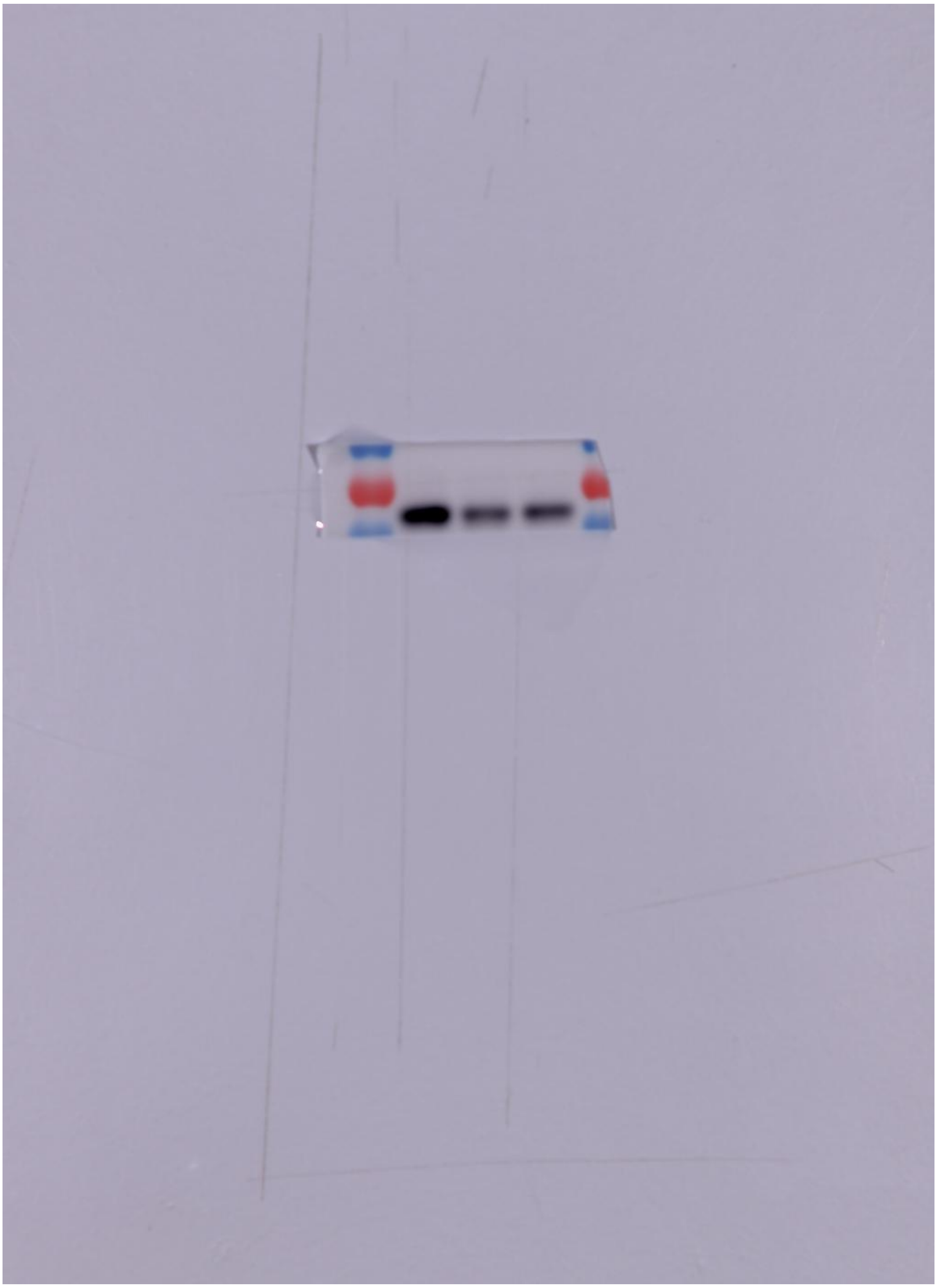

IMR-32-GAPDH

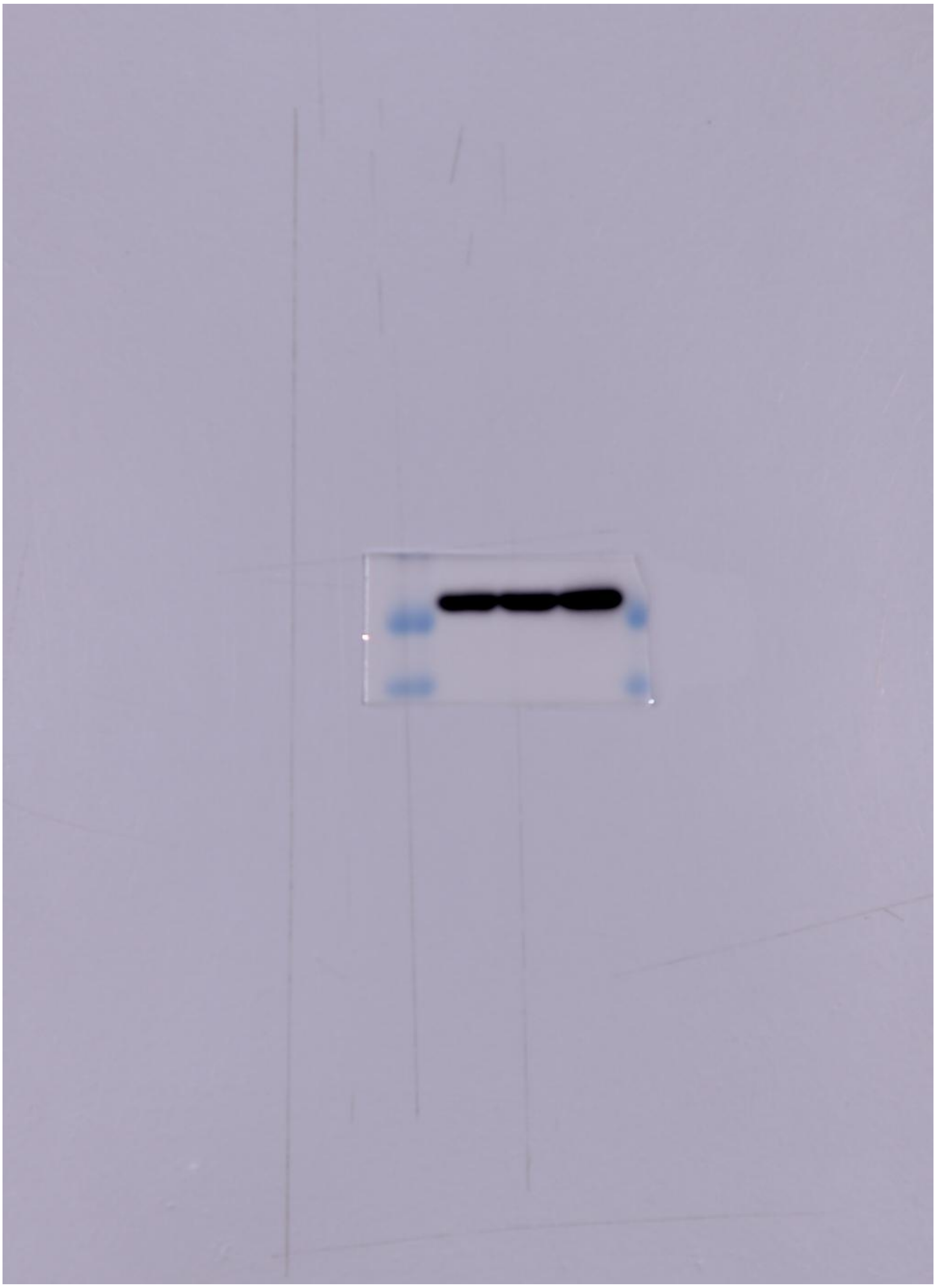

BE(2)-M17-NeuroD1

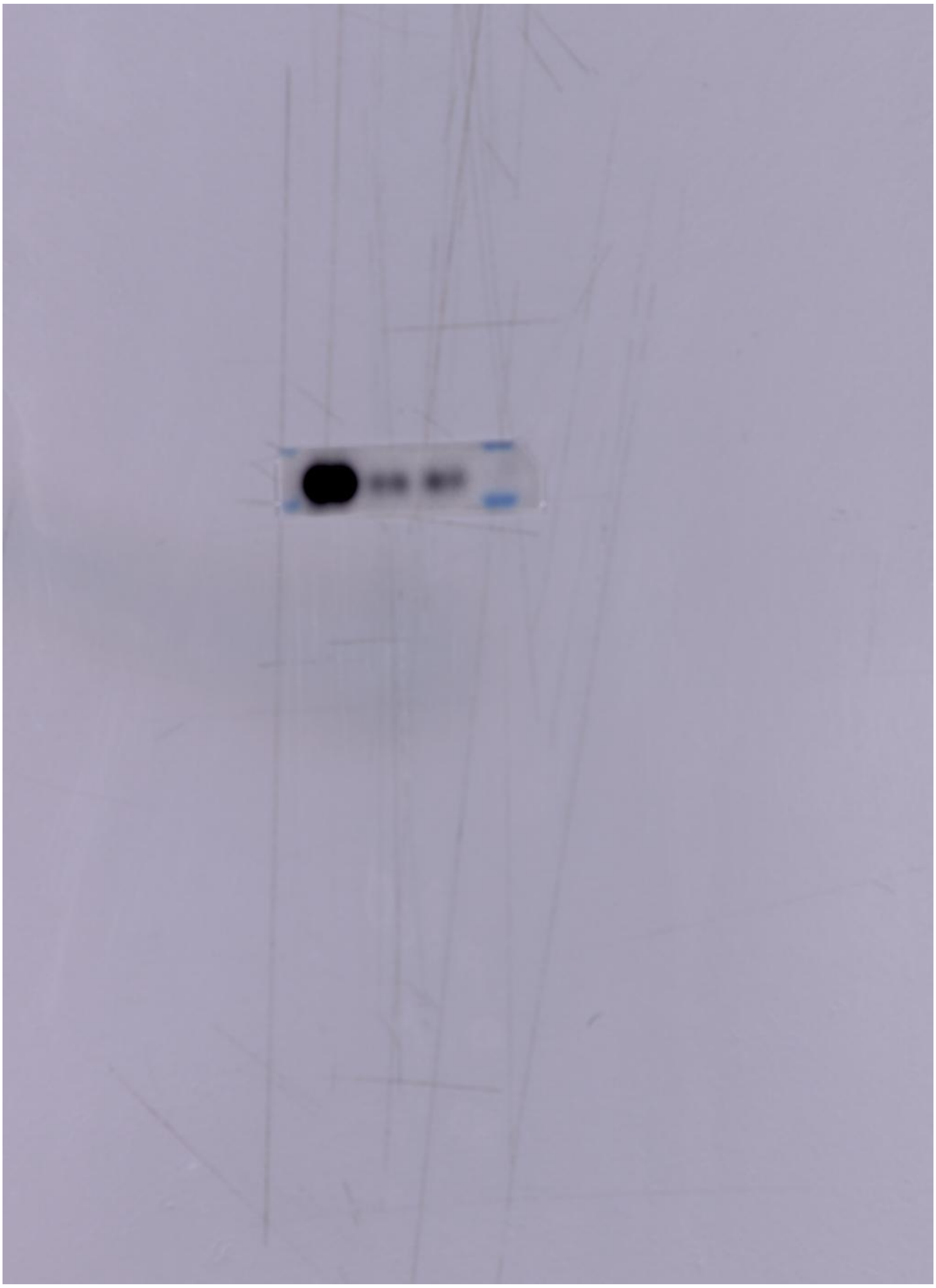

BE(2)-M17-N-Myc

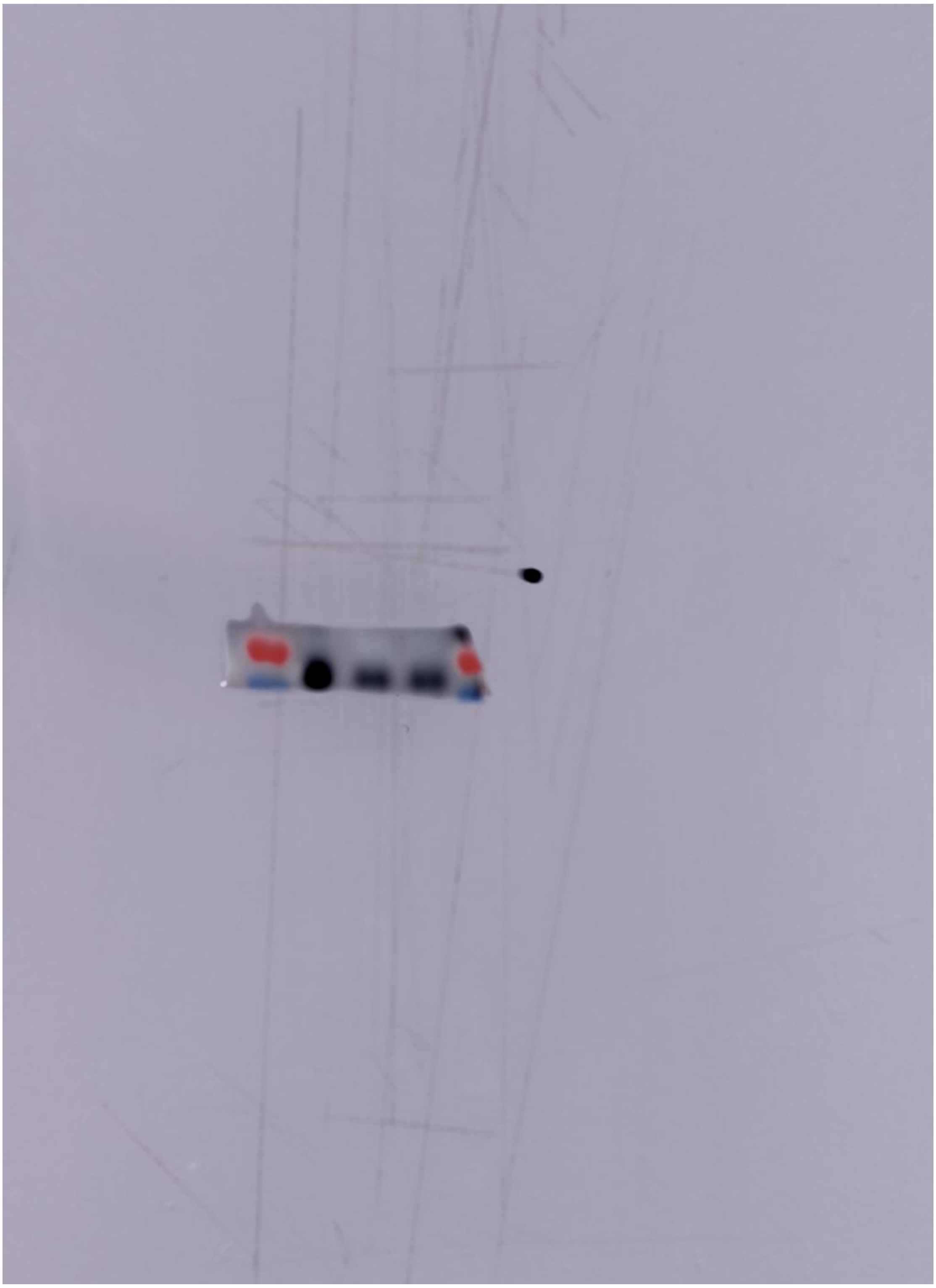

BE(2)-M17-GAPDH

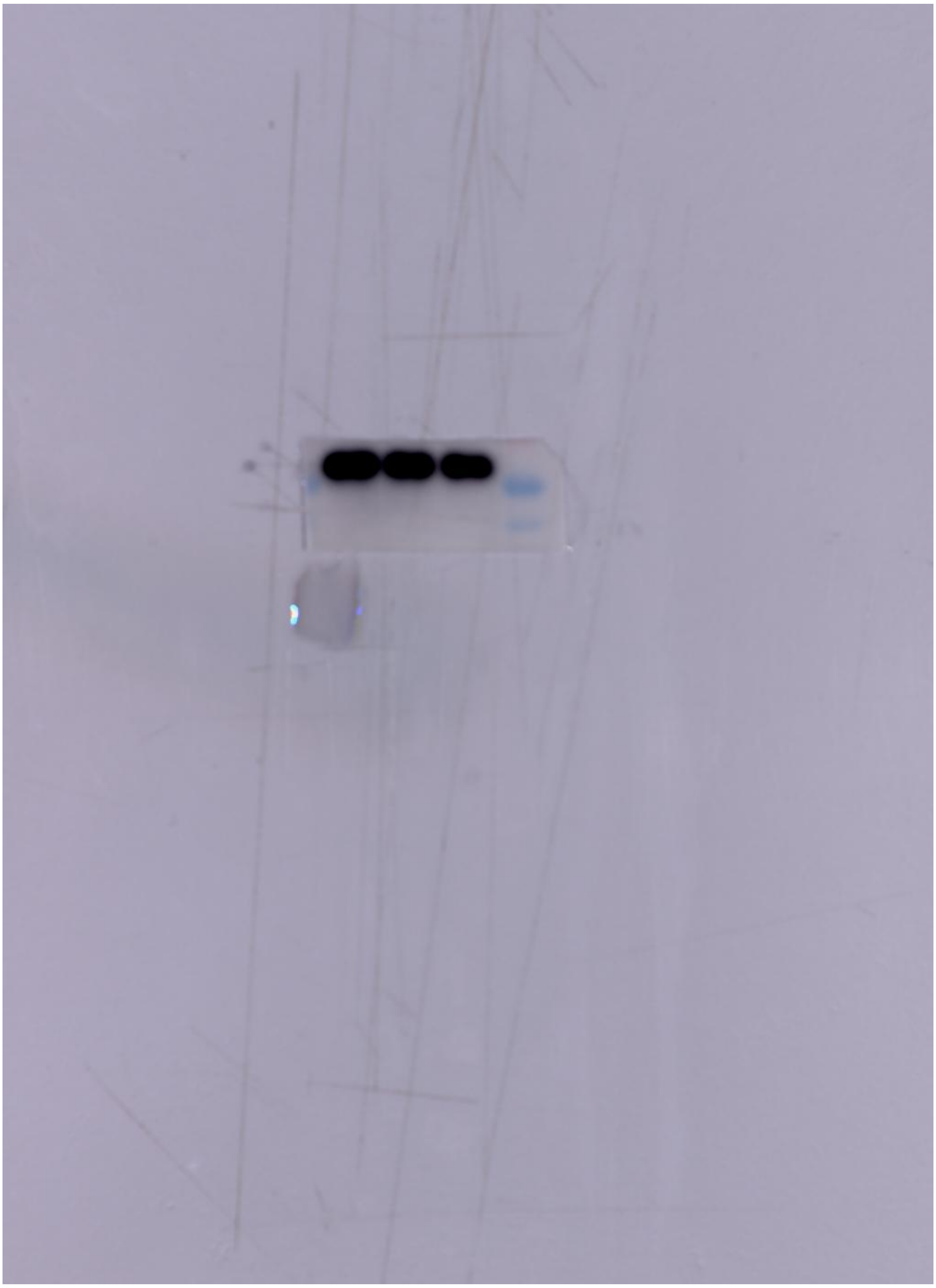

SK-N-DZ-NeuroD1

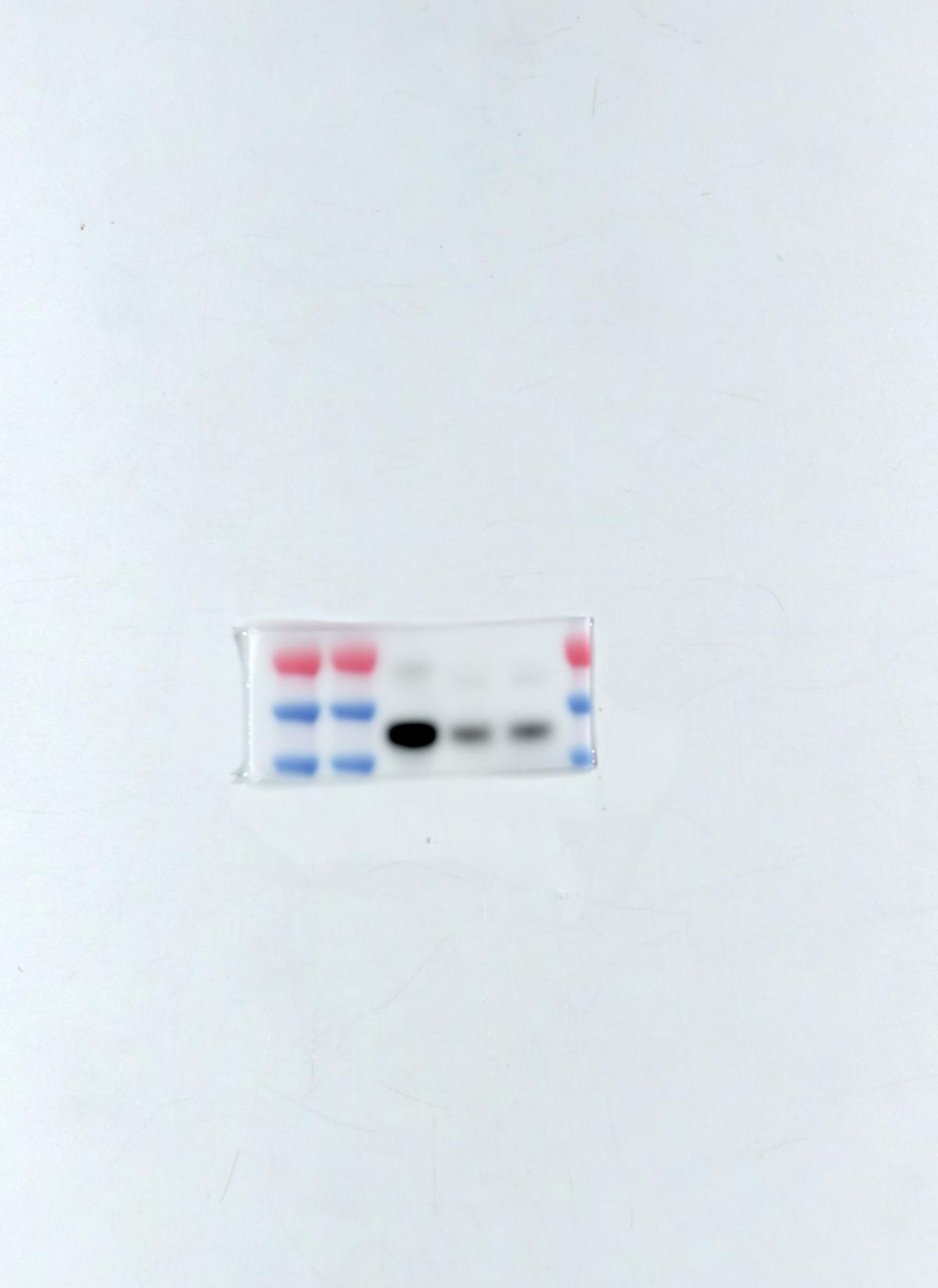

SK-N-DZ-N-Myc

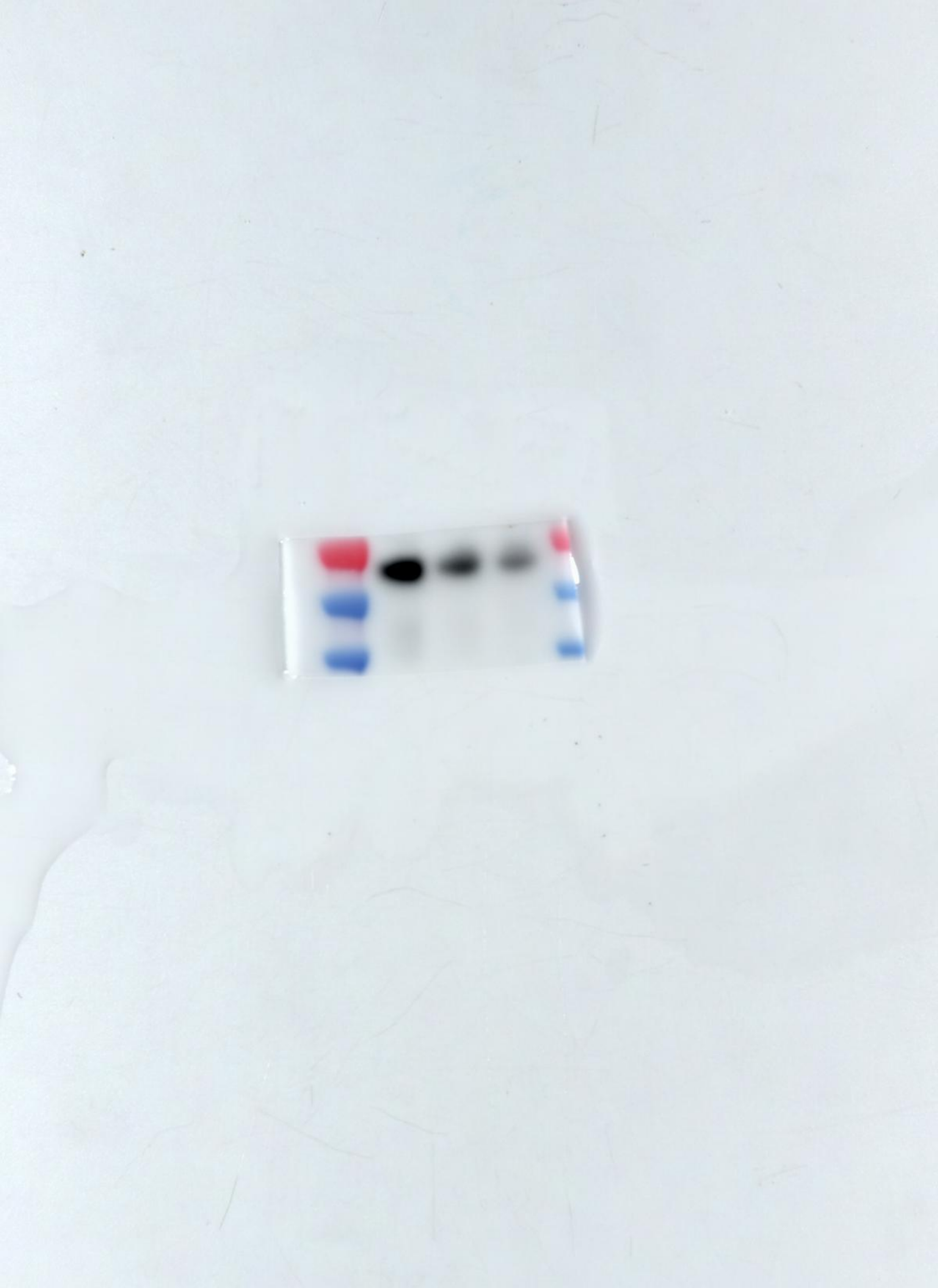

SK-N-DZ-GAPDH

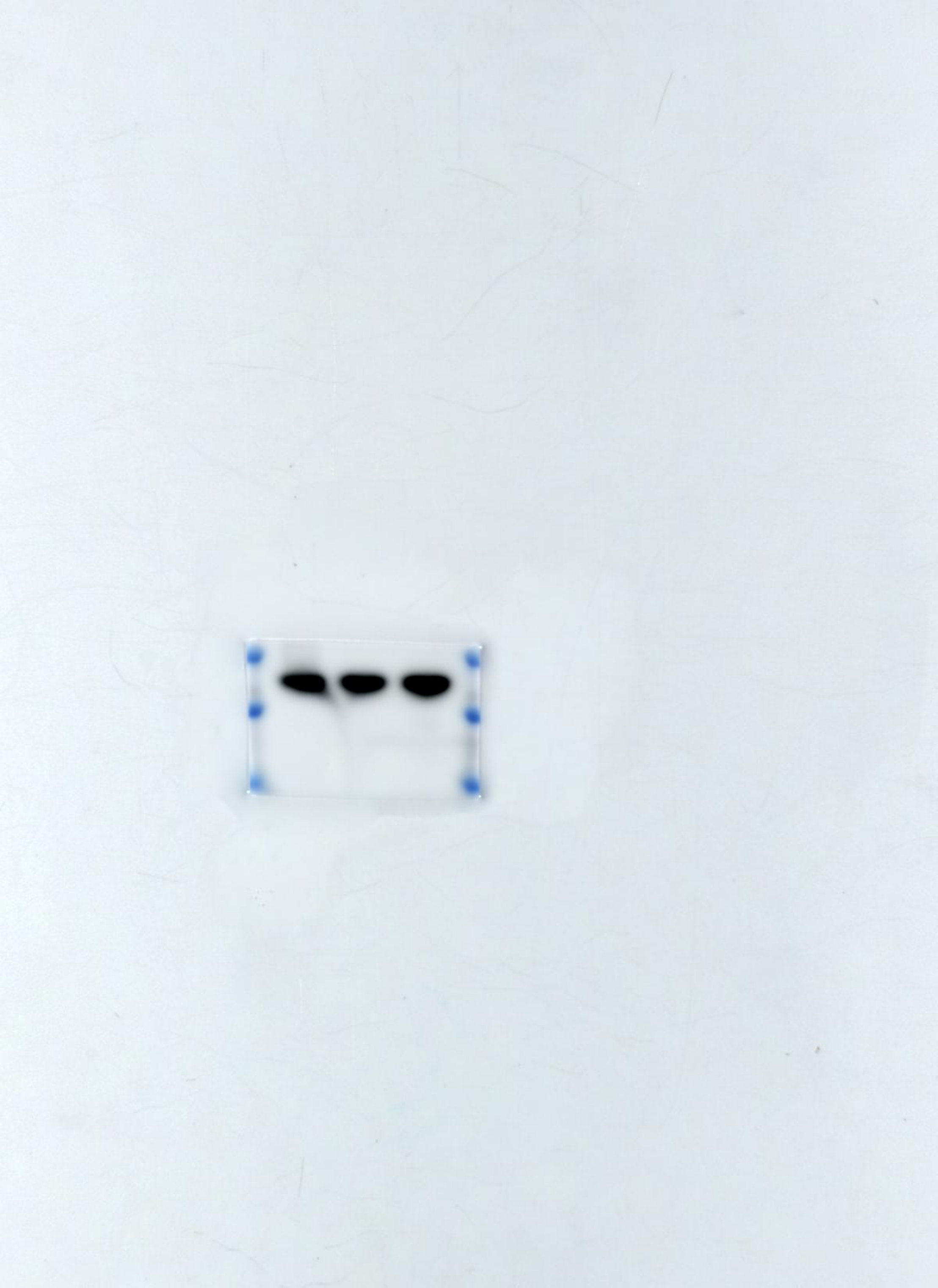

FIgure 4

A

IMR-32-NeuroD1

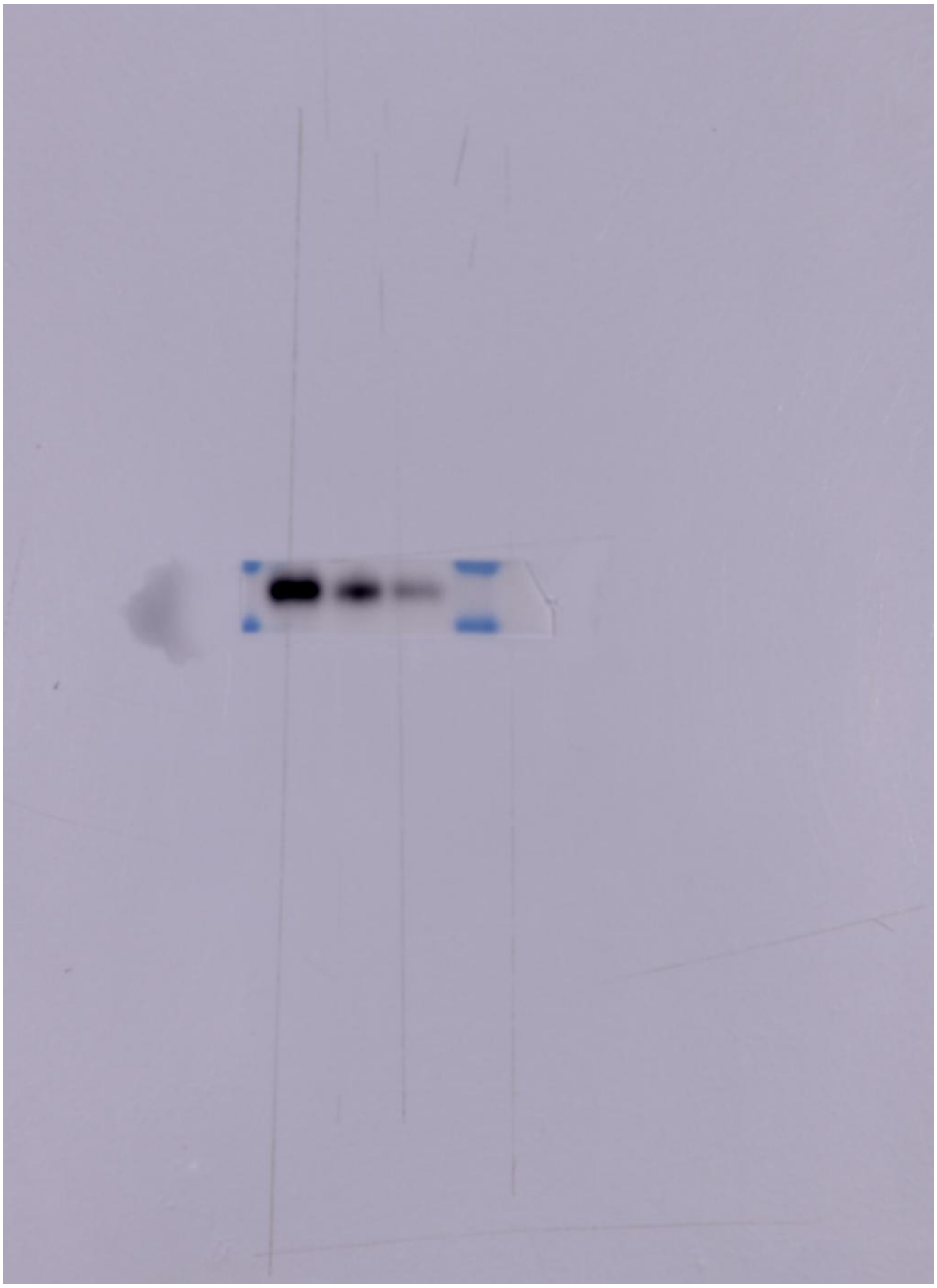

IMR-32-N-Myc

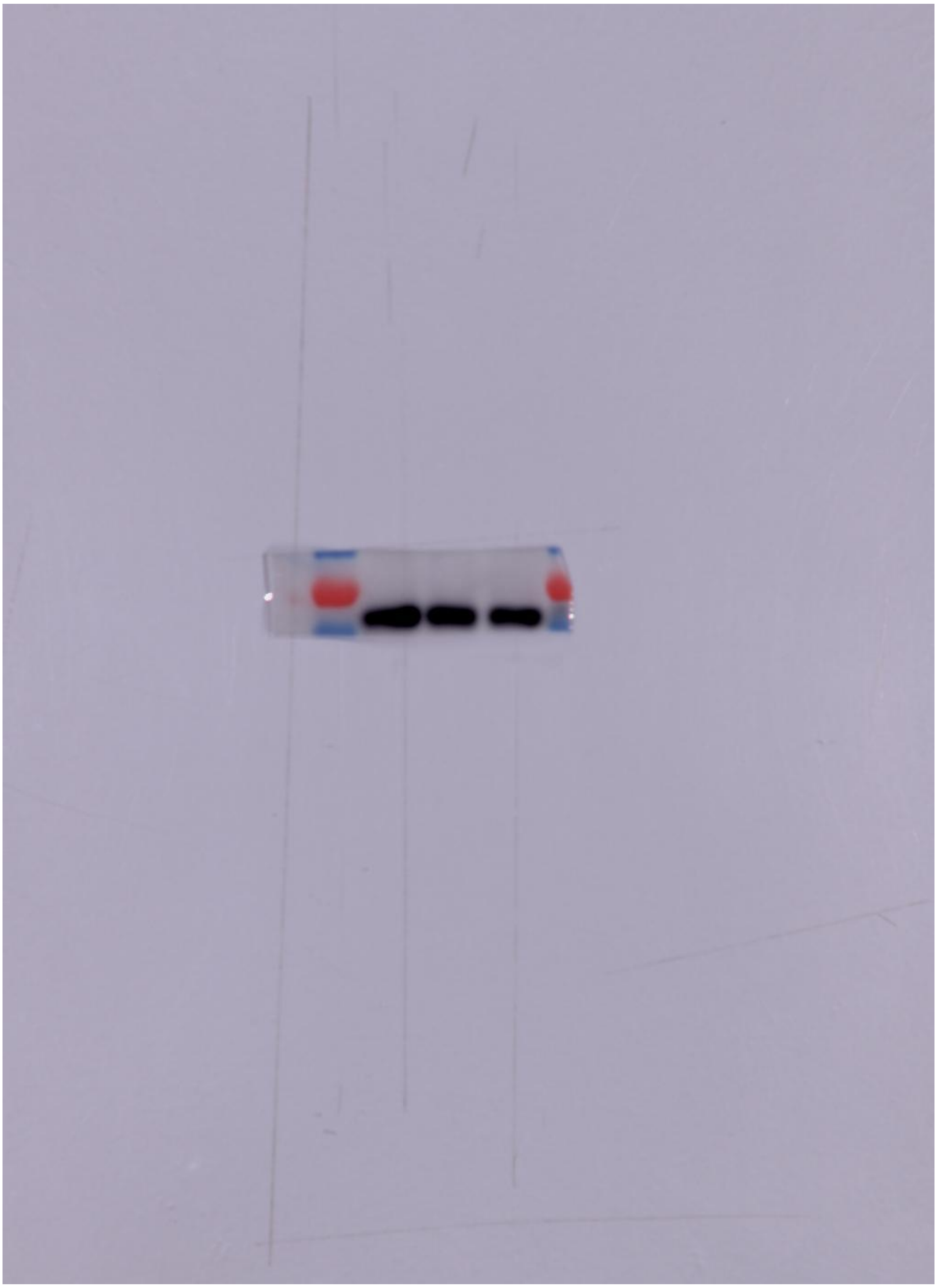

IMR-32-GAPDH

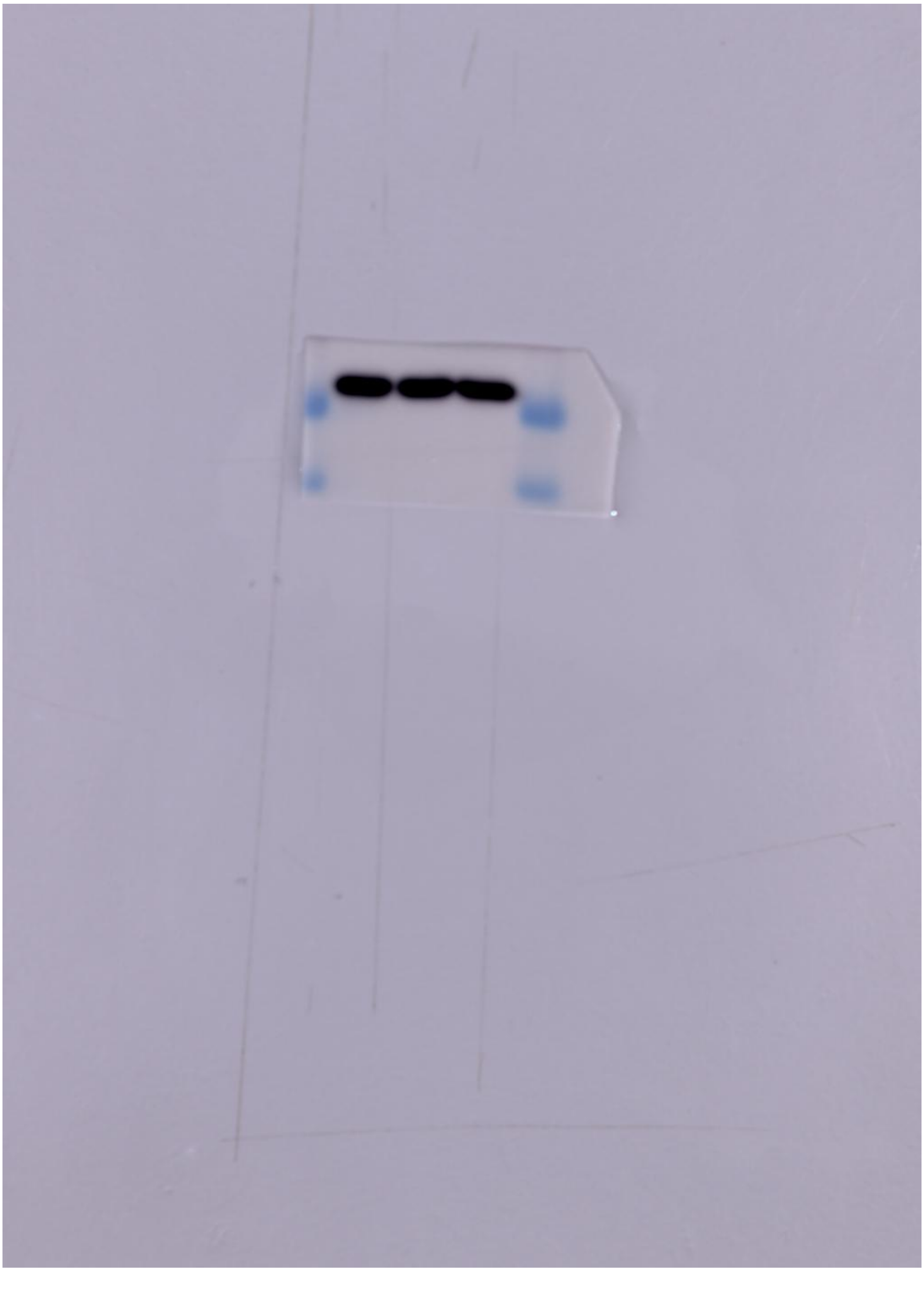

B

BE(2)-M17-NeuroD1

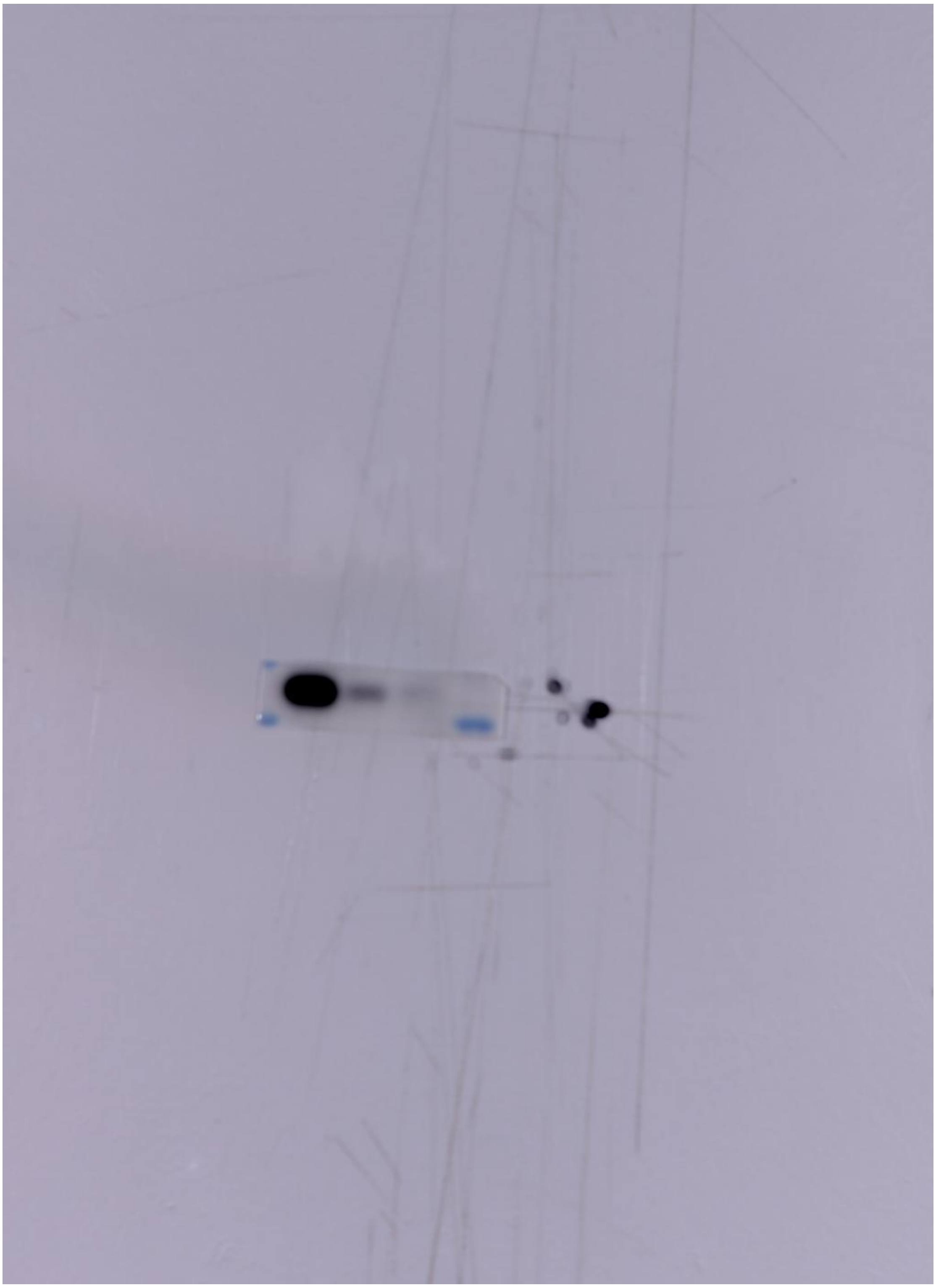

BE(2)-M17-N-myc

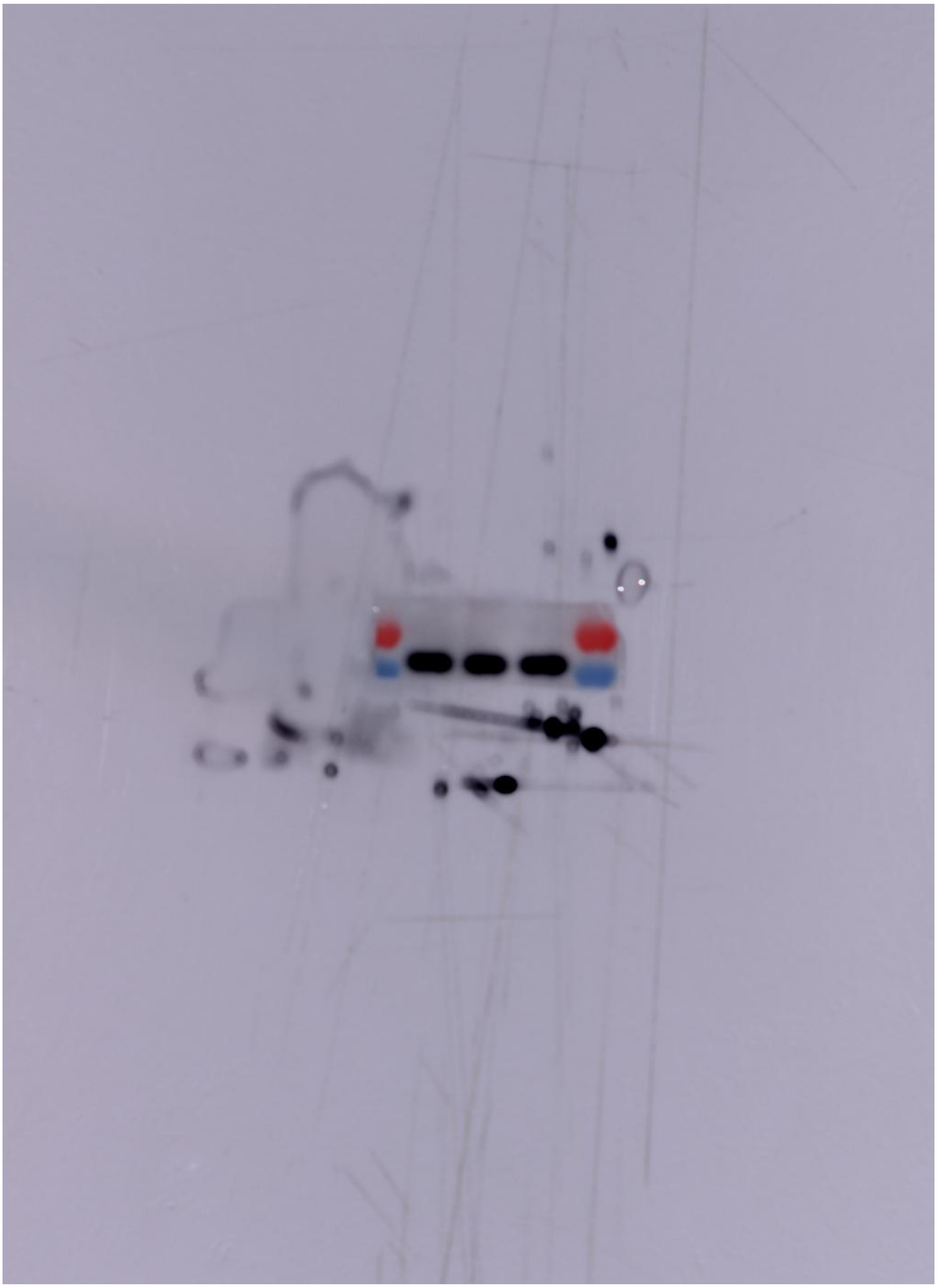

BE(2)-M17-GAPDH

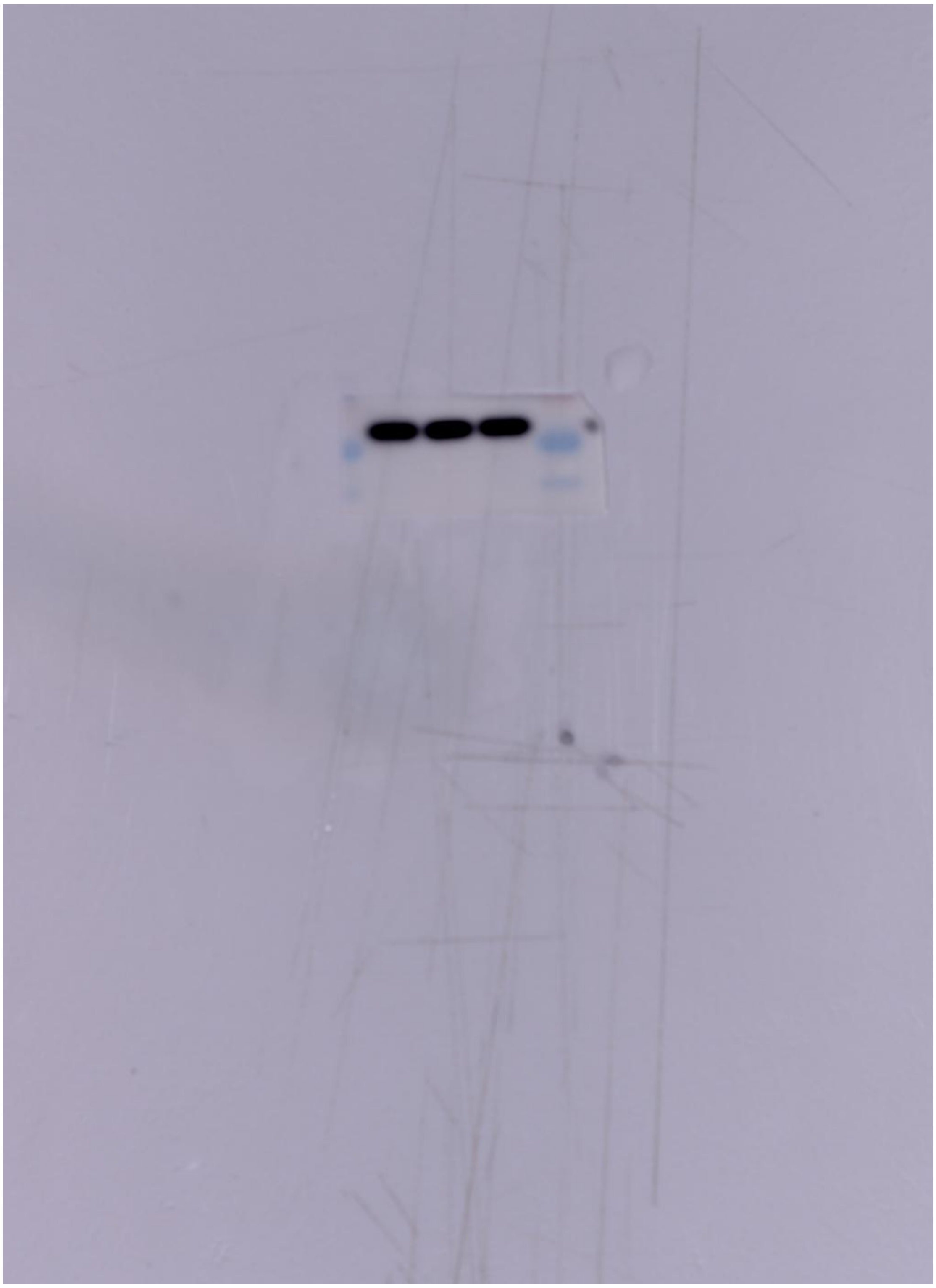

C

SK-N-DZ-NeuroD1

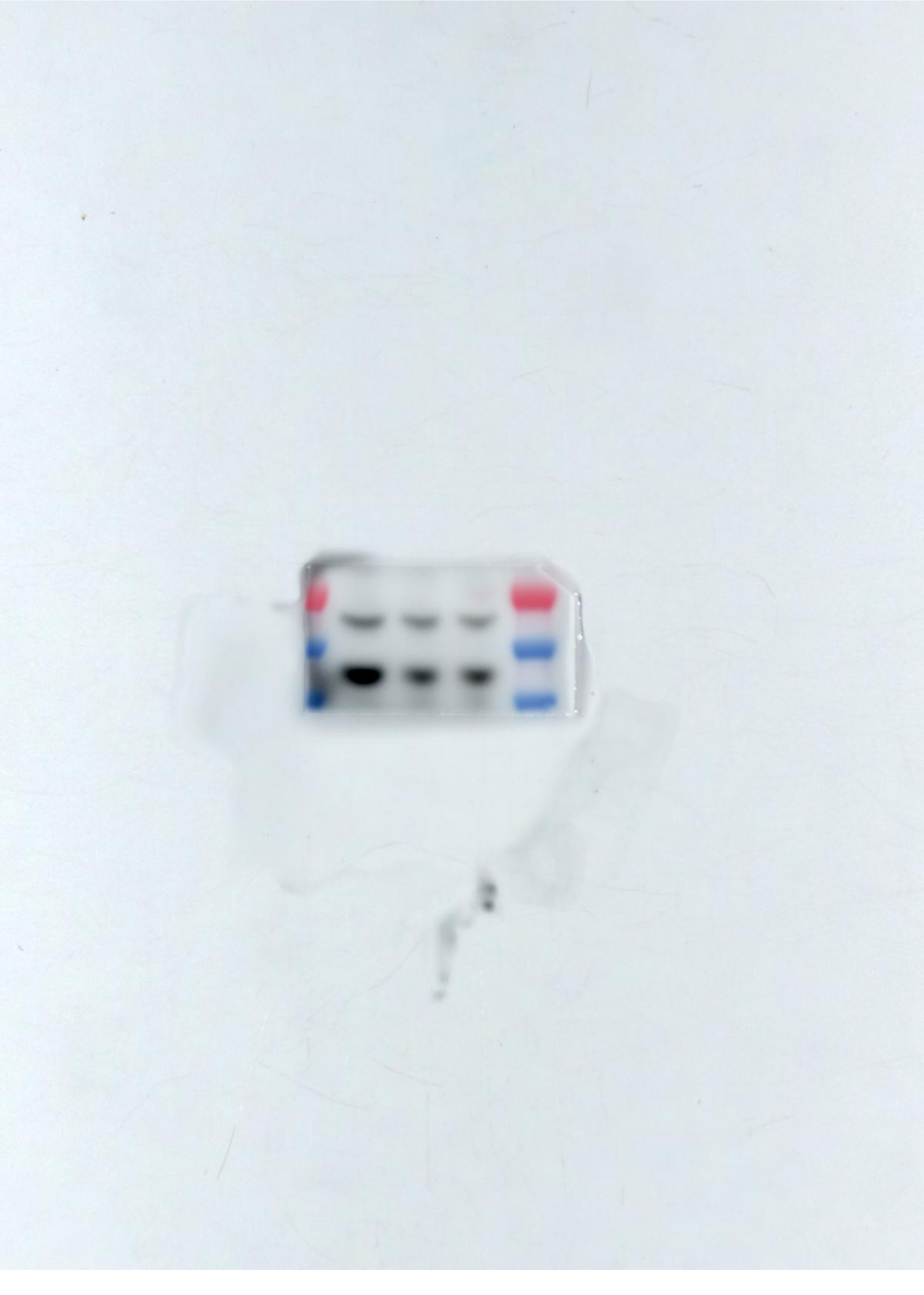

SK-N-DZ-N-Myc

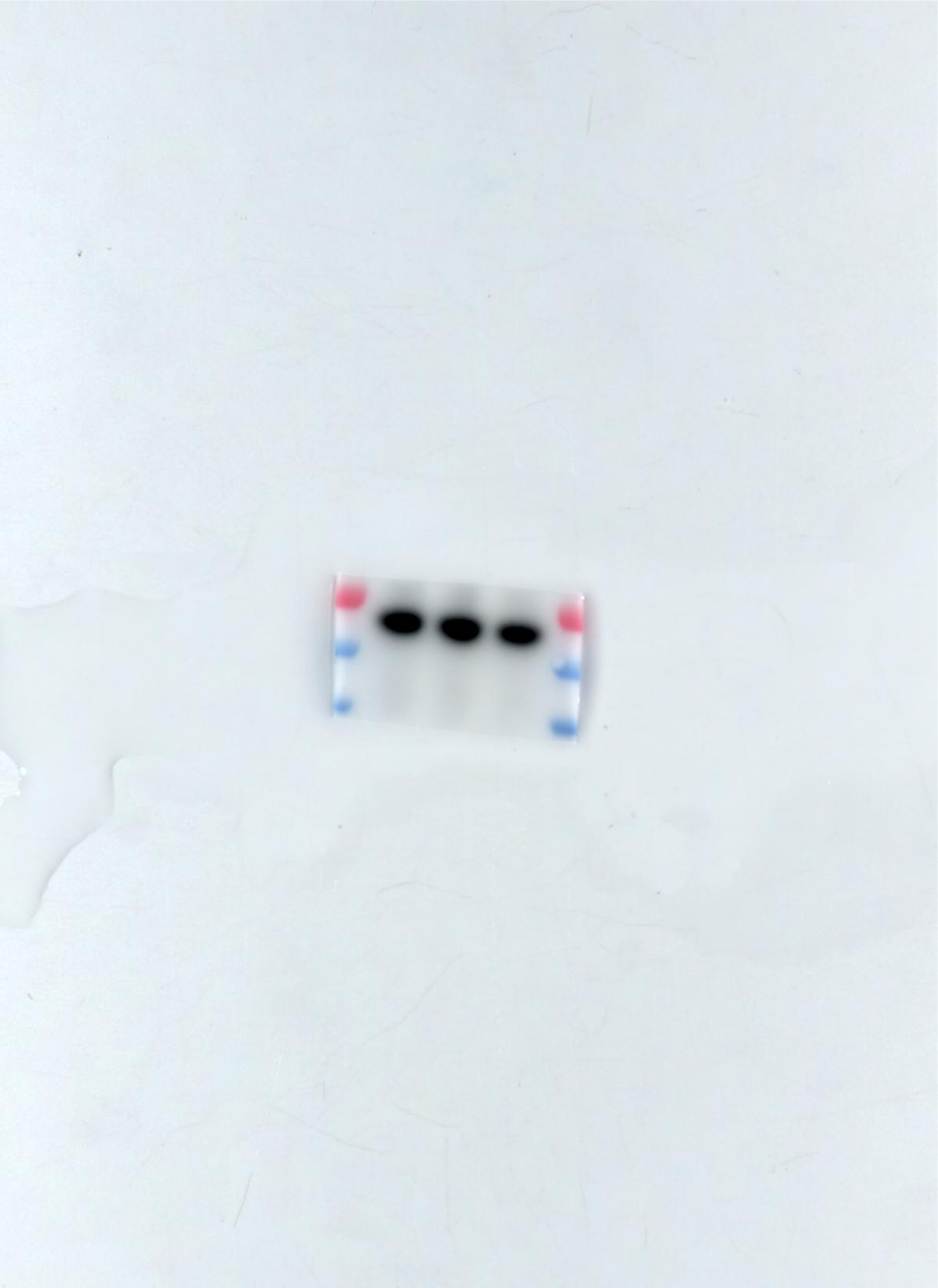

SK-N-DZ-GAPDH

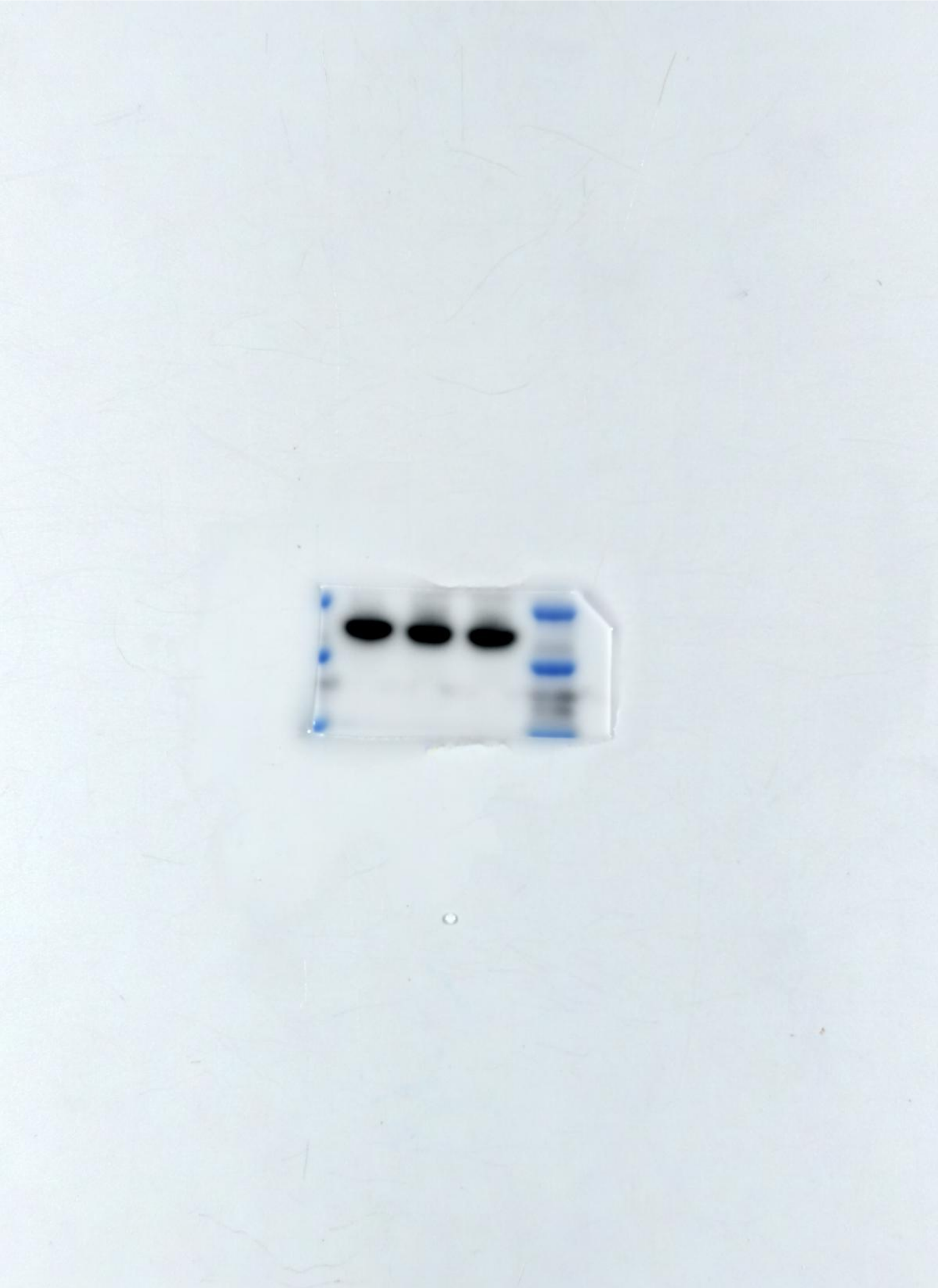

D

IP-Ub-K48

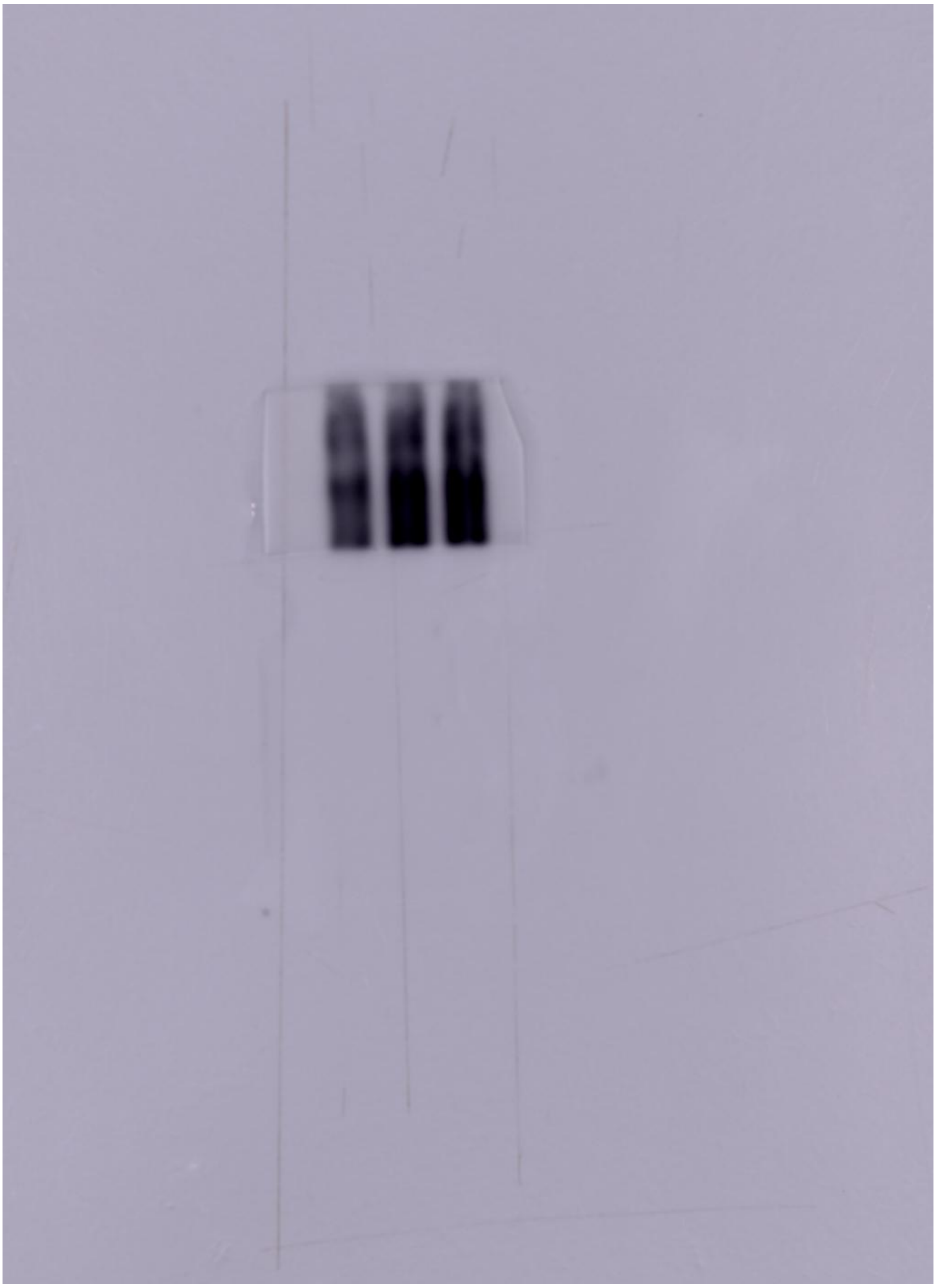

IP-N-Myc

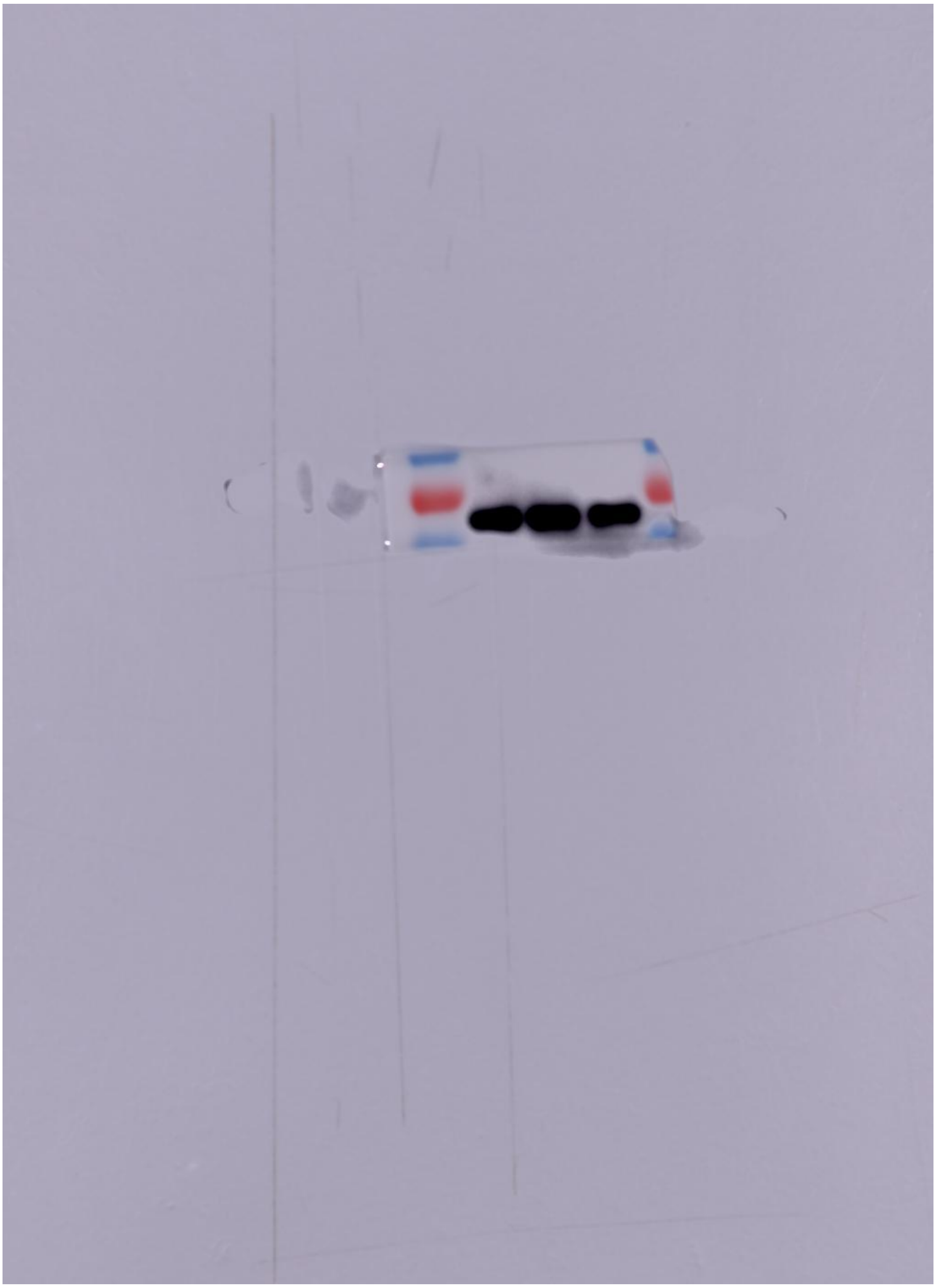

Input-Ub-K48

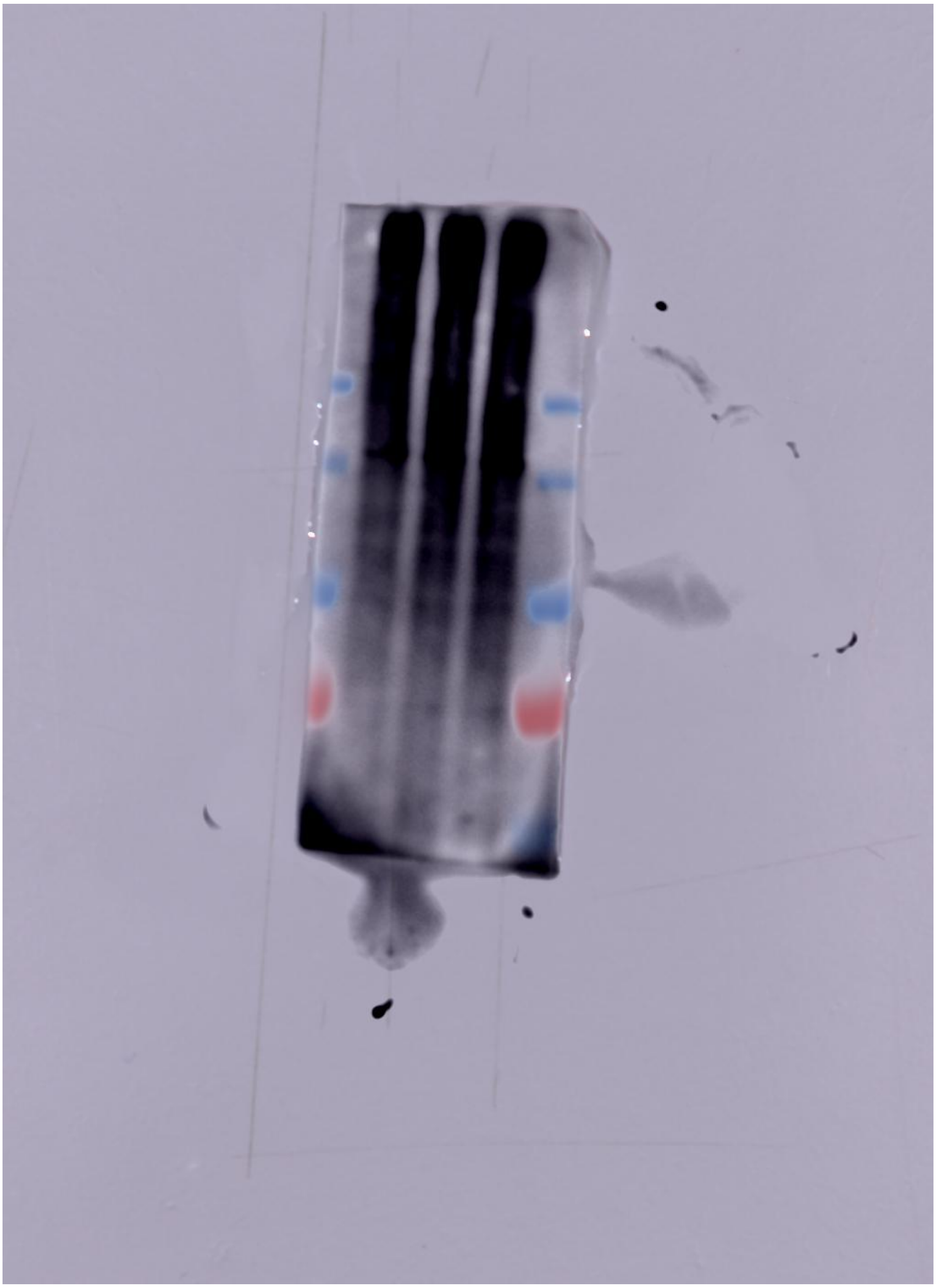

Input-NeuroD1

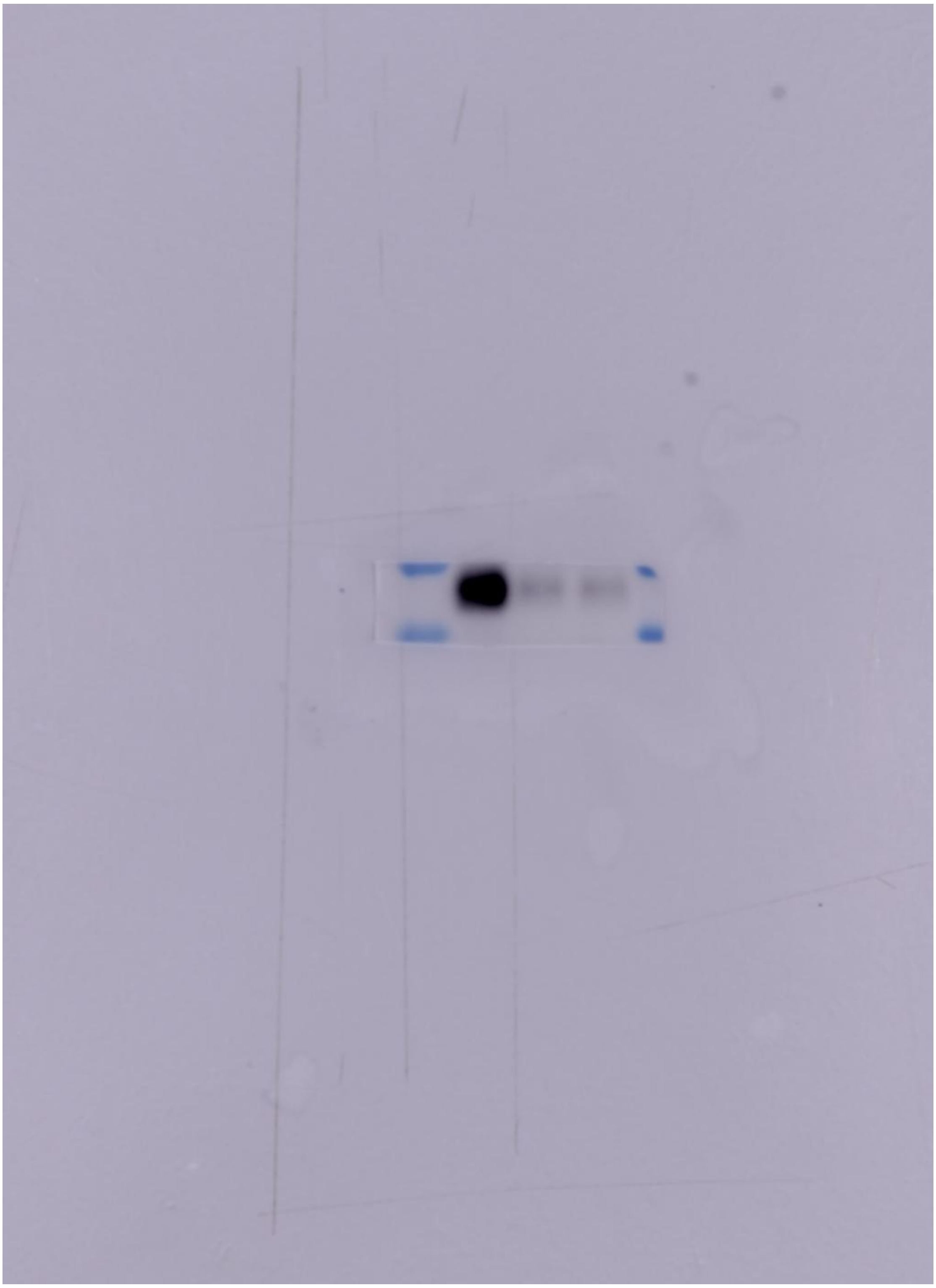

Input-N-Myc

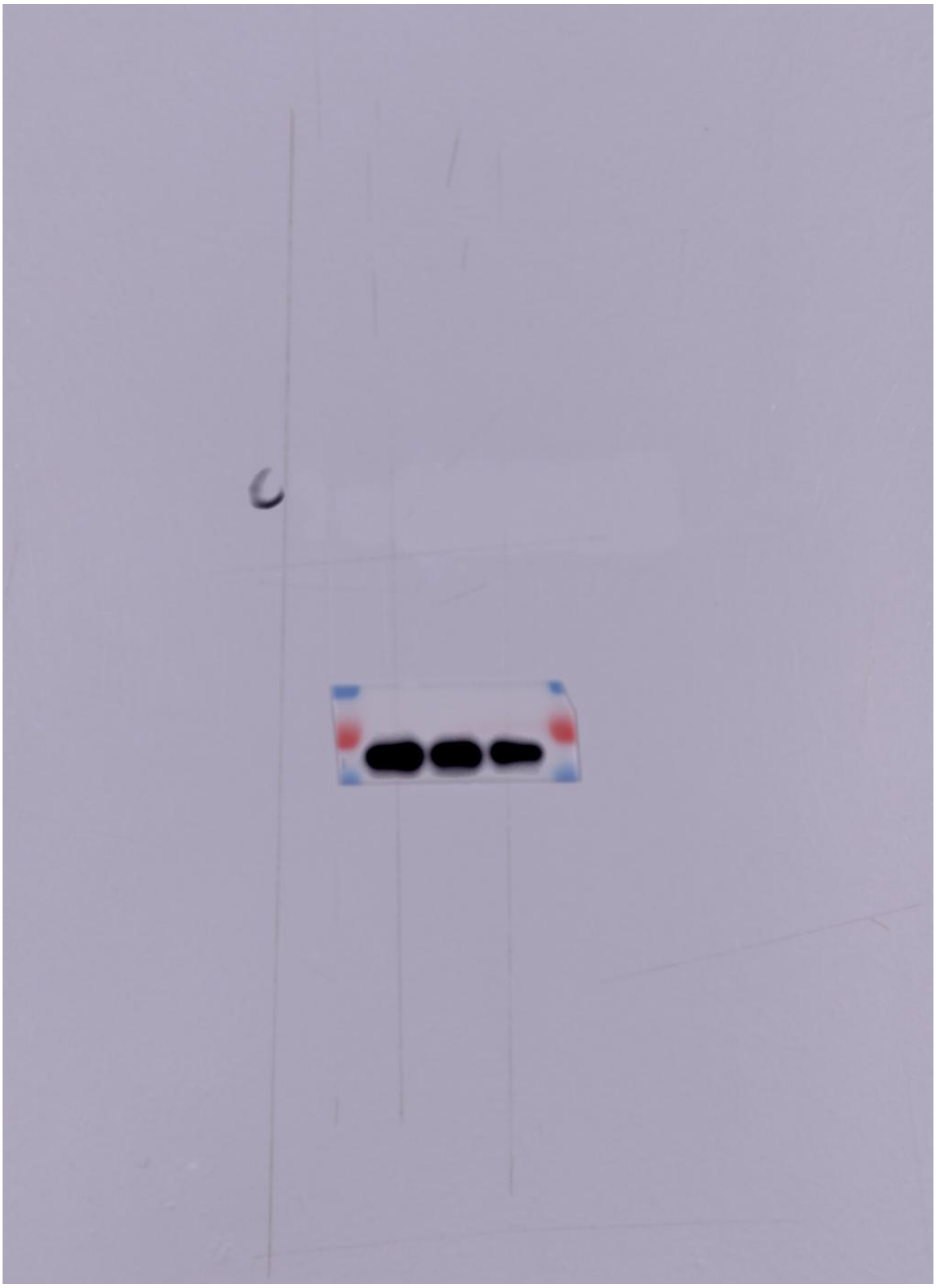

Input-GAPDH

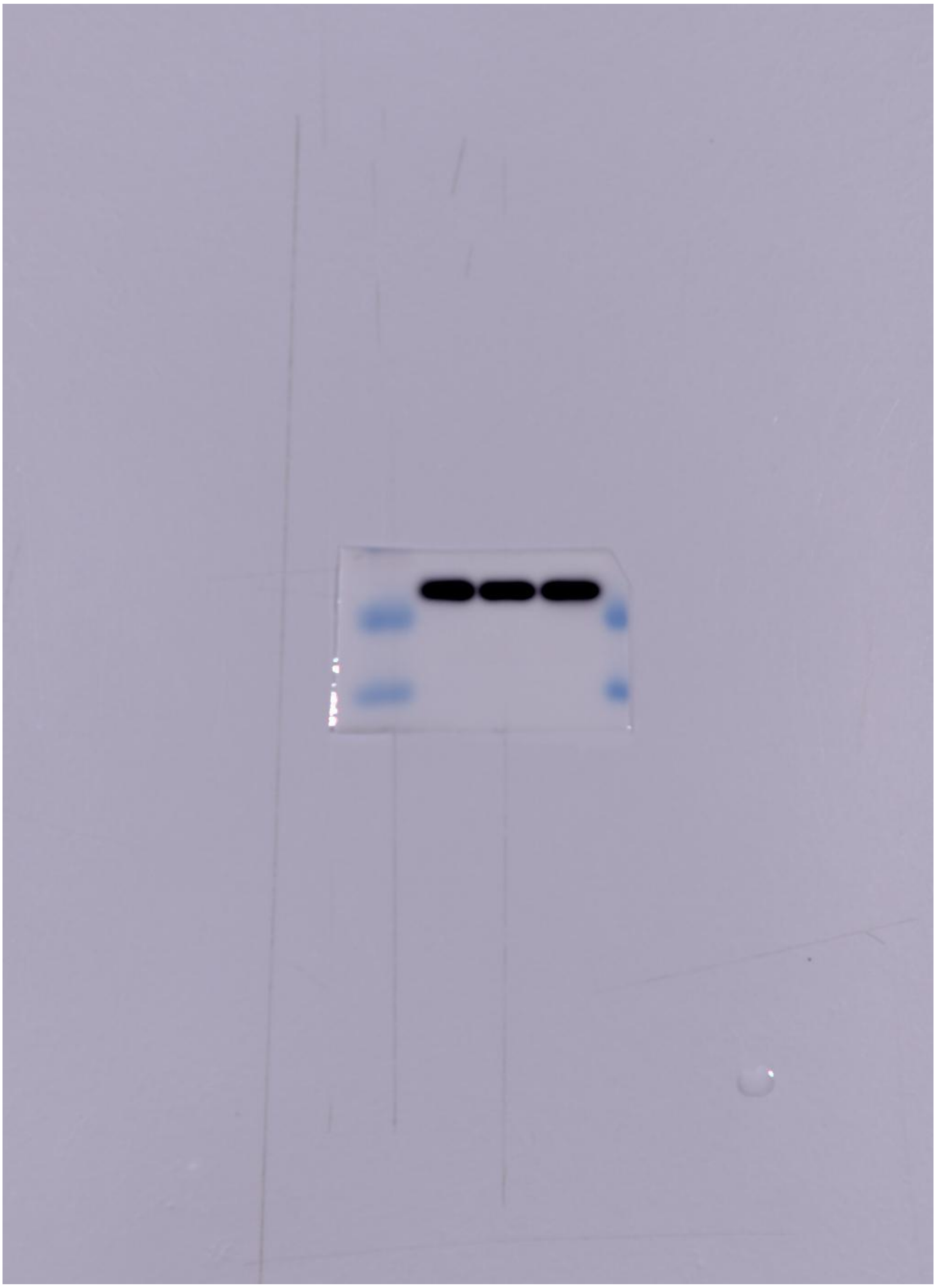

FIgure 5

A

IMR-32-NeuroD1

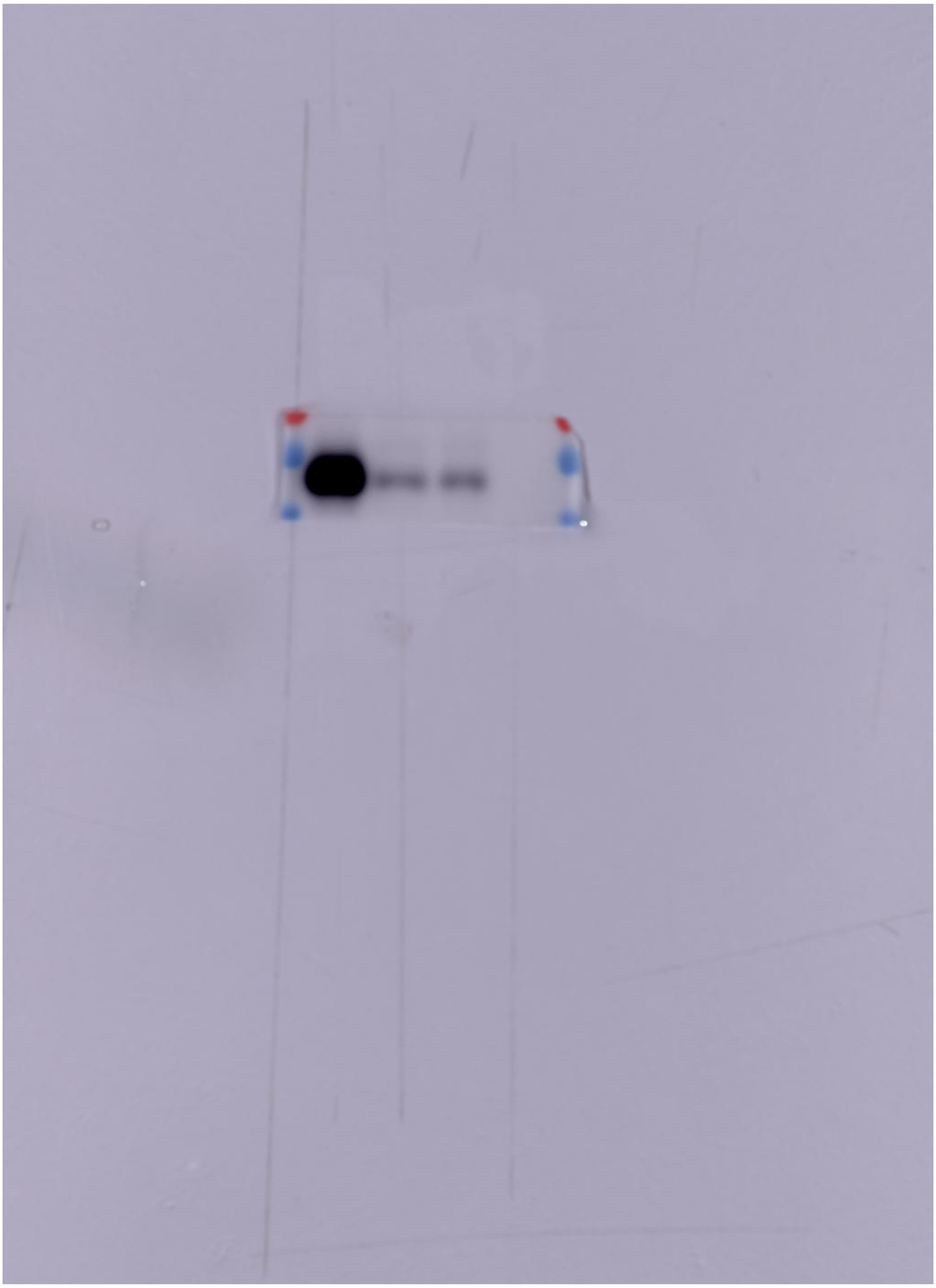

IMR-32-USP1

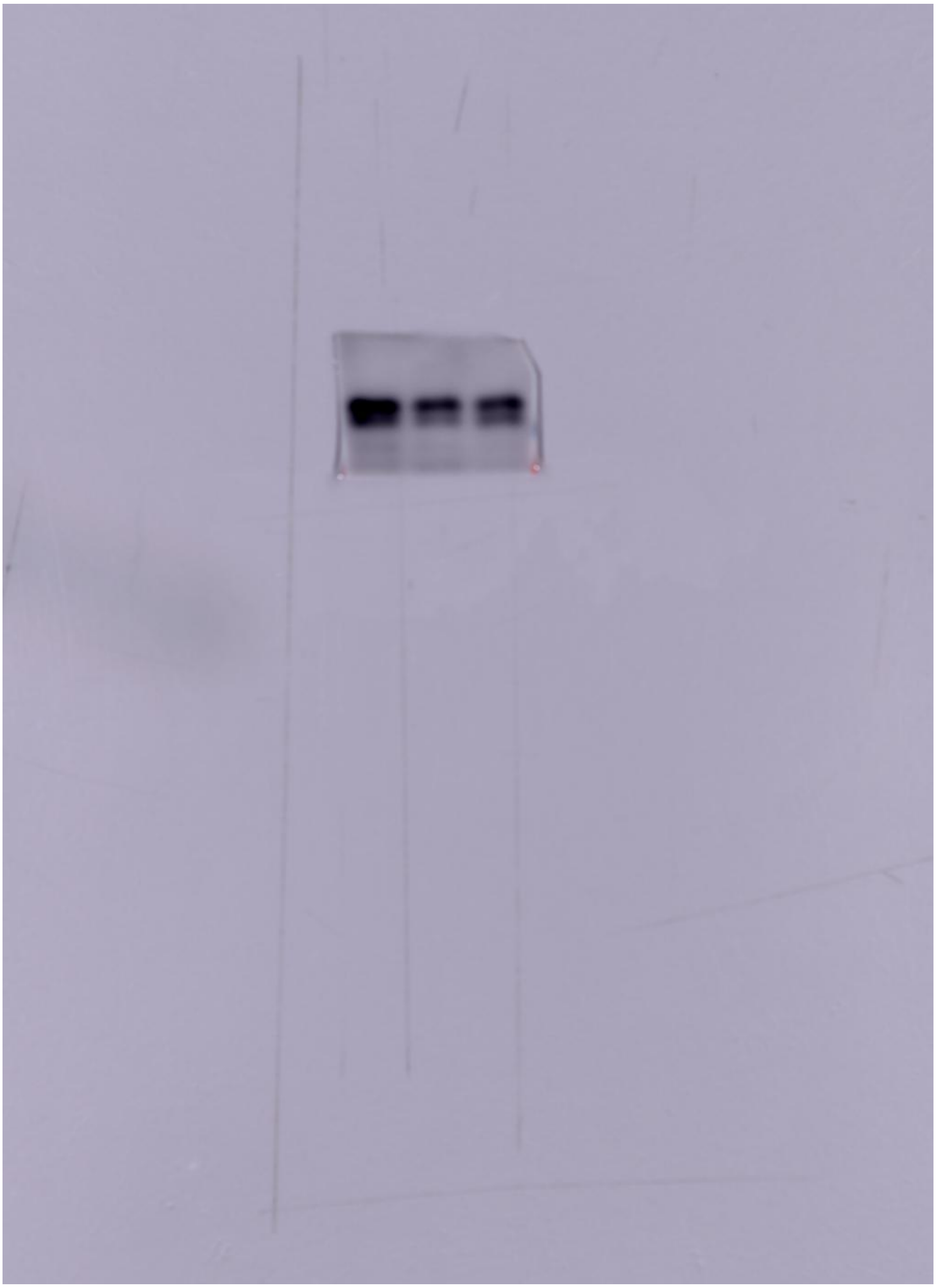

IMR-32-GAPDH

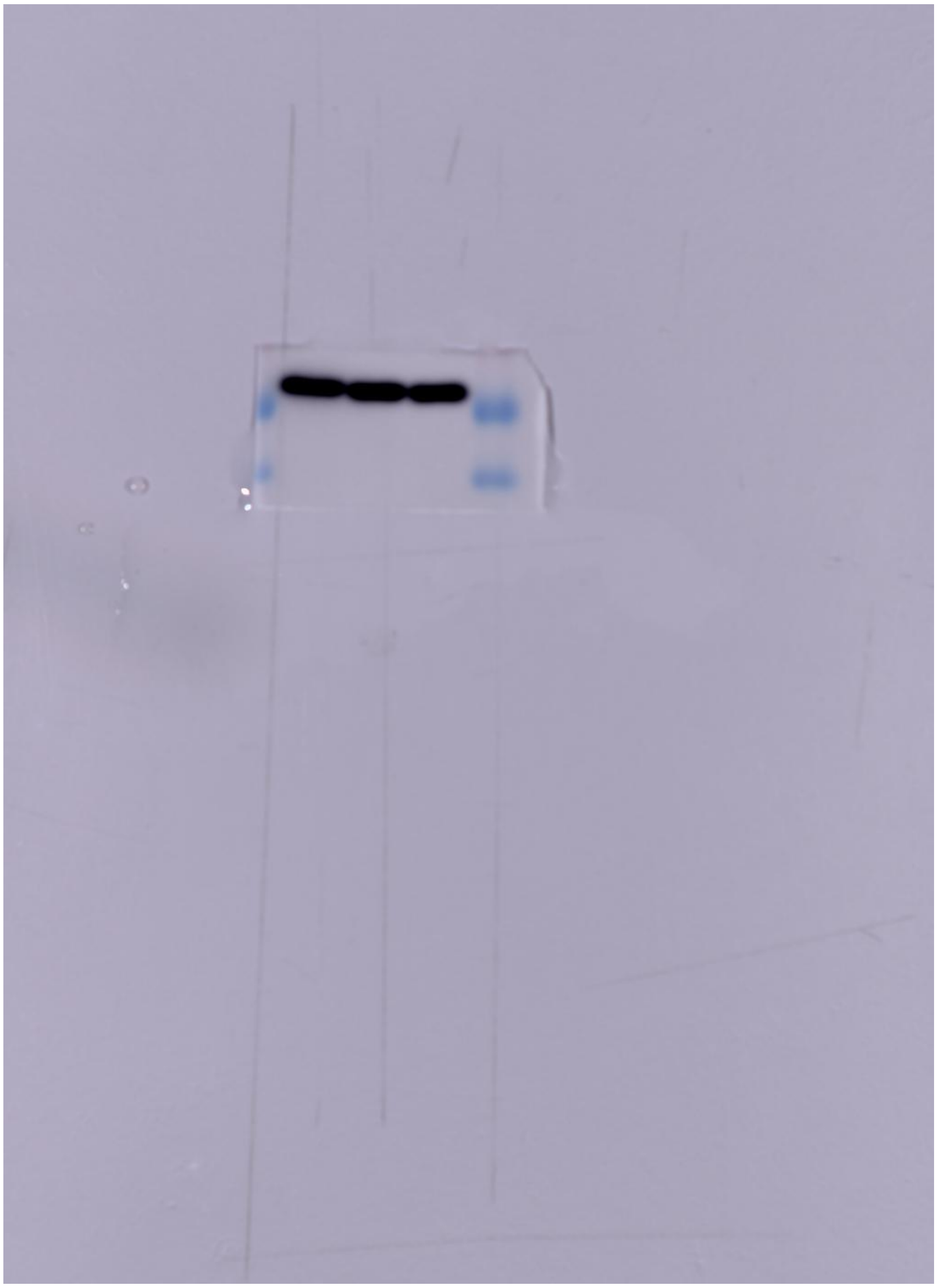

B

BE(2)-M17-NeuroD1

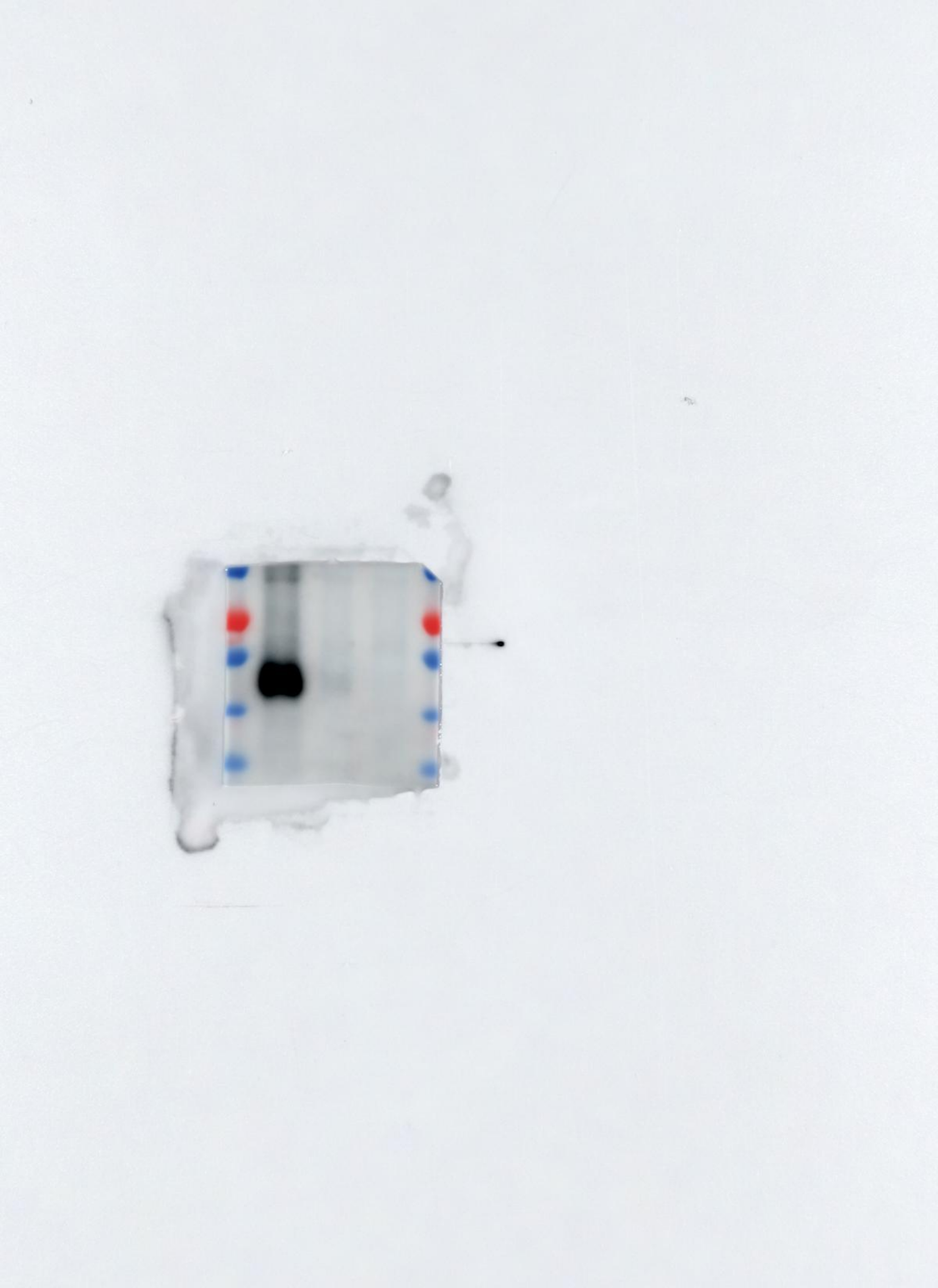

BE(2)-M17-USP1

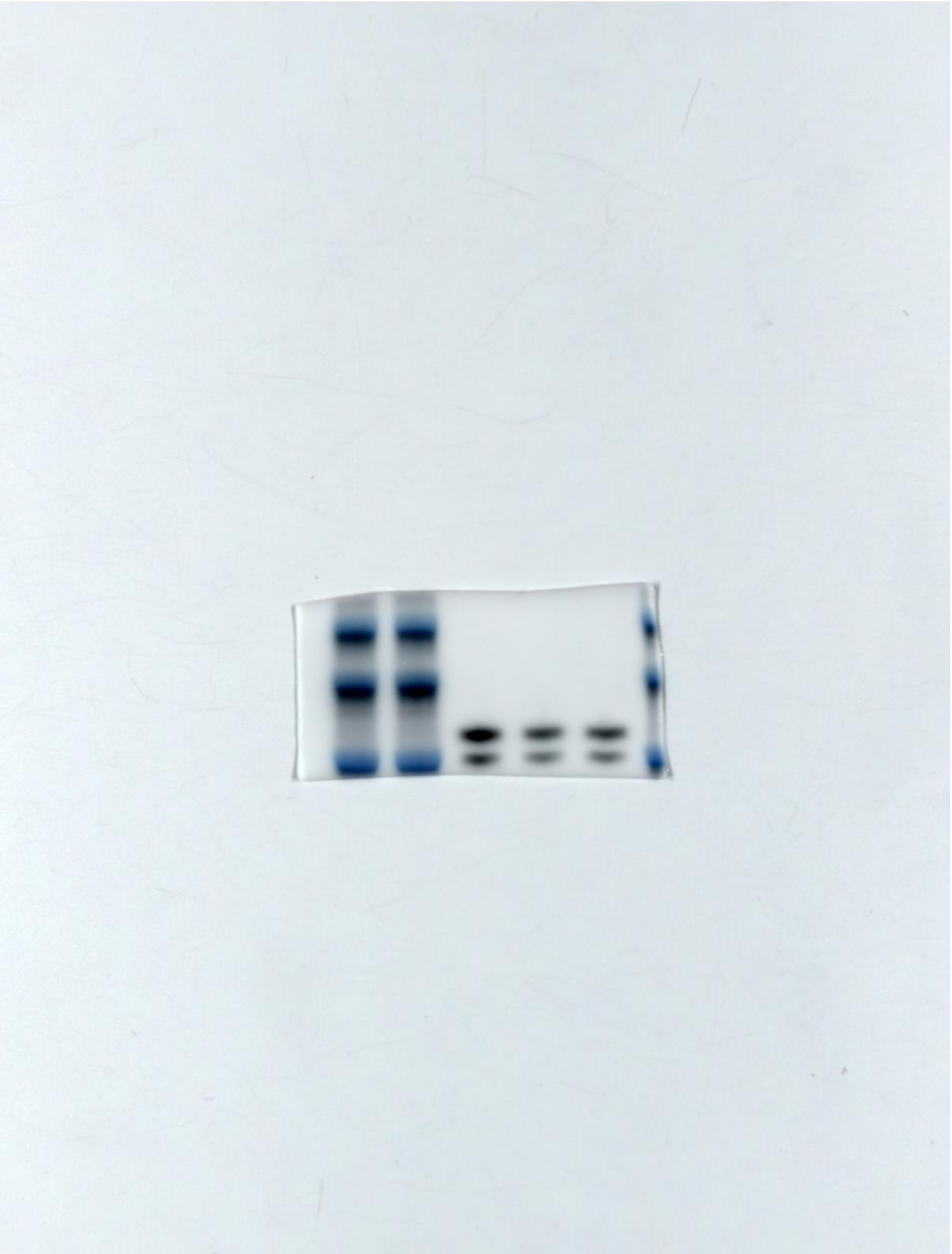

BE(2)-M17-GAPDH

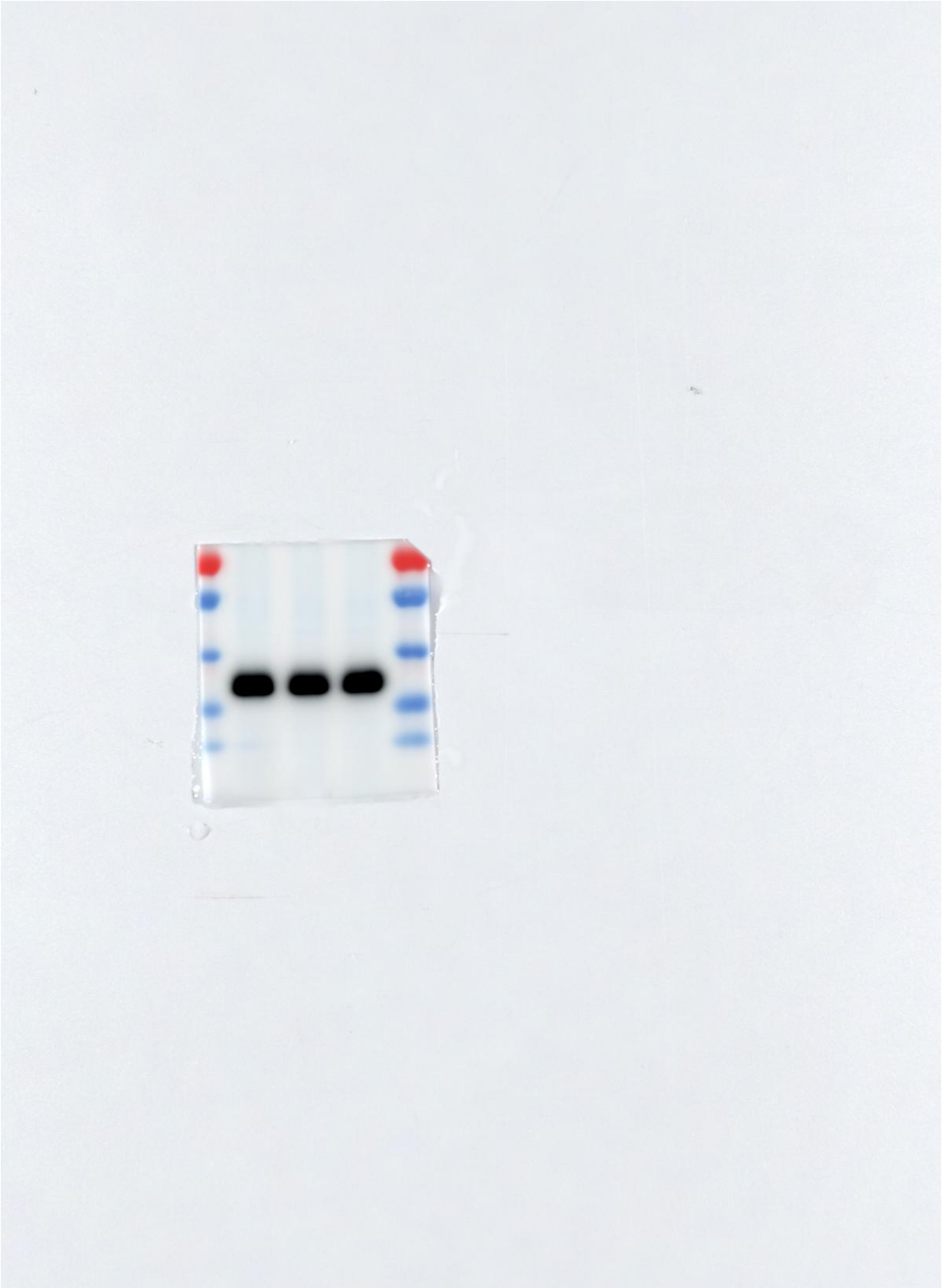

C

SK-N-DZ-NeuroD1

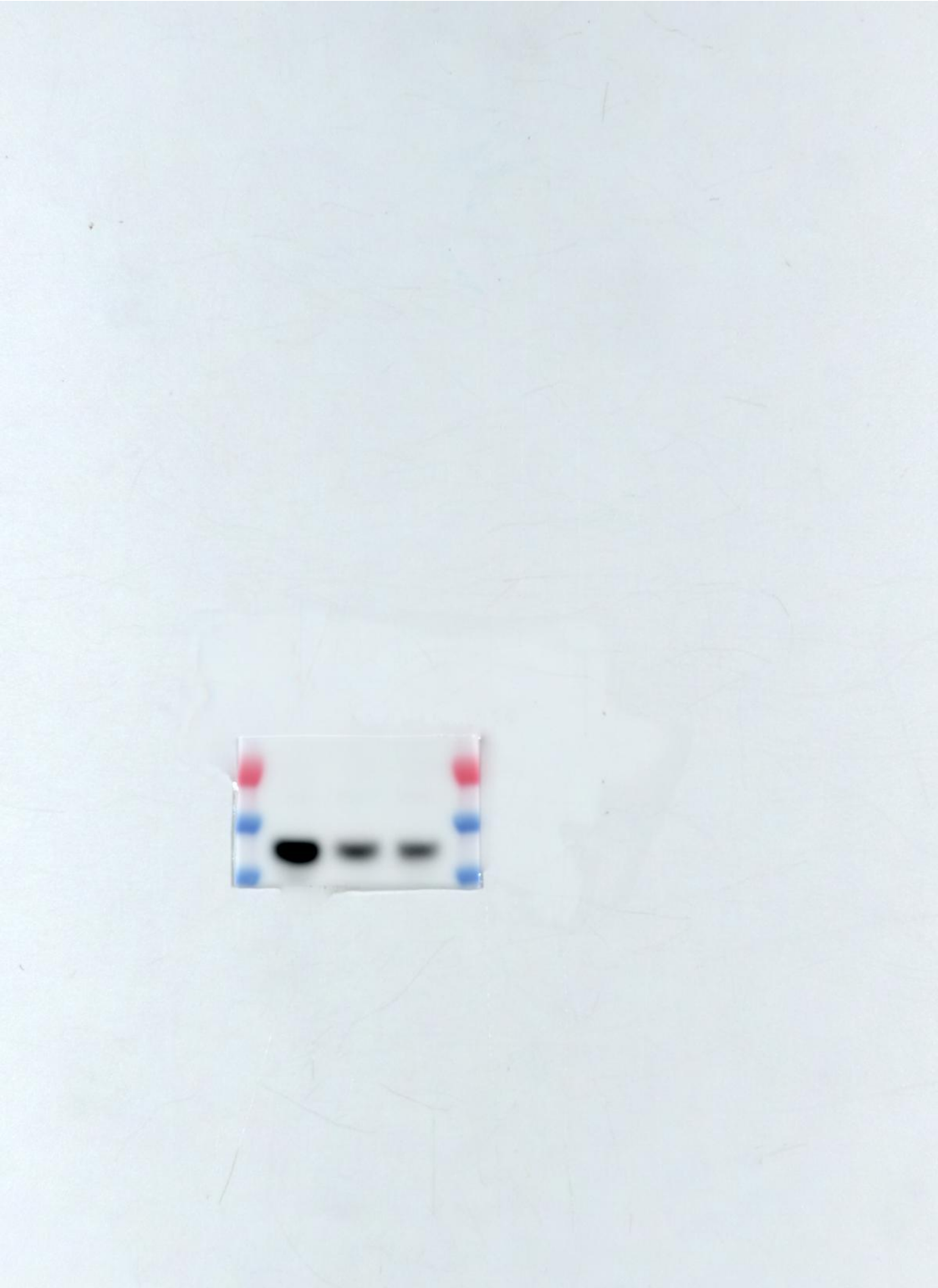

SK-N-DZ-USP1

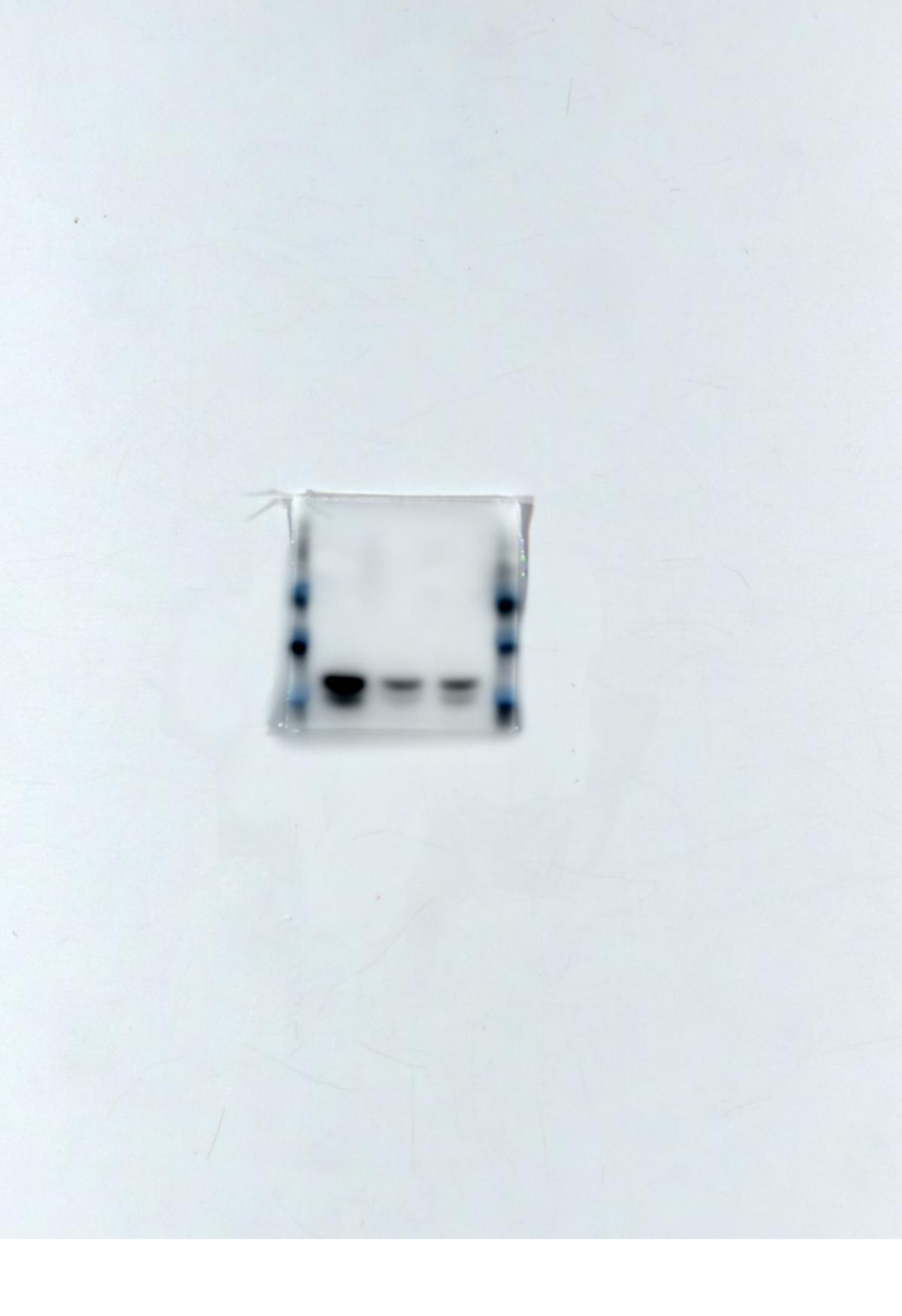

SK-N-DZ-GAPDH

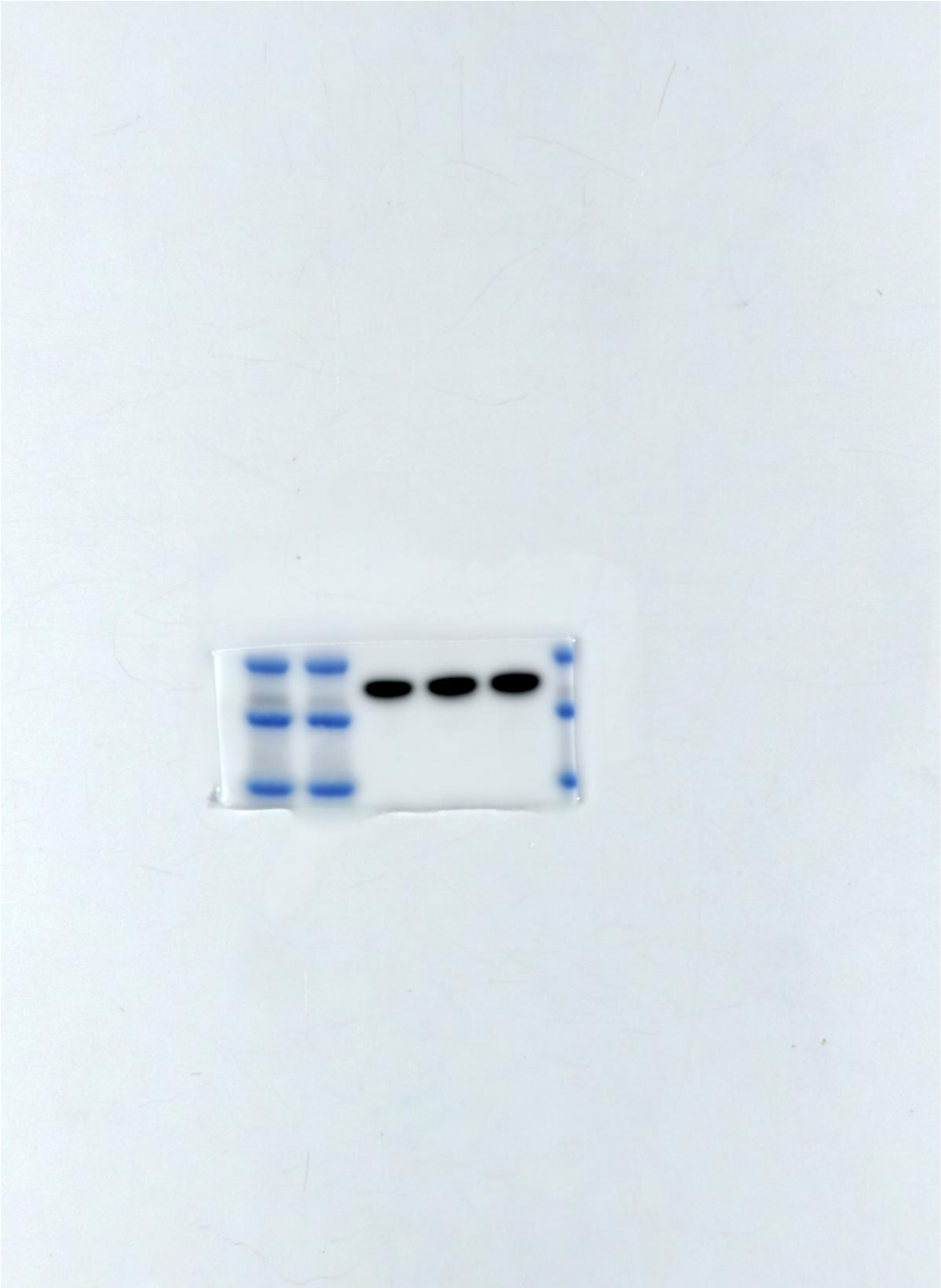

FIgure 6

E

IMR-32-USP1

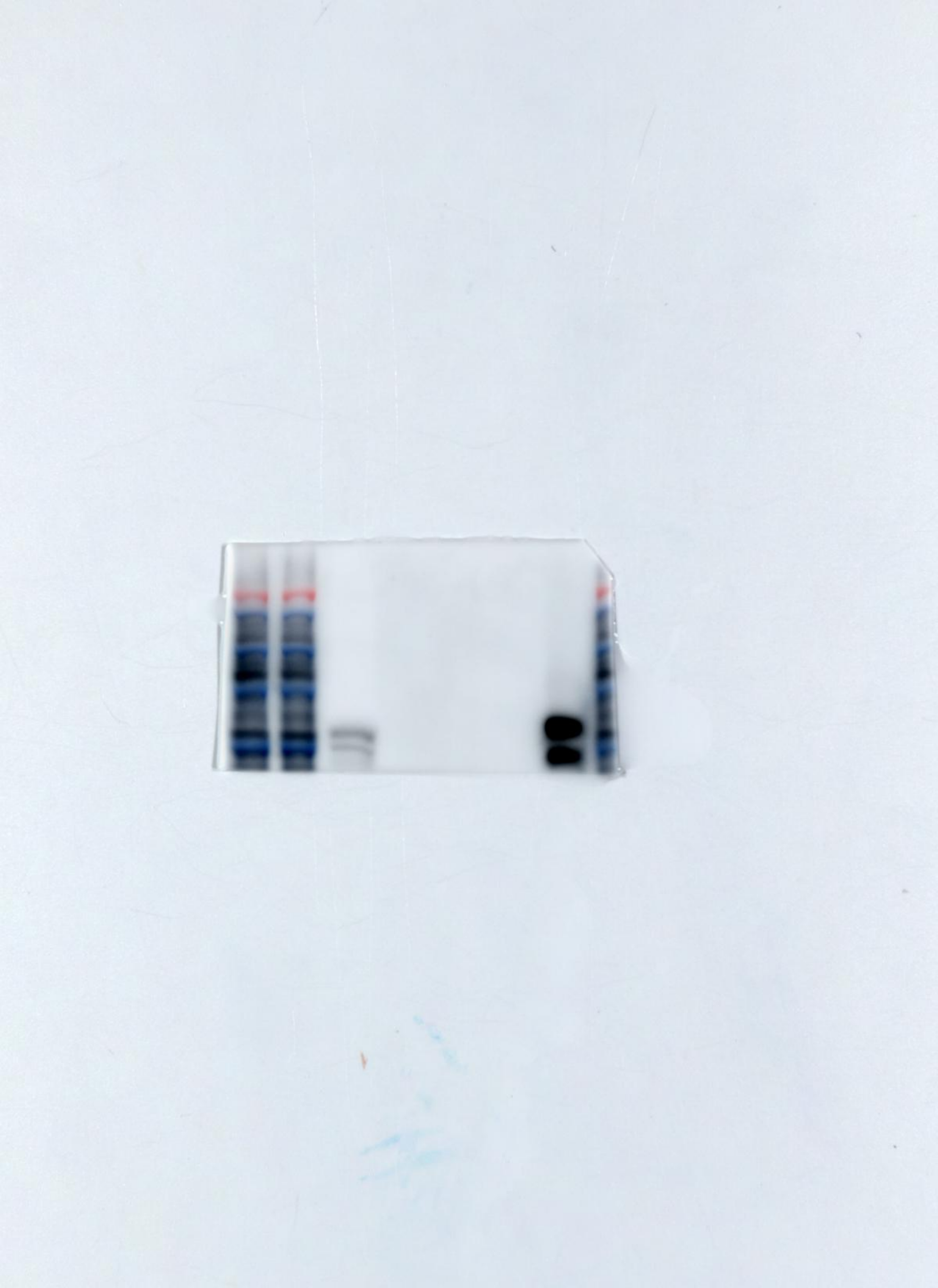

IMR-32-N-Myc

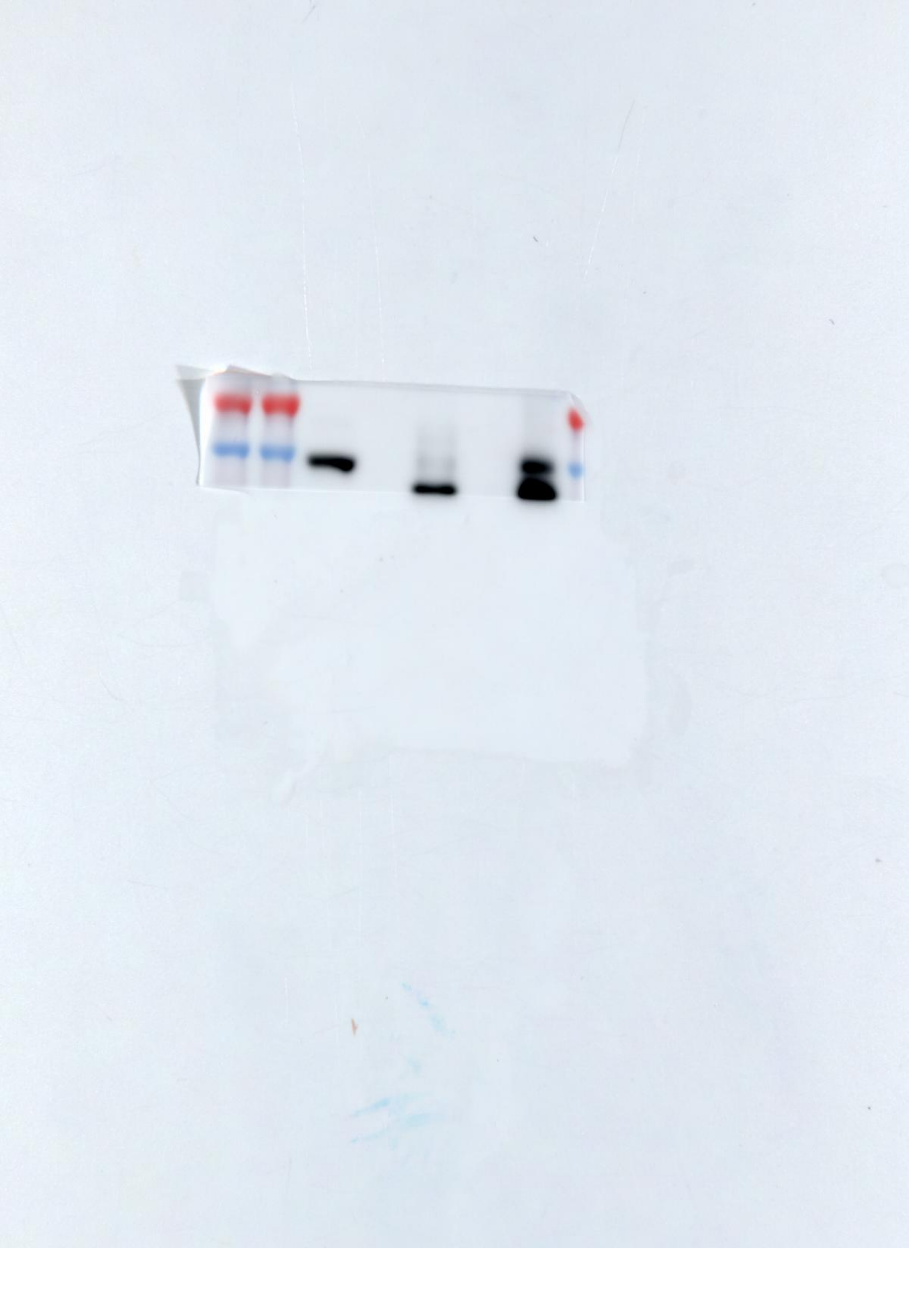

IMR-32-GAPDH

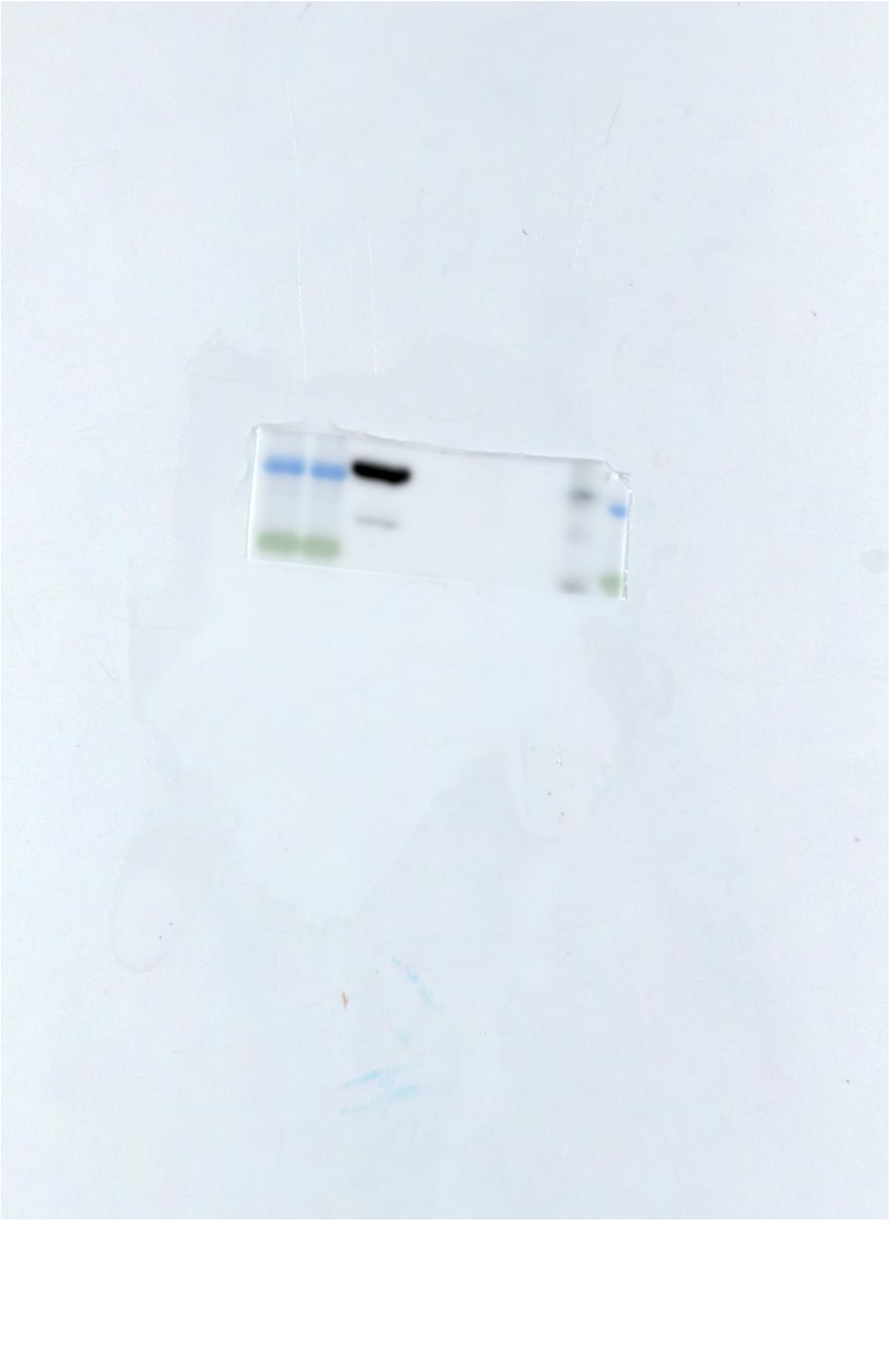

BE(2)-M17-USP1

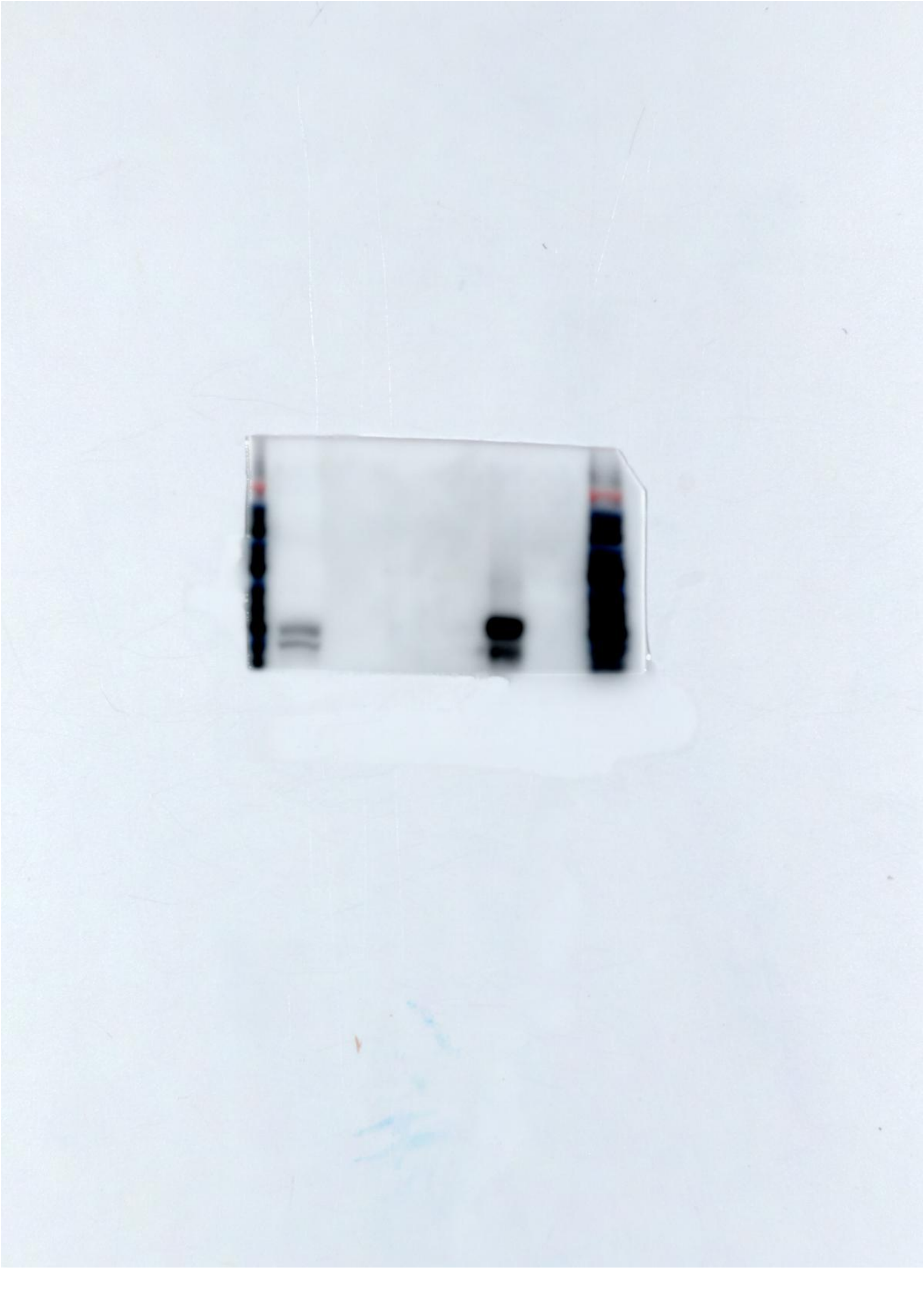

BE(2)-M17-N-Myc

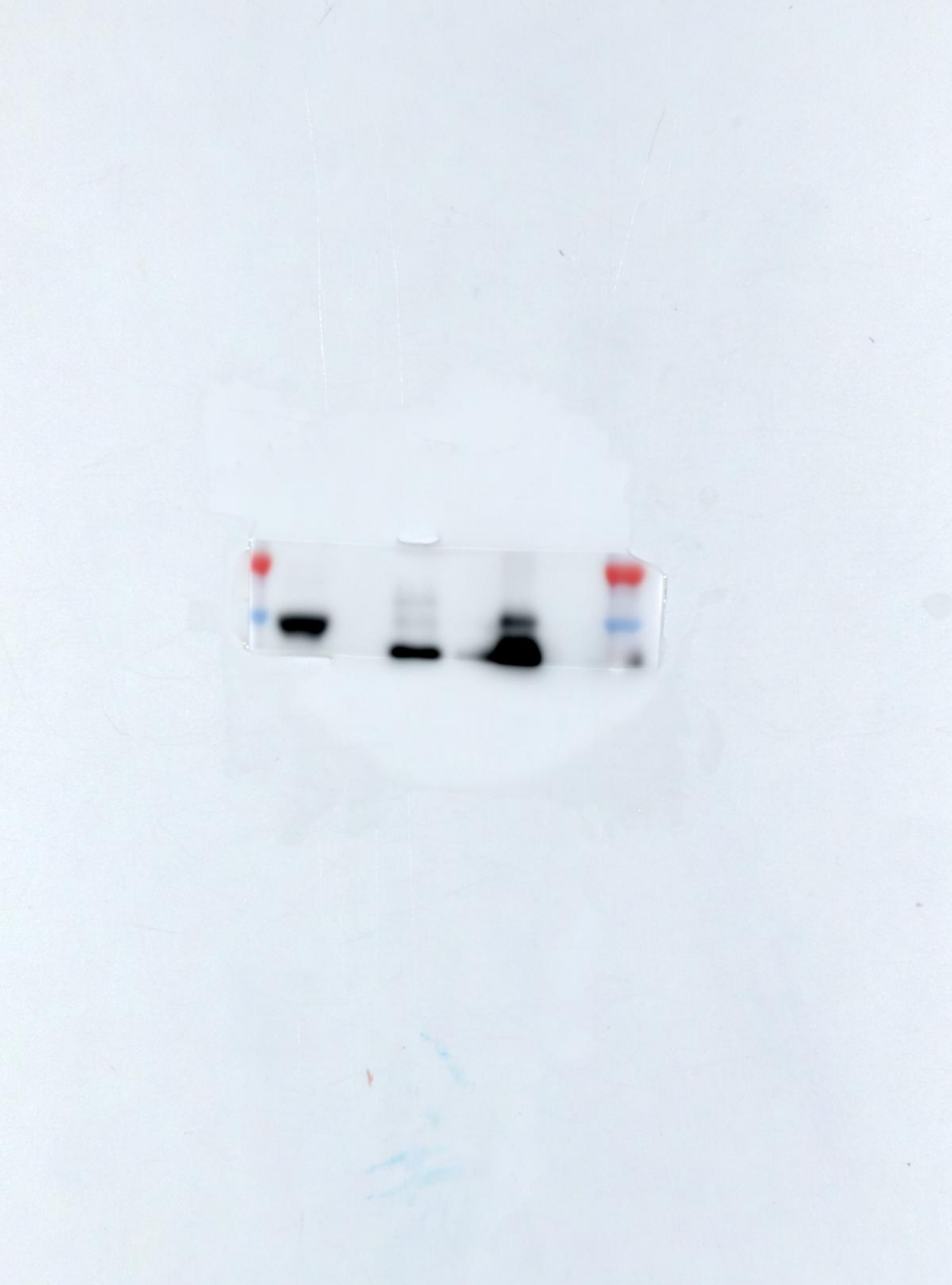

BE(2)-M17-GAPDH

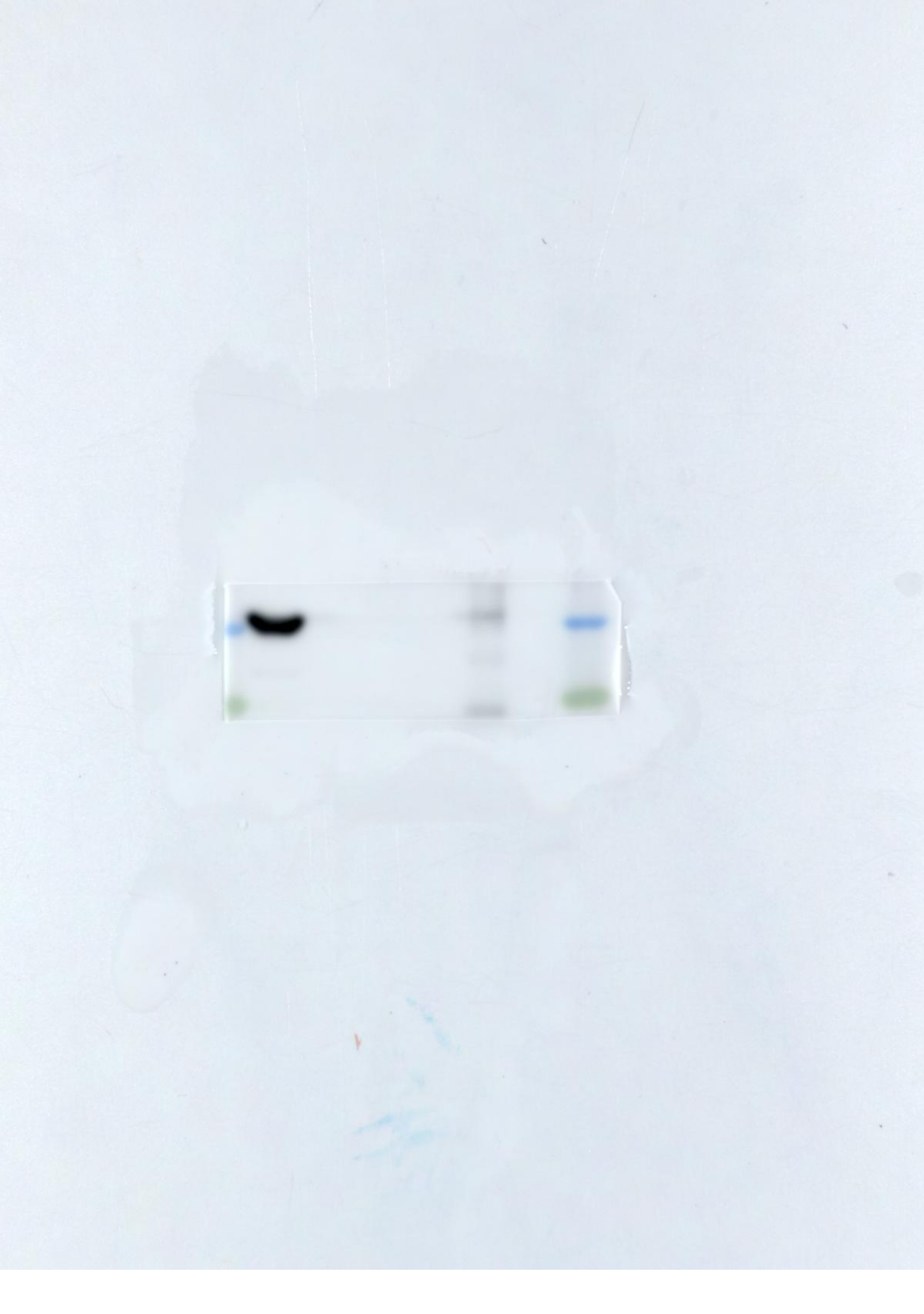

F

IMR-32-USP1

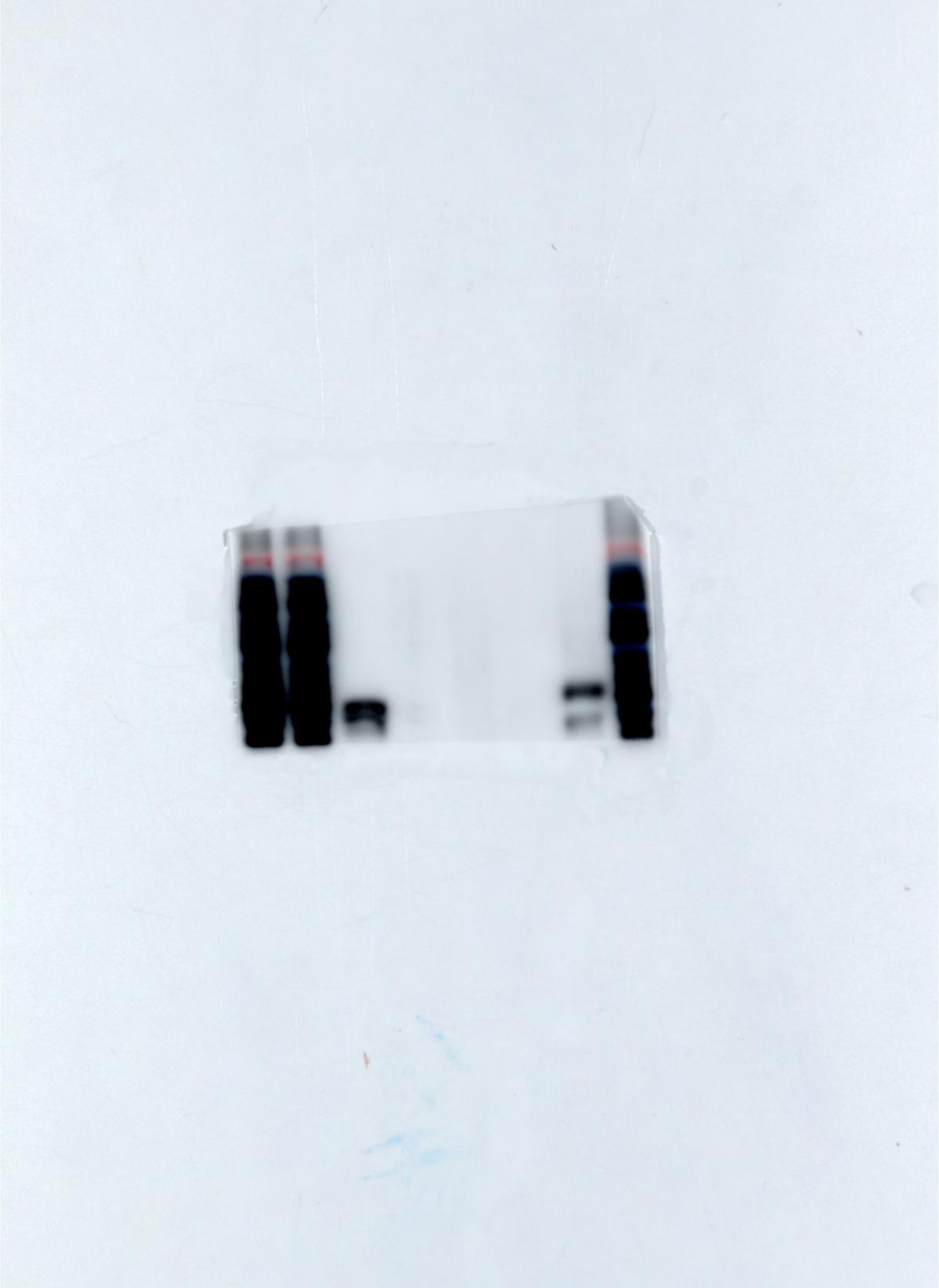

IMR-32-N-Myc

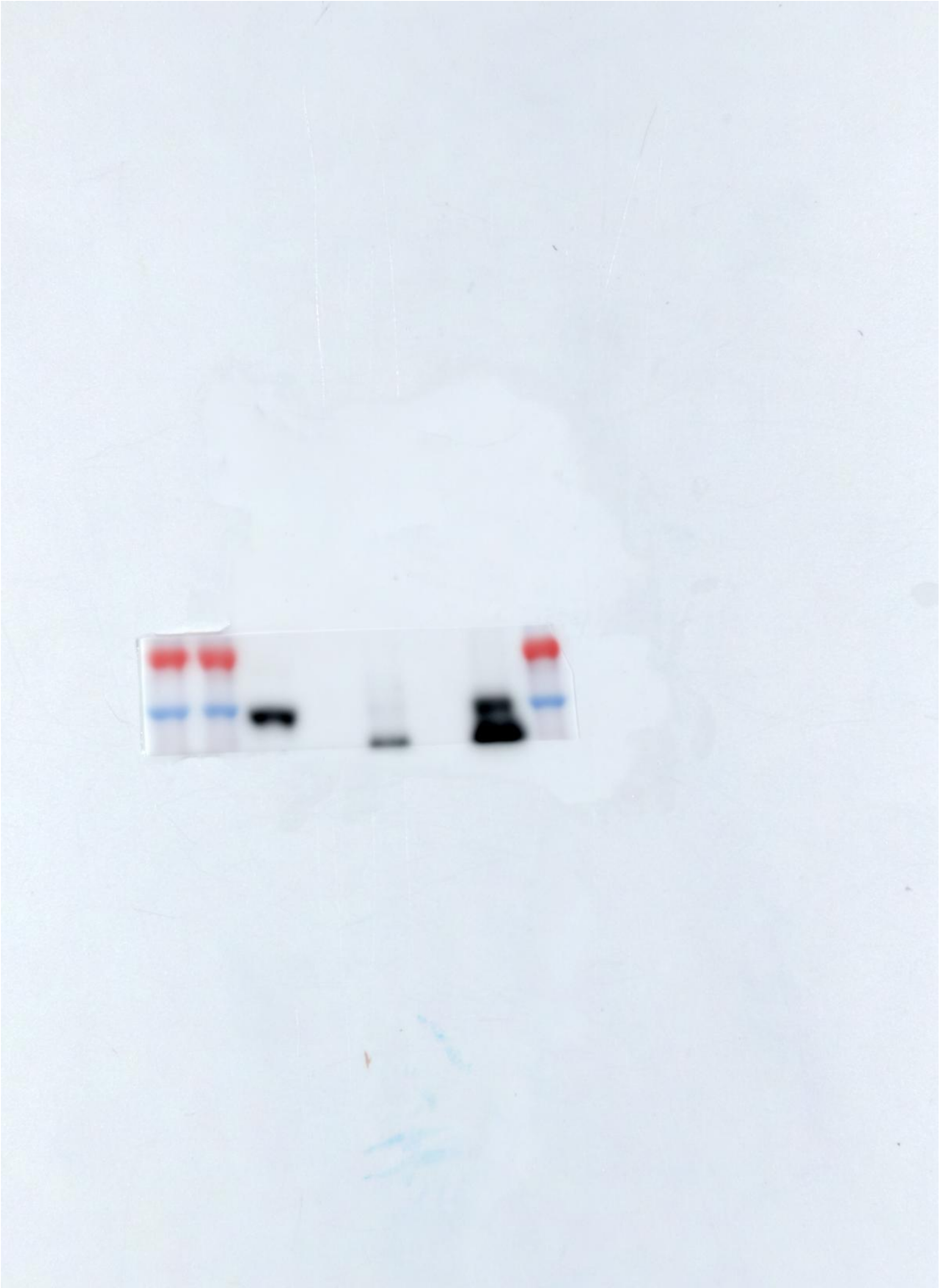

IMR-32-GAPDH

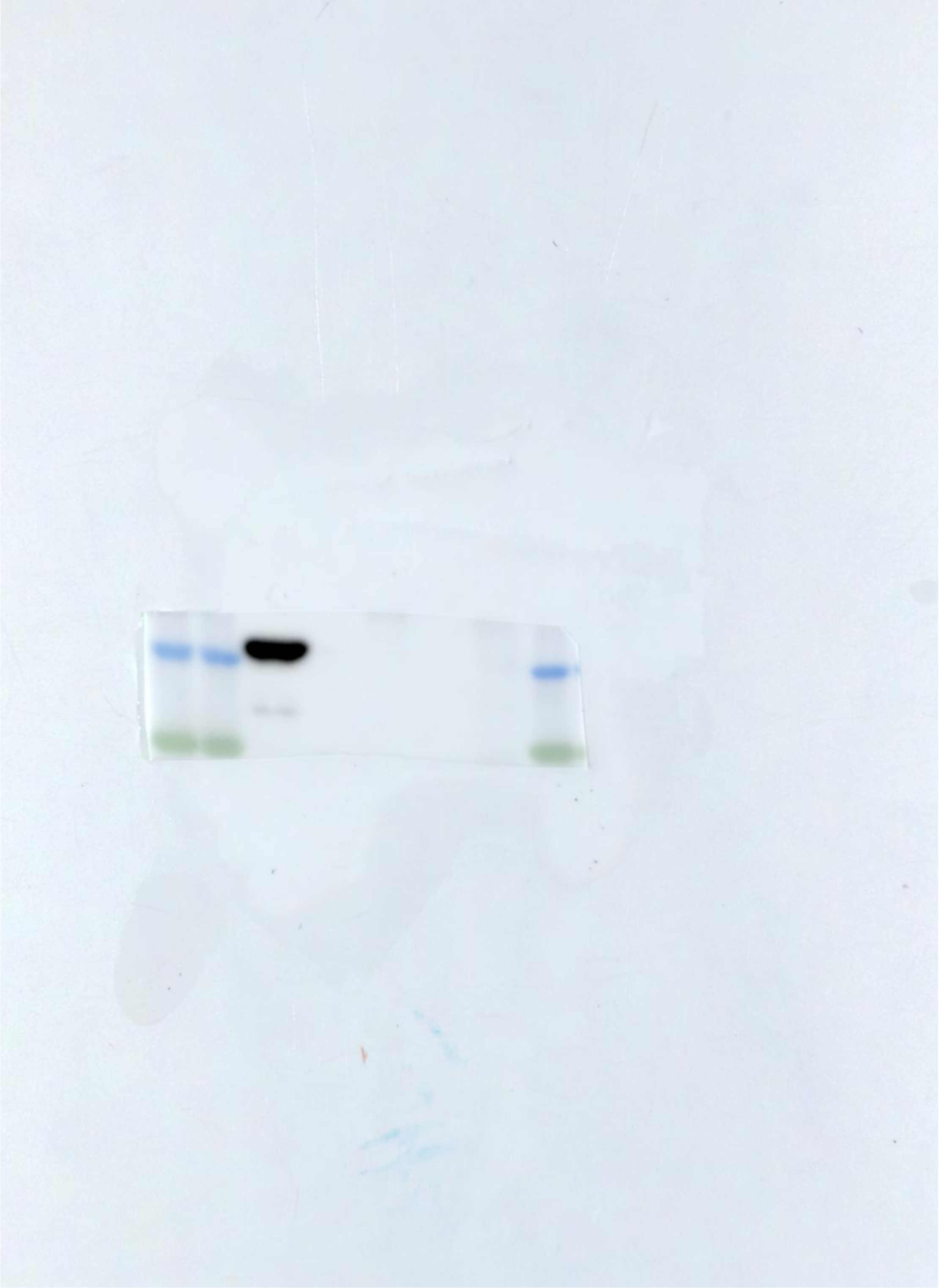

BE(2)-M17-USP1

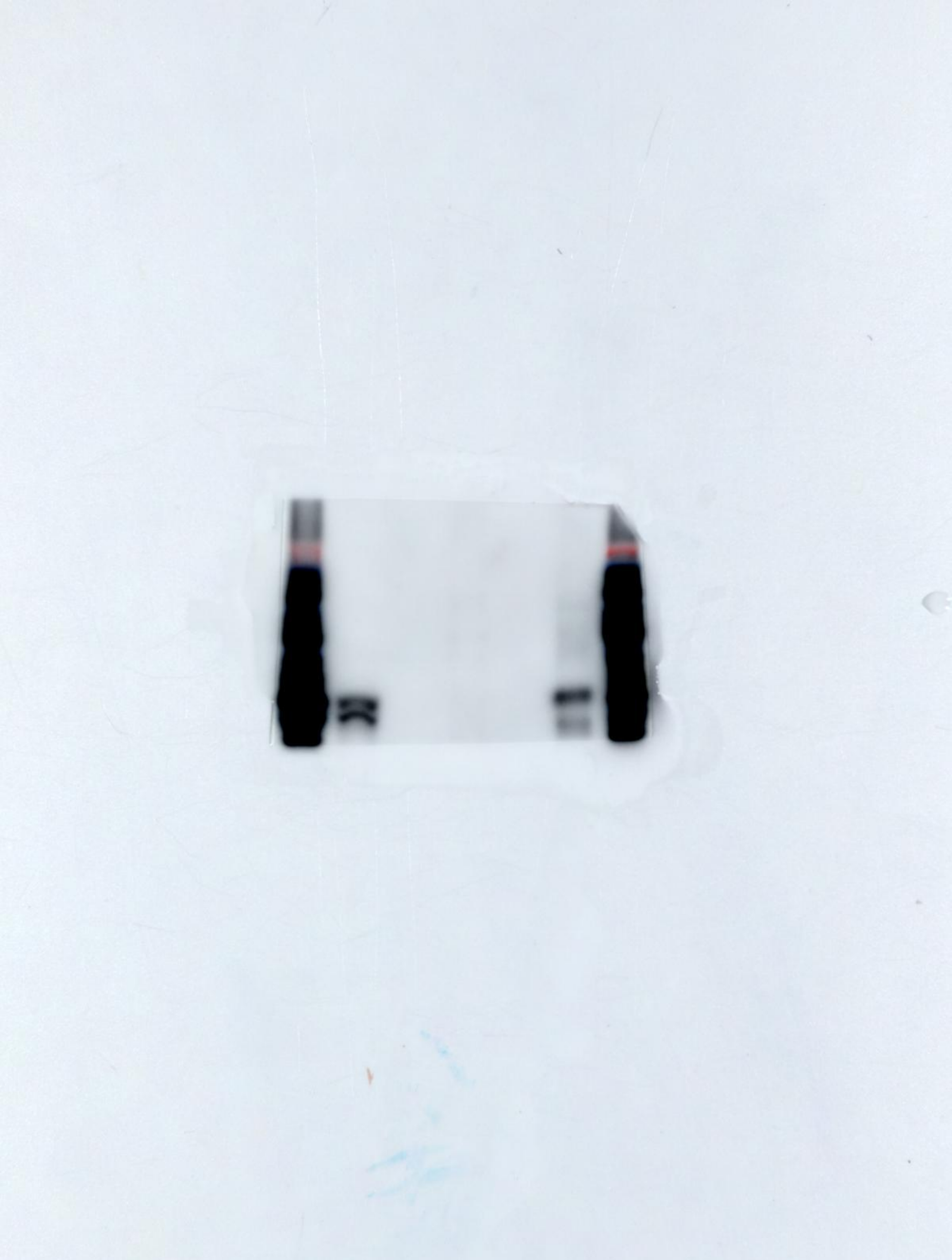

BE(2)-M17-N-Myc

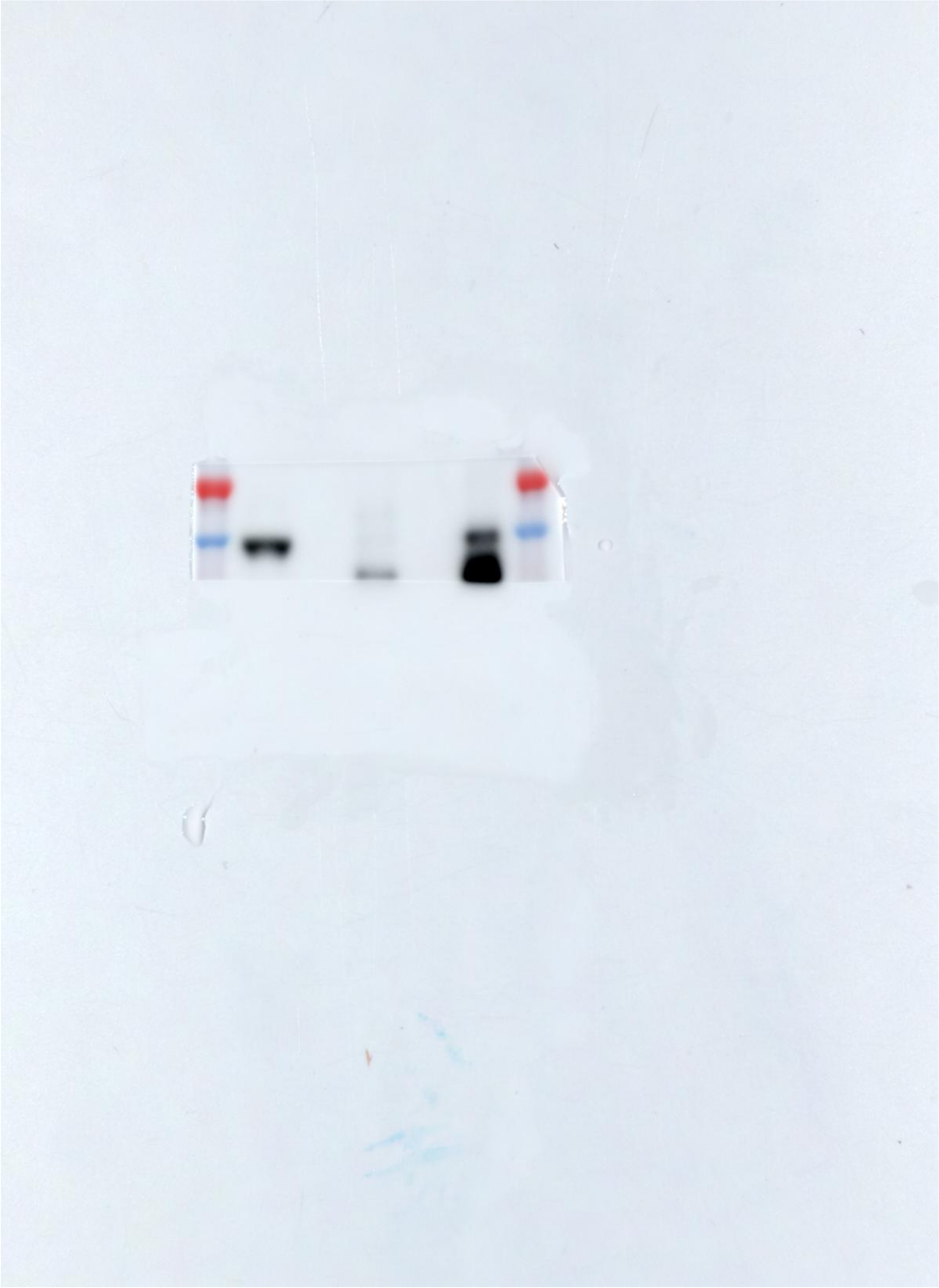

BE(2)-M17-GAPDH

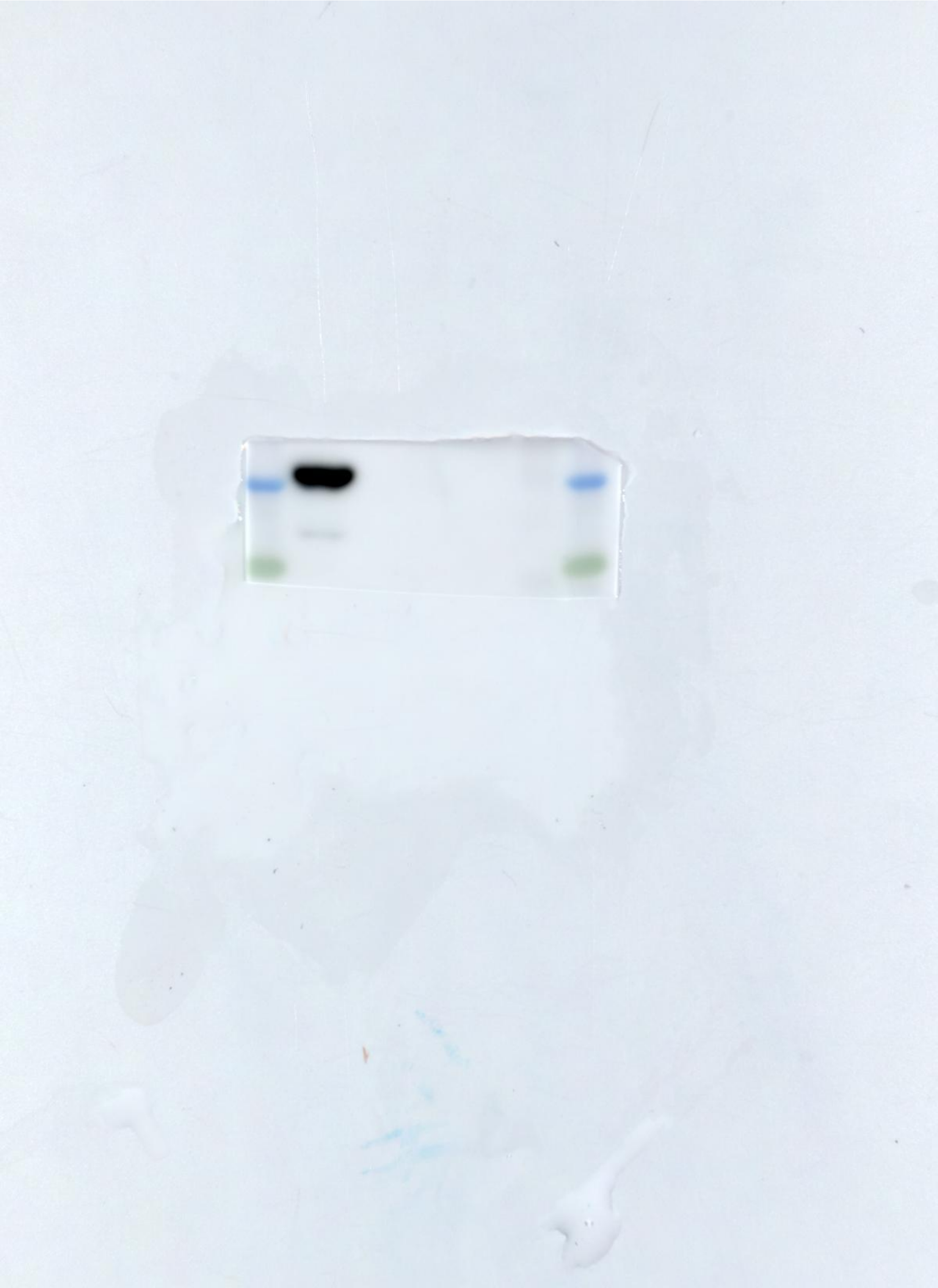

H

IP-GFP

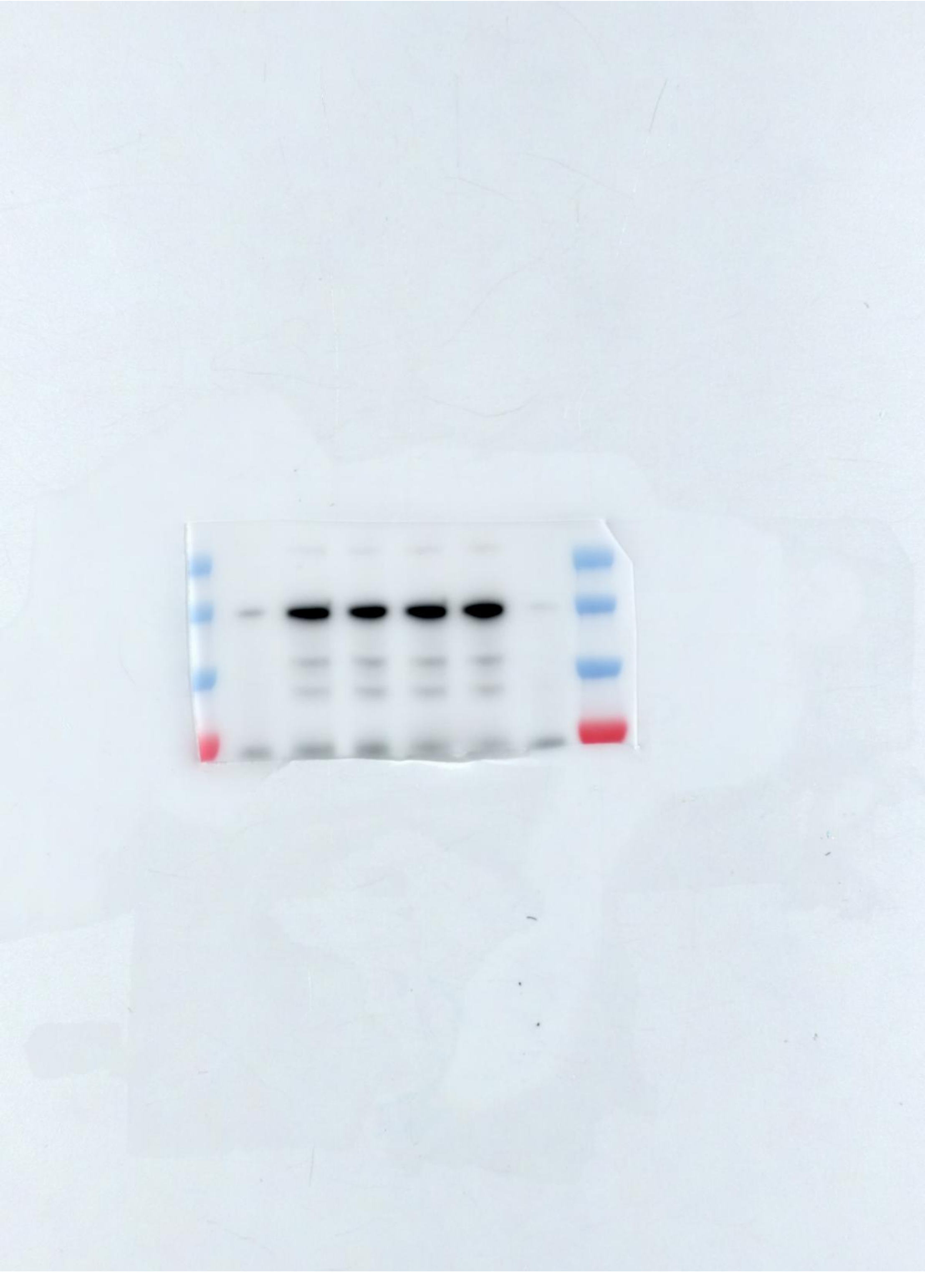

IP-Flag

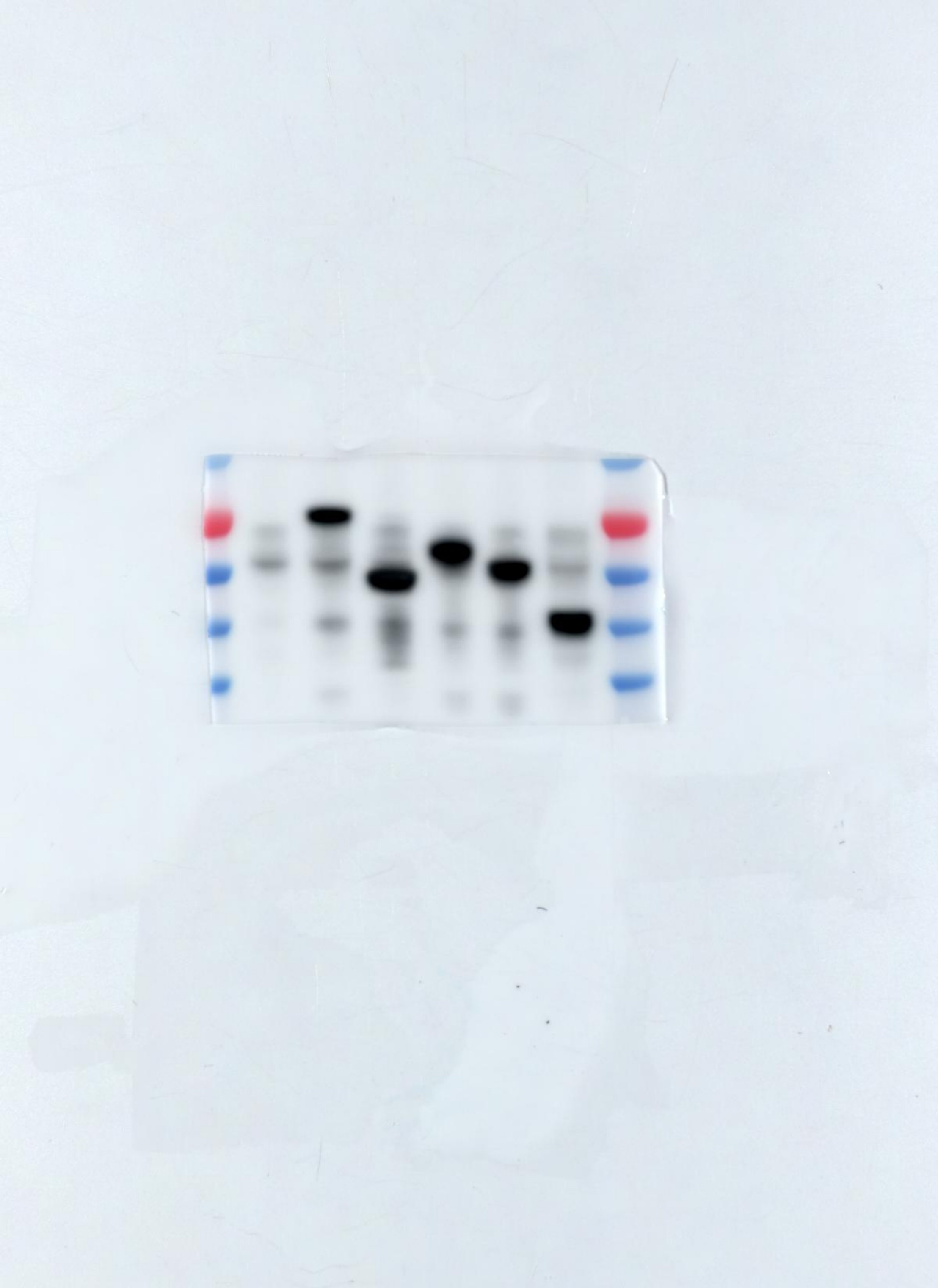

Input-Flag

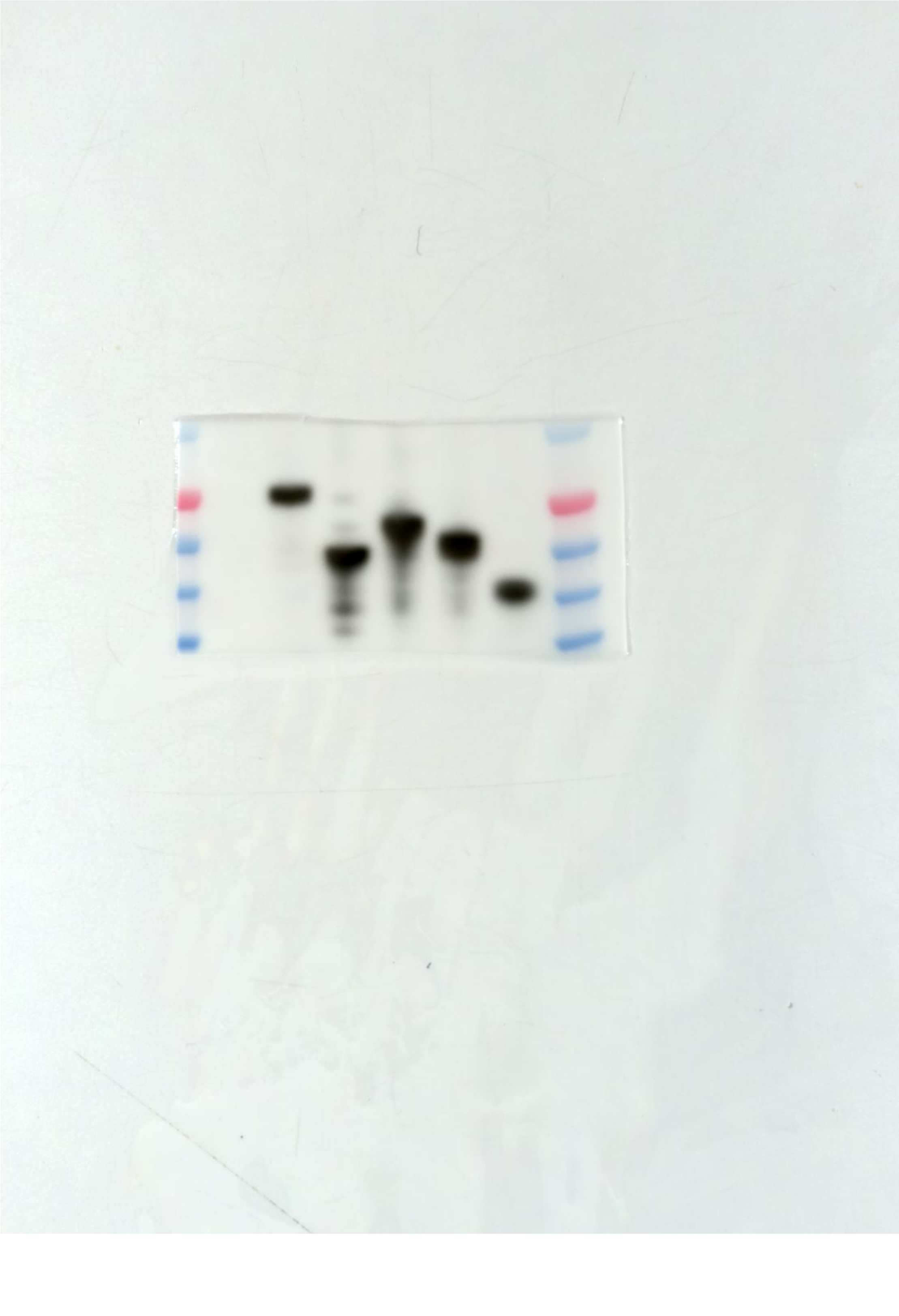

Input-GFP

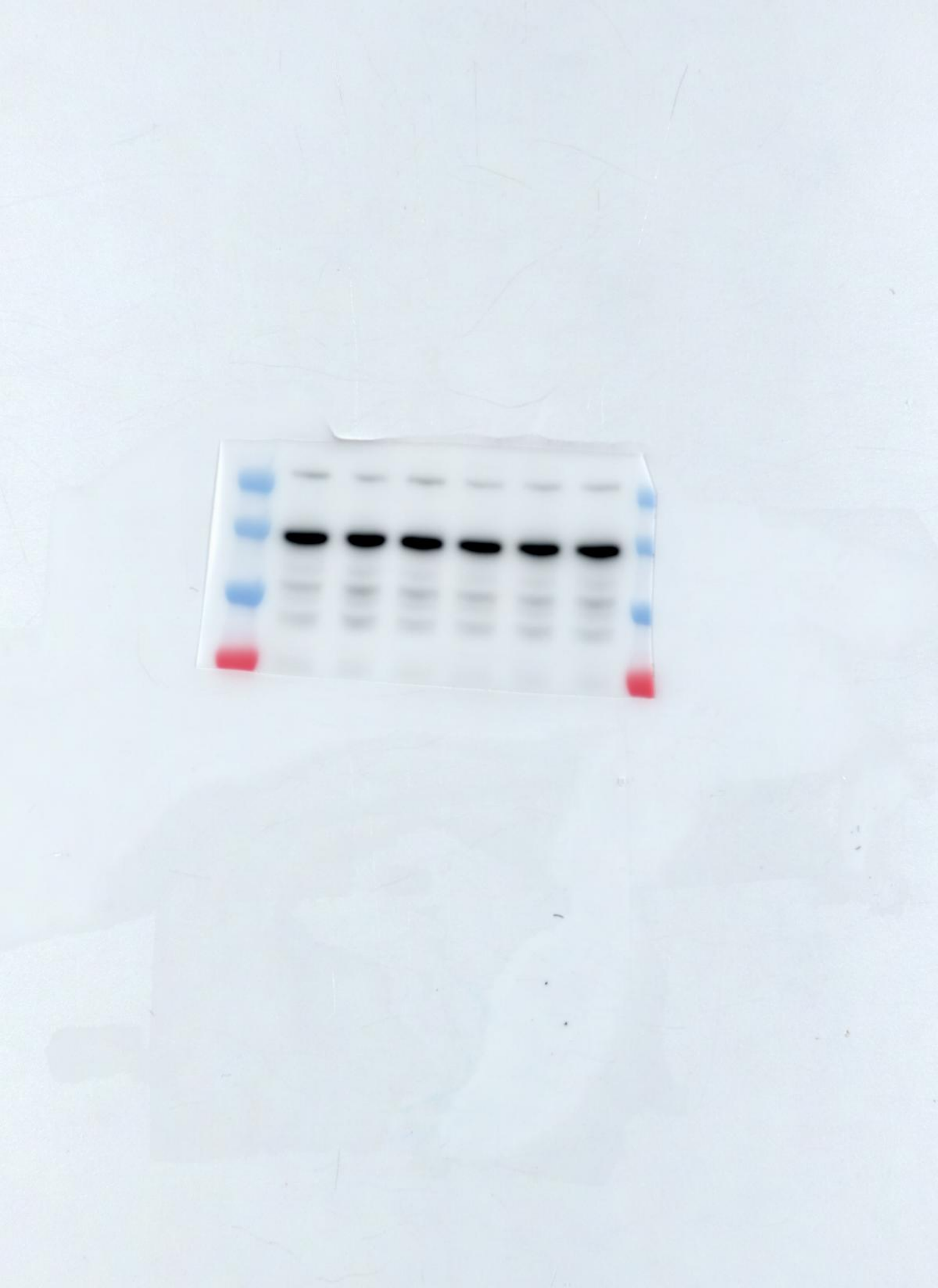

FIgure 7

A

HA-IP

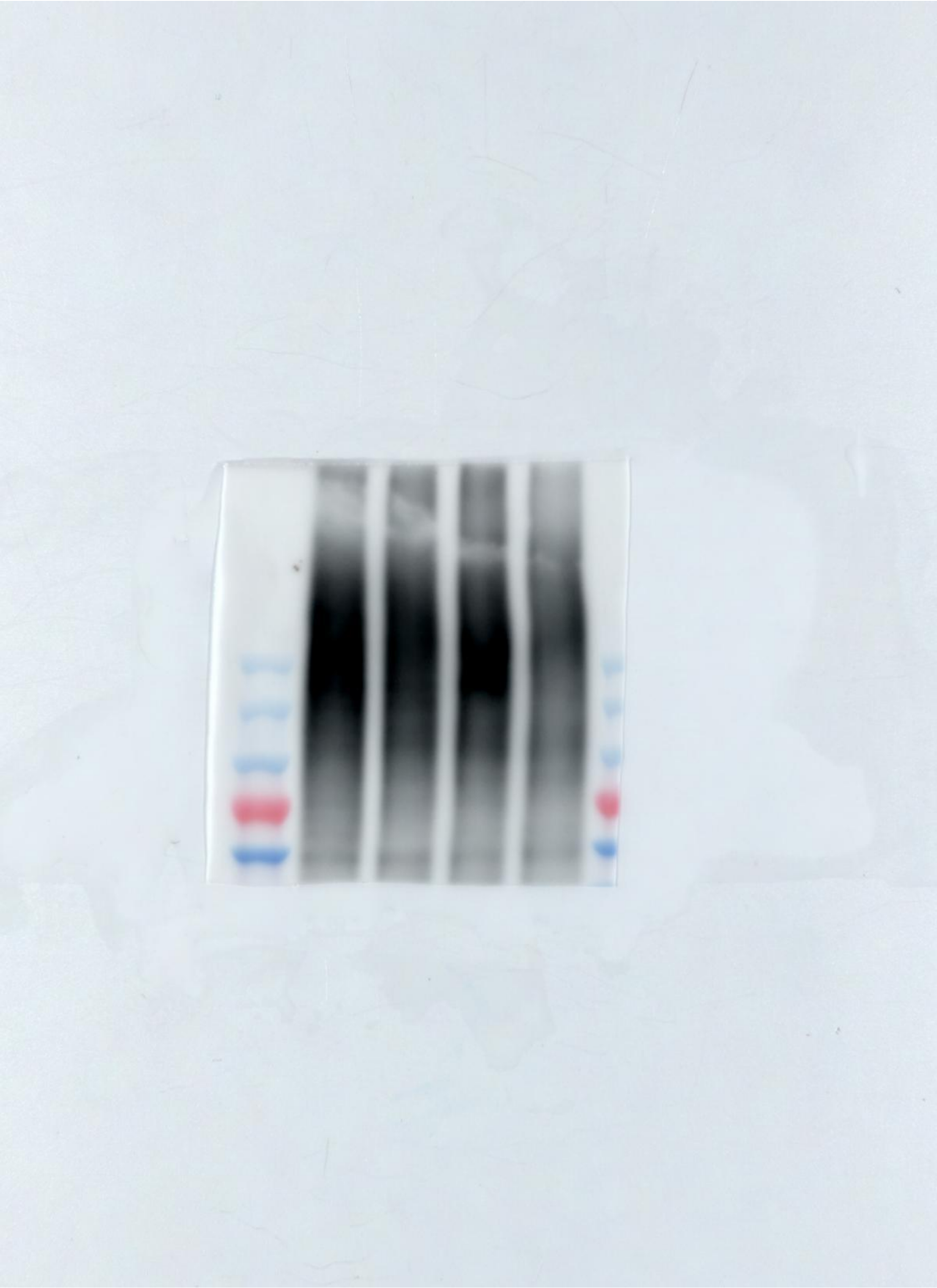

GFP-IP

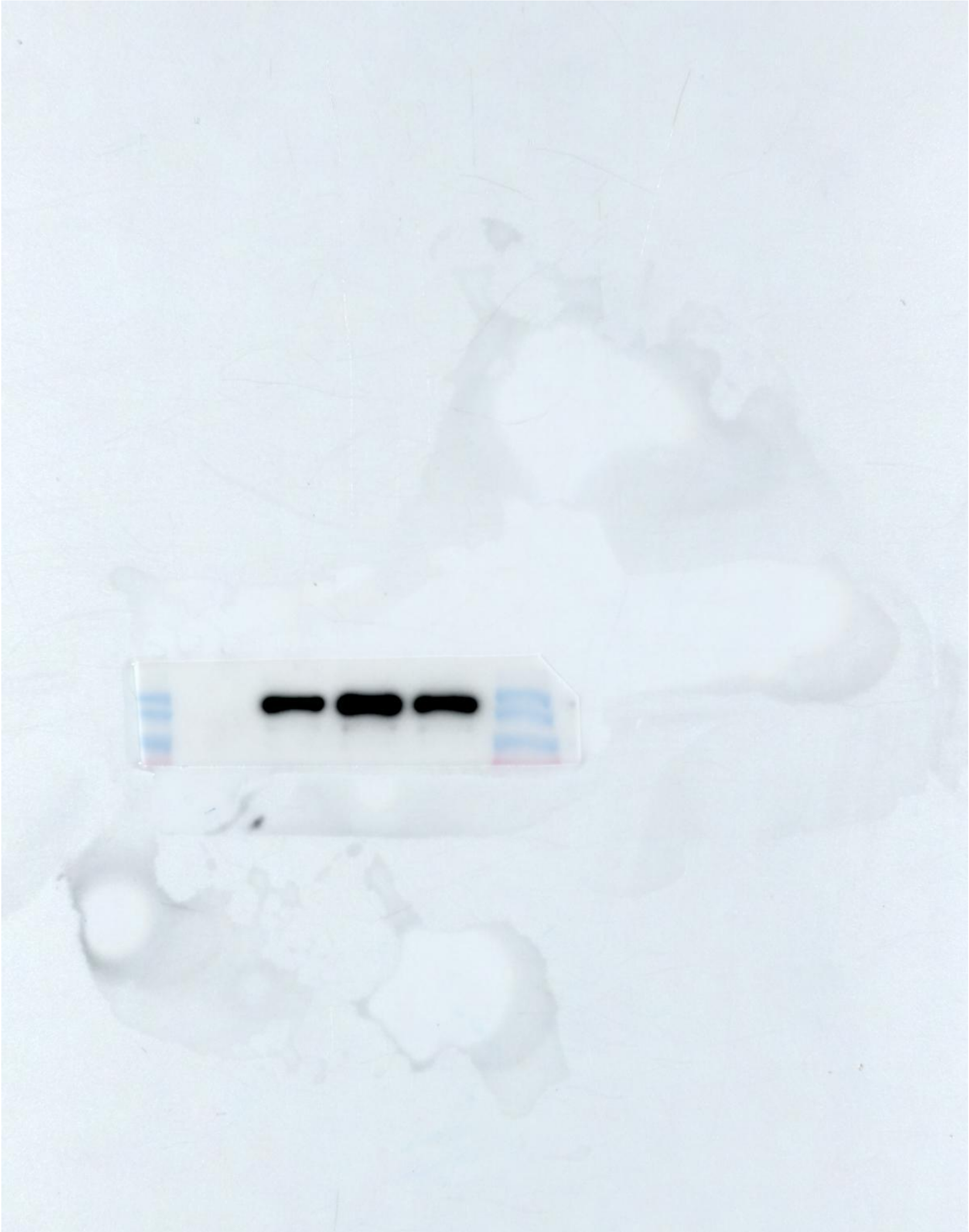

Flag-IP

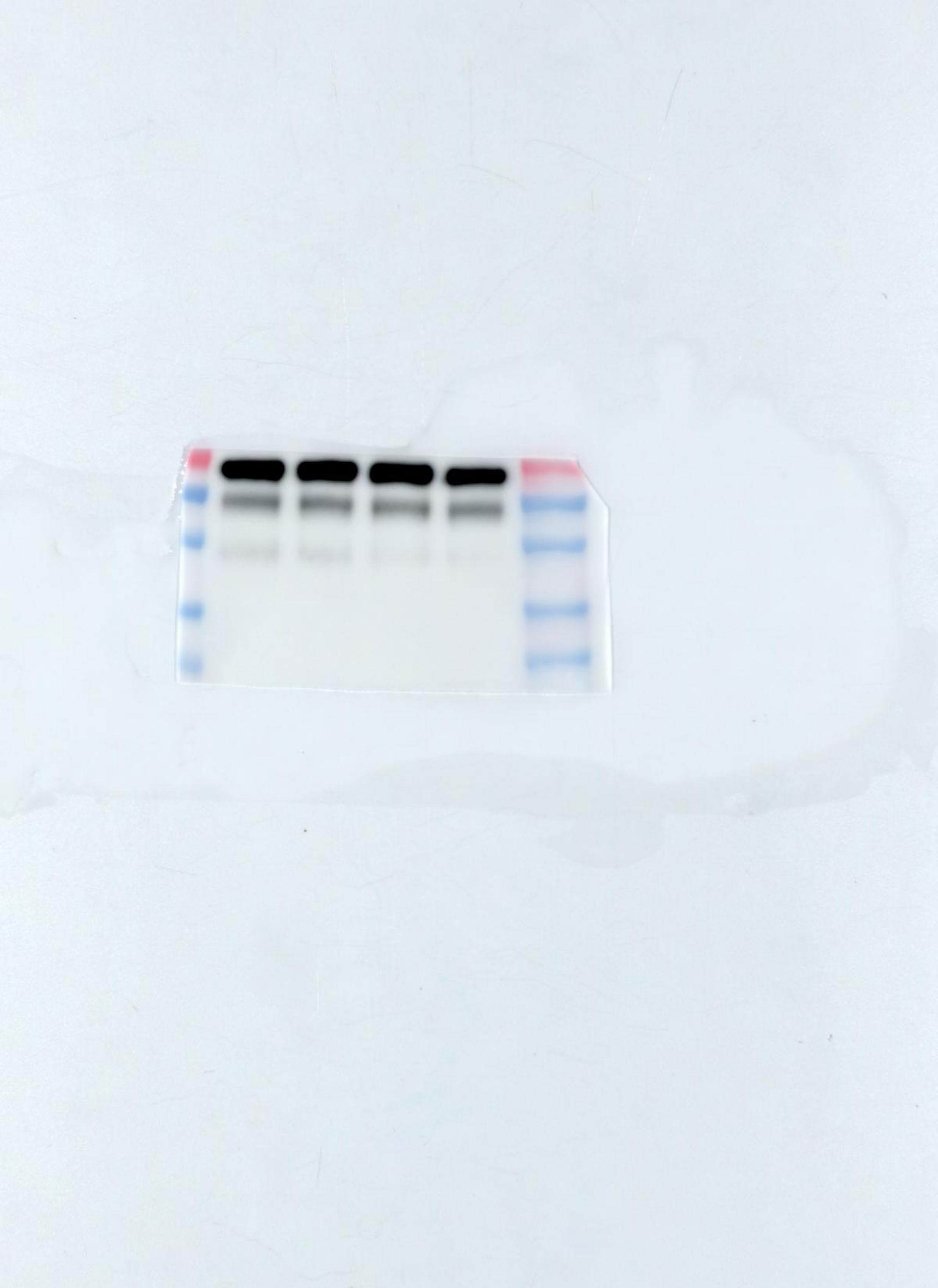

HA-Input

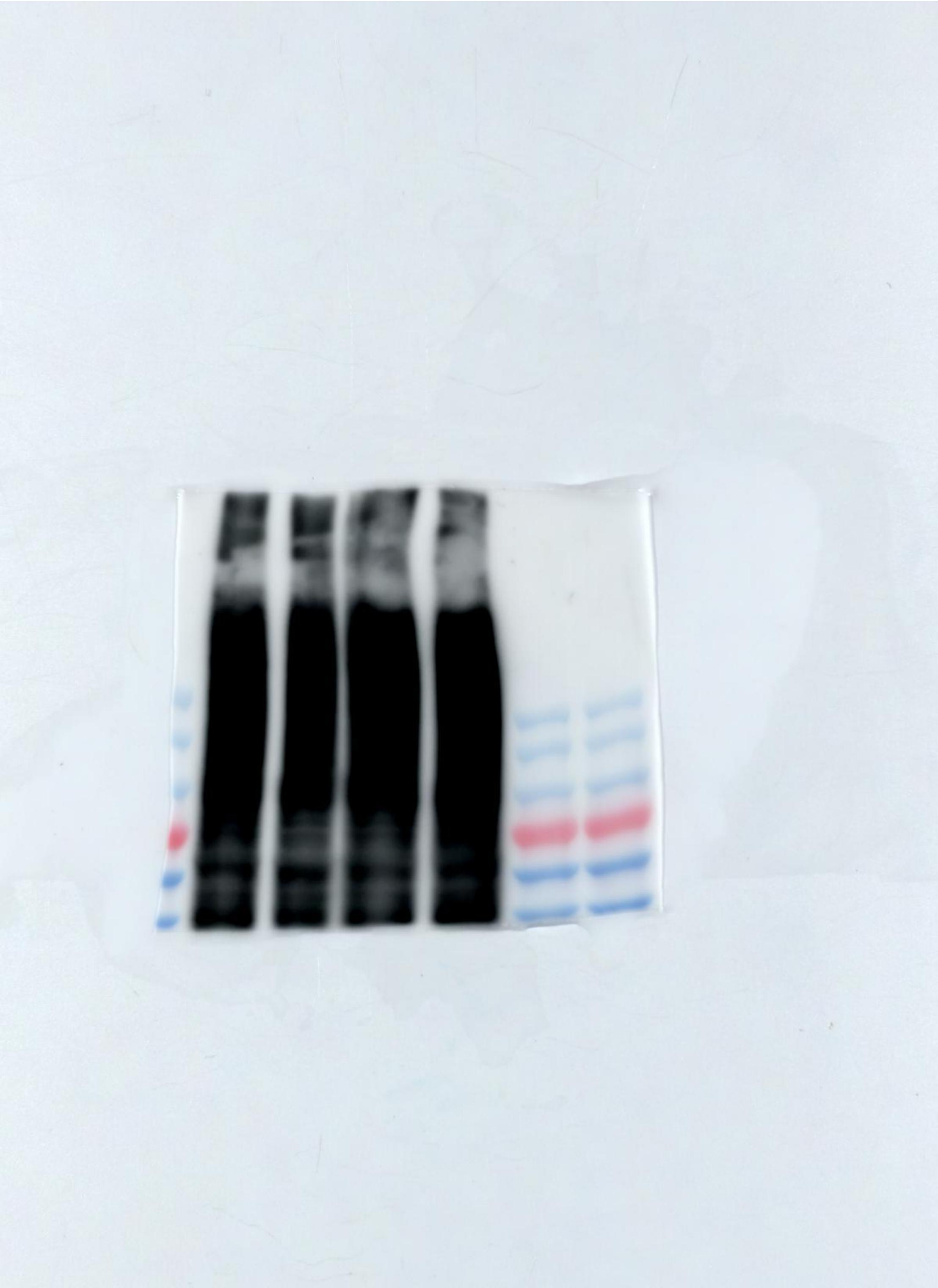

GFP-Input

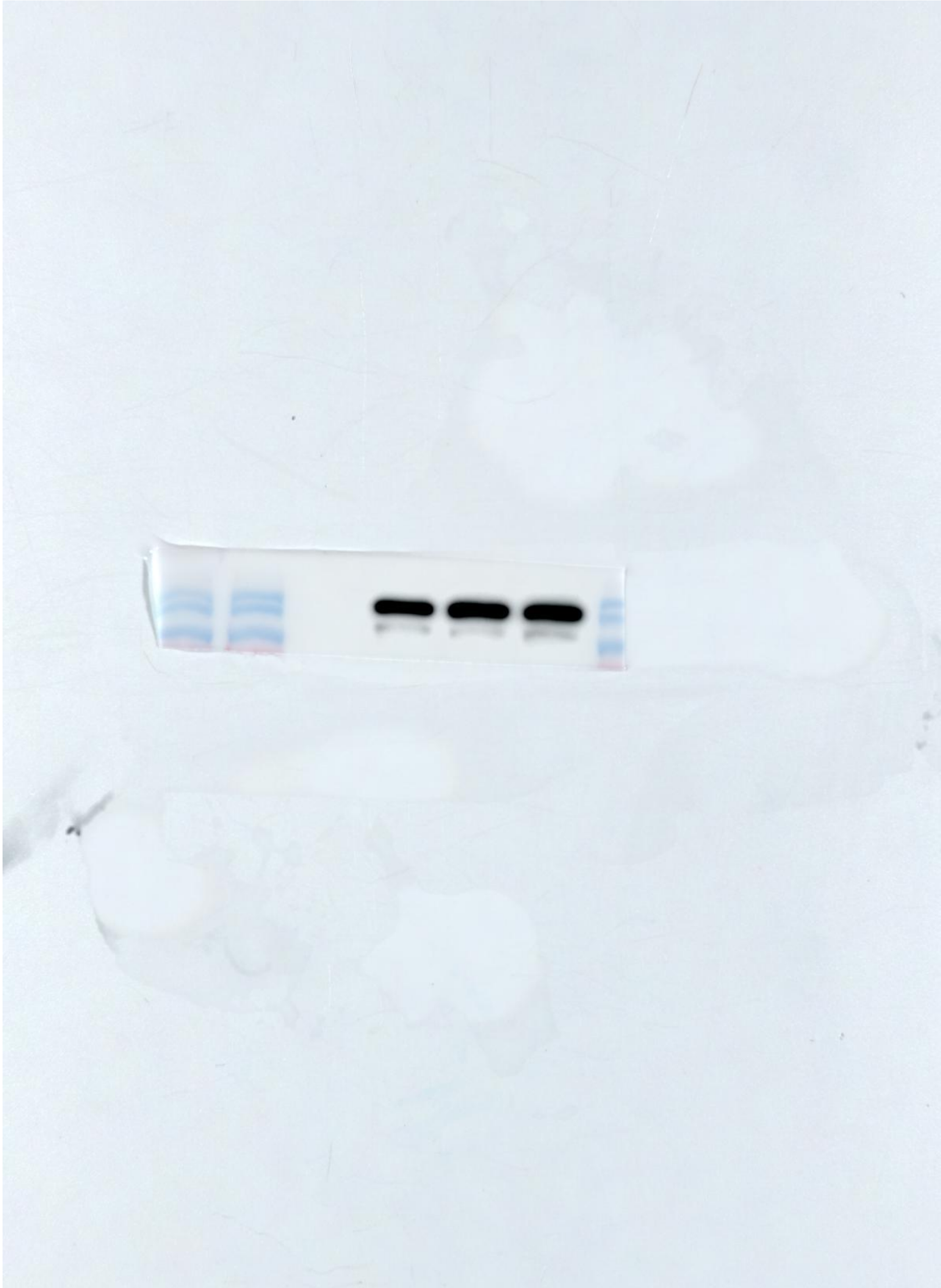

Flag-Input

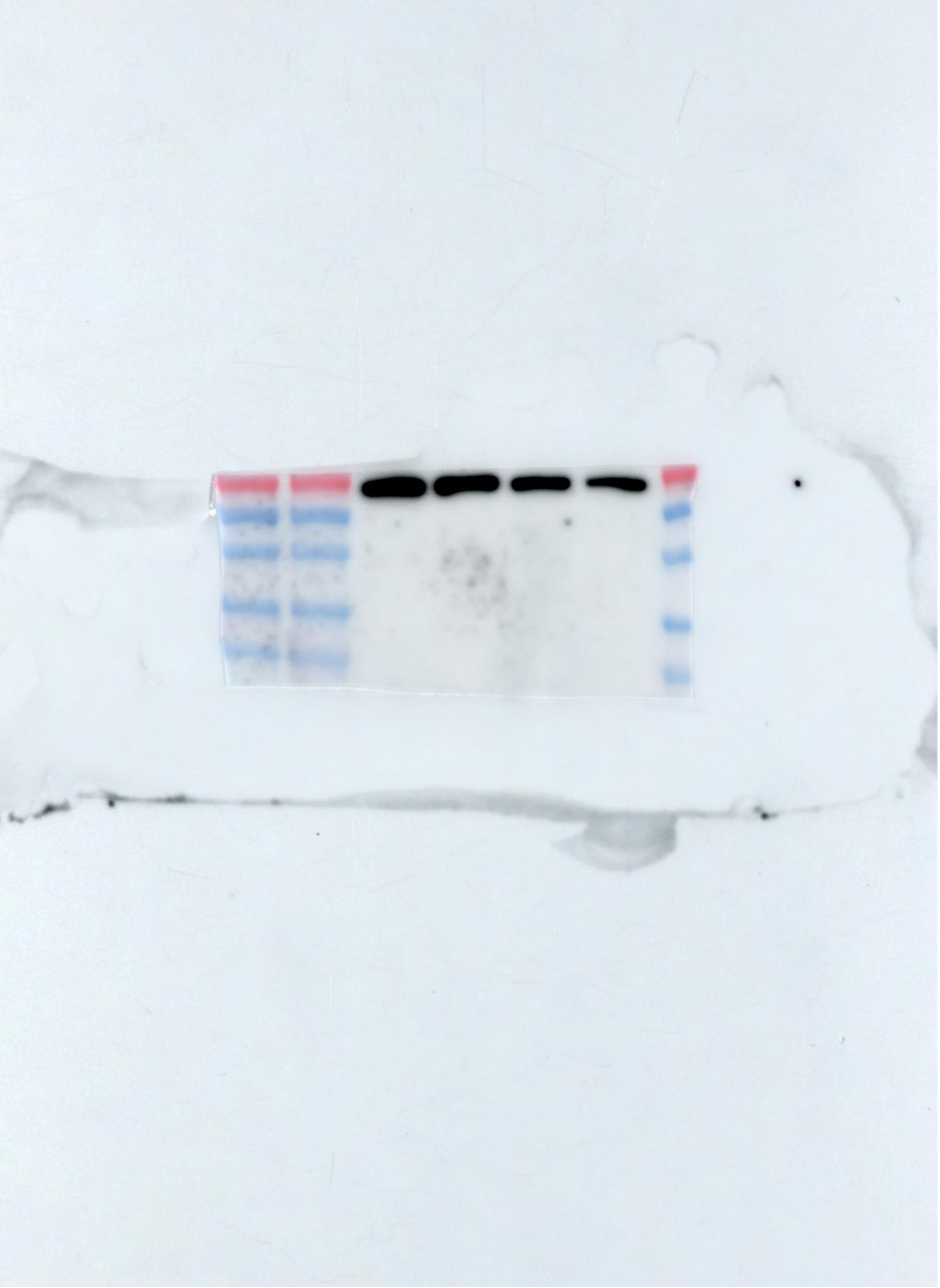

GAPDH-Input

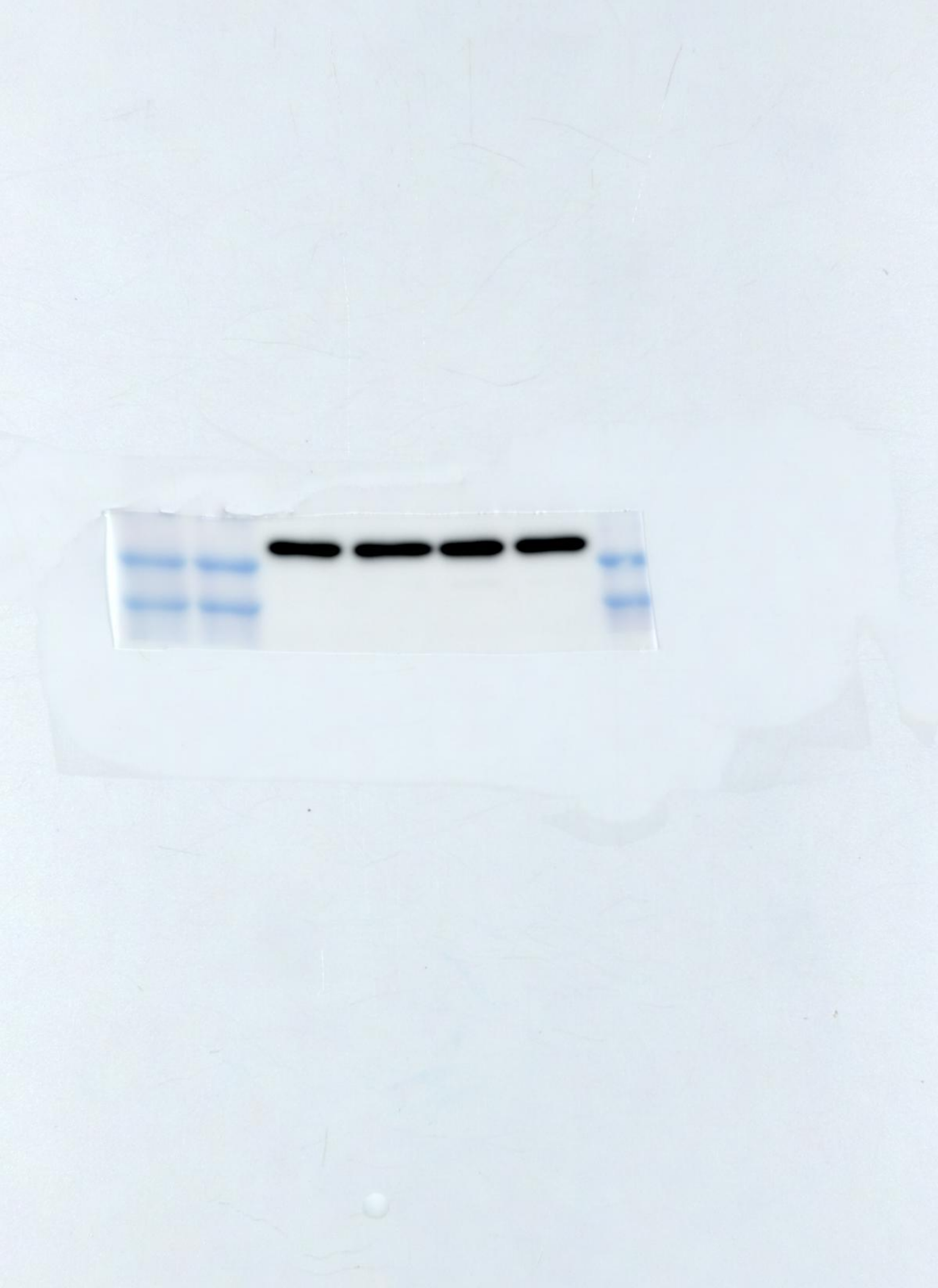

B

HA-IP

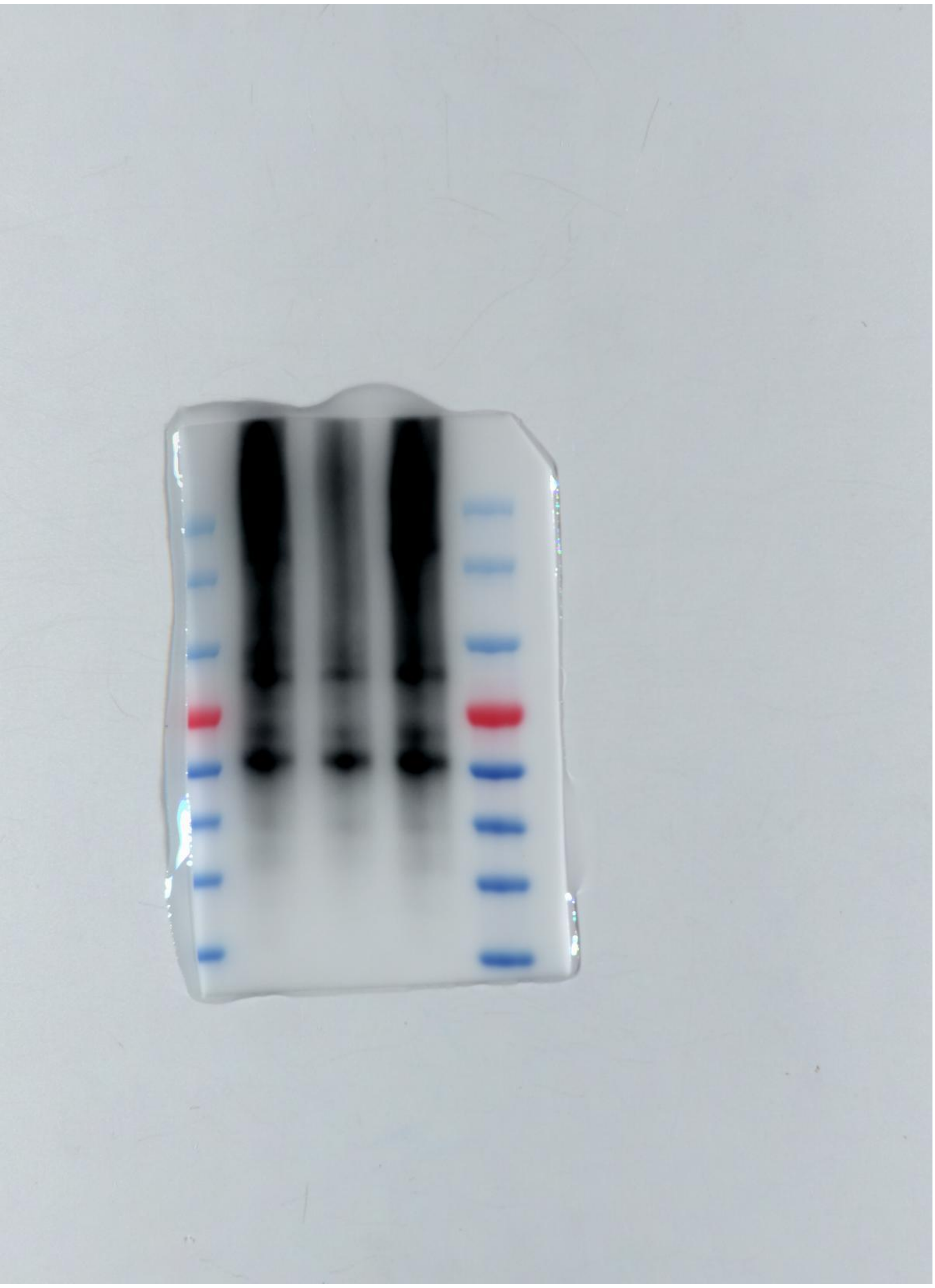

GFP-IP

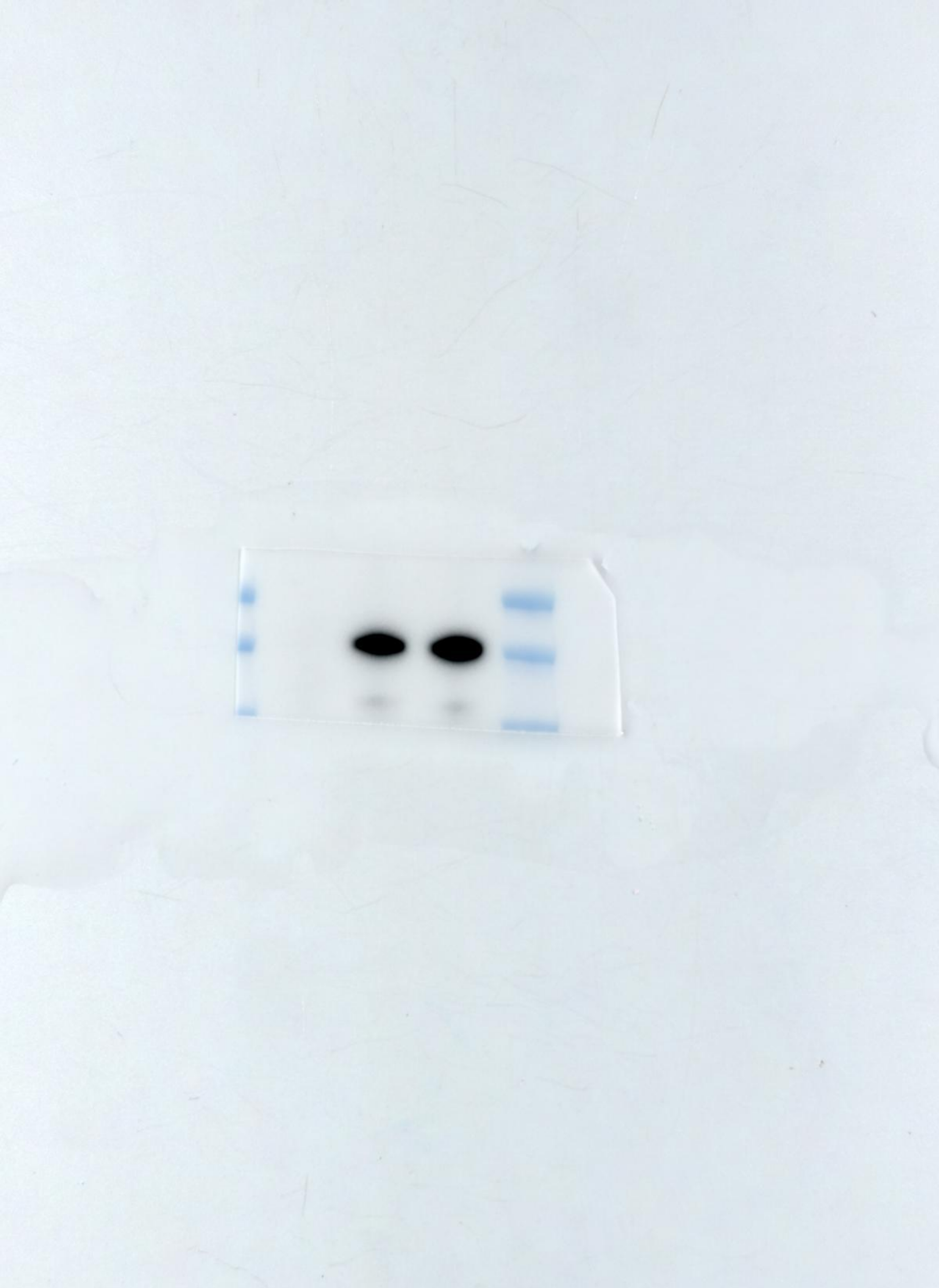

Flag-IP

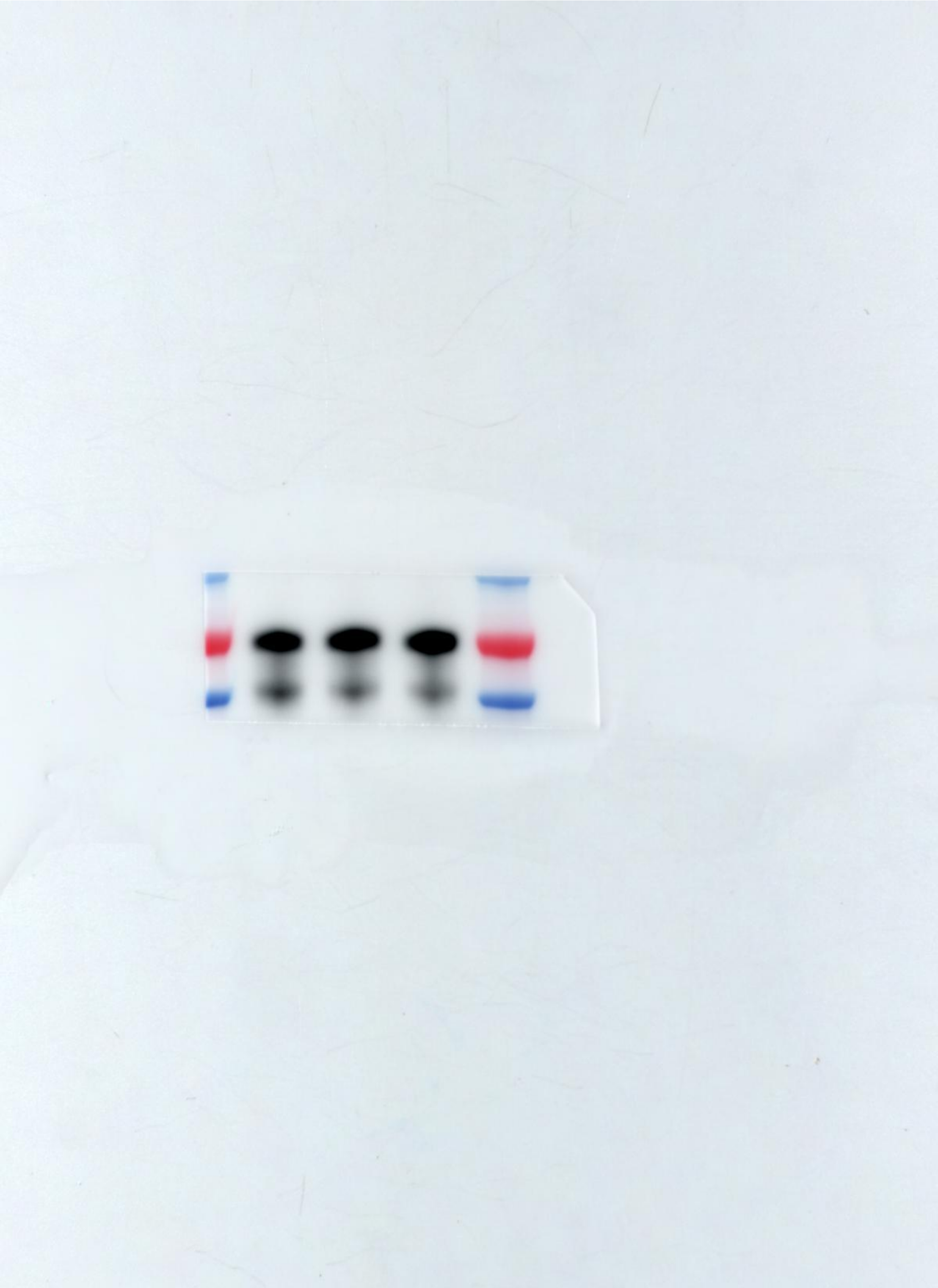

HA-Input

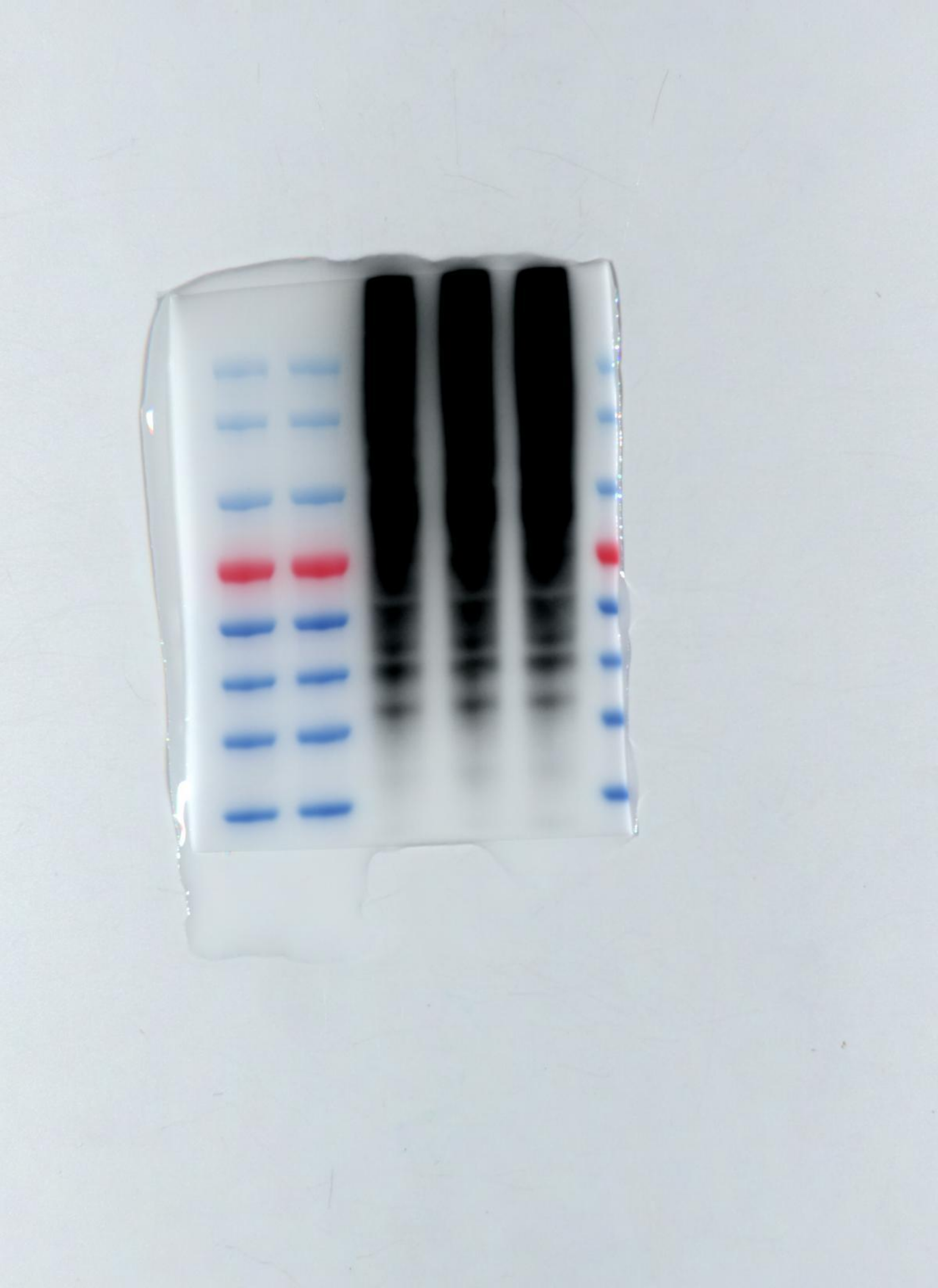

GFP-input

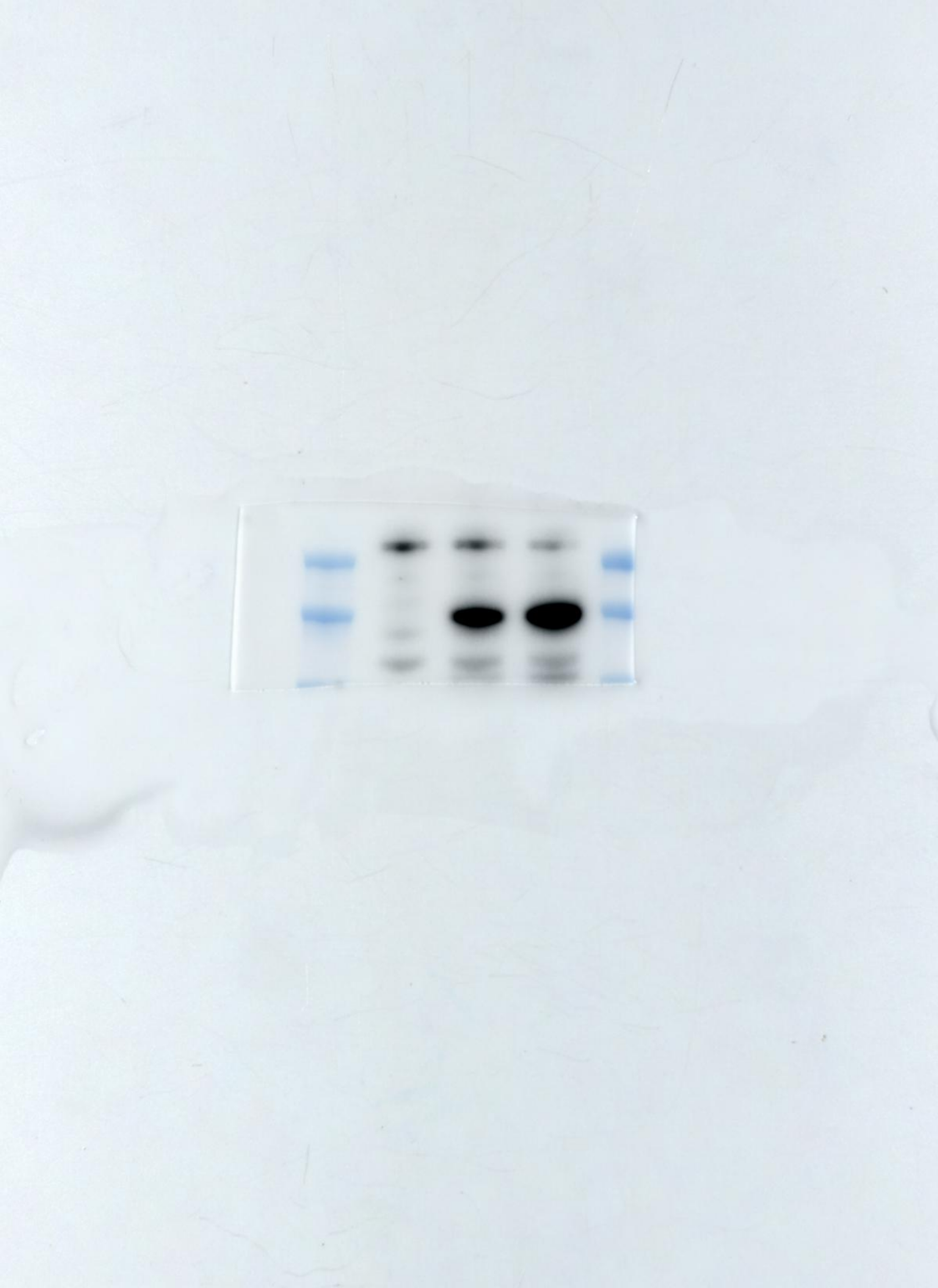

Flag-input

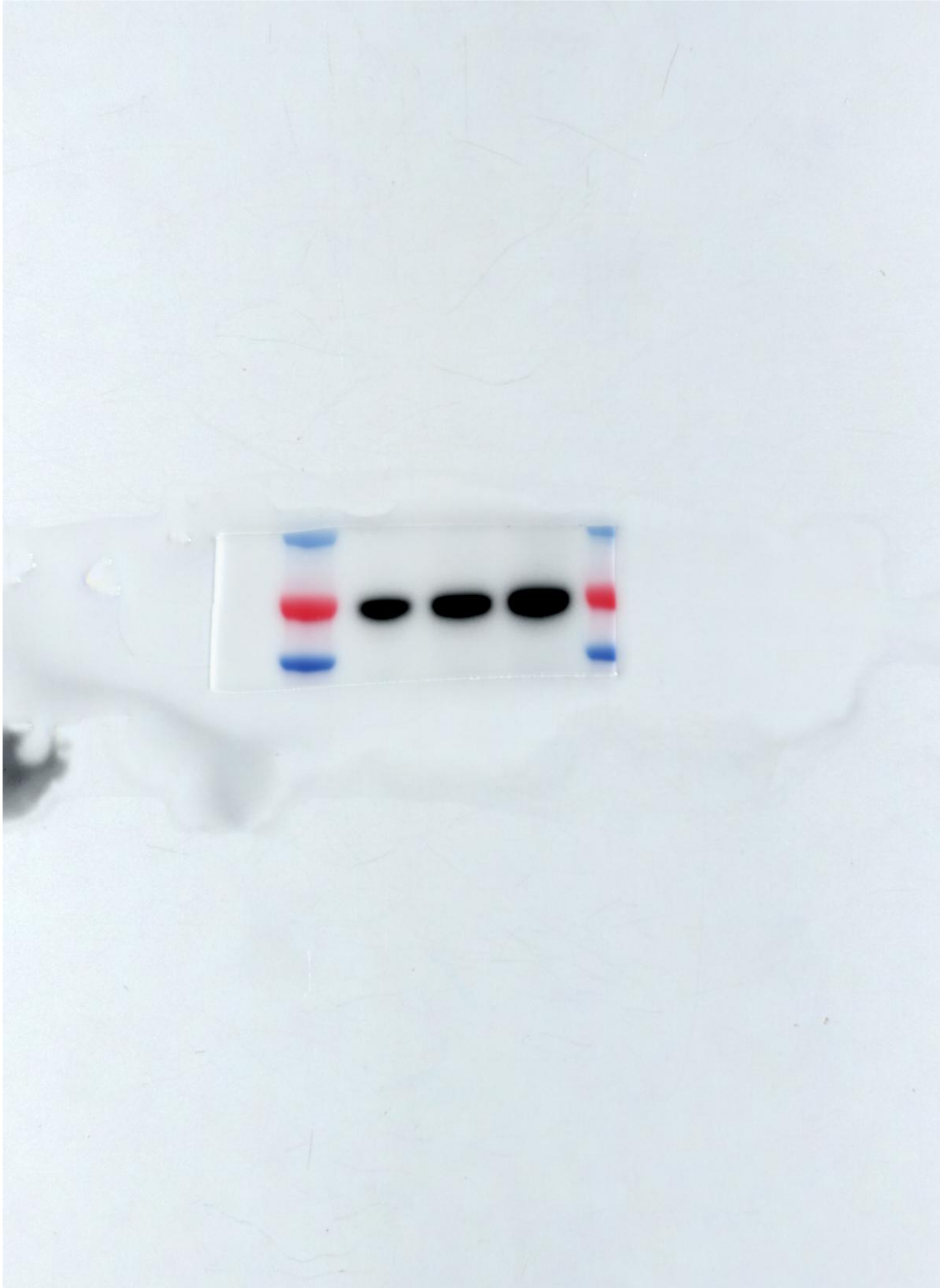

GAPDH-input

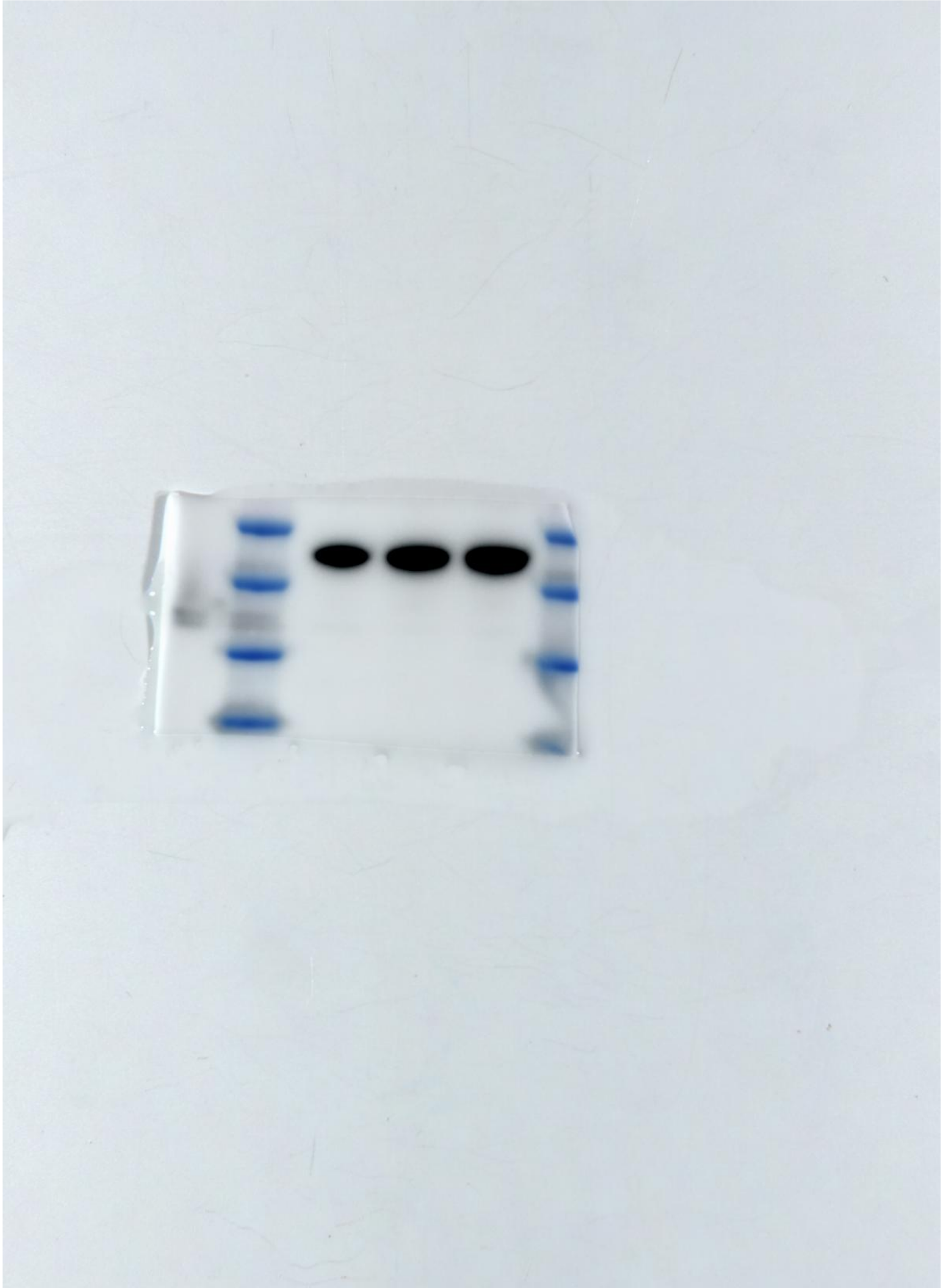

C

IMR32-GFP

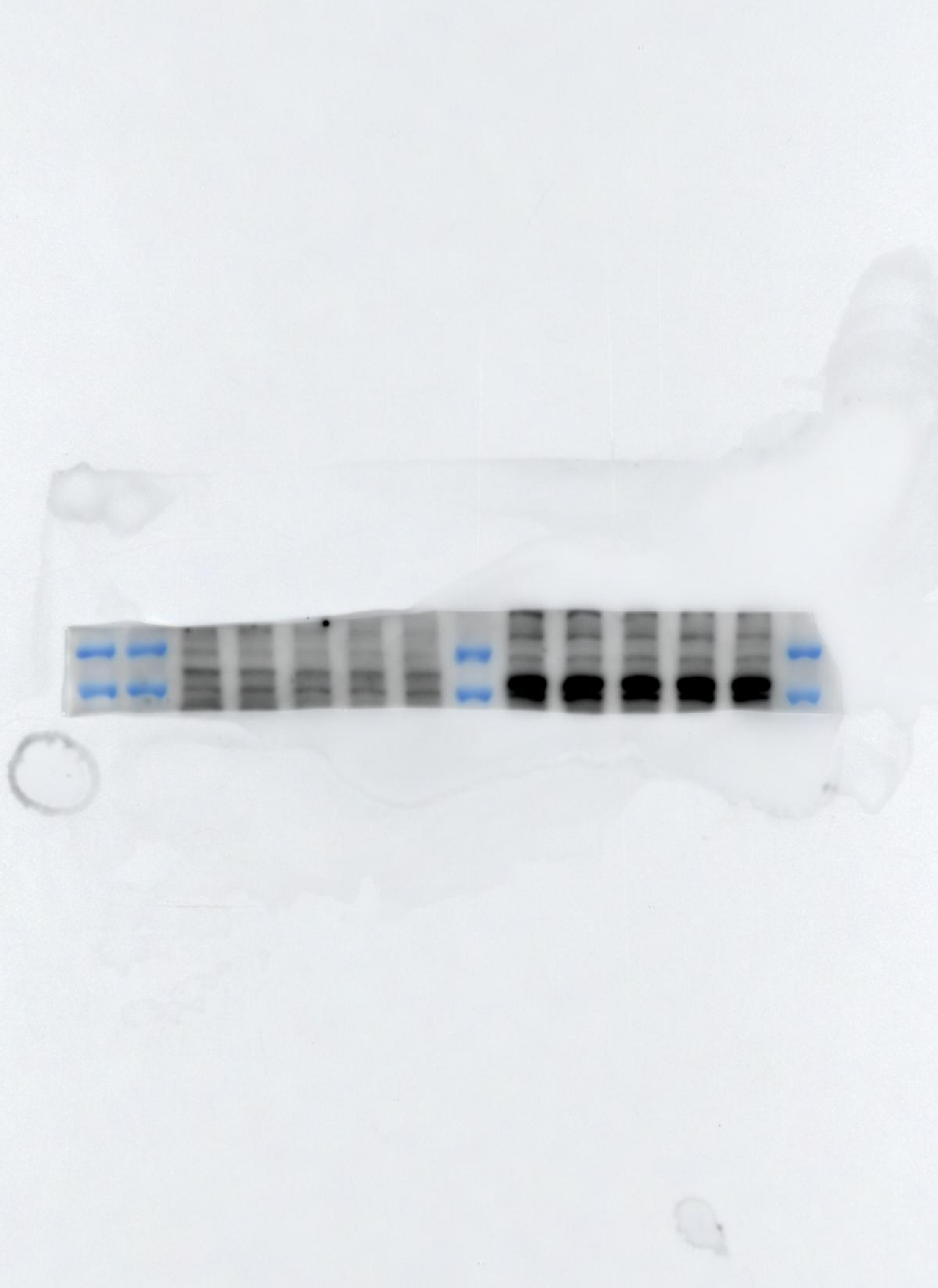

IMR32-N-Myc

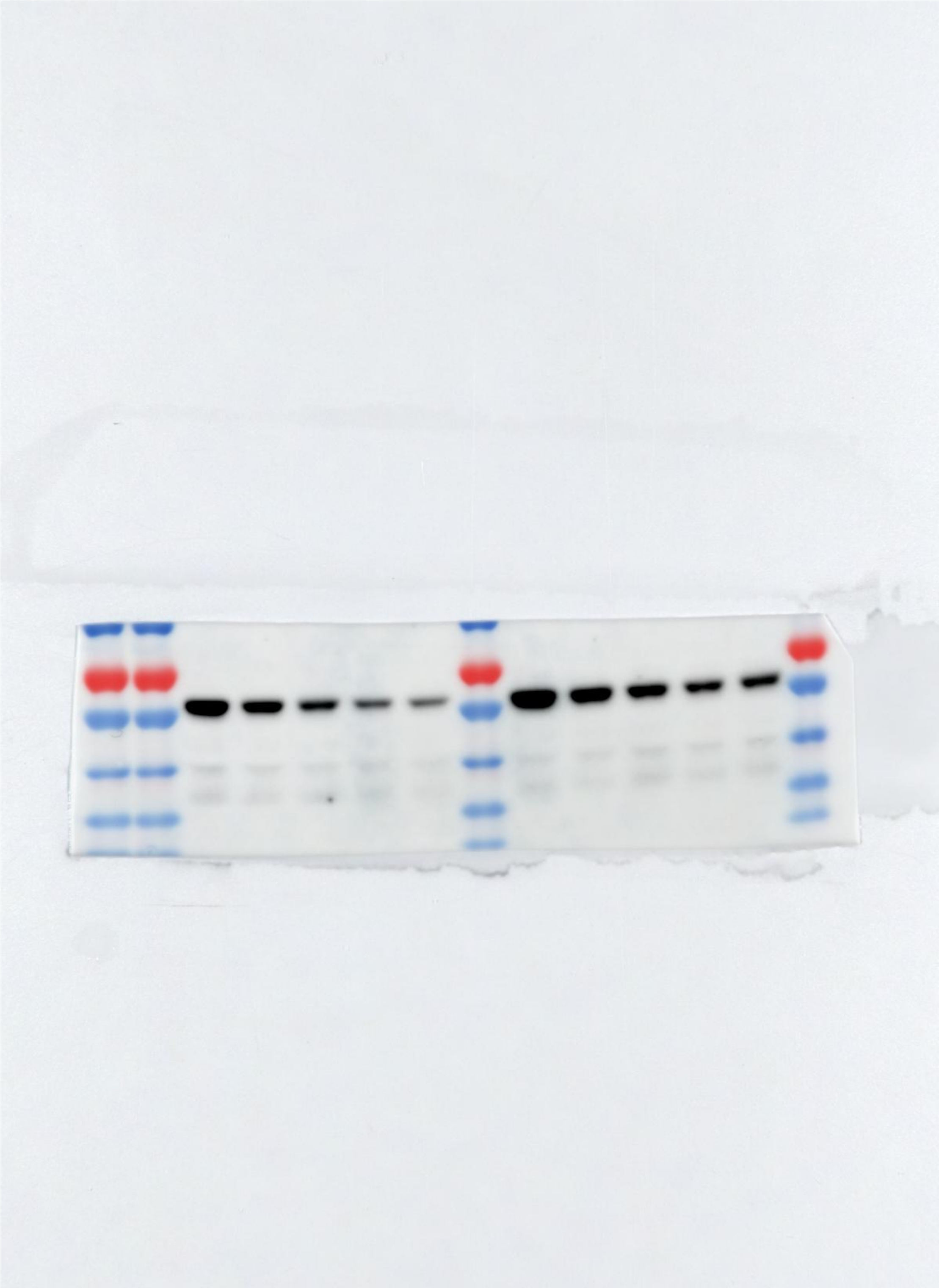

IMR32-GAPDH

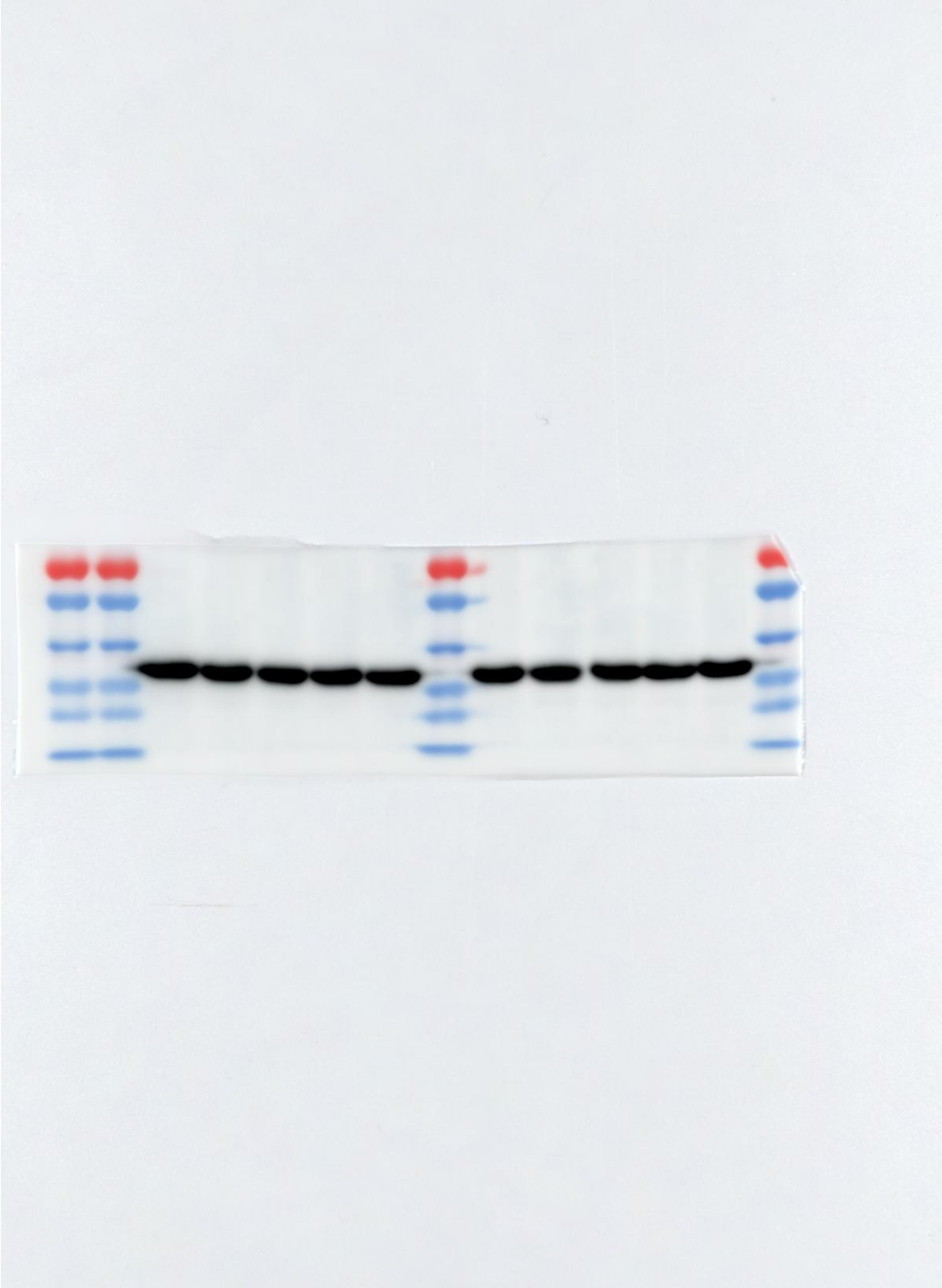

D

BE(2)-M17-GFP

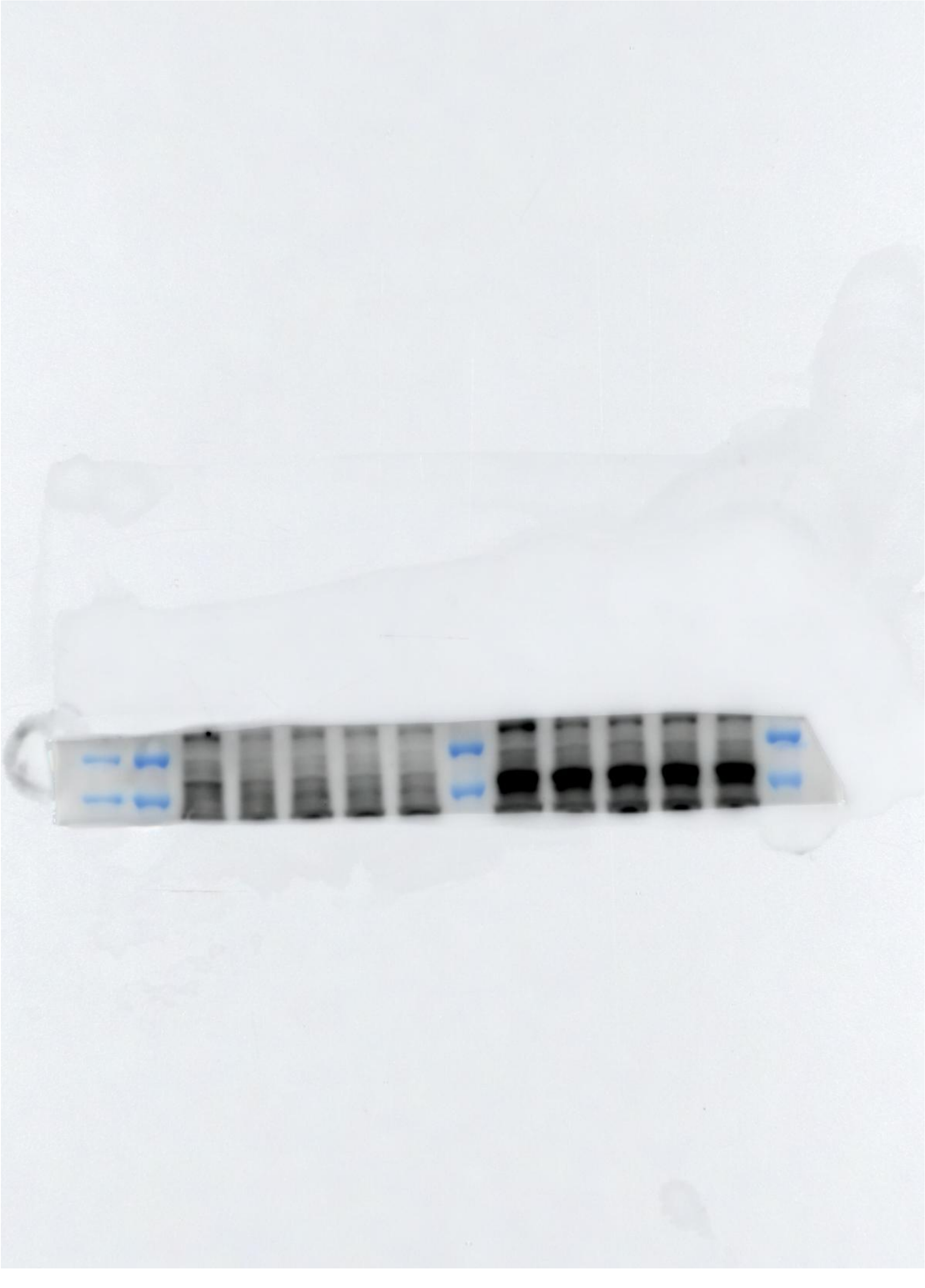

BE(2)-M17-N-Myc

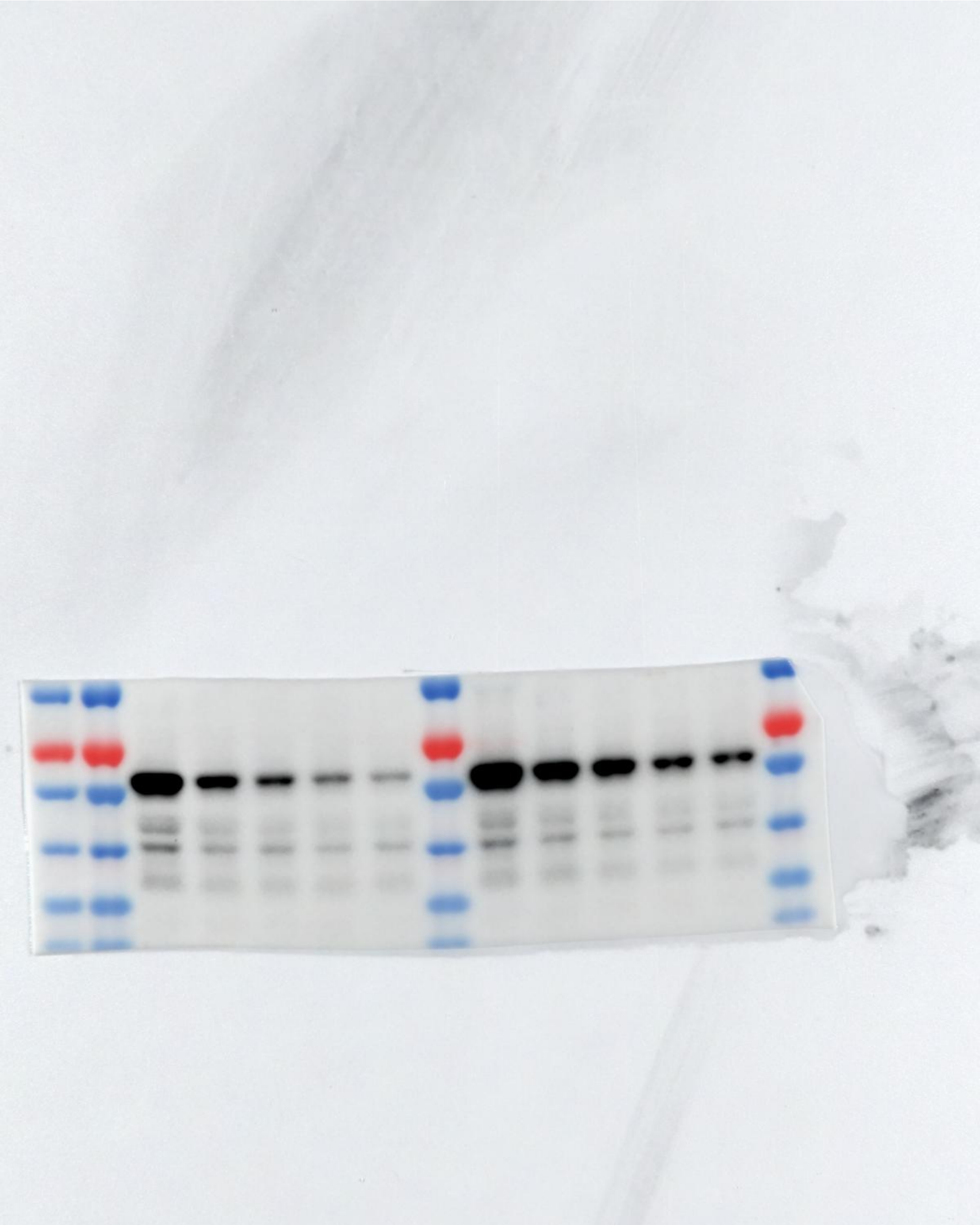

BE(2)-M17-GAPDH

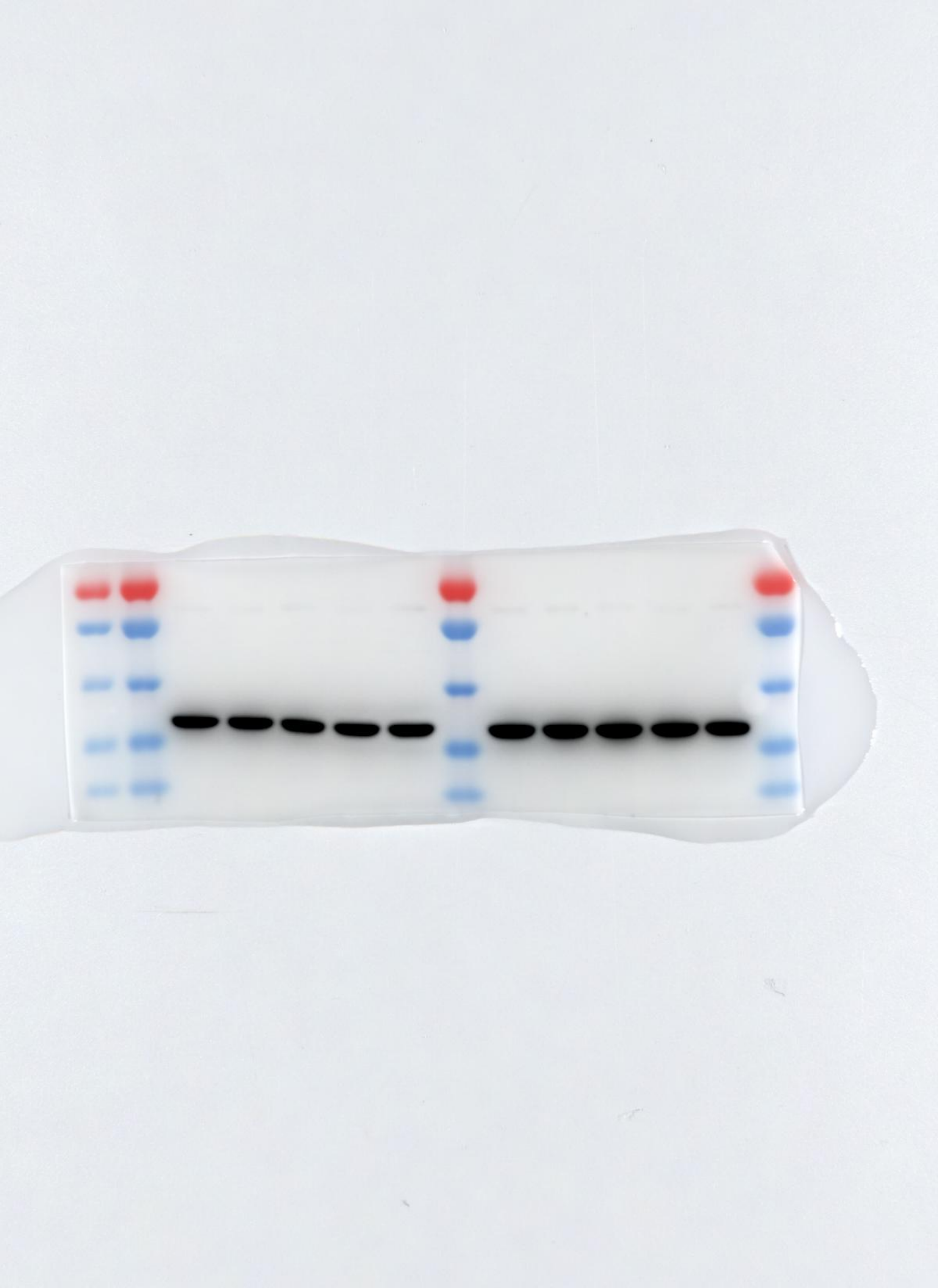

FIgure 8

USP1-IMR-32

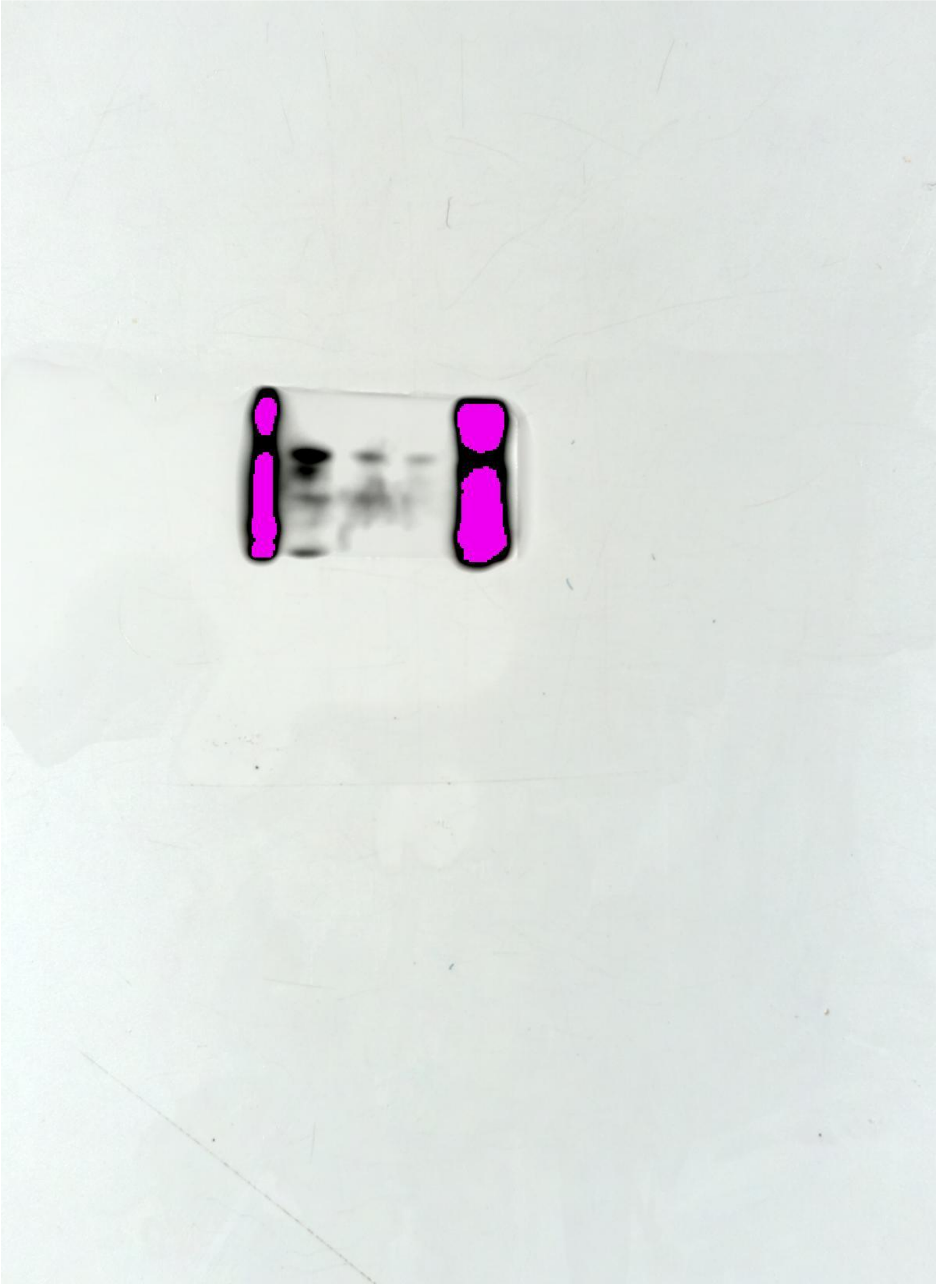

N-Myc-IMR-32

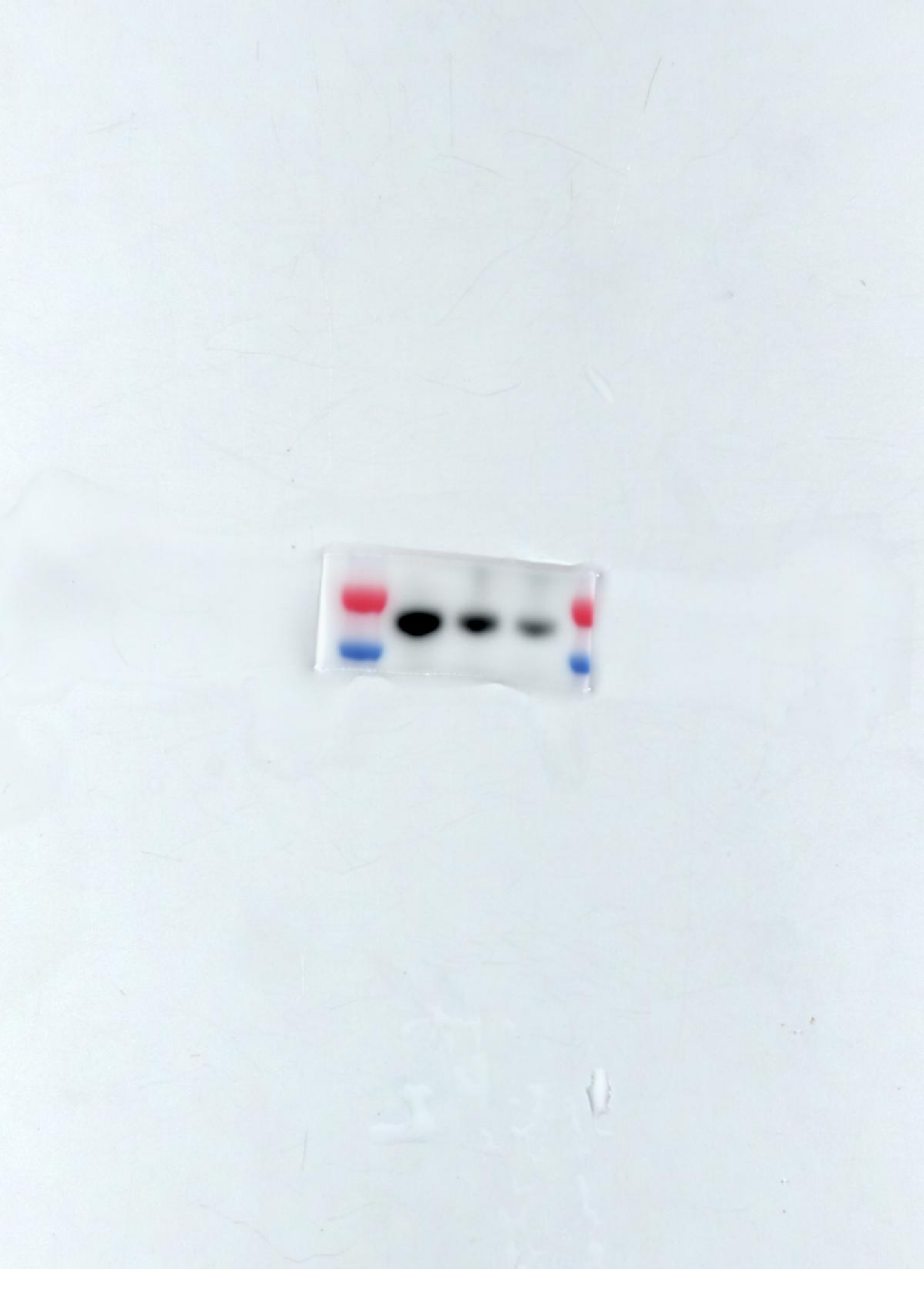

GAPDH-IMR-32

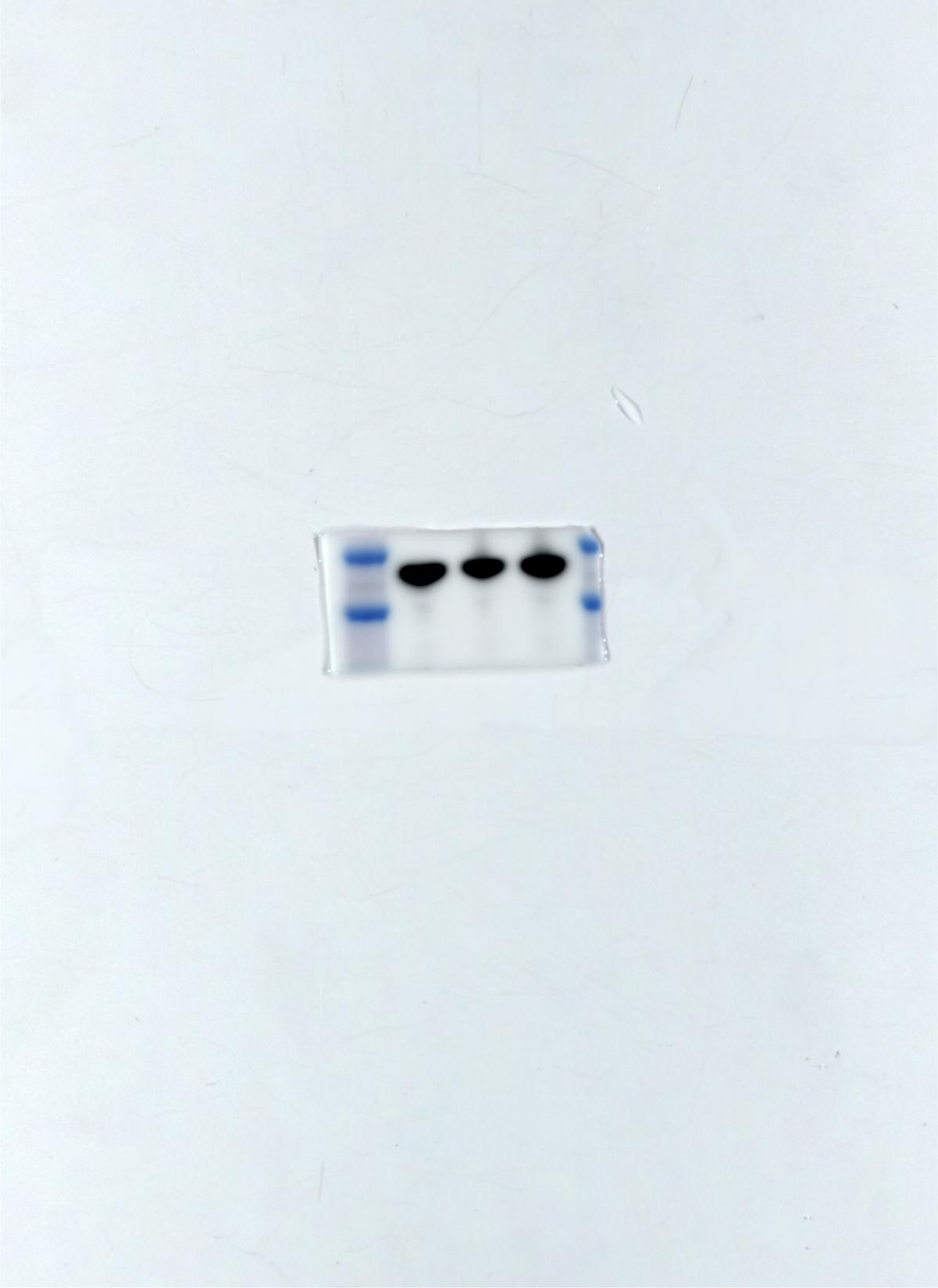

USP1-BE(2)-M17

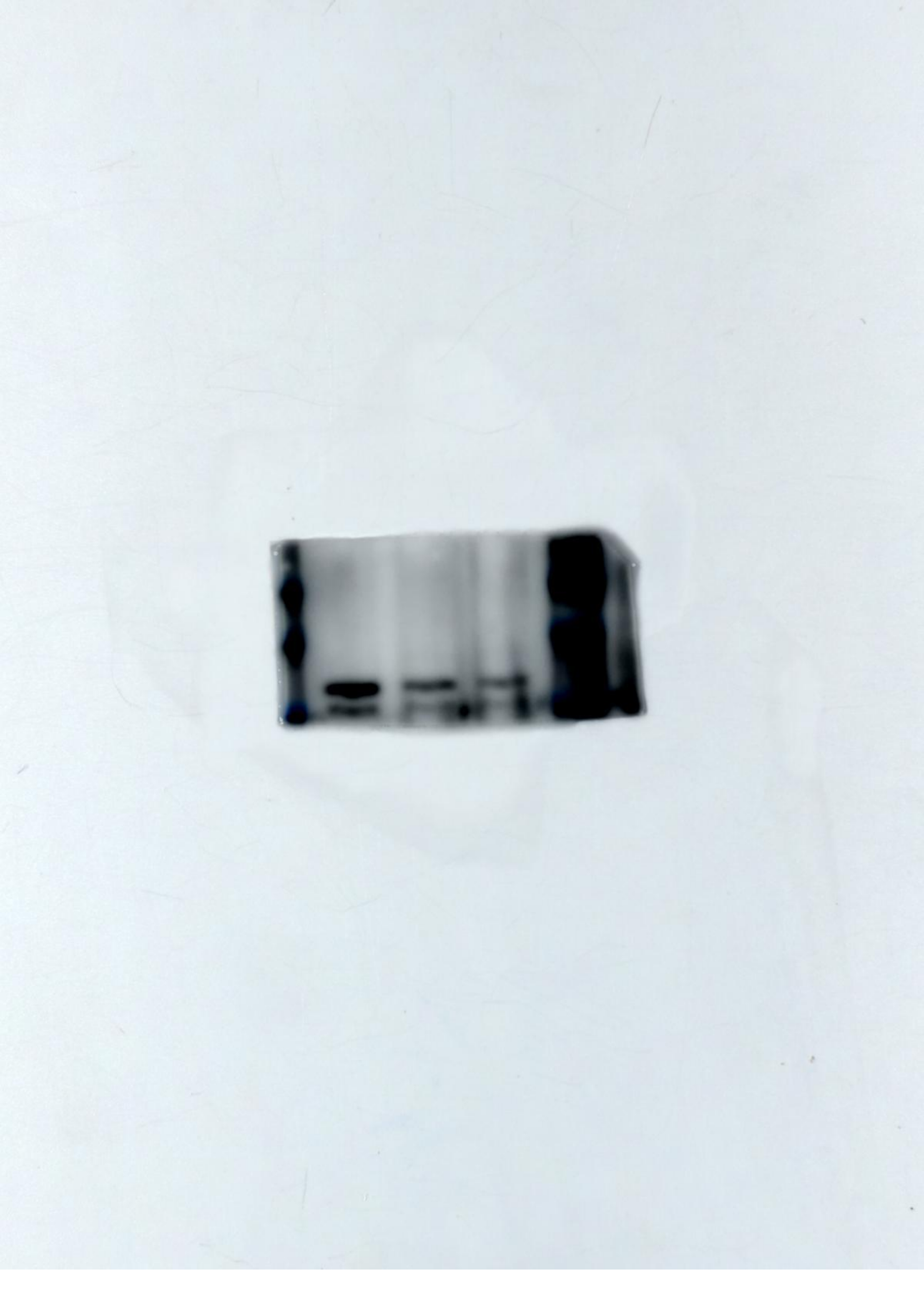

N-Myc-BE(2)-M17

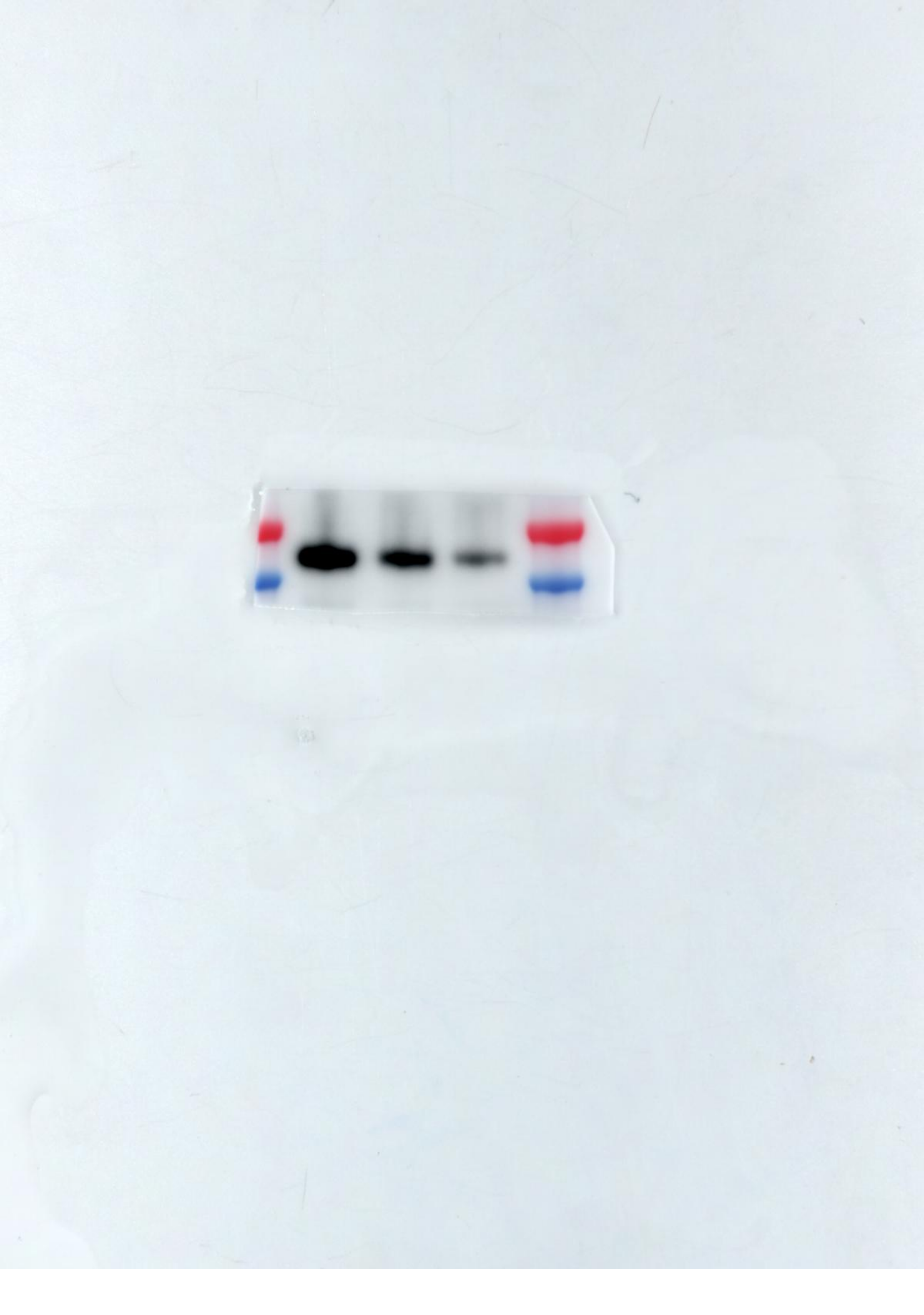

GAPDH-BE(2)-M17

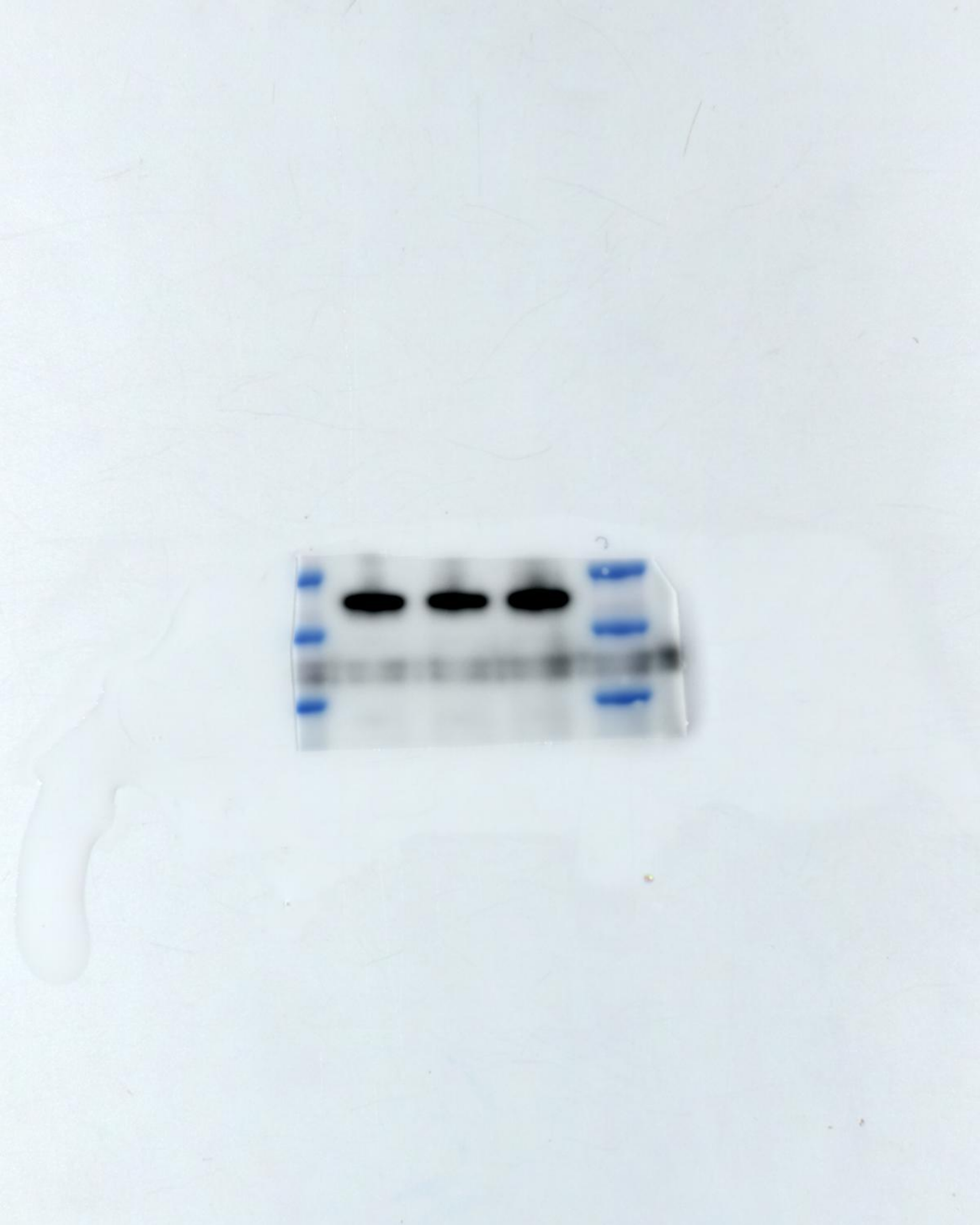

USP1-SK-N-DZ

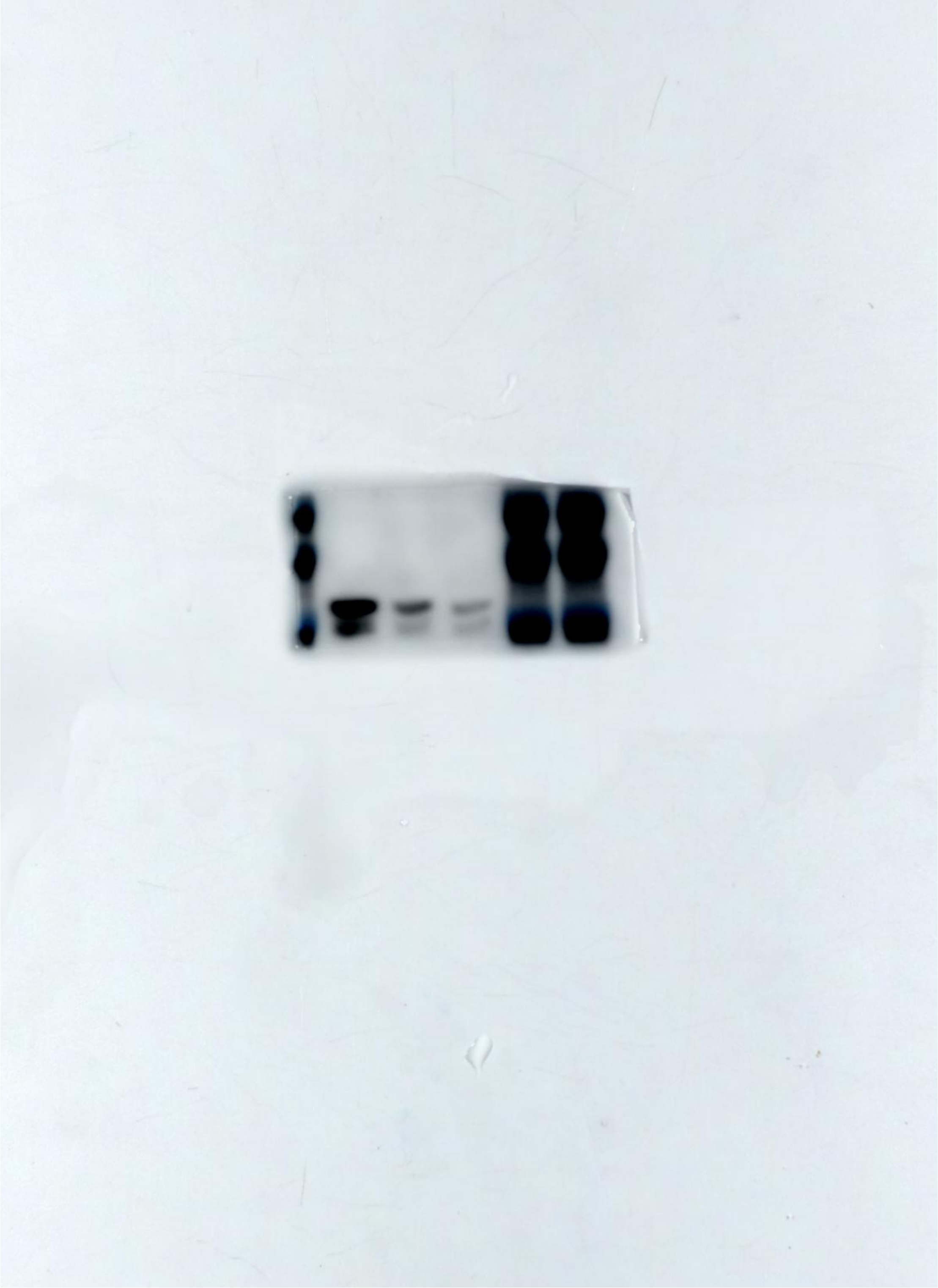

N-Myc-SK-N-DZ

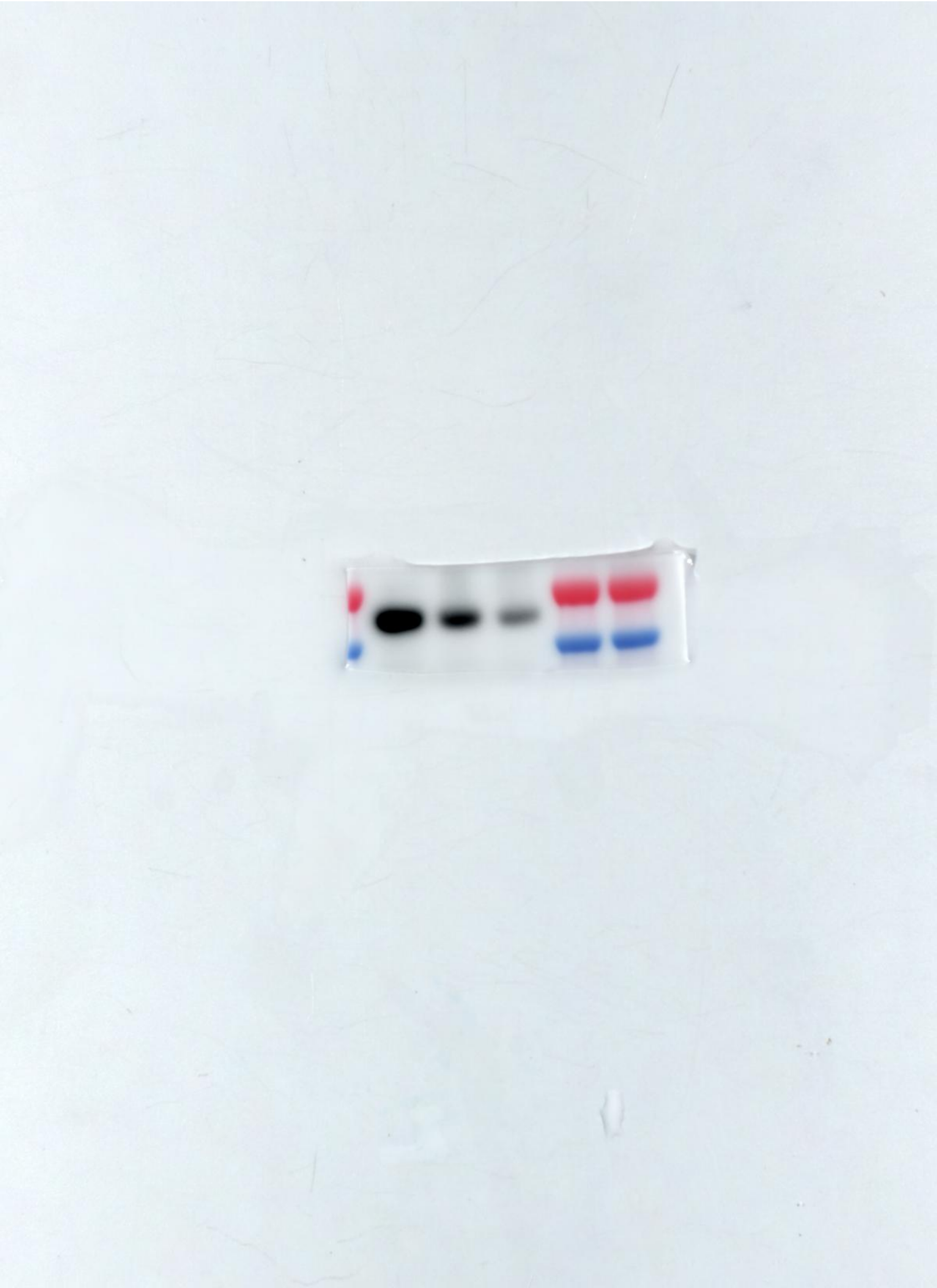

GAPDH-SK-N-DZ

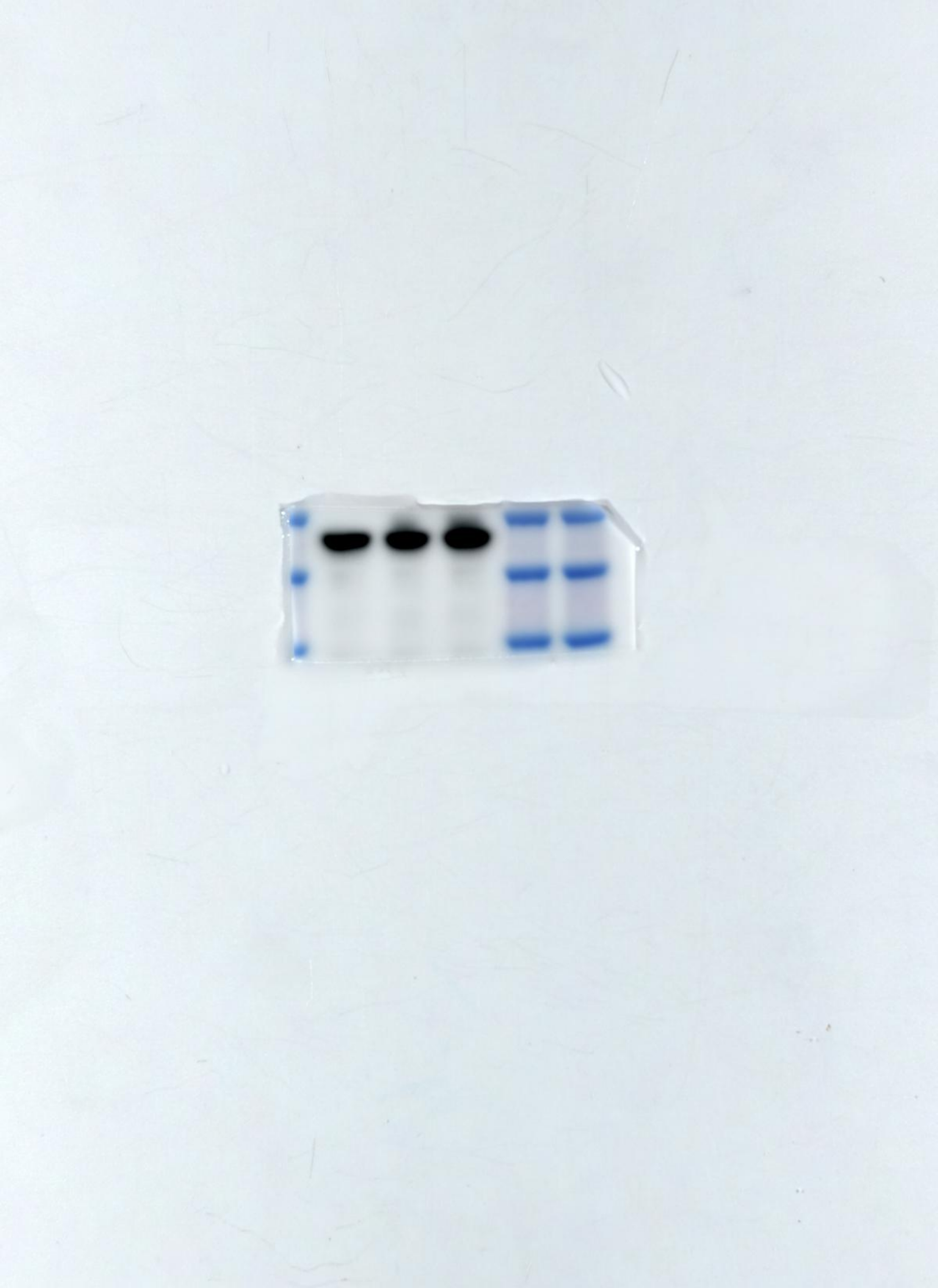

Supplement: Supplementary file 5 — Supplementary Material 5 [file 12967_2026_7844_MOESM5_ESM.pdf]
